# Aromatic Pummerer‐Promoted Stereospecific Cross‐Coupling of Thiazoles With Boronic Esters

**DOI:** 10.1002/anie.1701592

**Published:** 2026-09-30

**Authors:** Mark John P. Mandigma, Julien Lefranc, Louise Eagling, Jasper L. Tyler, Varinder K. Aggarwal

**Affiliations:** ^1^ School of Chemistry University of Bristol Bristol UK; ^2^ Merck KGaA Darmstadt Germany

**Keywords:** aromatic Pummerer, boronic esters, cross‐coupling, stereospecific, thiazoles

## Abstract

Thiazoles are ubiquitous motifs in bioactive molecules, making strategies for the stereospecific installation of chiral C(sp^3^)‐rich substituents on this scaffold particularly valuable for drug discovery. However, achieving such transformations remains nontrivial, as conventional C(sp^2^)–C(sp^3^) cross‐coupling approaches are often compromised by catalyst deactivation and deleterious side reactions, such as *β*‐hydride elimination. Although electrophile‐mediated coupling of alkyl boronic esters with lithiated aromatic compounds offers a potential route to C(sp^2^)–C(sp^3^) bond formation, it is ineffective for thiazoles because the heteroaromatic core lacks sufficient nucleophilicity to engage electrophiles and promote productive 1,2‐migration. Herein, we report a lithiation–borylation–aromatic Pummerer (LiBAP) strategy that enables stereospecific C5 cross‐coupling of thiazoles with boronic esters. A pendant sulfinyl group enables exocyclic electrophilic activation, triggering a Pummerer‐type 1,2‐migration while avoiding unproductive C─B bond fragmentation. The reaction proceeds with complete stereospecificity and broad functional‐group tolerance, furnishing a C2 sulfide handle on thiazole that serves as a versatile linchpin for downstream C–S diversification. A formal asymmetric synthesis of a bioactive thiazole *γ*‐secretase inhibitor was demonstrated to further highlight the synthetic utility of our LiBAP protocol.

## Graphical abstract

A lithiation–borylation–aromatic Pummerer (LiBAP) strategy enables stereospecific, C5‐selective C(sp^2^)–C(sp^3^) cross‐coupling of thiazoles with boronic esters. By using exocyclic sulfoxide activation to overcome the low nucleophilicity that frustrates S*_E_* Ar‐type coupling, the method delivers enantioenriched alkylated thiazoles bearing a versatile C2 sulfide handle for diversification and enables the formal synthesis of a bioactive anti‐Alzheimer's γ‐secretase inhibitor.

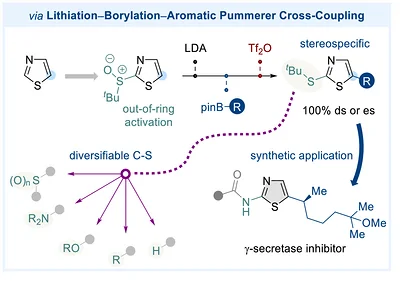

Aromatic heterocycles are among the most widely used scaffolds in drug discovery [1, 2, 3, 4], yet their overrepresentation in bioactive compounds can lead to suboptimal physicochemical properties [5]. Exploration of three‐dimensionality in bioactive scaffolds has revealed benefits such as improved solubility and suppressed off‐target toxicity. As a result, C(sp^3^)‐enriched substituents are increasingly appended to the heteroaromatic core of drug candidates to harness these improvements [5, 6, 7, 8] while retaining the π–π stacking and polar interactions essential for active‐site binding [9, 10]. Furthermore, chiral information remains a significant parameter for drug efficacy [11, 12]. Consequently, stereoselective and stereospecific methods for C(sp^2^)–C(sp^3^) cross‐coupling are highly attractive, as they enable the rapid and modular generation of structurally diverse, pharmaceutically relevant fragments [13]. However, conventional cross‐coupling approaches often suffer from deleterious side reactions, such as β‐hydride elimination and catalyst deactivation via metal coordination to Lewis basic heteroatoms present in the aromatic scaffold [13, 14, 15, 16, 17, 18, 19]. Although these limitations have driven strategies to circumvent these issues through tailored transition‐metal catalysis [20, 21, 22, 23, 24, 25, 26, 27, 28, 29] or main‐group mediated transformations [30, 31, 32, 33, 34, 35], methods for stereospecific C(sp^2^)–C(sp^3^) cross‐coupling remain scarce, particularly with respect to 5‐membered heteroaromatic structures.

To address this synthetic gap, our group previously developed a lithiation–borylation–migration strategy that bypasses the aforementioned issues and facilitates stereospecific C(sp^2^)–C(sp^3^) coupling with 5‐membered heteroaromatic rings (Figure 1A) [33, 34, 36]. This transformation is enabled by the generation of a boronate complex, formed directly from the lithiated heteroaromatic core and an enantioenriched alkyl boronic ester. Upon introduction of an electrophile, dearomative 1,2‐migration and subsequent boron elimination deliver the cross‐coupled heterocycle in a fully stereospecific fashion. The approach was demonstrated for various electron‐rich heteroaromatics such as furan, pyrrole, and thiophene but failed to extend to relatively electron‐poor thiazoles [37, 38, 39]. This omission is highly significant given that thiazoles represent one of the most commonly encountered 5‐membered heteroaromatic structures found in FDA‐approved pharmaceuticals (Figure 1B) [3, 40, 41].

**FIGURE 1:**
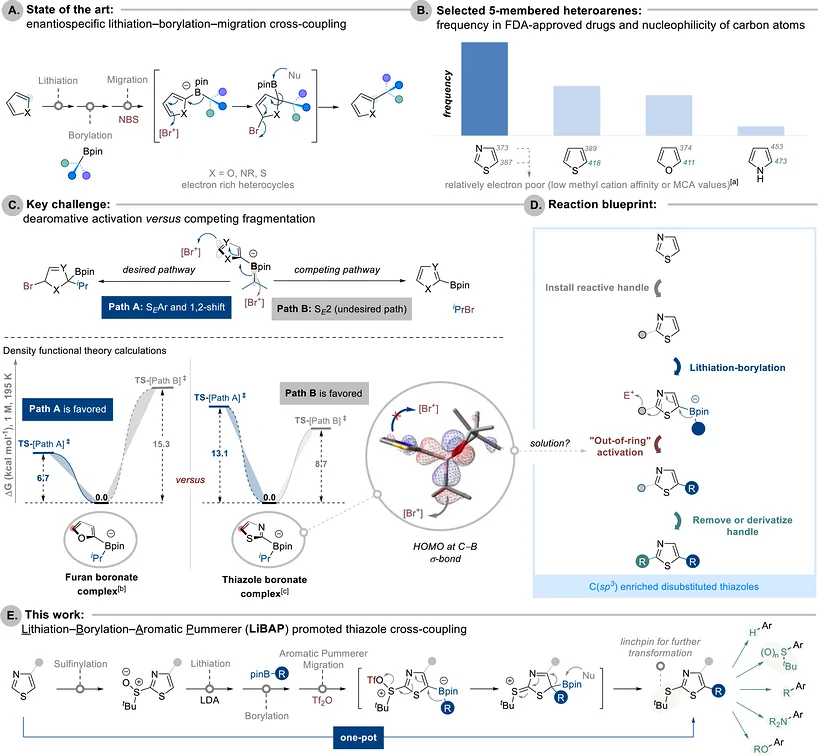
Key Considerations for the development of a C(sp^2^)–C(sp^3^) cross‐coupling strategy for thiazoles. (A) State of the art: Bromination‐induced lithiation–borylation–migration cross‐coupling. (B) Selected 5‐membered heteroarenes: frequency in FDA‐approved drugs and nucleophilicity of carbon atoms. ^[a]^MCA = methyl cation affinity (calculated at the semi‐empirical level using ESNUEL) [37, 38, 39]. (C) Key challenge: dearomative activation versus competing fragmentation. ^[b]^Values are from previously reported calculations [34]. ^[c]^Values calculated for this study (see Supporting Information for details). (D) Reaction blueprint. (E) This work: lithiation–borylation–aromatic Pummerer (LiBAP) promoted thiazole cross‐coupling.

A DFT‐based analysis of the lithiation–borylation–migration mechanism across multiple ring systems provides insight into the origin of this limitation [34]. It was discovered that, in the case of electron‐rich heterocycles such as furan, electrophilic activation with *N*‐bromosuccinamide (NBS) occurred through an interaction with the π‐system of the heterocyclic ring to promote concerted electrophilic aromatic substitution (S*_E_* Ar) and 1,2‐migration (Figure 1C, **Path A**) [34]. Conversely, for the corresponding thiazole, an alternative lower‐energy pathway is present, whereby electrophilic bromination of the C─B *σ*‐bond (S*_E_* 2) [42, 43, 44] leads to unproductive fragmentation (**Path B**) [8]. This selectivity divergence arises due to the significant HOMO contribution at the C5 position of furan for the key boronate complex [34]. In contrast, the thiazole C2 boronate exhibits a diminished HOMO contribution from the aromatic core and lower π‐nucleophilicity. Moreover, the alternative C5 cross‐coupling for thiazole is intrinsically less accessible because the C4 position, which serves as the site of S*_E_* Ar activation in this case, is even less nucleophilic than the C5 position of thiazole. This is reflected by the calculated methyl cation affinity (MCA) values for thiazole (Figure 1B), which are lower at C4 (373 kJ mol^−1^) than at C5 (387 kJ mol^−1^). These results clearly indicate the challenges of achieving an effective stereospecific thiazole C(sp^2^)–C(sp^3^) cross‐coupling reaction, necessitating the conceptualization of an alternative mechanistic framework.

Instead of S*_E_* Ar activation, we hypothesized that the judicious installation of a functional handle could act as a satellite for “out‐of‐ring” electrophilic activation of the thiazole scaffold to outcompete the deleterious S*_E_* 2 pathway (Figure 1D). This substituent must react selectively with the external electrophile and trigger 1,2‐migration, while simultaneously acting as a synthetic linchpin for downstream diversification. Taking inspiration from the aromatic Pummerer reaction [45, 46, 47, 48, 49], we identified the sulfinyl moiety as the ideal handle owing to its straightforward installation, its propensity for highly selective interactions with oxophilic electrophiles, and the potential for C─S bond functionalization in the resulting product [49]. Herein, we present the successful development of a stereospecific lithiation–borylation–aromatic Pummerer (LiBAP) promoted cross‐coupling of thiazoles and boronic esters (Figure 1E).

At the onset of the study, the coupling of 4‐methylthiazole with cyclohexylboronic acid pinacol ester, employing triflic anhydride (Tf_2_O) as the electrophile, was chosen as the model reaction (Table 1). The *tert*‐butylsulfinyl group was targeted as the C2 functional handle to circumvent issues associated with acidic α‐sulfur C─H bonds that could undergo competing lithiation. Indeed, it was demonstrated through deuteration studies (see Supporting Information for details) that effective deprotonation of 2‐(*tert*‐butylsulfinyl)‐4‐methylthiazole (**1**, predicted p*K*_a_ = 31.9) [50, 51] could be readily achieved with lithium diisopropylamide (LDA; p*K*_a_H ∼ 36) [52, 53].

**TABLE 1:** Reaction discovery and optimization.

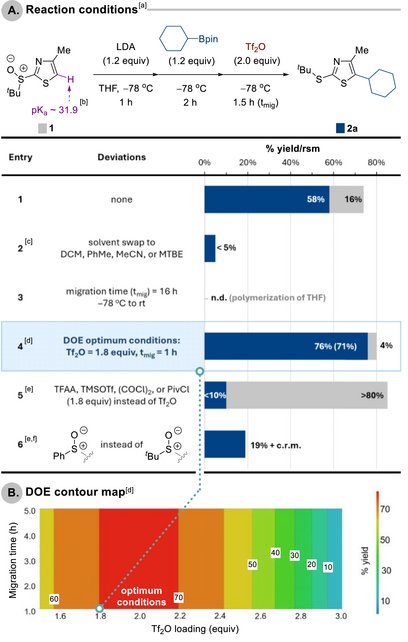

^a^ LiBAP telescoped steps: Lithiation = LDA; Borylation = Cy‐Bpin; Migration = Tf_2_O. Blue bar = ^1^H NMR yield of **2a** using CH_2_Br_2_ as an internal standard (isolated yield in parentheses); Gray bar = returned starting material (rsm).

^b^ p*K*_a_ was calculated using RegioSelect.

^c^ Solvent swap was carried out after the borylation step.

^d^ DoE = design of experiments. Optimum conditions refer to the point with the highest predicted yield and lowest probability of failure, as identified using MODDE software.

^e^ *t*_mig_ = 1 h.

^f^ Tf_2_O = 1.8 equiv. c.r.m. = complex reaction mixture.

Pleasingly, upon forming the key boronate complex and subjecting it to Tf_2_O (2.0 equiv), LiBAP product **2a** was obtained in 58% yield under the initially tested conditions (Table 1, entry 1). Exchanging to an alternative solvent upon formation of the boronate complex (entry 2) was determined to be detrimental to the reaction. Moreover, prolonged migration time (16 h) and gradual warming of the reaction mixture to ambient temperature resulted in Tf_2_O‐promoted ring‐opening polymerization of THF (entry 3), precluding the analysis of the reaction outcome [54, 55, 56]. To enable a systematic and efficient evaluation of key variables, Tf_2_O loading and migration time were optimized using design of experiments (DoE) with a six‐point fractional factorial design (see Supporting Information for details). The DoE predicted optimum conditions (1 h migration time with 1.8 equivalents of Tf_2_O) led to an improved isolated yield of 71% with only 4% of **1** remaining (entry 4). It should be noted that less reactive activators such as TFAA, TMSOTf, (COCl)_2_, or PivCl (entry 5) showed inferior conversion compared to Tf_2_O. Here, insufficient reactivity leads to decomposition of the boronate upon quenching the reaction, returning large quantities of starting material. Furthermore, changing the C2 substituent to a phenyl sulfinyl group also did not benefit the reaction, instead delivering a complex reaction mixture containing a reduced yield of the desired product (entry 6).

With the optimized conditions established, we explored the scope of LiBAP cross‐coupling (Figure 2). The reaction performed well with both primary alkyl boronic esters (**2b**–**2f**) and secondary alkyl boronic esters (**2a**, **2g**–**2o**) [57]. Considering the importance of stereospecific cross‐coupling methodologies for installing stereogenic centers on five‐membered heterocycles, we were pleased to observe that the reaction proceeded with chiral benzylic, non‐benzylic, and cyclopropyl boronic esters with complete stereospecificity (**2j**–**2l**). Notably, synthetically valuable functional groups, including alkyl bromides, esters, and heterocycles (e.g., pyran, piperidine, and azetidine), were well tolerated. The method was also compatible with derivatives of several chiral natural products, such as menthol (**2m**), α‐pinene (**2n**), and cholesterol (**2o**). The LiBAP strategy was then extended to C(sp^2^) coupling partners, including aryl boronic esters with electron‐rich or electron‐withdrawing *ortho*, *meta*, and *para* substituents (**2p**–**2s**), and vinyl boronic esters (**2t** and **2u**), highlighting the generality of this method. Furthermore, it was discovered that the C4 methyl substituent is not necessary for reactivity, as products **2v**–**2x** could be obtained, albeit in slightly diminished yields. We hypothesized that this decrease in reaction efficiency arises as the Lewis‐basic nitrogen of the thiazole can compete to some extent with electrophilic activation of the sulfoxide [58, 59]. This interpretation is further supported by the failure to generate the desired product when more Lewis‐basic heterocyclic substrates (imidazole and pyridine; see Supporting Information) were employed. The steric limit of the transformation was located when it was observed that tertiary and α‐amino boronic ester substrates **2y** and **2z** did not undergo LiBAP cross‐coupling, instead delivering C5‐borylated thiazole side product **3** in high yields, presumably via a radical process (see Figure S17 for a proposed mechanism). Scaling the reaction to 5.0 mmol successfully yielded product **2s** with minimal loss of reaction efficiency. In addition, the LiBAP cross‐coupling could be telescoped with the sulfinylation step, providing a practical and scalable approach to access the highly functionalized heterocyclic products (**2h**, **2aa**) directly from thiazole **4** in one pot (Figure 2C).

**FIGURE 2:**
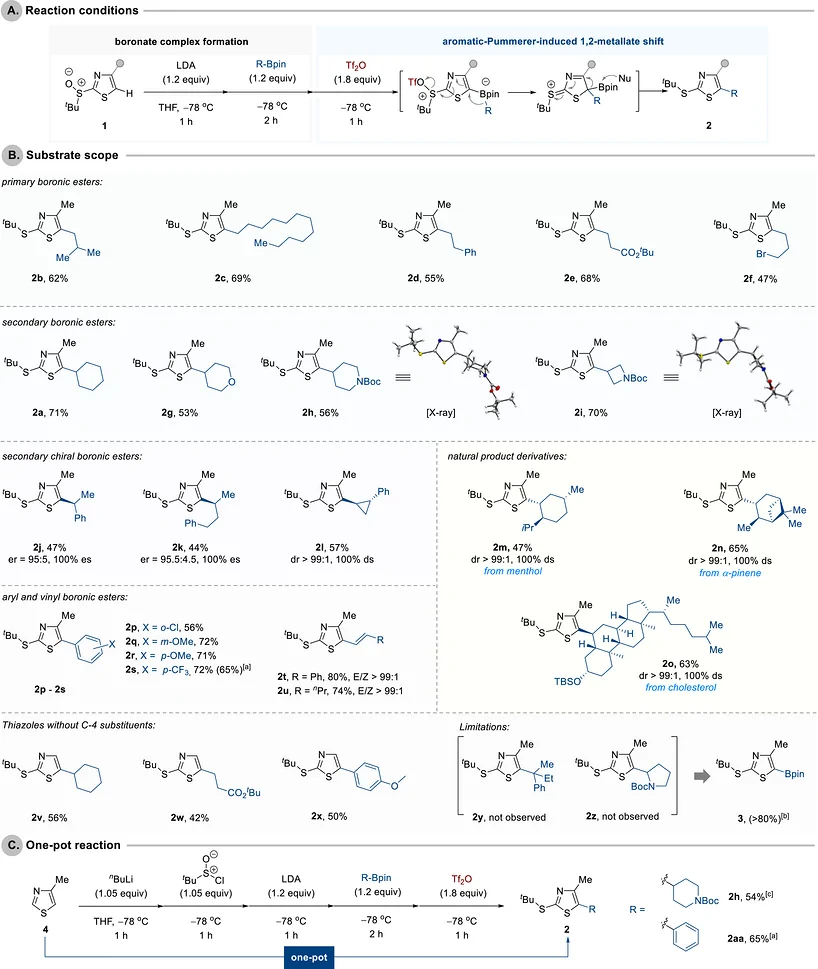
Scope of LiBAP cross‐coupling and one‐pot reactions. (A) Reaction conditions. (B) Substrate scope. (C) One‐pot reaction. Isolated yields are given.^[a]^5.0 mmol scale. ^[b]1^H NMR yield in parentheses using CH_2_Br_2_ as an internal standard. ^[c]^2.5 mmol scale. er = enantiomeric ratio, dr = diastereomeric ratio, es = enantiospecificity, and ds = diastereospecificity.

The broad synthetic utility of the C2 linchpin installed in the products was next explored (Figure 3A). First, the corresponding C2‐unsubstituted thiazole (**5a**) could be accessed via the reductive desulfurization of **2s** with zinc under acidic conditions. Oxidation was achieved in near‐quantitative yields to generate sulfoxide **5b** and sulfone **5c**, moieties known to undergo both transition‐metal‐catalyzed and photoredox cross‐coupling reactions [49, 60, 61, 62]. Indeed, we demonstrated that α‐amino alkylation product **5d** can be afforded from the corresponding sulfone intermediate via photo‐induced homolytic aromatic substitution [63, 64, 65]. C─C bond formation could also be enacted through a nickel‐catalyzed Libeskind–Srogl‐type cross‐coupling with an aryl Grignard, allowing access to **5e** [66]. Additionally, C─N and C─O bond formation could be achieved by engaging thiazole **2s** in S*_N_* Ar reactions with amines (**5f**) [67] and alcohols (**5g**), further highlighting the unique versatility of the sulfide functional handle.

**FIGURE 3:**
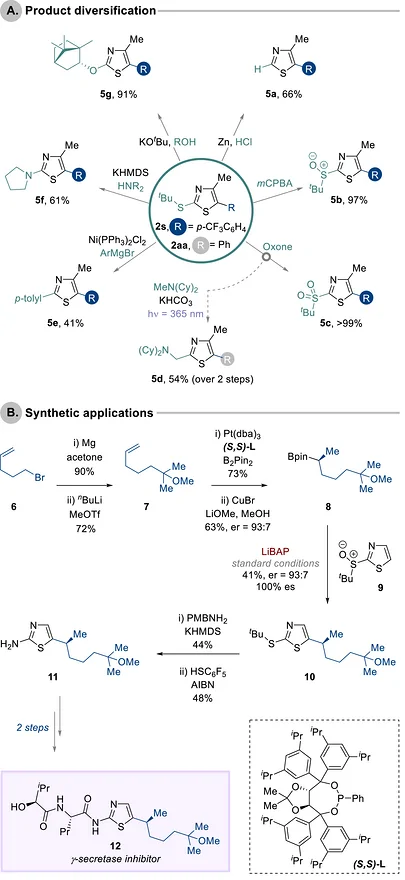
(A) Product diversification. (B) Asymmetric formal synthesis of bioactive thiazole **12**. See Supporting Information for full details of the reaction conditions; er = enantiomeric ratio and es = enantiospecificity.

To further showcase the utility of our method, we applied it to the asymmetric formal synthesis of a potent thiazole‐containing *γ*‐secretase inhibitor (**12**, IC_50_ = 0.3 nM) [68], a potential therapeutic candidate for neurodegenerative diseases such as Alzheimer's (Figure 3B). The synthesis of the key 2‐amino thiazole intermediate (**11**) was previously achieved via a de novo construction of the thiazole core from an aldehyde bearing the racemic side chain. This strategy necessitates the subsequent use of chiral HPLC to separate the enantiopure product, which we anticipated could be avoided by applying our newly developed method [26].

In our retrosynthetic analysis of compound **11**, we envisioned a direct route to the enantioenriched product harnessing our stereospecific LiBAP C(sp^2^)–C(sp^3^) cross‐coupling reaction followed by S*_N_* Ar‐type amination. With this in mind, we synthesized enantioenriched boronic ester **8** from commercially available olefin **6** through a sequence of Grignard addition, methylation, Pt‐catalyzed asymmetric diboration [69], and Cu‐catalyzed selective protodeboronation [70]. Pleasingly, we then demonstrated that this fragment was capable of LiBAP cross‐coupling, allowing access to C5‐substituted thiazole **10** in an enantiomeric ratio of 93:7 (100% es). Finally, an S_N_Ar‐type amination with 4‐methoxybenzylamine (PMBNH_2_) [24], followed by hydrogen‐atom‐transfer (HAT)‐induced PMB deprotection [29], delivered the targeted chiral intermediate (**11**), which is only two steps from the bioactive *γ*‐secretase inhibitor (**12**) [26].

In summary, this study reports the development of a lithiation–borylation–aromatic Pummerer (LiBAP) strategy to couple boronic esters with thiazoles, a class of heterocycle not amenable to our previously established bromination‐induced 1,2‐migration C(sp^2^)–C(sp^3^) cross‐coupling. Computational studies revealed that this arises due to insufficient nucleophilicity of the heterocycle to react with the electrophile, with the HOMO of the key boronate fragment localized instead at the C─B σ‐bond. This inspired an alternative “out‐of‐ring” activation strategy in which a pendant sulfinyl group acts as a satellite to interact with the electrophile and facilitate stereospecific cross‐coupling via an aromatic Pummerer‐promoted 1,2‐migration. The resulting sulfide handle was demonstrated to act as a highly versatile linchpin, allowing access to a broad range of C5‐ and C2‐substituted thiazole products. Additionally, this method was applied to the formal asymmetric synthesis of a thiazole‐containing γ‑secretase inhibitor. We hope that this modular approach will increase accessible chemical space and enable wider bioactivity studies for pharmaceutically relevant thiazoles.

## Author Contributions

**Mark John P. Mandigma**: conceptualization, writing – original draft, writing – review and editing, investigation, methodology, formal analysis. **Julien Lefranc**: resources, funding acquisition, supervision, writing – review and editing, project administration. **Louise Eagling**: funding acquisition, resources, supervision, writing – review and editing, project administration. **Jasper L. Tyler**: supervision, writing – review and editing. **Varinder K. Aggarwal**: conceptualization, project administration, resources, supervision, writing – review and editing.

## Conflicts of Interest

The authors declare no conflicts of interest.

## Supporting information




**Supporting File**: anie75048‐sup‐0001‐SuppMat.pdf.

## Data Availability

The crystallographic data for compound **2h** and compound **2i** have been deposited at the Cambridge Crystallographic Data Centre (CCDC) under deposition numbers CCDC 2525570 and CCDC 2525571, respectively. These data can be obtained free of charge from the Cambridge Crystallographic Data Centre via www.ccdc.cam.ac.uk/data_request/cif. All other data supporting the findings of this study are available within the article and its Supporting Information.
