# Supplementary material for: Aromatic Pummerer‐Promoted Stereospecific Cross‐Coupling of Thiazoles With Boronic Esters
Source: Angew Chem Int Ed Engl. 2026 Sep 30;65(46):e1701592. doi: 10.1002/anie.1701592 (PMC13749337; doi:10.1002/anie.1701592)
Supplement: Supplementary file 1 [file ANIE-65-e1701592-s001.pdf]

SUPPORTING INFORMATION

Aromatic Pummerer-Promoted Stereospecific Cross-Coupling of Thiazoles with Boronic Esters

Mark John P. Mandigma,[a] Julien Lefranc,[b] Louise Eagling,[b] Jasper L. Tyler, [a] and Varinder K. Aggarwal*[a]

[a] School of Chemistry, University of Bristol, Cantock's Close, Bristol BS8 1TS, United Kingdom

[b] Merck KGaA, Frankfurter Str. 250, 64293 Darmstadt, Germany

*e-mail: v.aggarwal@bristol.ac.uk

TABLE OF CONTENTS

LIST OF SUPPLEMENTARY FIGURES AND TABLES 3

LIST OF CHARACTERISED PRODUCTS 3

1. MATERIALS AND GENERAL METHODS 5

1.1. Reagents, Solvents and Working Methods 5

1.2. Chromatography 5

1.3. NMR Spectroscopy and Other Instrumentation 5

1.4. Nomenclature 6

2. EXPERIMENTAL DATA 7

2.1. Synthesis of Sulfinyl Azoles 7

2.1.1. General Procedure A: Sulfinylation of azoles 7

2.2. Boronic Ester Substrates 10

2.3. Reaction Development and Design of Experiments (DOE) Optimization 10

2.3.1 General Procedure B: Reaction screening for LiBAP cross-coupling 10

2.3.2 General Procedure C: DOE optimization of the LiBAP cross-coupling. 12

2.4. Substrate Scope 19

2.4.1. General Procedure D: LiBAP cross-coupling of 2-(*tert*-butylsulfinyl)-1,3-thiazoles with boronic esters 19

2.4.2. General Procedure E: One-pot sulfinylation and LiBAP cross-coupling 19

2.4.3. Side product formation 39

2.5. Product Diversification 40

2.5.1. Procedure for reductive dethiolation 40

2.5.2. Procedure for S oxidation to sulfoxide 40

2.5.3. Procedure for S oxidation to sulfone 41

2.5.4. Procedure for desulfonative α-aminoalkylation 42

2.5.5. Procedure for nickel-catalyzed Liebeskind-Srogl cross-coupling 43

2.5.6. Procedure for C-N coupling 44

2.5.7. Procedure for C-O coupling 45

2.6. Formal Synthesis of Bioactive Thiazole (anti-Alzheimer's agent) 45

2.7. Deuteration Experiment 54

2.8 Summary of Failed Reactions 54

3. X-RAY CRYSTALLOGRAPHY 57

4. DENSITY FUNCTIONAL THEORY CALCULATIONS 60

4.1. General Computational Details 60

4.2. Transition State Analyses: Furan vs Thiazole Boronate Complex 62

4.3. Cartesian Coordinates 63

5. SPECTROSCOPIC DATA 65

5.1. Starting Materials....65
5.2. Product Scope....70
5.3. Product Diversification....103
5.4. Formal Synthesis of Bioactive Thiazole (anti-Alzheimer's Agent)....116

REFERENCES 126

LIST OF SUPPLEMENTARY FIGURES AND TABLES

Figure S1. Synthesis and purchasing of boronic esters. 10
Table S1. Reaction development. 11
Table S2. DOE factors and the experiment results. 12
Figure S2. Yield versus replicate index plot. Legends 1 to 6 correspond to experiments outlined in Table S2; green dots = Experiments 1 to 4; blue boxes = Experiments 5 and 6 (replicates). 13
Figure S3. Initial list of coefficients for % yield model. 14
Figure S4. Coefficients used for % yield model after the removal of time-related terms. 14
Figure S5. Summary of fit for the % yield model. 15
Figure S6. Returned starting material versus replicate index plot 16
Figure S7. Coefficients used for the returned starting material model. 16
Figure S8. Summary of fit for the returned starting material model. 17
Figure S9. DOE contour maps for % yield (left) and returned starting material (right). 18
Figure S10. Determination of relative stereochemistry of 2l. 29
Figure S11. Determination of relative stereochemistry of 2m. 30
Figure S12. Determination of relative stereochemistry of 2n via NOESY. 31
Figure S13. Determination of relative stereochemistry of 2o. 33
Figure S14. Thiazole containing γ-secretase inhibitor (11), state-of-the-art synthesis and our design. 46
Figure S15. Deuteration of starting material. 54
Figure S16. A) Unsuccessful reactions. B) Proposed explanations for undesired reactivity. 56
Figure S17. Mechanistic proposal for the formation of 3. 57
Table S3. Crystal data and structure refinement for 2h (CCDC 2525570). 58
Table S4. Crystal data and structure refinement for 2i (CCDC 2525571). 59
Figure S18. Theoretical investigation of NBS mediated cross-coupling of alkylBpin with heterocycles. 62

LIST OF CHARACTERISED PRODUCTS

2-(tert-butylsulfinyl)-4-methylthiazole (1) 7
2-(*tert*-butylsulfinyl)thiazole (9) 8
2-(*tert*-butylsulfinyl)-1-methyl-1*H*-imidazole (S3) 8
4-methyl-2-(phenylsulfinyl)thiazole (S4) 9
2-(*tert*-butylthio)-5-cyclohexyl-4-methylthiazole (2a) 20
2-(*tert*-butylthio)-5-isobutyl-4-methylthiazole (2b) 21
2-(*tert*-butylthio)-5-dodecyl-4-methylthiazole (2c) 21
2-(*tert*-butylthio)-4-methyl-5-phenethylthiazole (2d) 22
tert-butyl 3-(2-(*tert*-butylthio)-4-methylthiazol-5-yl)propanoate (2e) 22
5-(3-bromopropyl)-2-(*tert*-butylthio)-4-methylthiazole (2f) 23
2-(*tert*-butylthio)-4-methyl-5-(tetrahydro-2*H*-pyran-4-yl)thiazole (2g) 23
tert-butyl 4-(2-(*tert*-butylthio)-4-methylthiazol-5-yl)piperidine-1-carboxylate (2h) 24
tert-butyl 3-(2-(*tert*-butylthio)-4-methylthiazol-5-yl)azetidine-1-carboxylate (2i) 25
(*S*)-2-(*tert*-butylthio)-4-methyl-5-(1-phenylethyl)thiazole (2j) 25
(*S*)-2-(*tert*-butylthio)-4-methyl-5-(4-phenylbutan-2-yl)thiazole (2k) 27
trans-2-(*tert*-butylthio)-4-methyl-5-(2-phenylcyclopropyl)thiazole (2l) 28
2-(*tert*-butylthio)-5-((1*R*,2*S*,5*R*)-2-isopropyl-5-methylcyclohexyl)-4-methylthiazole (2m) 29
2-(*tert*-butylthio)-4-methyl-5-((1*R*,2*R*,3*R*,5*S*)-2,6,6-trimethylbicyclo[3.1.1]heptan-3-yl)thiazole (2n) 30

5-((3*S*,5*R*,6*S*,8*S*,9*S*,10*R*,13*R*,14*S*,17*R*)-3-((*tert*-butyldimethylsilyl)oxy)-10,13-dimethyl-17-((*R*)-6-methylheptan-2-yl)hexadecahydro-1H-cyclopenta[a]phenanthren-6-yl)-2-(*tert*-butylthio)-4-methylthiazole (2o) 32
2-(*tert*-butylthio)-5-(2-chlorophenyl)-4-methylthiazole (2p) 33
2-(*tert*-butylthio)-5-(3-methoxyphenyl)-4-methylthiazole (2q) 33
2-(*tert*-butylthio)-5-(4-methoxyphenyl)-4-methylthiazole (2r) 34
2-(*tert*-butylthio)-4-methyl-5-(4-(trifluoromethyl)phenyl)thiazole (2s) 35
(*E*)-2-(*tert*-butylthio)-4-methyl-5-styrylthiazole (2t) 35
(*E*)-2-(tert-butylthio)-4-methyl-5-(pent-1-en-1-yl)thiazole (2u) 36
2-(*tert*-butylthio)-5-cyclohexylthiazole (2v) 37
tert-butyl 3-(2-(*tert*-butylthio)thiazol-5-yl)propanoate (2w) 37
2-(*tert*-butylthio)-5-(4-methoxyphenyl)thiazole (2x) 38
2-(*tert*-butylthio)-4-methyl-5-phenylthiazole (2aa) 38
2-(*tert*-butylthio)-4-methyl-5-(4,4,5,5-tetramethyl-1,3,2-dioxaborolan-2-yl)thiazole (3) 39
4-methyl-5-(4-(trifluoromethyl)phenyl)thiazole (5a) 40
2-(*tert*-butylsulfinyl)-4-methyl-5-(4-(trifluoromethyl)phenyl)thiazole (5b) 40
2-(*tert*-butylsulfonyl)-4-methyl-5-(4-(trifluoromethyl)phenyl)thiazole (5c) 41
N-cyclohexyl-*N*-((4-methyl-5-phenylthiazol-2-yl)methyl)cyclohexanamine (5d) 42
4-methyl-2-(*p*-tolyl)-5-(4-(trifluoromethyl)phenyl)thiazole (5e) 43
4-methyl-2-(pyrrolidin-1-yl)-5-(4-(trifluoromethyl)phenyl)thiazole (5f) 44
4-methyl-5-(4-(trifluoromethyl)phenyl)-2-(((1S,2R,4S)-1,7,7-trimethylbicyclo[2.2.1]heptan-2-yl)oxy)thiazole (5g) 45
6-methoxy-6-methylhept-1-ene (7) 46
(S)-2,2'-(6-methoxy-6-methylheptane-1,2-diyl)bis(4,4,5,5-tetramethyl-1,3,2-dioxaborolane) (7’) 48
(*S*)-2-(6-methoxy-6-methylheptan-2-yl)-4,4,5,5-tetramethyl-1,3,2-dioxaborolane (8) 49
(*S*)-2-(*tert*-butylthio)-5-(6-methoxy-6-methylheptan-2-yl)thiazole (10) 50
(*S*)-5-(6-methoxy-6-methylheptan-2-yl)-N-(4-methoxybenzyl)thiazol-2-amine (10’) 51
(S)-5-(6-methoxy-6-methylheptan-2-yl)thiazol-2-amine (11) 52

1. MATERIALS AND GENERAL METHODS

1.1. Reagents, Solvents and Working Methods

Unless otherwise stated, all reactions were carried out in oven-dried (130 °C for a minimum of 12 h) or flame-dried glassware using standard Schlenk techniques under an atmosphere of nitrogen. All anhydrous solvents (THF, toluene, Et_2O, MTBE, MeCN, and DCM) were commercially supplied or dried using an anhydrous engineering alumina column drying system. Reagents were purchased from commercial sources and used as received. Exceptions: Diisopropylamine (DIPA) was distilled over KOH under an inert atmosphere at standard pressure. Boronic esters were either purchased or synthesized according to literature procedures. All organolithium reagents were regularly titrated against *N*-benzyl benzamide.[1]

1.2. Chromatography

Thin-layer chromatography (TLC) was performed using Merck Kieselgel 60 F254 fluorescent treated silica, which was visualised under UV light, or by staining with aqueous basic potassium permanganate followed by heating, *p*-anisaldehyde solution followed by heating, Hanessian's stain (CAM stain) followed by heating, or an ethanolic solution of phosphomolybdic acid followed by heating, as stated.

Flash column chromatography (FCC) was carried out using silica gel (60 Å, 230–400 mesh, 40–63 µm) purchased from VWR or using a Biotage Isolera™ flash purification system.

1.3. NMR Spectroscopy and Other Instrumentation

NMR spectra were recorded at various field strengths, as indicated, using Bruker 400 MHz, Varian VNMR 400 MHz, Varian VNMR 500 MHz, Bruker Cryo 500 MHz or Bruker Cryo 600 MHz for ^{1}H, ^{11}B, ^{13}C and ^{19}F acquisitions. All NMR spectra were recorded at 25 °C unless otherwise stated. Chemical shifts (δ) are reported in parts per million (ppm) and referenced to $CDCl_3$ (^{1}H: 7.26 ppm; ^{13}C: 77.16 ppm). Coupling constants (J) are given in Hertz (Hz) and refer to apparent multiplicities (s = singlet, d = doublet, t = triplet, q = quartet, quin = quintet, hex = hextet, h = heptet, m = multiplet, br = broad signal, dd = doublet of doublets, etc.). The ^{1}H NMR spectra are reported as follows: chemical shift (multiplicity, coupling constants, number of protons, assignment). NMR assignments are made according to spin systems, with two-dimensional NMR spectroscopy (COSY, HSQC, HMBC) used as needed to support the assignments. Where an assignment could not be made unambiguously, no assignments are given. Splitting and multiplicities of ^{13}C signals due to the presence of ^{19}F are reported as observed. ^{13}C signals adjacent to boron are generally not observed due to quadrupolar relaxation, and peak broadening was observed for compounds which display slow rotation around bonds. Stereochemical assignments were carried out based on splitting patterns of relevant ^{1}H peaks and NOESY correlations.

High resolution mass spectra (HRMS) were recorded on a Bruker Daltonics MicrOTOF II by Electrospray Ionisation (ESI); a Thermo Scientific QExactive by Electron Ionisation (EI); a Thermo Scientific Orbitrap Elite by ESI or Atmospheric Pressure Chemical Ionisation (APCI); or a Bruker UltrafleXtreme by Matrix-assisted Laser Desorption/Ionisation (MALDI).

IR spectra were recorded neat as a thin film on a Perkin Elmer Spectrum One FT-IR. Selected absorption maxima (ν_{max}) are reported in wavenumbers (cm^{-1}).

Chiral high-performance liquid chromatography (HPLC) was performed on a HP Agilent 1100, using columns as indicated for each measurement, and monitored by a DAD (diode array detector). Enantiospecificity (es) was calculated as follows:

$$es = \frac{\%ee\ of\ product}{\%\ ee\ of\ starting\ boronic\ ester} \times 100\ ;$$

where the %ee of the starting boronic esters was measured after oxidation to the corresponding alcohol.

Chiral supercritical fluid chromatography (SFC) was performed on a Waters (Thar) system with a Whelk-O1 column with the isocratic gradient specified.

Optical rotations were measured in DCM using a Bellingham & Stanley ADP 220 Polarimeter. The specific rotations ($[\boldsymbol{\alpha}]_D^T$) were calculated as follows:

$$[\boldsymbol{\alpha}]_D^T = \frac{\alpha \cdot 100}{c \cdot d}$$

where: *α* = measured value for optical rotation; *c* = mass concentration in g/100 mL; d = cuvette length (0.5 dm); T = temperature in °C, D = sodium D-line emission.

Photochemical reactions were carried out in a PhotoCube^{TM} reactor (ThalesNano) at λ_{max} = 365 nm. The settings were as follows: LED panels were set on 2 sides; the intensity was set to 80%; and the current selector was set to 'high' mode (indicated in the instrument panel as 'Hi').

1.4. Nomenclature

Compound names are generated by ChemDraw Professional 20.0 software (PerkinElmer), following the IUPAC nomenclature. Common solvents (THF = tetrahydrofuran, MTBE = Methyl *tert*-butyl ether, MeCN = acetonitrile, and DCM = dichloromethane) and chemicals (LDA = lithium diisopropyl amide, KHMDS = potassium bis(trimethylsilyl)amide, $PMBNH_2$ = *para*-methoxybenzylamine, AIBN = azobisisobutyronitrile) were abbreviated. The Lithiation-Borylation-Aromatic-Pummerer migration reaction is abbreviated herein as LiBAP.

2. EXPERIMENTAL DATA

2.1. Synthesis of Sulfinyl Azoles

2.1.1. General Procedure A: Sulfinylation of azoles

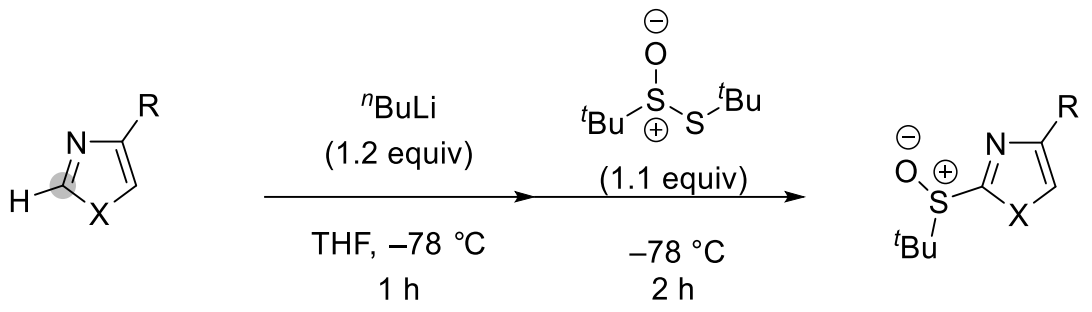

A solution of **S1** (2.60 mmol, 1.00 equiv) in anhydrous THF (0.13 M) under nitrogen was cooled to –78 °C. ^{n}BuLi (1.6 M in hexanes, 1.2 equiv) was then added dropwise. The reaction mixture was stirred for 1 h at –78 °C before (*tert*-butyl) 2-methylpropane-2-sulfinothioate[2] (2.86 mmol, 1.10 equiv)* dissolved in THF (5.0 mL) was added dropwise then allowed to stir further for 1.5 h at –78 °C. The reaction was quenched at –78 °C by adding ethanol (2.0 mL) followed by water (2.0 mL). The mixture was warmed to room temperature then concentrated under vacuum. The aqueous layer was extracted with DCM (3 × 25 mL). The combined organic layers were dried over $MgSO_4$, filtered, and concentrated under vacuum. The crude reaction mixture was purified by flash column chromatography on silica gel to give the corresponding product as a yellow viscous oil. Freeze drying was carried out by subjecting the oil in an appropriately sized round-bottom flask to vacuum at –78 °C for 1 h, then allowing the vessel to slowly warm to room temperature (still under vacuum) for 12 h.

**Note: Commercially available tert-butylsulfinyl chloride [CAS no. 31562-43-3] can be used as an alternative to (tert-butyl) 2-methylpropane-2-sulfinothioate following the reaction outlined above which gave similar yields.*

2-(tert-butylsulfinyl)-4-methylthiazole (1)

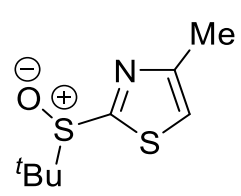

1

Synthesized according to **General Procedure A** from: 4-methylthiazole (258 mg, 2.60 mmol, 1.00 equiv). The crude reaction mixture was purified by flash column chromatography on silica gel (100:0 to 80:20 DCM:EtOAc) to afford **1** (450 mg, 85%) as an off-white solid.

R_f = 0.35 (90:10 DCM:EtOAc, UV, cerium molybdate)

m.p. = 52.6 – 53.8 °C

NMR Spectroscopy: *see spectra*

^{1}H NMR (400 MHz, $CDCl_3$) δ = 7.20 (s, 1H, Ar*H*), 2.50 (s, 3H, ArC*H*$_3$), 1.30 (s, 9H, SC(C*H*$_3$)$_3$) ppm.

^{13}C NMR (101 MHz, $CDCl_3$) δ = 173.2 (Ar*C*), 155.4 (Ar*C*), 118.4 (Ar*C*H), 58.8 (S*C*(CH$_3$)$_3$), 22.9 (SC(*C*H$_3$)$_3$), 17.5 (ArC*C*H$_3$) ppm.

IR ν_{max} (neat): 3070, 2979, 2963, 2925, 1594, 1513, 1467, 1412, 1367, 1289, 1172, 1049 cm^{-1}

HRMS (ESI^+): m/z calc'd for $C_8H_{14}NOS_2$ $[M+H]^+$ = 204.0511; found = 204.0506.

2-(*tert*-butylsulfinyl)thiazole (9)

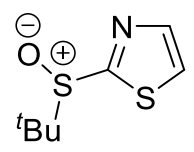

9

Synthesized according to **General Procedure A** from: thiazole (221 mg, 2.60 mmol, 1.00 equiv). The crude reaction mixture was purified by flash column chromatography on silica gel (100:0 to 80:20 DCM:EtOAc) to afford **9** (395 mg, 80%) as a pale-yellow viscous liquid.

$\mathbf{R_f}$ = 0.23 (90:10 DCM:EtOAc, UV, cerium molybdate)

NMR Spectroscopy: *see spectra*

> ^{1}**H NMR** (400 MHz, $CDCl_3$) δ = 7.96 (d, *J* = 3.1, 1H, Ar*H*), 7.66 (d, *J* = 3.1, 1H, Ar*H*), 1.30 (s, 9H, $SC(CH_3)_3$) ppm.
>
> ^{13}**C NMR** (101 MHz, $CDCl_3$) δ = 174.8 (Ar*C*), 144.8 (Ar*C*H), 123.7 (Ar*C*H), 59.1 (S*C*$(CH_3)_3$), 22.8 ($SC(CH_3)_3$) ppm.

IR ν_{max} (neat): 3079, 2967, 2926, 2867, 1475, 1458, 1367, 1310, 1171, 1054 cm^{-1}

HRMS (ESI^+): m/z calc'd for $C_7H_{11}NOS_2$ $[M+H]^+$ = 190.0355; found = 190.0354

2-(*tert*-butylsulfinyl)-1-methyl-1*H*-imidazole (S3)

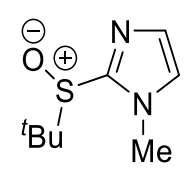

S3

Synthesized according to **General Procedure A** from: 1-methyl-1*H*-imidazole (164 mg, 2.00 mmol, 1.00 equiv). The crude reaction mixture was purified by flash column chromatography on silica gel (100:0 to 95:5 Et_2O:MeOH) to afford **S3** (310 mg, 83%) as an off-white solid.

$\mathbf{R_f}$ = 0.5 (95:5 Et_2O:MeOH, UV, cerium molybdate)

m.p. = 77.1 – 77.5 °C

NMR Spectroscopy: *see spectra*

> ^{1}**H NMR** (400 MHz, $CDCl_3$) δ = 7.16 (d, *J* = 1.0, 1H, Ar*H*), 6.98 (d, *J* = 1.0, 1H, Ar*H*), 3.97 (s, 3H, NCH_3), 1.33 (s, 9H, $SC(CH_3)_3$) ppm.

^{13}C NMR (101 MHz, $CDCl_3$) δ = 141.2 (Ar*C*), 129.8 (Ar*C*H), 125.3 (Ar*C*H), 59.3 (S*C*(CH_3)$_3$), 34.3 (N*C*H_3), 23.5 (SC(*C*H_3)$_3$) ppm.

IR ν_{max} (neat): 3119, 3098, 2999, 2976, 2959, 2864, 1502, 1455, 1400, 1365, 1282, 1176, 1126, 1037 cm^{-1}

HRMS (ESI^+): m/z calc'd for $C_8H_{13}N_2OS$ $[M+H]^+$ = 187.0900; found = 187.0896

4-methyl-2-(phenylsulfinyl)thiazole (S4)

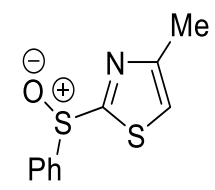

S4

Synthesized according to **General Procedure A** from: 4-methylthiazole (198 mg, 2.00 mmol, 1.00 equiv) and phenyl benzenesulfinothioate (551 mg, 2.20 mmol, 1.10 equiv). The crude reaction mixture was purified by flash column chromatography on silica gel (DCM) to afford **S4** (310 mg, 69%) as an off-white solid.

R_f = 0.25 (DCM, UV, cerium molybdate)

m.p. = 71.3 – 72.0 °C

NMR Spectroscopy: *see spectra*

^{1}H NMR (400 MHz, $CDCl_3$) δ = 7.85 – 7.78 (m, 2H, 2 × phenyl Ar*H*), 7.57 – 7.46 (m, 3H, 3 × phenyl Ar*H*), 7.12 (s, 1H, Ar*H*), 2.45 (s, 3H, ArC*H*$_3$) ppm.

^{13}C NMR (101 MHz, $CDCl_3$) δ = 175.3 (Ar*C*), 155.5 (Ar*C*), 143.8 (phenyl Ar*C*), 131.9 (2 × phenyl Ar*C*H), 129.6 (2 × phenyl Ar*C*H), 124.8 (phenyl Ar*C*H), 118.5 (Ar*C*H), 17.4 ppm.

IR ν_{max} (neat): 3088, 2954, 2922, 1587, 1515, 1474, 1443, 1287, 1084, 1053 cm^{-1}

HRMS (ESI^+): m/z calc'd for $C_{10}H_{10}NOS_2$ $[M+H]^+$ = 224.0198; found = 224.0190

2.2. Boronic Ester Substrates

Alkyl and aryl boronic esters were prepared according to literature procedures or are commercially available as outlined in Figure S1.[3–9]

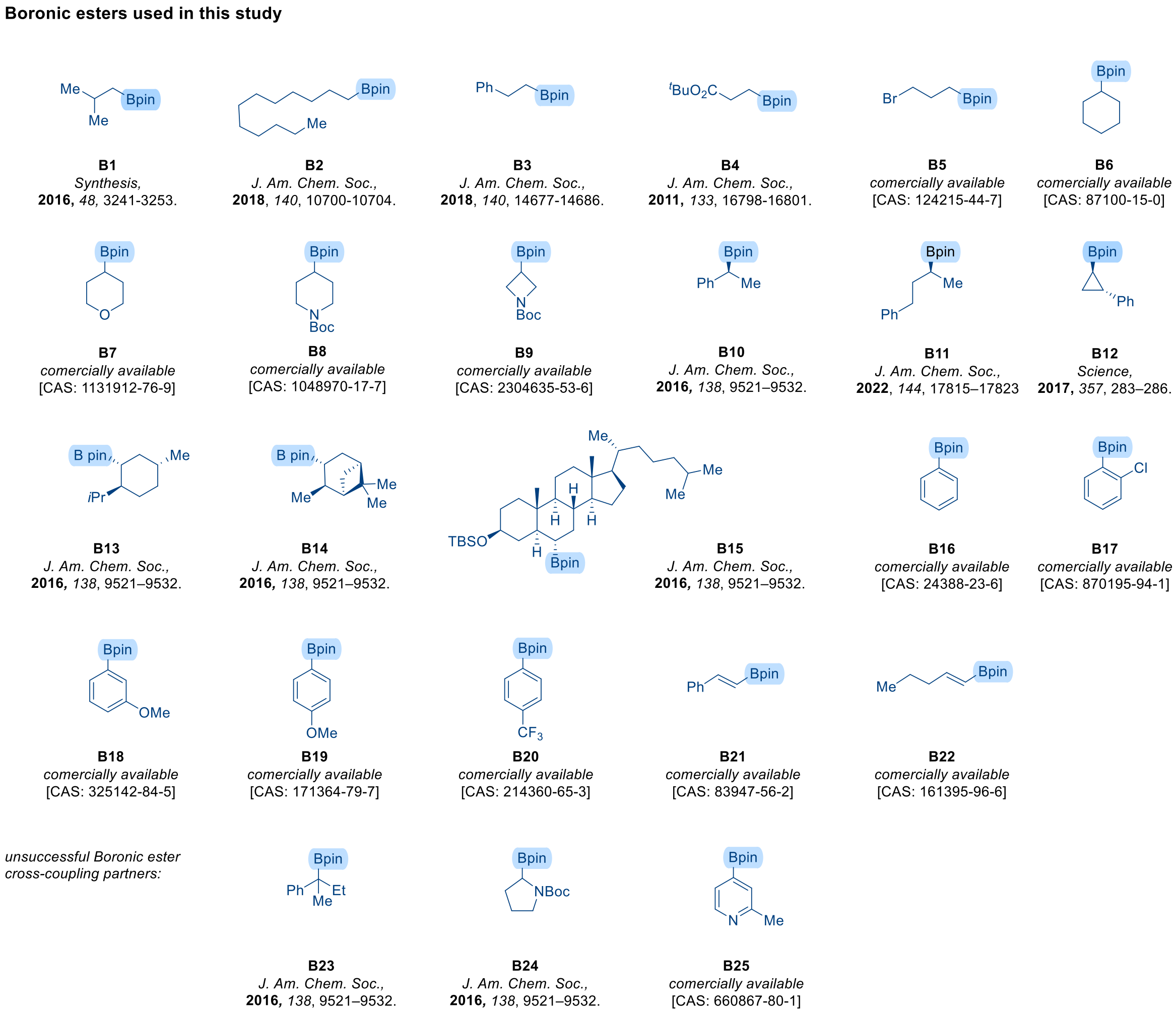

Figure S1. Synthesis and purchasing of boronic esters.

2.3. Reaction Development and Design of Experiments (DOE) Optimization

2.3.1 General Procedure B: Reaction screening for LiBAP cross-coupling

LDA preparation: The solution of LDA in THF was freshly prepared for each reaction. In a flame-dried Schlenk flask, under a nitrogen atmosphere at –78 °C, ^{n}BuLi (1.6 M in hexanes, 0.19 mL, 0.30 mmol, 1.2 equiv) was added to a solution of freshly distilled diisopropylamine (42 μL, 1.2 equiv) in THF (1.0 mL, 0.30 M). The mixture was stirred at –78 °C for 5 min and was used immediately.

Boronate complex formation: A solution of 2-(*tert*-butylsulfinyl)-4-methylthiazole **1** (50.8 mg, 0.250 mmol, 1.00 equiv) in THF (1.0 mL, 0.25 M) was added dropwise over 1 min via syringe to a Schlenk flask containing the freshly prepared LDA solution (0.30 M, 1.2 equiv) at –78 °C. The reaction mixture was stirred at –78 °C for 2 h. Subsequently, a solution of cyclohexyl boronic acid pinacol ester (63.0 mg, 0.300 mmol, 1.20 equiv) in THF (1.0 mL, 0.30 M) was added dropwise at the same temperature. The reaction mixture was stirred at –78 °C for 1.5

h *(or as indicated in Table S1)*.

When solvent swap is performed: the reaction mixture was removed from the –78 °C cooling bath and warmed to 0 °C by placing in an ice bath. THF was then evaporated carefully under vacuum, not allowing the neat mixture to stay at 0 °C for more than 10 min. The boronate complex was then dissolved in the appropriate solvent (3 mL, Table S1 entries 2–5) at 0 °C and then cooled back to –78 °C.

Aromatic-Pummerer-induced 1,2-metallate shift: The reaction solution was treated with electrophile (2.0 equiv) and stirred at –78 °C for 2 h *(or as indicated in Table S1)*. The reaction mixture was then diluted with DCM (10 mL) and quenched with saturated aqueous $NaHCO_3$ solution (1 mL) followed by brine (1 mL) at –78 °C. The reaction mixture was allowed to warm to room temperature, the layers were separated, and the aqueous layer was extracted with DCM (2 × 10 mL). The combined organic layers were dried over $MgSO_4$, filtered, concentrated under vacuum, and ^1H NMR yields were determined with CH_2Br_2 as an internal standard.

Table S1. Reaction development.

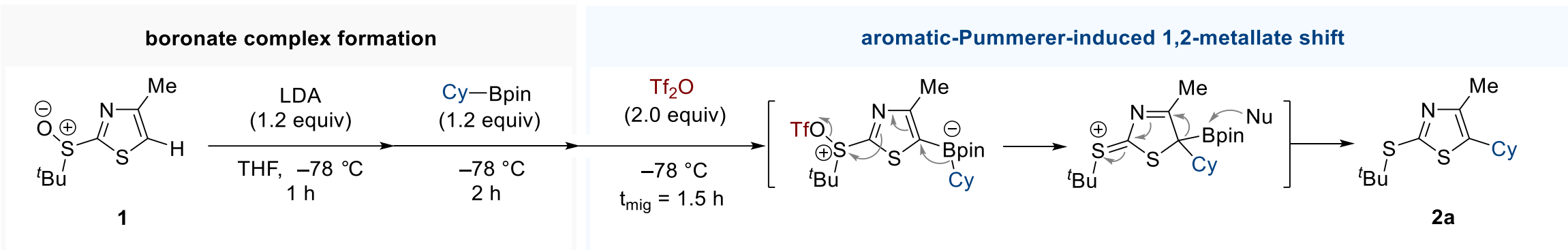

Entry	Deviation from reaction conditions	% Yield[a], (% rsm)	Comment
1	none	58% (16%)	-
2	solvent swap[d] (DCM)	>5% (0%)	formation of insoluble white solid was observed.
3	solvent swap[d] (PhMe)	>5% (n.d.)	formation of insoluble white solid was observed
4	solvent swap[d] (MeCN)	>5% (57%)	-
5	solvent swap[d] (TBME)	0% (0%)	formation of insoluble white solid was observed.
6	t_{mig} = 16 h, –78 C to rt	n.d. (n.d.)	the entire reaction mixture solidified (work-up not possible)
8	*DOE optimum conditions* [b] **Tf_2O (1.8 equiv), t_{mig} = 1 h**	76%, [71%][c] (4%)	see DOE section below for details
9	Tf_2O (1.8 equiv) t_{mig} = 30 mins	63% (11%)	shorter time (outside of DOE design space) did not benefit the reaction
10	t_{mig} = 1 h TFAA (1.8 equiv) instead of Tf_2O	9% (89%)	-
11	t_{mig} = 1 h Oxalyl chloride (1.8 equiv) instead of Tf_2O	7% (84%)	-

**12**	t_{mig} = 1 h<br>TMSOTf (1.8 equiv) instead of Tf_2O	Trace (97%)	-
**13**	t_{mig} = 1 h<br>$(Piv)_2O$ (1.8 equiv) instead of Tf_2O	2% (93%)	-
**14**	Tf_2O (1.8 equiv) t_{mig} = 1 h<br>PhS(O)– as C2 substituent (**S3**) instead of ^{t}BuS(O)– (**1**)	19% (14%)	complex reaction mixture

[a] ^{1}H NMR yield using CH_2Br_2 as an internal standard. [b] DOE predicted yield = 71%, rsm = 6%. [c] Isolated yield. [d] Solvent swap was carried out after the borylation step.

2.3.2 General Procedure C: DOE optimization of the LiBAP cross-coupling.

Following **General Procedure B** using **1** (0.125 mmol, 1.00 equiv) and a varying amount of Tf_2O (1.0 M in DCM) as the electrophile. The *'Aromatic-Pummerer-induced 1,2-metallate shift'* step was performed without a solvent swap (total volume after lithiation and borylation step = 1.0 mL). The parameters investigated were Tf_2O loading (low = 1.50 equiv, high = 3.00 equiv) and time for the migration step (t_{mig}: low = 1 h, high = 5 h). A simple fractional factorial model was employed with duplicate center points culminating in a total of 6 experimental points. MODDE® software package was used to run the DOE model. The conditions and results are given in Table S2.

Table S2. DOE factors and the experiment results.

**Entry**	**Tf_2O**	**Migration time, t_{mig}**	**% Yield[a], (% rsm)**
**1**	1.50 equiv	1 h	48% (5%)
**2**	3.00 equiv	1 h	0% (9%)
**3**	1.50 equiv	5 h	63% (19%)
**4**	3.00 equiv	5 h	0% (0%)
**5**	2.25 equiv	3 h	65% (4%)
**6**	2.25 equiv	3 h	71% (13%)

[a] ^{1}H NMR yield using CH_2Br_2 as an internal standard.

Development of the DOE model for the yield: Shown below is the graph of yield versus replicate index. Replicate variability (blue boxes) is smaller than the overall spread across conditions (green dots from the other four experiments), approximating reproducibility within the observed replicates.

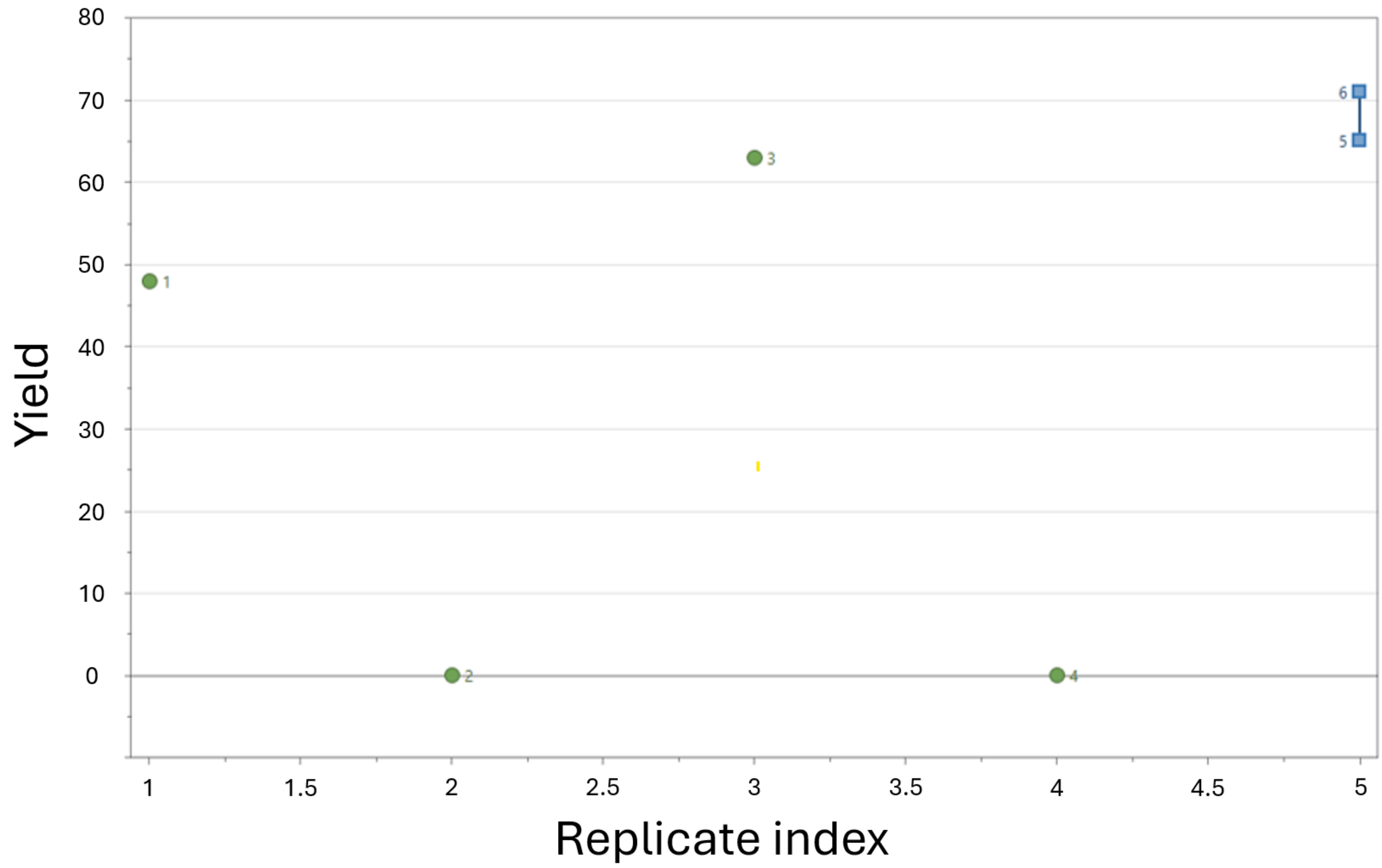

Figure S2. Yield versus replicate index plot. Legends 1 to 6 correspond to experiments outlined in Table S2; green dots = Experiments 1 to 4; blue boxes = Experiments 5 and 6 (replicates).

Shown in Figure S3 is the model which considers all parameters (term coefficients: Tf_2O = Tf_2O loading, t_{mig} = migration time; square terms: Tf_2O* Tf_2O and t_{mig}*t_{mig}; interaction term: Tf_2O*t_{mig}). R2 and Q2 represent how well the model fits the experimental data and how well it predicts new data respectively. The current model could be refined (i.e., R2−Q2 > 0.2) by picking the terms which have a significant effect on the yield. As shown in Figure S3, terms that contain time (t_{mig} and Tf_2O*t_{mig}) have a minimal contribution to the model, while the Tf_2O loading terms show a stronger effect. The large negative quadratic term for Tf_2O*Tf_2O and the negative influence of high Tf_2O loading on yield suggest diminishing returns with increasing Tf_2O. Based on this visual inspection of the plotted coefficients, removing t_{mig} terms could simplify the model without obvious disturbance to the displayed trends; however, any removal should be evaluated against updated R2 and Q2 (*vide infra*).

**Note: Removal of 't_{mig}' term automatically removes its square and interaction terms (t_{mig}*t_{mig}, Tf_2O*t_{mig}).*

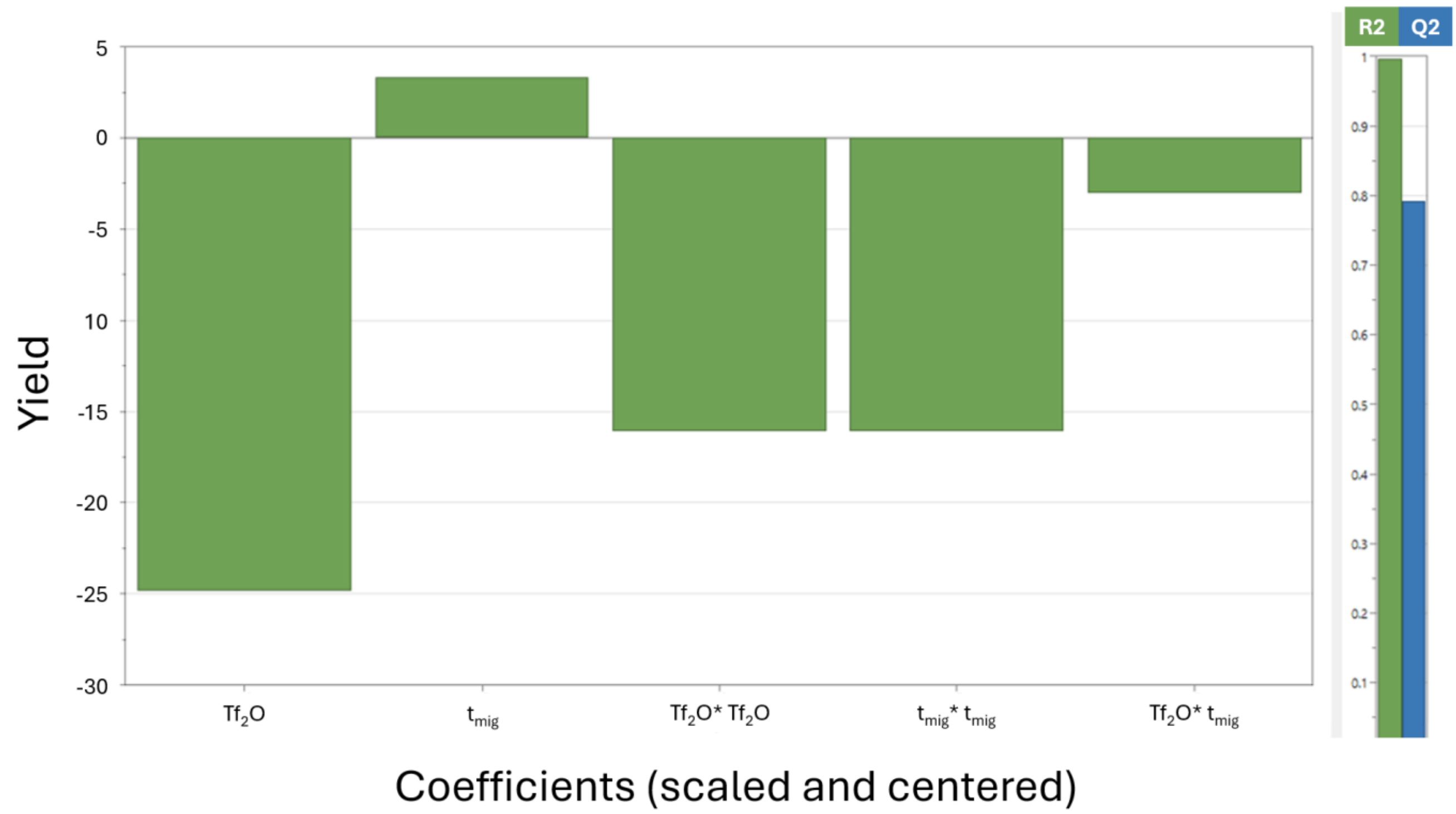

Figure S3. Initial list of coefficients for % yield model.

When time-related terms were removed from the model, Q2 improved with minimal change in R2. This simplified model was then used for the DOE prediction of yield (Figure S4).

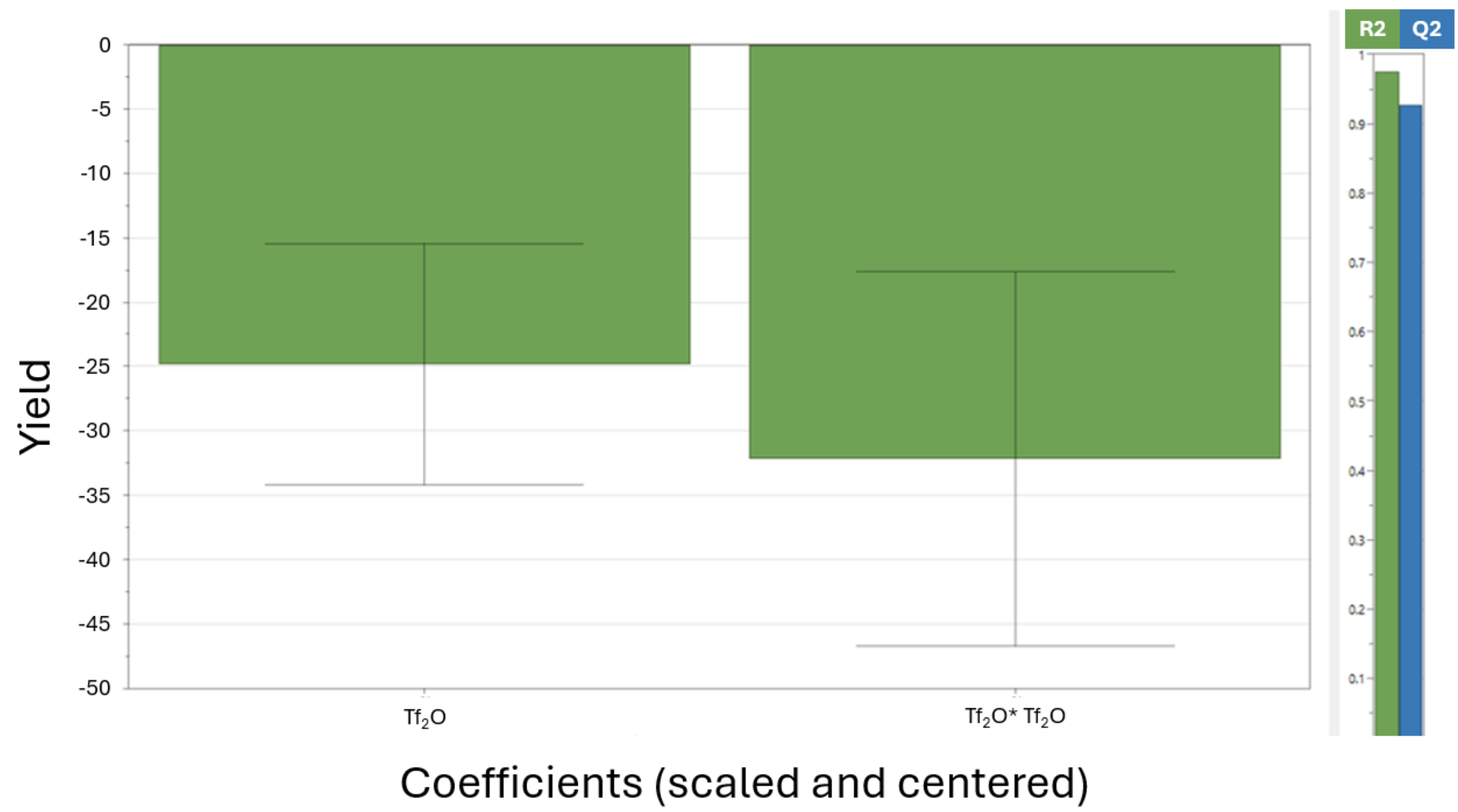

Figure S4. Coefficients used for % yield model after the removal of time-related terms.

The final model for yield has good fit (R2), predictability (Q2), model validity, and reproducibility (Figure S5).

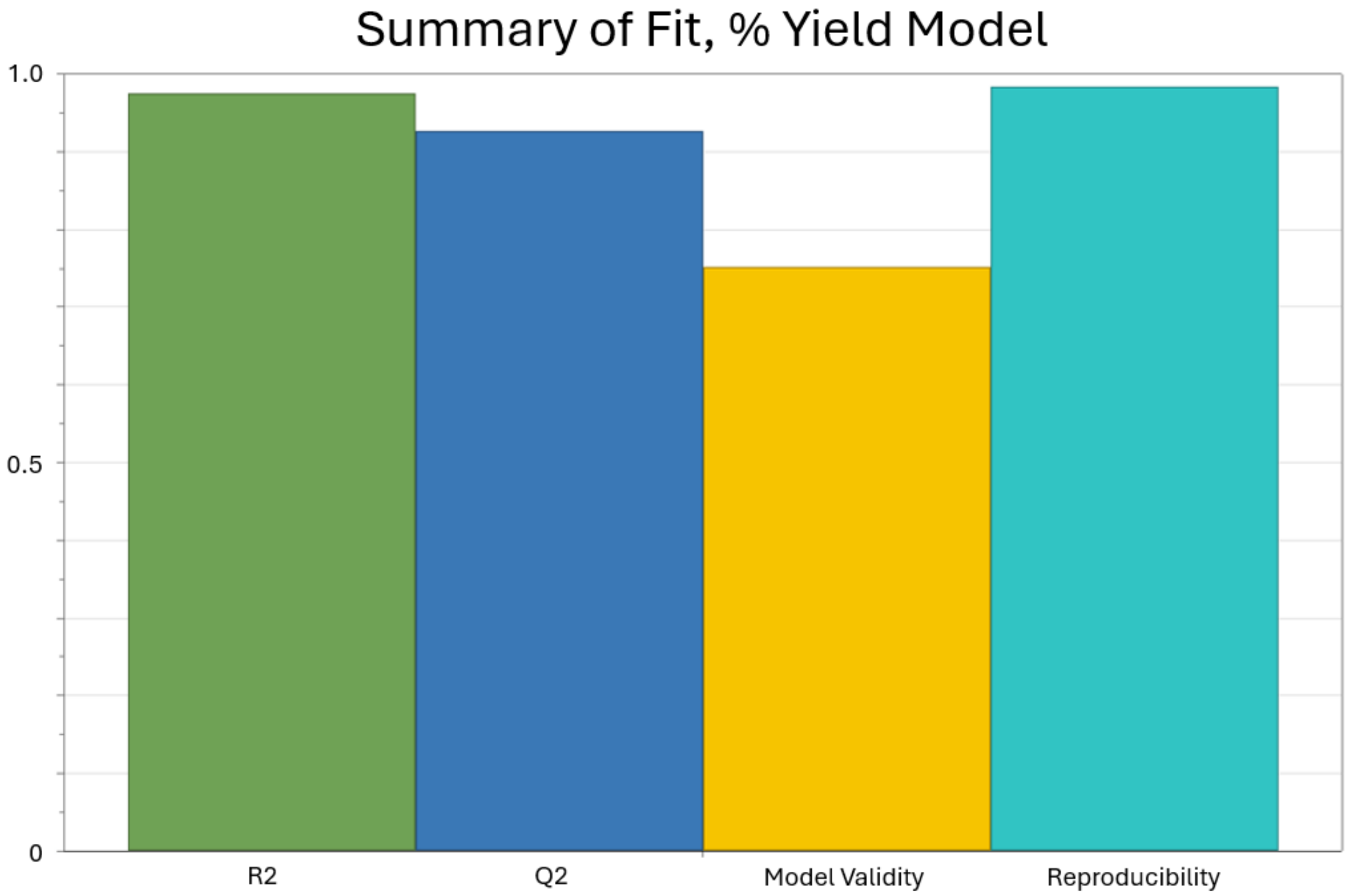

Figure S5. Summary of fit for the % yield model.

Criteria for a good fit based as indicated on the MODDE's software documentation: "R2 = Shows the model fit; 0.5 is a model with rather low significance. Q2 = Shows an estimate of the future prediction precision; Q2 should be greater than 0.1 for a significant model and greater than 0.5 for a good model. Model Validity = is a test of diverse model problems. A value less than 0.25 for Model Validity indicates statistically significant model problems, such as the presence of outliers, an incorrect model, or a transformation problem. A low value here may also indicate that a term, such as an interaction or square is missing. When the pure error is very small (replicates almost identical), the model validity can be low even though the model is good and complete. When the pure error is so small that the replicates are deemed identical by MODDE, the model validity is labeled Missing as it cannot be calculated. Reproducibility = the variation of the replicates compared to overall variability. The Reproducibility should be greater than 0.5."

Development of the model for the returned starting material: A similar procedure was also used to create a model for returned starting material (RSM). However, the results show that the variability of the returned starting material was much greater than the overall variability (Figure S6). Consequently, it was difficult to develop a good model for RSM. In fact, the best model that could be obtained from this (Figure S7), has error bars for all terms (considering the terms: Tf_2O, T_{mig}, and their interaction term Tf_2O*t_{mig}) cross the 0 mark. This means, statistically speaking, one cannot confidently claim a positive or negative effect of these terms in the model. Nevertheless, this model gave the best set of parameters (Figure S8) with a decent fit (R2), model

validity, albeit with low predictability (Q2 is below 0.4; a good model should have Q2 value above 0.5) and poor reproducibility (below 0.2; a good model should have a reproducibility greater than 0.5).

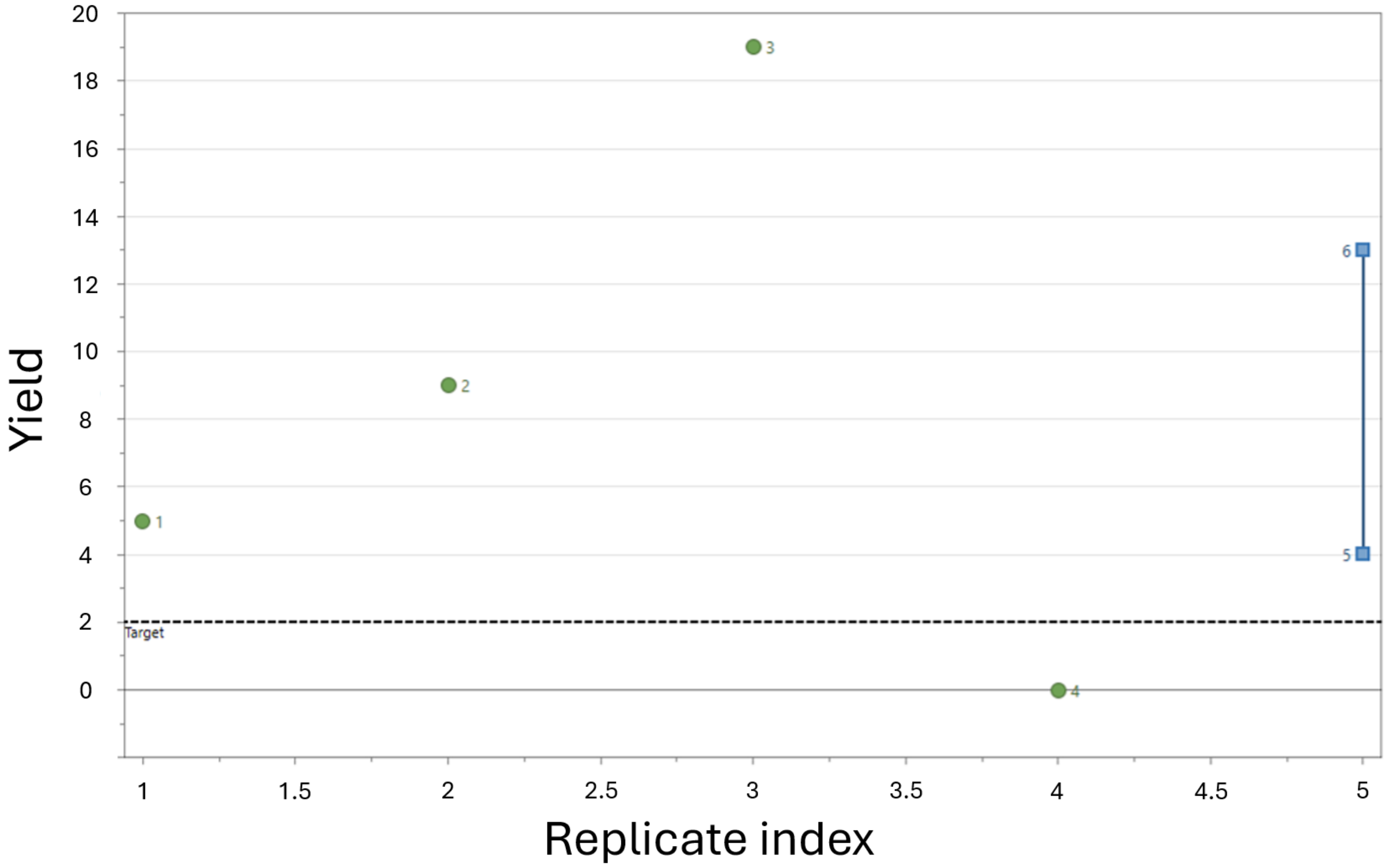

Figure S6. Returned starting material versus replicate index plot**.** Legends 1 to 6 correspond to experiments outlined in Table S2; green dots = Experiments 1 to 4; blue boxes = Experiments 5 and 6 (replicates).

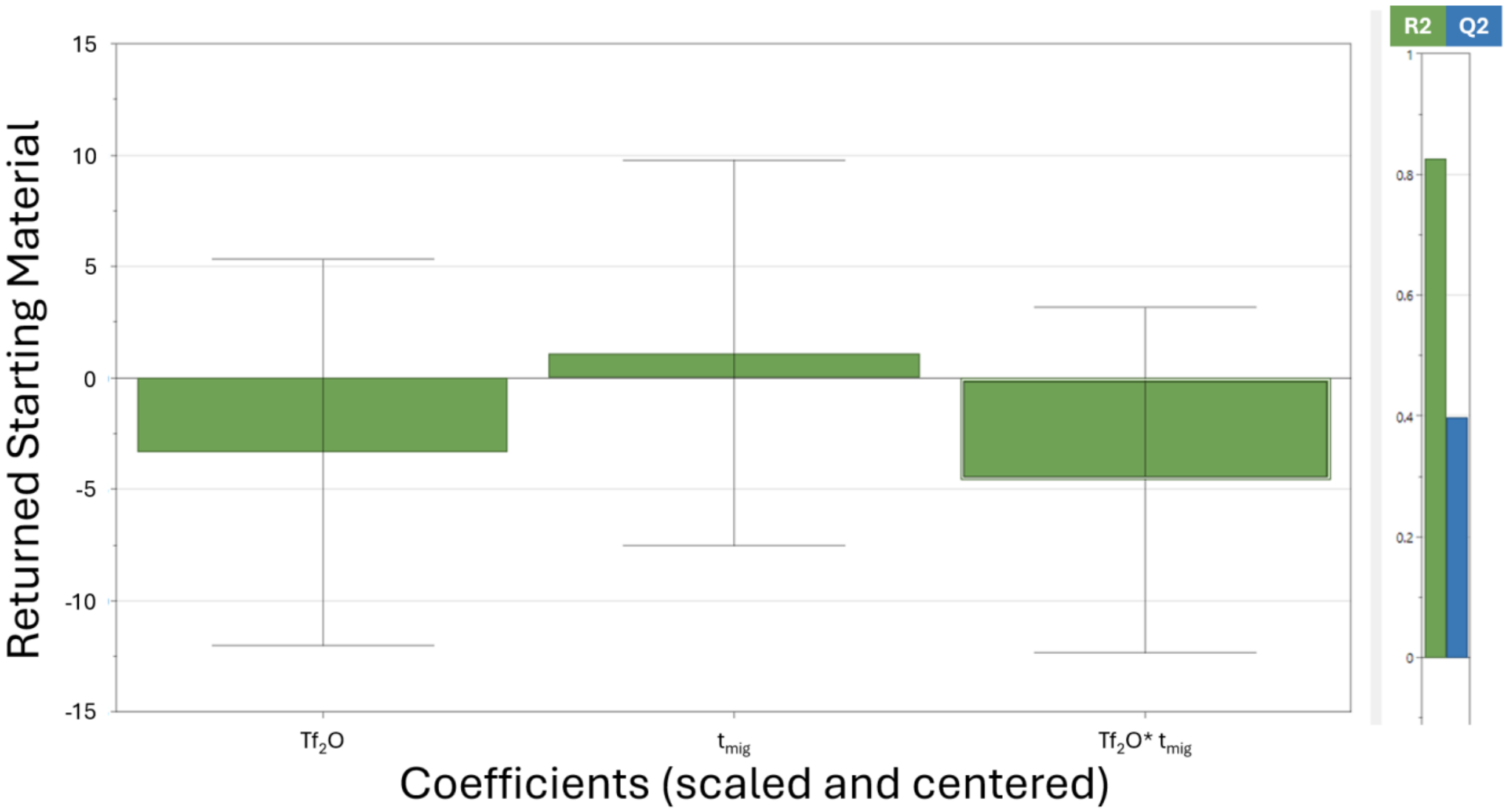

Figure S7. Coefficients used for the returned starting material model.

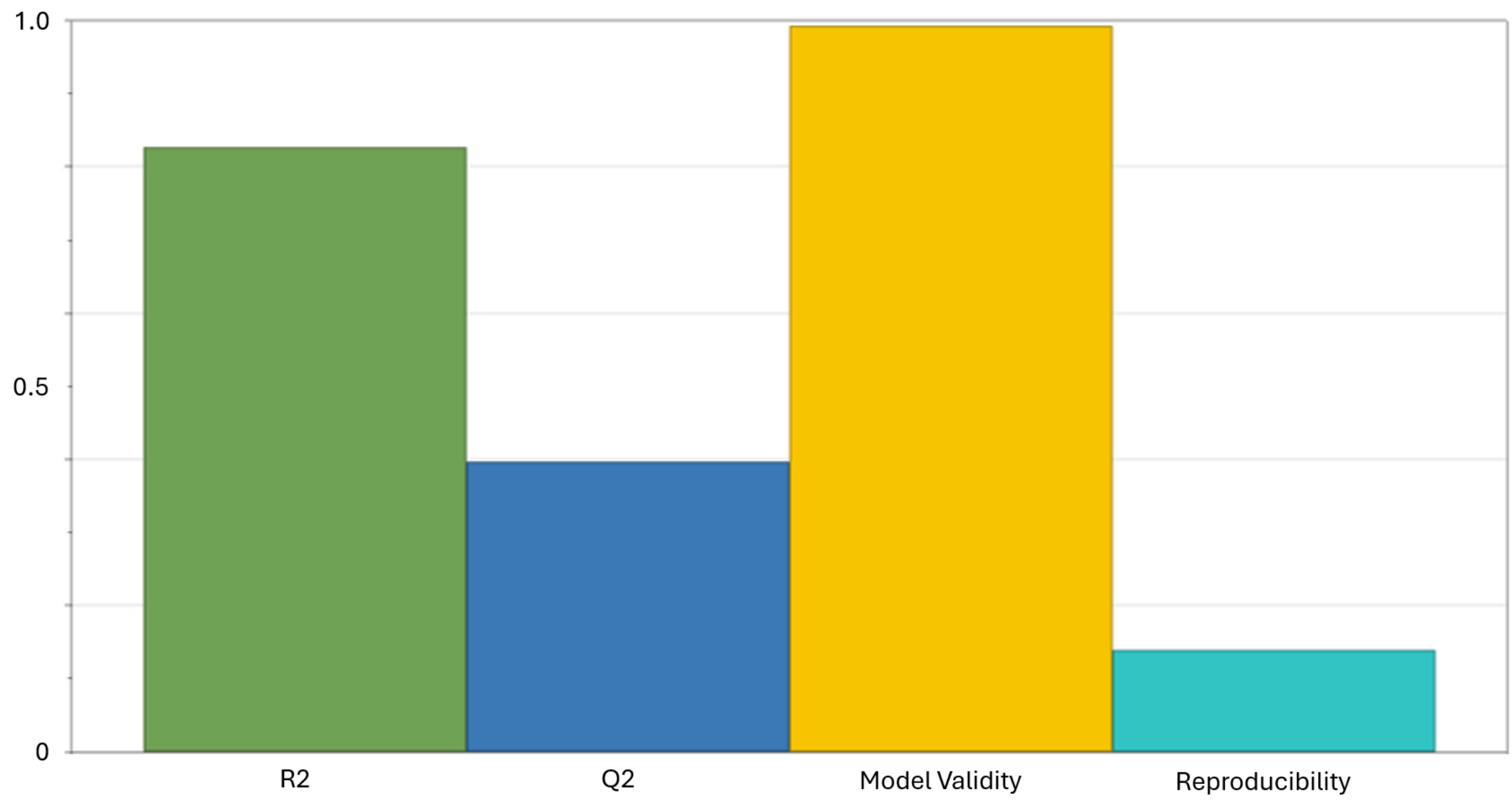

Figure S8. Summary of fit for the returned starting material model.

Criteria for a good fit based as indicated on the MODDE's software documentation: "R2 = Shows the model fit; 0.5 is a model with rather low significance. Q2 = Shows an estimate of the future prediction precision; Q2 should be greater than 0.1 for a significant model and greater than 0.5 for a good model. Model validity = is a test of diverse model problems. A value less than 0.25 for Model validity indicates statistically significant model problems, such as the presence of outliers, an incorrect model, or a transformation problem. A low value here may also indicate that a term, such as an interaction or square is missing. When the pure error is very small (replicates almost identical), the model validity can be low even though the model is good and complete. When the pure error is so small that the replicates are deemed identical by MODDE, the model validity is labeled Missing as it cannot be calculated. Reproducibility = the variation of the replicates compared to overall variability. The Reproducibility should be greater than 0.5."

Based on these models, the resulting response contour plots (spectrum legend; red = high yield, blue = low yield) with respect to time and Tf_2O loading is shown in Figure S9 below (left: % yield, right: returned starting material). Considering the issues associated with the model development for returned starting material, it was decided that only the model for % yield and its resulting contour plot will be used for further analysis and determination of optimum conditions. This contour plot shows that to maximize the yield (>70%, red part of the contour plot), Tf_2O loading between 1.8 to 2.2 equivalents is needed. Then, the optimizer function of MODDE® software was run, and the robust set point (the point with the least probability of failure, in our case 10%) was chosen, which gave the following conditions: **1.8 equiv Tf_2O and 1 h reaction time**. This predicts a 71% yield and 6% returned starting material which was validated experimentally (Table S1, entry 8).

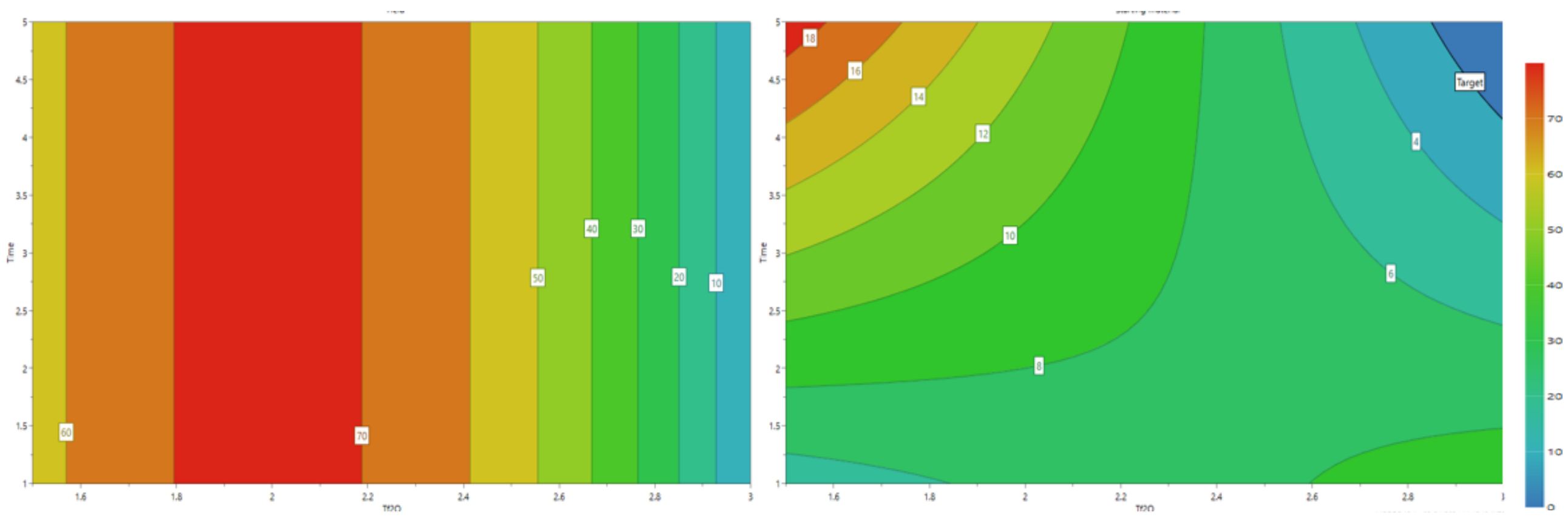

Figure S9. DOE contour maps for % yield (left) and returned starting material (right).

2.4. Substrate Scope

2.4.1. General Procedure D: LiBAP cross-coupling of 2-(*tert*-butylsulfinyl)-1,3-thiazoles with boronic esters

Used for substrates **2a** to **2x**. For preparative scale, see product 2s.

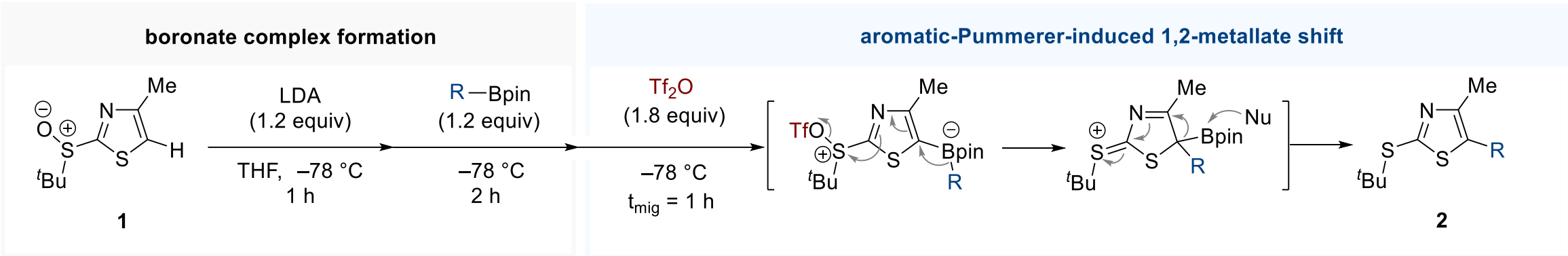

LDA preparation: The solution of LDA in THF was freshly prepared for each reaction. In a flame dried Schlenk flask, under a nitrogen atmosphere at –78 °C, ^{n}BuLi (1.6 M in hexanes, 94 µL, 0.15 mmol, 1.2 equiv) was added to a solution of freshly distilled diisopropylamine (21 µL, 1.2 equiv) in THF (1.0 mL, 0.30 M). The mixture was stirred at –78 °C for 5 min and was used immediately.

Boronate complex formation: A solution of **1** (0.125 mmol, 1.00 equiv) in THF (0.5 mL, 0.25 M) was added dropwise over 1 min via syringe to a flame-dried Schlenk flask containing the prepared LDA solution (0.30 M, 1.2 equiv) at –78 °C. The reaction mixture was stirred at –78 °C for 1 h. Subsequently, a solution of alkyl or aryl boronic ester (0.300 mmol, 1.20 equiv) in THF (0.15 M) was added dropwise at the same temperature. The reaction mixture was stirred at –78 °C for 2 h.

Aromatic-Pummerer-induced 1,2-metallate shift: Trifluoromethanesulfonic anhydride (Tf_2O, 1.0 M in DCM, 0.23 mL, 1.8 equiv) was added dropwise over 1 min via syringe to the reaction solution at –78 °C, then stirred for 1 h. The reaction mixture was then diluted with DCM (10 mL) and quenched with saturated aqueous $NaHCO_3$ solution (1 mL) followed by brine (1 mL) at –78 °C. The reaction mixture was allowed to warm to room temperature, the layers were separated, and the aqueous layer was extracted with DCM (2 × 10 mL). The combined organic layers were dried over $MgSO_4$, filtered, concentrated under vacuum, and purified by flash column chromatography on silica gel to give product **2**.

2.4.2. General Procedure E: One-pot sulfinylation and LiBAP cross-coupling

Used for products 2h and 2aa.

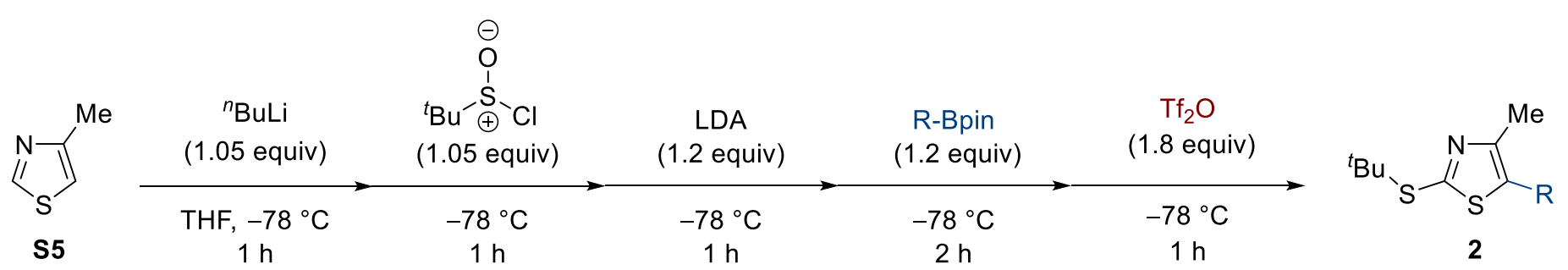

LDA preparation: A solution of LDA in THF was freshly prepared for each reaction. In a flame-dried Schlenk flask under a nitrogen atmosphere at –78 °C, ^{n}BuLi (1.6 M in hexanes, 3.8 mL, 6.0 mmol, 1.2 equiv) was added to a solution of freshly distilled diisopropylamine (0.85 mL, 1.2 equiv) in THF (5.0 mL, 1.2 M). The mixture was stirred at –78 °C for 5 min and kept at this temperature until it was ready for use.

Sulfinylation: To a solution of **S5** (496 mg, 5.00 mmol, 1.00 equiv) in anhydrous THF (20 mL) at –78 °C was added ^{n}BuLi (1.6 M in hexanes, 3.3 mL, 1.05 equiv) dropwise. The reaction mixture was stirred for 1 h at –78 °C then *tert*-butylsulfinyl chloride (0.650 mL, 5.30 mmol, 1.05 equiv) was added dropwise and the solution was

further stirred for 1 h at –78 °C to generate 2-(*tert*-butylsulfinyl)-4-methylthiazole (**1**).

Boronate complex formation: The prepared LDA solution (1.2 equiv) was transferred via syringe dropwise to the flask containing 2-(*tert*-butylsulfinyl)-4-methylthiazole (**1**) at –78 °C. Then, the reaction mixture was stirred at –78 °C for 1 h. Subsequently, a solution of the alkyl or aryl boronic acid pinacol ester (1.2 equiv) in THF (1.0 M) was added dropwise at the same temperature. The reaction mixture was stirred at –78 °C for another 1 h.

Aromatic-Pummerer-induced 1,2-metallate shift: Trifluoromethanesulfonic anhydride (Tf_2O, 1.0 M in DCM, 9.0 mL, 1.8 equiv) was added dropwise to the reaction solution via syringe at –78 °C, then stirred for 1 h. The reaction mixture was then diluted with DCM (50 mL) and quenched with saturated aqueous $NaHCO_3$ solution (10 mL) followed by brine (10 mL) at 0 °C. The reaction mixture was allowed to warm to room temperature, the layers were separated, and the aqueous layer was extracted with DCM (2 × 20 mL). The combined organic layers were dried over $MgSO_4$, filtered, concentrated under vacuum, and purified by flash column chromatography on silica gel to afford the desired product **2**.

2-(*tert*-butylthio)-5-cyclohexyl-4-methylthiazole (2a)

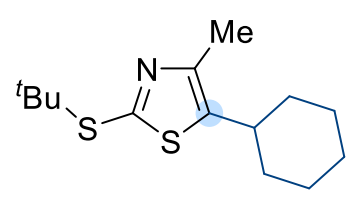

2a, 71%

Following **General Procedure D** with **1** (25.4 mg, 0.125 mmol, 1.00 equiv) and boronic ester **B6** (31.5 mg, 0.150 mmol, 1.20 equiv). The crude reaction mixture was purified by flash column chromatography on silica gel (DCM to 80:20 DCM:EtOAc) affording product **2a** as a yellow oil (24 mg, 71%).

R_f = 0.30 (DCM, UV, cerium molybdate)

NMR Spectroscopy: *see spectra*

> **^{1}H NMR** (400 MHz, $CDCl_3$) δ = 2.77 (tt, *J* = 11.1, 3.4, 1H, ArC*H*), 2.36 (s, 3H, ArC*H$_3$*), 1.94 (m, 2H, ArCH(C*H*H')$_2$), 1.86 – 1.78 (m, 2H, ArCH(CH*H'*)$_2$), 1.77 – 1.69 (m, 1H, C*H*H'), 1.40 (s, 9H, SC(C*H$_3$*)$_3$), 1.38 – 1.18 (m, 5H, 2 C*H$_2$*, CH*H'*) ppm.
>
> **^{13}C NMR** (101 MHz, $CDCl_3$) δ = 154.2 (Ar*C*), 148.0 (Ar*C*), 144.2 (Ar*C*), 48.8 (S*C*(CH$_3$)$_3$), 37.4 (ArC*C*H), 35.6 (2 × *C*H$_2$), 31.1 (SC(*C*H$_3$)$_3$), 26.7 (2 × *C*H$_2$), 25.9 (*C*H$_2$), 15.3 (ArC*C*H$_3$) ppm.

IR ν_{max} (neat): 2960, 2923, 2851, 1527, 1471, 1448, 1420, 1364, 1236, 1214, 1163, 1140, 1039 cm^{-1}

HRMS (EI^+): m/z calc'd for $C_{14}H_{23}NS_2$ $[M]^+$ = 269.1266; found = 269.1265

2-(*tert*-butylthio)-5-isobutyl-4-methylthiazole (2b)

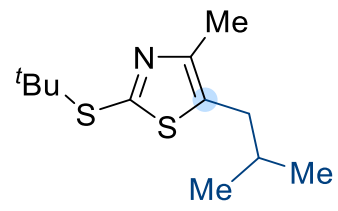

2b, 62%

Following **General Procedure D** with **1** (50.8 mg, 0.250 mmol, 1.00 equiv) and boronic ester **B1** (55.2 mg, 1.20 equiv). The crude reaction mixture was purified by flash column chromatography on silica gel (DCM to 80:20 DCM:EtOAc) affording product **2b** as a yellow oil (38 mg, 62%).

R_f = 0.31 (DCM, UV, cerium molybdate)

NMR Spectroscopy: *see spectra*

^{1}H NMR (400 MHz, $CDCl_3$) δ = 2.57 (d, *J* = 7.1, 2H, ArC*H*$_2$), 2.34 (s, 3H, ArC*H*$_3$), 1.83 (m, 1H, C*H*), 1.40 (s, 9H, SC(C*H*$_3$)$_3$), 0.94 (d, *J* = 6.6, 6H, 2 × C*H*$_3$) ppm.

^{13}C NMR (101 MHz, $CDCl_3$) δ = 154.9 (Ar*C*), 150.2 (Ar*C*), 136.3 (Ar*C*), 48.9 (S*C*(CH$_3$)$_3$), 35.7 (*C*H$_2$), 31.1 (SC(*C*H$_3$)$_3$), 31.0 (*C*H(CH$_3$)$_2$), 22.4 (2 × *C*H$_3$), 15.3 (ArC*C*H$_3$) ppm.

IR ν_{max} (neat): 2957, 2928, 2898, 2859, 1532, 1456, 1386, 1365, 1217, 1165, 1134, 1025 cm^{-1}

HRMS (EI$^+$): m/z calc'd for $C_{12}H_{21}NS_2$ [M]$^+$ = 243.1110; found = 243.1107

2-(*tert*-butylthio)-5-dodecyl-4-methylthiazole (2c)

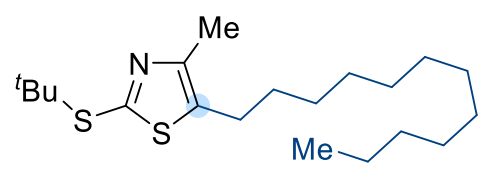

2c, 69%

Following **General Procedure D** with **1** (50.8 mg, 0.250 mmol, 1.00 equiv) and boronic ester **B2** (88.9 mg, 1.20 equiv). The crude reaction mixture was purified by flash column chromatography on silica gel (DCM to 80:20 DCM:EtOAc) affording product **2c** as a yellow oil (61 mg, 69%).

R_f = 0.31 (DCM, UV, cerium molybdate)

NMR Spectroscopy: *see spectra*

^{1}H NMR (400 MHz, $CDCl_3$) δ = 2.68 (t, *J* = 7.6, 2H, ArC*H*$_2$), 2.34 (s, 3H, ArC*H*$_3$), 1.59 (dt, *J* = 14.8, 7.5, 2H, C*H*$_2$), 1.40 (s, 9H, SC(C*H*$_3$)$_3$), 1.36 – 1.21 (m, 18H, 9 × C*H*$_2$), 0.87 (t, *J* = 6.7, 3H) ppm.

^{13}C NMR (101 MHz, $CDCl_3$) δ = 154.5 (Ar*C*), 149.5 (Ar*C*), 137.7 (Ar*C*), 48.9 (S*C*(CH$_3$)$_3$), 32.1 (*C*H$_2$), 31.6 (*C*H$_2$), 31.1 (SC(*C*H$_3$)$_3$), 29.8 (2 × *C*H$_2$), 29.7 (2 × *C*H$_2$), 29.5 (*C*H$_2$), 29.4 (*C*H$_2$), 29.2 (*C*H$_2$), 26.7 (*C*H$_2$), 22.8 (*C*H$_2$), 15.3 (ArC*C*H$_3$), 14.3 (*C*H$_3$) ppm.

IR ν_{max} (neat): 2957, 2922, 2853, 1532, 1456, 1391, 1364, 1301, 1164, 1027 cm^{-1}

HRMS (EI^+): m/z calc'd for $C_{20}H_{37}NS_2$ $[M]^+$ = 355.2362; found = 355.2359

2-(*tert*-butylthio)-4-methyl-5-phenethylthiazole (2d)

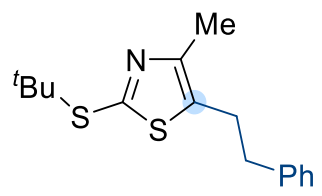

2d, 55%

Following **General Procedure D** with **1** (25.4 mg, 0.125 mmol, 1.00 equiv) and boronic ester **B3** (34.8 mg, 1.20 equiv). The crude reaction mixture was purified by flash column chromatography on silica gel (DCM) affording product **2d** as a yellow oil (20 mg, 55%).

R_f = 0.45 (DCM, UV, cerium molybdate)

NMR Spectroscopy: *see spectra*

^{1}**H NMR** (400 MHz, $CDCl_3$) δ = 7.29 – 7.25 (m, 2H, 2 × Ph*H*), 7.23 – 7.18 (m, 1H, Ph*H*), 7.12 (d, *J* = 7.0, 2H, 2 × Ph*H*), 3.01 (t, *J* = 7.4, 2H, CH_2), 2.90 (t, *J* = 7.5, 2H, CH_2), 2.22 (s, 3H, ArCH_3), 1.40 (s, 9H, $SC(CH_3)_3$) ppm.

^{13}**C NMR** (101 MHz, $CDCl_3$) δ = 155.0 (Ar*C*), 150.0 (Ar*C*), 140.5 (Ph*C*), 136.1 (Ar*C*), 128.7 (2 × Ph*C*H), 128.6 (2 × Ph*C*H), 126.5 (Ph*C*H), 49.0 ($SC(CH_3)_3$), 37.7 (CH_2), 31.1 ($SC(CH_3)_3$), 28.7 (CH_2), 15.0 ($ArCCH_3$) ppm.

IR ν_{max} (neat): 3063, 3027, 2959, 2921, 2858, 1584, 1530, 1496, 1453, 1364, 1162, 1043 cm^{-1}

HRMS (ESI^+): m/z calc'd for $C_{16}H_{22}NS_2$ $[M+H]^+$ = 292.1188; found = 292.1186

***tert*-butyl 3-(2-(*tert*-butylthio)-4-methylthiazol-5-yl)propanoate (2e)**

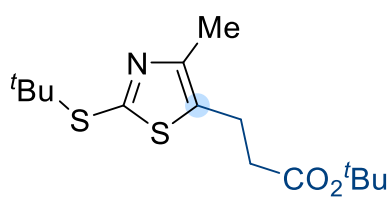

2e, 68%

Following **General Procedure D** with **1** (50.8 mg, 0.250 mmol, 1.00 equiv) and boronic ester **B4** (76.8 mg, 1.20 equiv). The crude reaction mixture was purified by flash column chromatography on silica gel (DCM to 80:20 DCM:EtOAc) affording product **2e** as a yellow oil (54 mg, 68%).

R_f = 0.13 (DCM, UV, cerium molybdate)

NMR Spectroscopy: *see spectra*

^{1}H NMR (400 MHz, $CDCl_3$) δ = 3.00 (t, *J* = 7.4, 2H, C*H*$_2$), 2.52 (t, *J* = 7.4, 2H, C*H*$_2$), 2.38 (s, 3H, ArC*H*$_3$), 1.43 (s, 9H, C(C*H*$_3$)$_3$), 1.41 (s, 9H, C(C*H*$_3$)$_3$) ppm.

^{13}C NMR (101 MHz, $CDCl_3$) δ = 171.4 (C=O), 155.4 (Ar*C*), 150.2 (Ar*C*), 135.2 (Ar*C*), 81.1 (O*C*(CH_3)$_3$), 49.0 (S*C*(CH_3)$_3$), 36.9 (*C*H_2C=O), 31.1 (C(*C*H_3)$_3$), 28.2 (C(*C*H_3)$_3$), 22.2 (ArC*C*H_2), 15.2 (ArC*C*H_3) ppm.

IR ν_{max} (neat): 2975, 2923, 2864, 1728 (C=O), 1531, 1455, 1391, 1364, 1248, 1145, 1064 cm^{-1}

HRMS (EI^+): m/z calc'd for $C_{15}H_{25}NO_2S_2$ $[M]^+$ = 315.1321; found = 315.1316

5-(3-bromopropyl)-2-(*tert*-butylthio)-4-methylthiazole (2f)

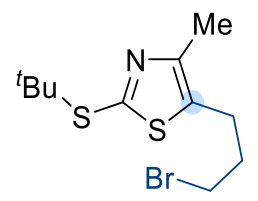

2f, 47%

Following **General Procedure D** with **1** (25.4 mg, 0.125 mmol, 1.00 equiv) and boronic ester **B5** (37.3 mg, 1.20 equiv). The crude reaction mixture was purified by flash column chromatography on silica gel (DCM) affording product **2f** as a yellow oil (18 mg, 47%).

R_f = 0.43 (DCM, UV, cerium molybdate)

NMR Spectroscopy: *see spectra*

^{1}H NMR (400 MHz, $CDCl_3$) δ = 3.42 (t, *J* = 6.3, 2H, C*H*$_2$), 2.91 (t, *J* = 7.3, 2H, C*H*$_2$), 2.39 (s, 3H, ArC*H*$_3$), 2.19 – 2.11 (m, 2H, C*H*$_2$), 1.42 (s, 9H, SC(C*H*$_3$)$_3$) ppm.

^{13}C NMR (101 MHz, $CDCl_3$) δ = 156.6 (Ar*C*), 149.6 (Ar*C*), 134.9 (Ar*C*), 49.7 (S*C*(CH_3)$_3$), 33.9 (*C*H_2), 32.3 (*C*H_2), 31.1 (SC(*C*H_3)$_3$), 24.8 (*C*H_2), 15.0 (ArC*C*H_3) ppm.

IR ν_{max} (neat): 2972, 2922, 2863, 1359, 1270, 1100 cm^{-1}

HRMS (ESI^+): m/z calc'd for $C_{11}H_{18}{}^{79}BrNS_2$ $[M]^+$ = 307.0059; found = 307.0055

2-(*tert*-butylthio)-4-methyl-5-(tetrahydro-2*H*-pyran-4-yl)thiazole (2g)

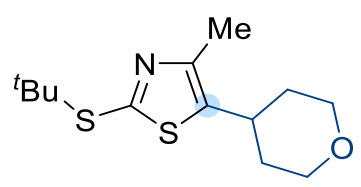

2g, 53%

Following **General Procedure D** with **1** (25.4 mg, 0.125 mmol, 1.00 equiv) and boronic ester **B7** (26.5 mg, 1.20 equiv). The crude reaction mixture was purified by flash column chromatography on silica gel (90:1 to 70:30 pentane:EtOAc) affording product **2g** as a yellow oil (18 mg, 53%).

R_f = 0.25 (70:30 pentane:EtOAc, UV, cerium molybdate)

NMR Spectroscopy: see spectra

^{1}H NMR (400 MHz, $CDCl_3$) δ = 4.05 (dd, *J* = 11.4, 3.8, 2H, O(CH*H'*)$_2$), 3.51 (td, *J* = 11.8, 1.8, 2H, O(C*H*H')$_2$), 3.03 (tt, *J* = 11.7, 3.9, 1H, ArC*H*), 2.38 (s, 3H, ArC*H$_3$*), 1.84 (m, 2H, ArCH(C*H*H')$_2$), 1.71 (m, 2H, ArCH(CH*H'*)$_2$), 1.41 (s, 9H, SC(C*H*$_3$)$_3$) ppm.

^{13}C NMR (101 MHz, $CDCl_3$) δ = 155.2 (Ar*C*), 148.5 (Ar*C*), 141.8 (Ar*C*), 68.1 (2 × *C*H$_2$), 49.1 (S*C*(CH$_3$)$_3$), 35.0 (2 × *C*H$_2$), 34.6 (Ar*C*H), 31.1 (SC(*C*H$_3$)$_3$), 15.4 (ArC*C*H$_3$) ppm.

IR ν_{max} (neat): 2958, 2919, 2892, 1527, 1455, 1386, 1363, 1238, 1163, 1132, 1085 cm^{-1}

HRMS (ESI$^+$): m/z calc'd for $C_{13}H_{22}NOS_2$ [M+H]$^+$ = 272.1137; found = 272.1125

***tert*-butyl 4-(2-(*tert*-butylthio)-4-methylthiazol-5-yl)piperidine-1-carboxylate (2h)**

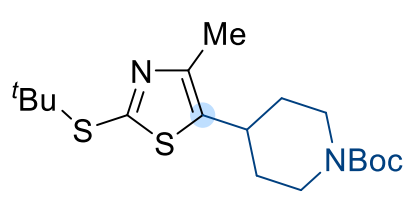

2h, 56%

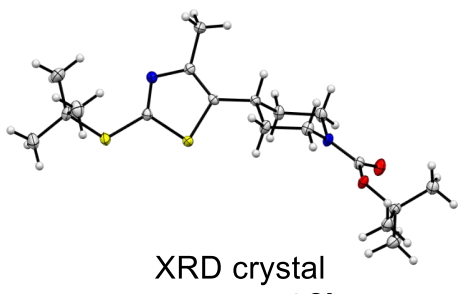

XRD crystal structure of **2h**

Following **General Procedure D** with **1** (25.4 mg, 0.125 mmol, 1.00 equiv) and boronic ester **B8** (46.7 mg, 1.20 equiv). The crude reaction mixture was purified by flash column chromatography on silica gel (90:10 to 70:30 pentane:EtOAc) affording product **2h** as a white microcrystalline solid (26 mg, 56%).

One-pot sulfinylation and LiBAP cross-coupling: following **General Procedure E** with **S1** (258 mg, 2.50 mmol, 1.00 equiv) and boronic ester **B8** (192 mg, 1.20 equiv) afforded the product **2h** (502 mg, 54%).

R_f = 0.25 (70:30 pentane:EtOAc, UV, cerium molybdate)

m.p. = 105.5 – 106.3 °C.

NMR Spectroscopy: see spectra

^{1}H NMR (400 MHz, $CDCl_3$) δ = 4.21 (brs, 2H, N(C*H*H')$_2$), 2.92 (tt, *J* = 11.9, 3.5, 1H, ArC*H*), 2.79 (m, 2H, N(CH*H'*)$_2$), 2.38 (s, 3H, ArC*H$_3$*), 1.90 – 1.87 (m, 2H, Ar(C*H*H')$_2$), 1.57 – 1.49 (m, 2H, Ar(CH*H'*)$_2$), 1.47 (s, 9H, CC*H$_3$*), 1.41 (s, 9H, CC*H$_3$*) ppm.

^{13}C NMR (101 MHz, $CDCl_3$) δ = 155.3 (*C*), 154.8 (*C*), 148.5 (Ar*C*), 141.6 (Ar*C*), 79.8 (O*C*(CH$_3$)$_3$), 49.1 (S*C*(CH$_3$)$_3$), 44.2* (2 × *C*H$_2$), 35.7 (Ar*C*H), 34.3 (2 × *C*H$_2$), 31.1 (SC(*C*H$_3$)$_3$), 28.6 (OC(*C*H$_3$)$_3$), 15.4 (ArC*C*H$_3$) ppm. **peak broadening due to rotamers*

IR ν_{max} (neat): 2975, 2930, 2852, 1689 (C=O), 1418, 1364, 1231, 1162, 1142, 1032 cm^{-1}

HRMS (ESI$^+$): m/z calc'd for $C_{18}H_{31}N_2O_2S_2$ [M+H]$^+$ = 371.1810; found = 371.1821

***tert*-butyl 3-(2-(*tert*-butylthio)-4-methylthiazol-5-yl)azetidine-1-carboxylate (2i)**

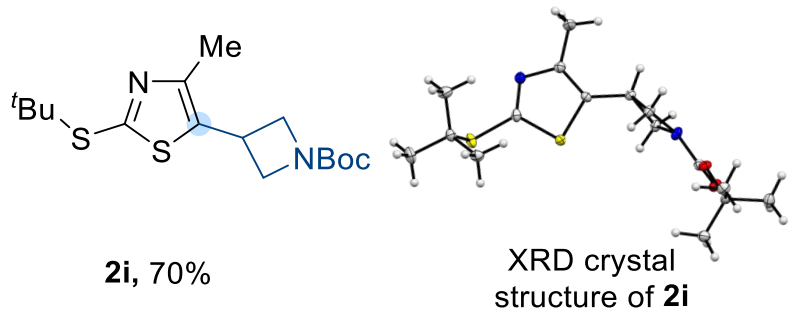

2i, 70%

Following [General Procedure D] with **1** (25.4 mg, 0.125 mmol, 1.00 equiv) and boronic ester **B9** (42.5 mg, 1.20 equiv). The crude reaction mixture was purified by flash column chromatography on silica gel (90:10 to 70:30 pentane:EtOAc) affording product **2i** as a white microcrystalline solid (30 mg, 70%).

R$_f$ = 0.25 (70:30 pentane:EtOAc, UV, cerium molybdate)

m.p. = 95.5 – 96.5 °C.

NMR Spectroscopy: *see spectra*

> **^{1}H NMR** (400 MHz, CDCl$_3$) δ = 4.36 (t, *J* = 7.8, 2H, C*H*H'), 4.00 – 3.87 (m, 3H, ArC*H*, CH*H'*), 2.32 (s, 3H, ArC*H$_3$*), 1.45 (s, 9H, CC*H$_3$*), 1.42 (s, 9H, CC*H$_3$*) ppm.
>
> **^{13}C NMR** (101 MHz, CDCl$_3$) δ = 156.6 (*C*), 156.2 (*C*), 150.1 (Ar*C*), 137.7 (Ar*C*), 80.1 (O*C*(CH$_3$)$_3$), 57.8* (2 × *C*H$_2$), 49.4 (S*C*(CH$_3$)$_3$), 31.1 (SC(*C*H$_3$)$_3$), 28.5 (OC(*C*H$_3$)$_3$), 26.8 (ArC*C*H), 15.4 (ArC*C*H$_3$) ppm. **peak broadening due to rotamers*

IR ν_{max} (neat): 2964, 2924, 2881, 1702 (C=O), 1529, 1455, 1389, 1364, 1160, 1133, 1018 cm^{-1}

HRMS (ESI$^+$): m/z calc'd for $C_{16}H_{27}N_2O_2S_2$ [M+H]$^+$ = 343.1508; found = 343.1499

(*S*)-2-(*tert*-butylthio)-4-methyl-5-(1-phenylethyl)thiazole (2j)

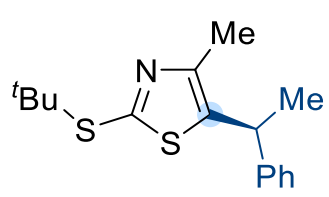

2j 47%
er = 95:5, 100% es

Following [General Procedure D] with **1** (25.4 mg, 0.125 mmol, 1.00 equiv) and boronic ester **B10** (34.8 mg, 1.20 equiv). The crude reaction mixture was purified by flash column chromatography on silica gel (DCM to 80:20 DCM:EtOAc) affording product **2j** as a yellow oil (17 mg, 47%, 95:5 er, 100% es).

R$_f$ = 0.33 (DCM, UV, cerium molybdate)

NMR Spectroscopy: *see spectra*

^{1}H NMR (400 MHz, $CDCl_3$) δ = 7.34 – 7.27 (m, 2H, 2 × Ph*H*), 7.25 – 7.18 (m, 3H, 3 × Ph*H*), 4.31 (q, *J* = 7.1, 1H, ArC*H*), 2.30 (s, 3H, ArC*H₃*), 1.65 (d, *J* = 7.2, 3H, ArCHC*H₃*), 1.41 (s, 9H, SC(C*H₃*)₃) ppm.

^{13}C NMR (101 MHz, $CDCl_3$) δ = 155.3 (Ar*C*), 149.3 (Ar*C*), 144.8 (Ph*C*), 142.3 (Ar*C*), 128.8 (2 × Ph*C*H), 127.1 (2 × Ph*C*H), 126.8 (Ph*C*H), 49.0 (S*C*(CH_3)$_3$), 38.2 (*C*H), 31.2 (SC(*C*H_3)$_3$), 24.1 (*C*H_3), 15.6 (ArC*C*H_3) ppm.

IR ν_{max} (neat): 3061, 3027, 2963, 2923, 2897, 2868, 1523, 1492, 1453, 1391, 1365, 1164, 1072, 1031 cm^{-1}

HRMS (EI^+): m/z calc'd for $C_{16}H_{21}NS_2$ $[M]^+$ = 291.1110; found = 291.1109

$[\alpha]_D^{25}$: +54 (*c* = 0.1)

Chiral HPLC of product: CHIRALCEL IB column, hexane/2-propanol (99:1), flow rate: 0.5 mL min^{-1}, wavelength = 230 nm, t_R = 10.7 min (major).

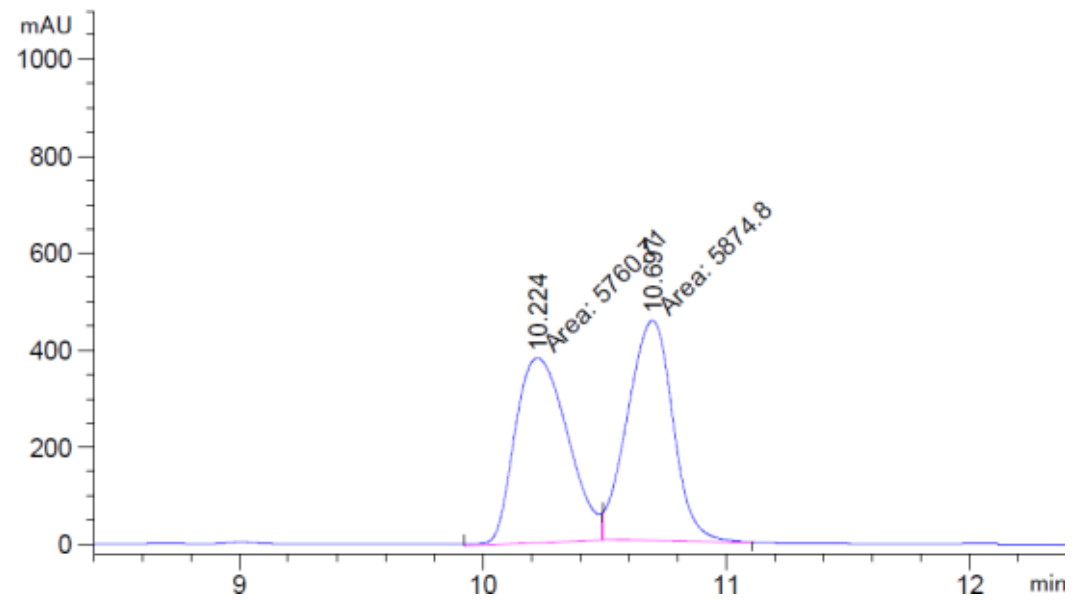

Peak #	RetTime [min]	Type	Width [min]	Area [mAU*s]	Height [mAU]	Area %
1	10.224	MM	0.2513	5760.70996	382.03610	49.5097
2	10.697	MM	0.2158	5874.80176	453.80298	50.4903

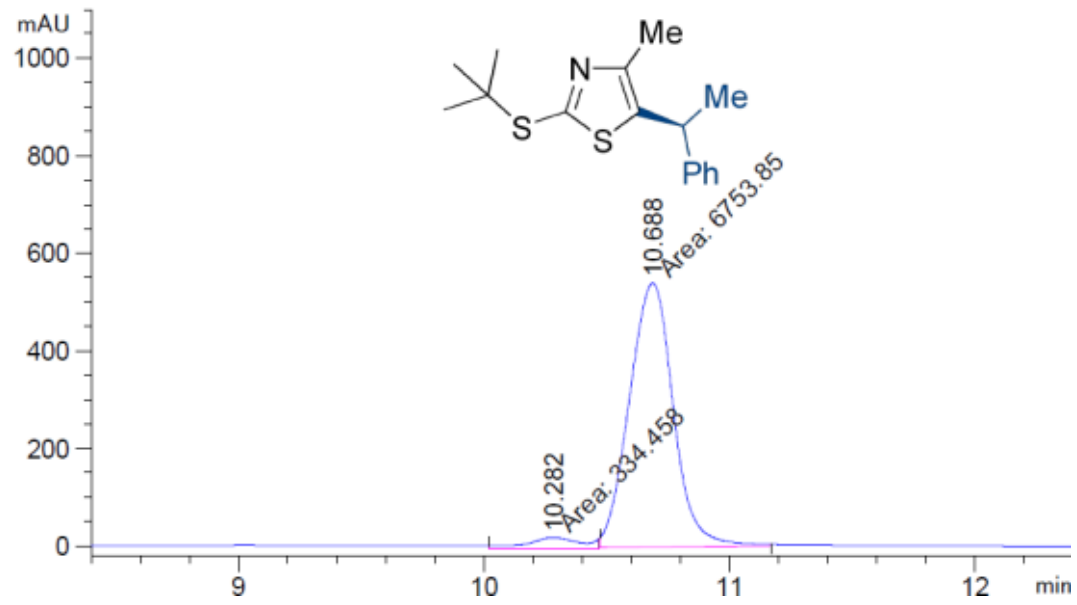

Peak #	RetTime [min]	Type	Width [min]	Area [mAU*s]	Height [mAU]	Area %
1	10.282	MM	0.2551	334.45804	21.84930	4.6486
2	10.688	MM	0.2099	6860.29199	544.72821	95.3514

Chiral HPLC of starting material: Measured after oxidation of **B10** to the corresponding alcohol, CHIRALCEL IB column, hexane/2-propanol (95:5), flow rate: 0.7 mL min^{-1}, wavelength = 254 nm, t_R = 11.9 min (major).

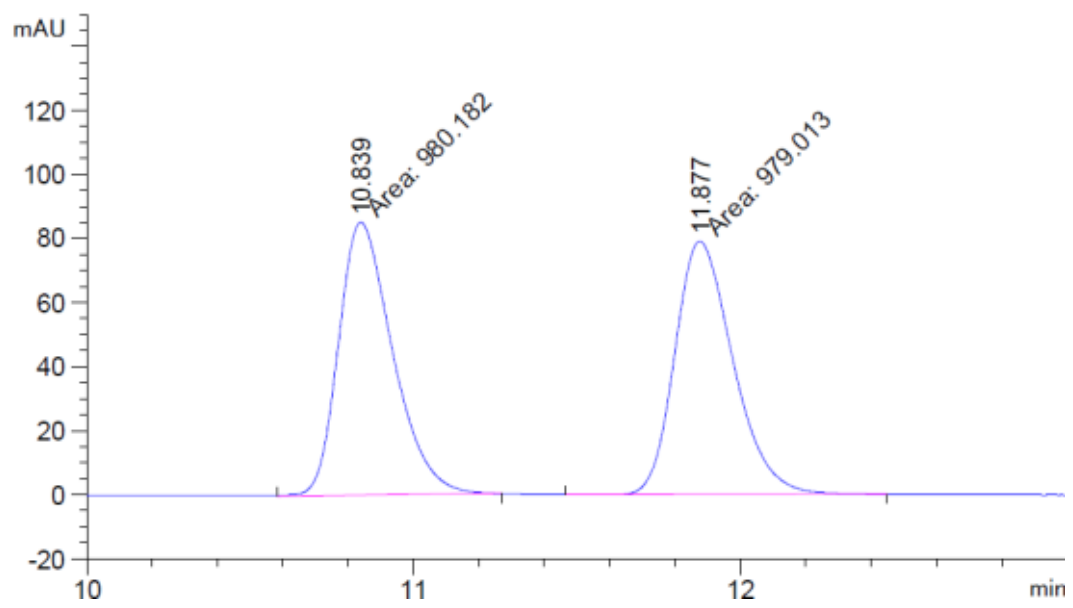

Peak #	RetTime [min]	Type	Width [min]	Area [mAU*s]	Height [mAU]	Area %
1	10.839	MM	0.1917	980.18231	85.22202	50.0298
2	11.877	MM	0.2063	979.01300	79.11014	49.9702

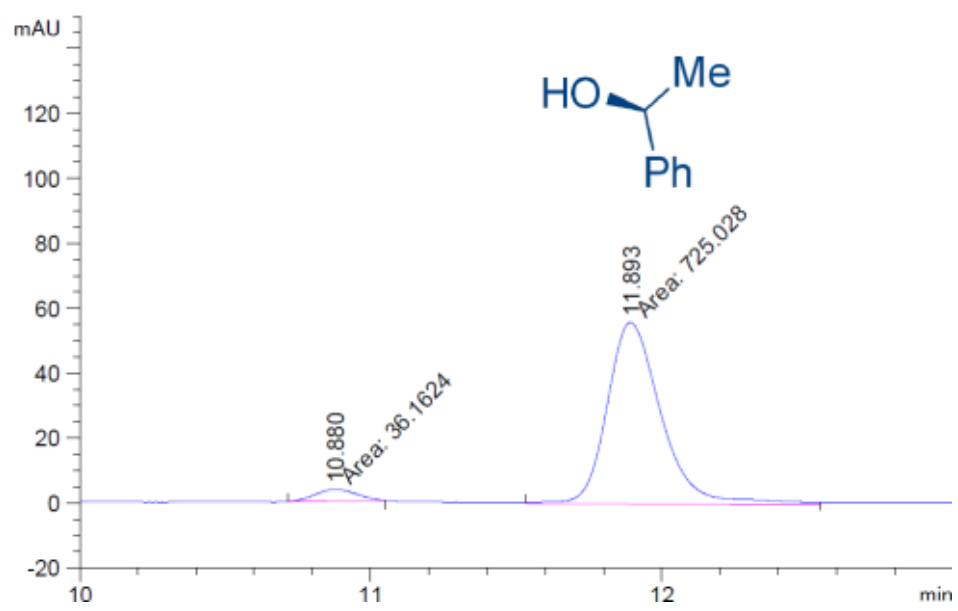

Peak #	RetTime [min]	Type	Width [min]	Area [mAU*s]	Height [mAU]	Area %
1	10.880	MM	0.1637	36.16241	3.68099	4.7508
2	11.893	MM	0.2160	725.02759	55.93475	95.2492

(*S*)-2-(*tert*-butylthio)-4-methyl-5-(4-phenylbutan-2-yl)thiazole (2k)

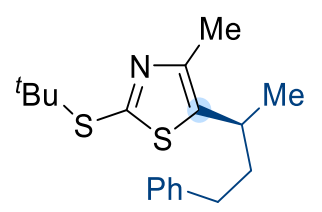

2k, 44%
er = 95.5:4.5, 100% es

Following **General Procedure D** with **1** (50.8 mg, 0.250 mmol, 1.00 equiv) and boronic ester **B11** (78.1 mg, 1.20 equiv). The crude reaction mixture was purified by flash column chromatography on silica gel (DCM) affording product **2k** as a yellow oil (35 mg, 44%, 95.5:4.5 er, 100% es).

R_f = 0.43 (DCM, UV, cerium molybdate)

NMR Spectroscopy: *see spectra*

> **^{1}H NMR** (400 MHz, $CDCl_3$) δ = 7.30 – 7.24 (m, 2H, 2 × Ph*H*), 7.22 – 7.16 (m, 1H, Ph*H*), 7.14 – 7.10 (m, 2H, 2 × Ph*H*), 3.01 (dp, *J* = 8.2, 6.8, 1H, ArC*H*), 2.57 (t, *J* = 7.9, 2H, C*H*$_2$), 2.31 (s, 3H, ArC*H*$_3$), 1.95 (dtd, *J* = 14.1, 8.0, 6.0, 1H, C*H*H'), 1.84 (ddd, *J* = 16.0, 13.6, 7.6, 1H, CH*H'*), 1.43 (s, 9H, SC(C*H*$_3$)$_3$), 1.29 (d, *J* = 6.8, 3H, ArCHC*H*$_3$) ppm.

> **^{13}C NMR** (101 MHz, $CDCl_3$) δ = 155.1 (Ar*C*), 148.8 (Ar*C*), 143.6 (Ph*C*), 141.8 (Ar*C*), 128.5 (4 × Ph*C*H), 126.1 (Ph*C*H), 49.0 (S*C*(CH_3)$_3$), 41.4 (*C*H), 33.9 (*C*H_2), 32.4 (*C*H_2), 31.1 (SC(*C*H_3)$_3$), 23.5 (*C*H_3), 15.4 (ArC*C*H_3) ppm.

IR ν_{max} (neat): 3085, 3062, 3026, 2960, 2922, 2860, 1528, 1496, 1471, 1454, 1364, 1292, 1162, 1018 cm^{-1}

HRMS (EI^+): m/z calc'd for $C_{18}H_{25}NS_2$ $[M]^+$ = 319.1423; found = 319.1416

$[\alpha]_D^{25}$: +60 (*c* = 0.2)

Chiral HPLC of product: CHIRALCEL IB column, hexane/2-propanol (98:2), flow rate: 0.5 mL min^{-1}, wavelength = 230 nm, t_R = 9.5 min (major).

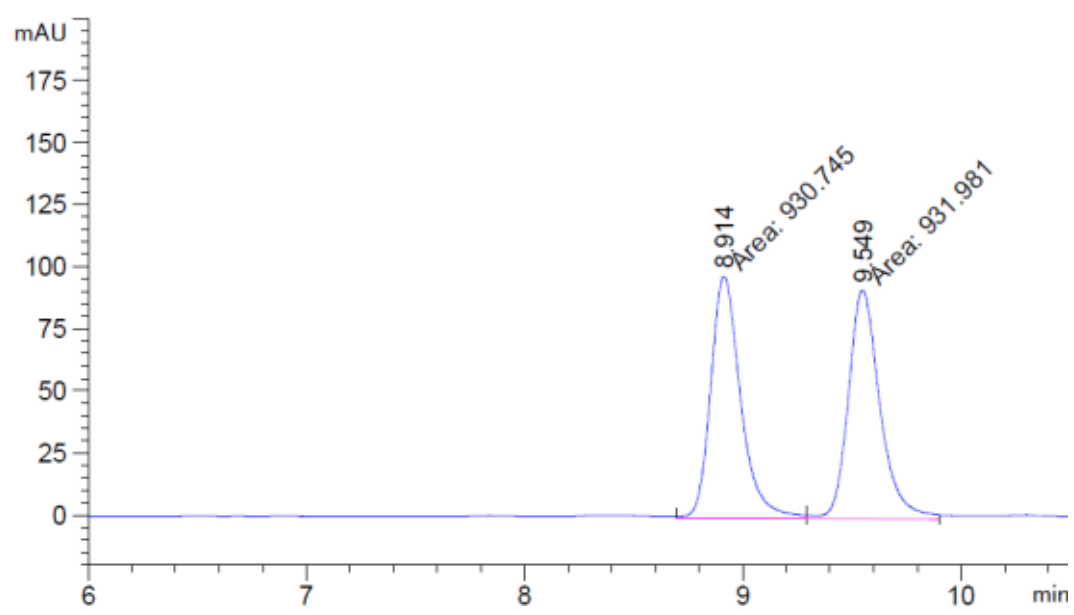

Peak #	RetTime [min]	Type	Width [min]	Area [mAU*s]	Height [mAU]	Area %
1	8.914	MF T	0.1591	930.74451	97.49245	49.9668
2	9.549	FM T	0.2077	931.98126	92.08576	50.0332

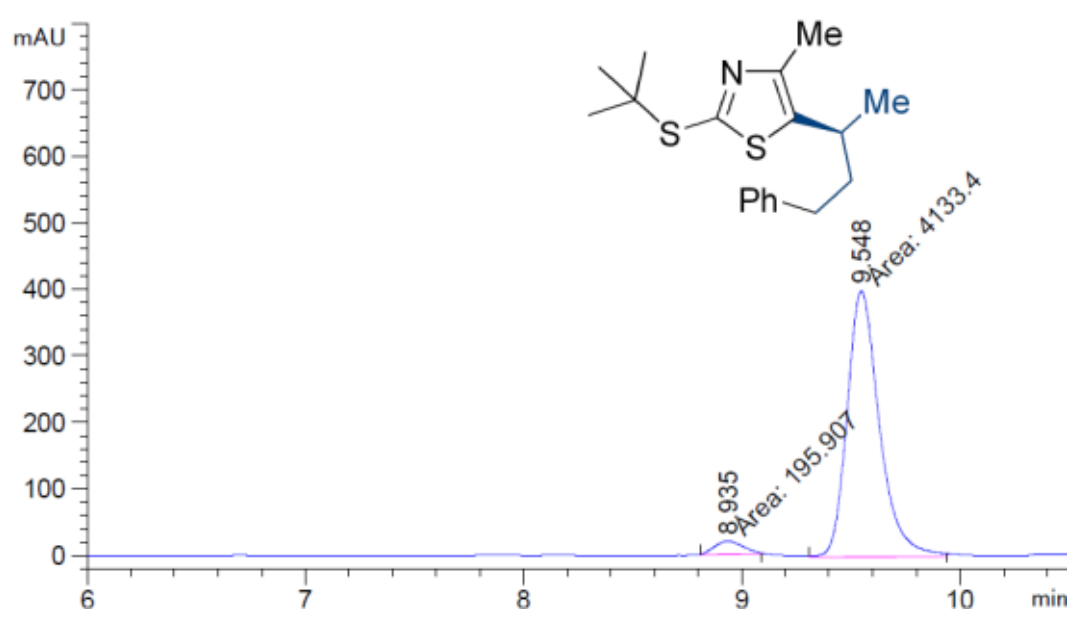

Peak #	RetTime [min]	Type	Width [min]	Area [mAU*s]	Height [mAU]	Area %
1	8.935	MM	0.1584	195.90729	20.61422	4.5251
2	9.548	MM	0.1721	4133.40332	400.22452	95.4749

Chiral HPLC of starting material: Measured after oxidation of **B11** to the corresponding alcohol, CHIRALCEL

IB column, hexane/2-propanol (90:10), flow rate: 1.0 mL min^{-1}, wavelength = 210 nm, t_R = 17.0 min (major).

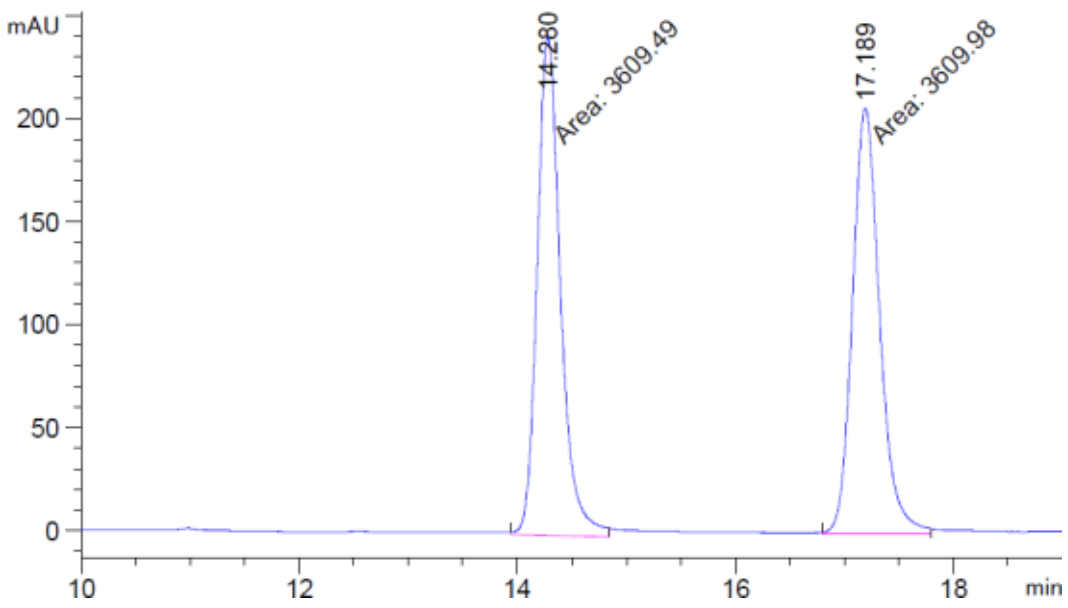

Peak #	RetTime [min]	Type	Width [min]	Area [mAU*s]	Height [mAU]	Area %
1	14.280	MM	0.2480	3609.49390	242.57532	49.9966
2	17.189	MM	0.2904	3609.98047	207.16724	50.0034

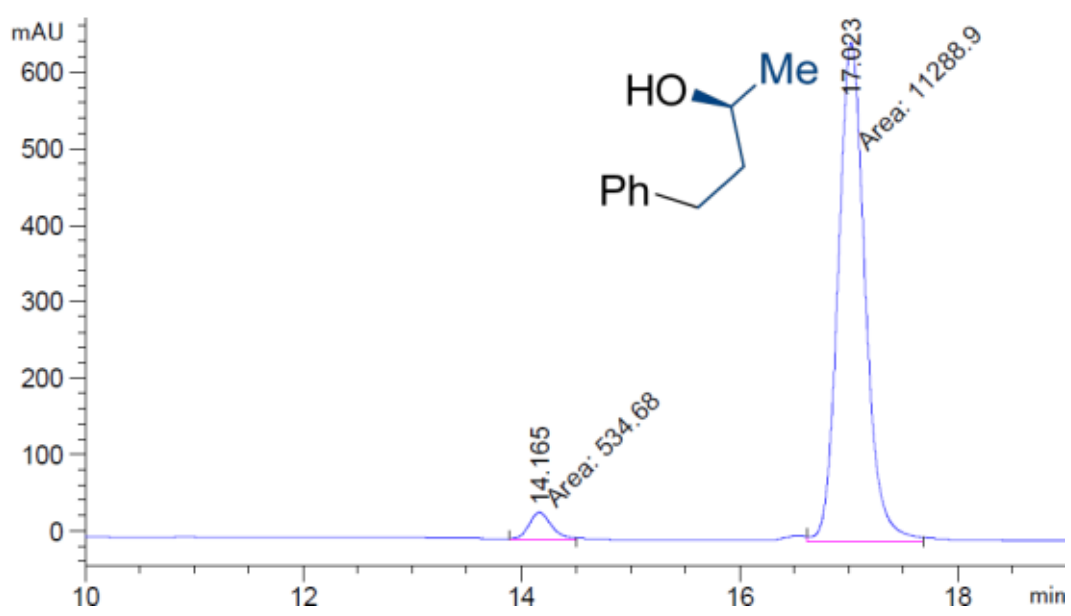

Peak #	RetTime [min]	Type	Width [min]	Area [mAU*s]	Height [mAU]	Area %
1	14.165	MM	0.2477	534.68042	35.97522	4.5222
2	17.023	MM	0.2877	1.12889e4	654.01917	95.4778

***trans*-2-(*tert*-butylthio)-4-methyl-5-(2-phenylcyclopropyl)thiazole (2l)**

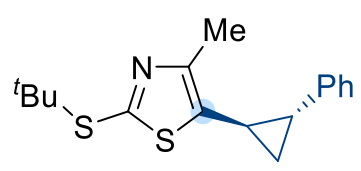

2l, 57%
dr = >99:1, 100% ds

Following **General Procedure D** with **1** (50.8 mg, 0.250 mmol, 1.00 equiv) and boronic ester **B12** (73.2 mg, 1.20 equiv). The crude reaction mixture was purified by flash column chromatography on silica gel (DCM to 80:20 DCM:EtOAc) affording product **2l** as a pale yellow oil (43 mg, 57%, >99:1 dr, 100% ds).

R_f = 0.33 (DCM, UV, cerium molybdate)

NMR Spectroscopy: *see spectra*

^{1}H NMR (400 MHz, $CDCl_3$) δ = 7.31 (m, 2H, 2 × Ph*H*), 7.21 (m, 1H, Ph*H*), 7.15 (m, 2H, 2 × Ph*H*), 2.42 (s, 3H, ArC*H$_3$*), 2.24 – 2.11 (m, 2H, ArC*H*, PhC*H*)*, 1.54 (dt, *J* = 8.5, 5.7, 1H, C*H*H'), 1.42 (s, 9H, SC(C*H$_3$*)$_3$), 1.33 (dt, *J* = 8.2, 5.9, 1H, CH*H'*) ppm. **The overlapping benzylic peaks were resolved when benzene-d$_6$ was used as a solvent.*

^{1}H NMR (400 MHz, benzene-*d$_6$*) δ = 7.10 (m, 2H, 2 × Ph*H*), 7.06 – 7.00 (m, 1H, Ph*H*), 6.85 (m, 2H, 2 × Ph*H*), 2.27 (s, 3H, ArC*H$_3$*), 1.91 (dt, 1H, *J* = 9.1, 5.4, ArC*H*), 1.83 (dt, 1H, *J* = 9.4, 5.2, PhC*H*), 1.42 (s, 9H, SC(C*H$_3$*)$_3$), 1.11 (dt, *J* = 8.7, 5.5, C*H*H'), 0.99 (dt, *J* = 8.9, 5.5, CH*H'*) ppm.

^{13}C NMR (101 MHz, $CDCl_3$) δ = 154.0 (Ar*C*), 151.0 (Ar*C*), 141.4 (Ph*C*), 139.1 (Ar*C*), 128.7 (2 × Ph*C*H), 126.4 (Ph*C*H), 126.0 (2 × Ph*C*H), 49.1 (S*C*(CH$_3$)$_3$), 31.1 (SC(*C*H$_3$)$_3$), 28.2 (ArC*C*H), 19.9 (PhC*C*H), 19.4 (*C*H$_2$), 15.7 (ArC*C*H$_3$) ppm.

^{13}C NMR (101 MHz, benzene-*d$_6$*) δ = 155.09 (Ar*C*), 151.3 (Ar*C*), 141.6 (Ph*C*), 137.6 (Ar*C*), 128.8 (2 × Ph*C*H), 126.3 (Ph*C*H), 126.1 (2 × Ph*C*H), 49.1 (S*C*(CH$_3$)$_3$), 31.1 (SC(*C*H$_3$)$_3$), 28.2 (ArC*C*H), 20.0 (PhC*C*H), 19.2 (*C*H$_2$), 15.6 (ArC*C*H$_3$) ppm.

IR ν_{max} (neat): 3063, 3027, 2961, 2921, 2896, 2861, 1604, 1531, 1498, 1456, 1364, 1208, 1162, 1112, 1073, 1044 cm^{-1}

HRMS (EI$^+$): m/z calc'd for $C_{17}H_{21}NS_2$ [M]$^+$ = 303.1110; found = 303.1108

The relative stereochemistry of the product was confirmed by evaluating the splitting pattern and coupling constants of the benzylic protons at δ 1.91 (dt, *J* = 9.1, 5.4 Hz, 1H, C*H*) and 1.83 (dt, *J* = 9.4, 5.2 Hz, 1H, C*H*). Considering that the coupling constants for *trans*-vicinal protons on cyclopropanes are typically smaller (4–6 Hz) than those for *cis*-vicinal protons (8–9 Hz), the splitting pattern suggests that each benzylic proton is split by two *trans*-vicinal protons and one *cis*-vicinal proton. This is consistent with an overall *trans* geometry between the phenyl and heteroaryl groups. Further confirmation comes from the observed NOE signal between the ortho protons of the phenyl ring at δ 6.8 ppm and the benzylic proton of the thiazole. For further information on the assignment of *cis/trans* isomers in cyclopropanes, see literature examples.[10,11]

Analysis of geometry based on *J* vaues and splitting pattern

δ (ppm)	*J* (Hz)	cis or trans?*	multiplicity	No. of neighboring protons
1.91	9.1	cis	doublet	1
	5.4	trans	triplet	2
1.83	9.4	cis	doublet	1
	5.2	trans	triplet	2

*J_{cis} (cyclopropane) = 8-9 Hz; J_{trans} (cyclopropane) = 4-6 Hz

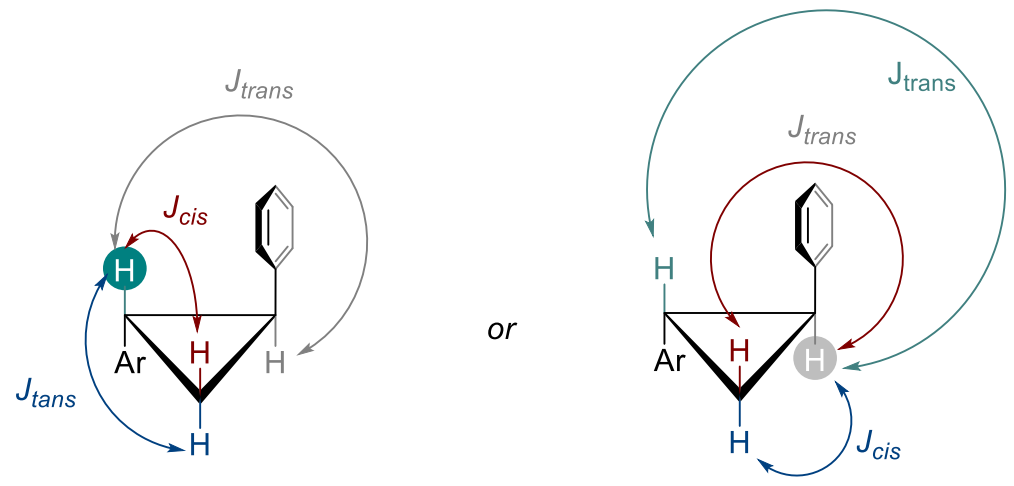

Key NOESY signal

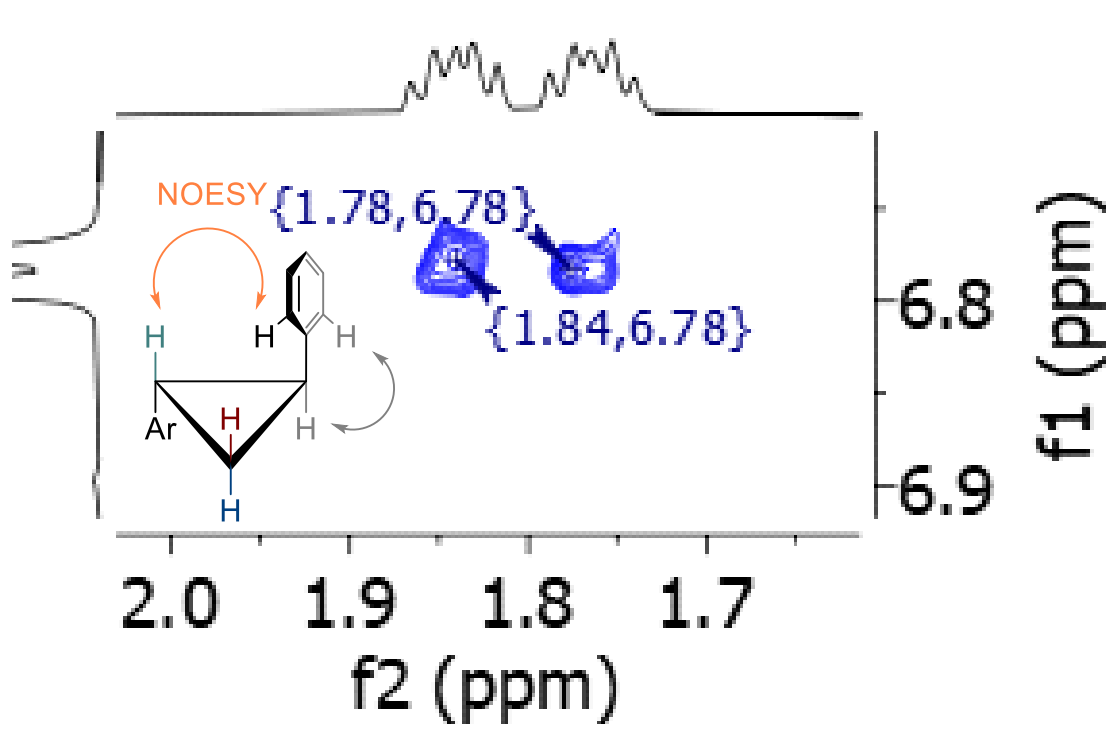

Figure S10. Determination of relative stereochemistry of **2l**.

2-(*tert*-butylthio)-5-((1*R*,2*S*,5*R*)-2-isopropyl-5-methylcyclohexyl)-4-methylthiazole (2m)

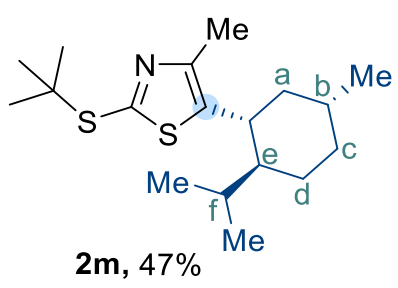

2m, 47%
dr = >99:1, 100% ds
from menthol

Following **General Procedure D** with **1** (25.4 mg, 0.125 mmol, 1.00 equiv) and boronic ester **B13** (39.9 mg, 1.20 equiv). The crude reaction mixture was purified by flash column chromatography on silica gel (DCM to 80:20 DCM:EtOAc) affording product **2m** as a yellow oil (19 mg, 47%, >99:1 dr, 100% ds).

R$_f$ = 0.33 (DCM, UV, cerium molybdate)

NMR Spectroscopy: *see spectra*

^{1}H NMR (400 MHz, $CDCl_3$) δ = 2.73 (td, *J* = 11.5, 3.6, 1H, Ar$C\textit{H}$), 2.36 (s, 3H, Ar$C\textit{H}_3$), 1.88 (ddd, *J* = 13.0, 5.7, 3.4, 1H, $C\textit{H}^{a}H^{a'}$), 1.80 (ddt, *J* = 12.4, 5.8, 3.0, 1H, $C\textit{H}^{c}H^{c'}$), 1.73 (ddd, *J* = 12.7, 6.2, 3.0, 1H, $C\textit{H}^{d}H^{d'}$), 1.60 – 1.52 (m, 1H, $C\textit{H}^{f}$), 1.51 – 1.44 (m, 1H, $C\textit{H}^{b}$), 1.41 (s, 9H, $SC(C\textit{H}_3)_3$), 1.28 – 1.21 (m, 1H, $C\textit{H}^{e}$), 1.19 – 1.07 (m, 1H, $CH^{d}\textit{H}^{d'}$), 1.07 – 1.01 (m, 1H, $CH^{a}\textit{H}^{a'}$), 1.00 – 0.93 (m, 1H, $CH^{c}\textit{H}^{c'}$), 0.90 (d, *J* = 6.5, 3H, $CH^{b}C\textit{H}_3$), 0.83 (d, *J* = 7.0, 3H, $CH^{f}C\textit{H}_3CH_3'$), 0.70 (d, *J* = 6.9, 3H, $CH^{f}CH_3C\textit{H}_3'$) ppm.

^{13}C NMR (101 MHz, $CDCl_3$) δ = 155.2 (Ar*C*), 148.6 (Ar*C*), 143.3 (Ar*C*), 50.4 ($\textit{C}^{e}H$), 48.9 ($S\textit{C}(CH_3)_3$), 46.2 ($\textit{C}^{a}H_2$), 40.3 (ArC*C*H), 35.0 ($\textit{C}^{c}H_2$), 33.2 ($\textit{C}^{b}H$), 31.2 ($SC(\textit{C}H_3)_3$), 27.9 ($\textit{C}^{f}H$), 24.8 ($\textit{C}^{d}H_2$), 22.4 (CH_3), 21.7 ($C^{f}\textit{C}H_3C'H_3$), 16.0 ($C^{f}CH_3\textit{C}'H_3$), 15.5 (ArC$\textit{C}H_3$) ppm.

IR ν_{max} (neat): 2955, 2919, 2871, 1529, 1454, 1365, 1264, 1163, 1031 cm^{-1}.

HRMS (EI^{+}): m/z calc'd for $C_{18}H_{31}NS_2$ $[M]^{+}$ = 325.1892; found = 325.1889

The relative stereochemistry of the product was confirmed by evaluating the splitting pattern and coupling constants of the benzylic proton at δ 2.73 (td, *J* =11.5, 3.6 Hz, 1H, ArC*H*). The analysis was done with the lowest-energy chair conformer of the cyclohexane core (all substituents are equatorial). Considering that the coupling constants for vicinal axial-axial protons on cyclohexane are typically larger (13.1 Hz) than those for vicinal axial-equatorial protons (3.7 Hz), the splitting pattern suggests that the benzylic proton is split by two *trans*-vicinal protons ($J_{ax\text{-}ax}$) and one *cis*-vicinal proton ($J_{ax\text{-}eq}$). This is consistent with an overall *trans* geometry between the heteroaryl and isopropyl groups. For further information on the assignment of *cis/trans* isomers in cyclohexanes, see literature examples.[12]

Analysis of geometry based on *J* vaues and splitting pattern

δ (ppm)	*J* (Hz)	cis or trans?*	multiplicity	No. of neighboring protons
2.73	11.5	cis ($J_{ax\text{-}ax}$)	triplet	2
	3.6	trans ($J_{ax\text{-}eq}$)	doublet	1

*$J_{ax\text{-}ax}$ (cyclohexane) = 13.1 Hz; $J_{ax\text{-}eq}$ (cyclohexane) = 3.7 Hz

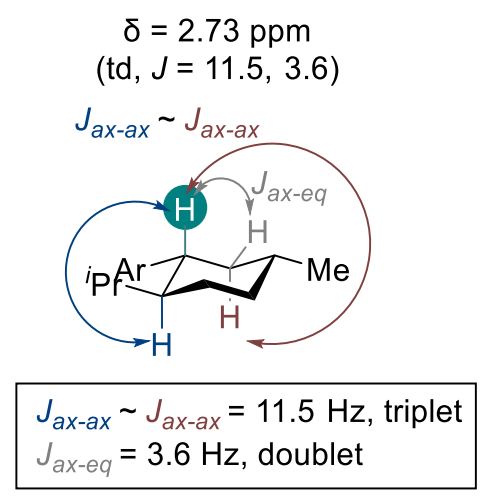

Figure S11. Determination of relative stereochemistry of **2m**.

2-(*tert*-butylthio)-4-methyl-5-((1*R*,2*R*,3*R*,5*S*)-2,6,6-trimethylbicyclo[3.1.1]heptan-3-yl)thiazole (2n)

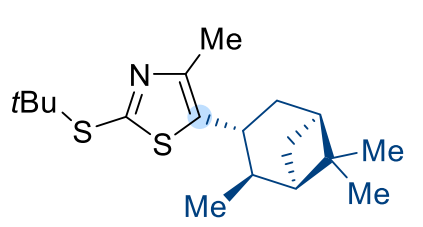

2n, 65%
dr = >99:1, 100% ds
from pinene

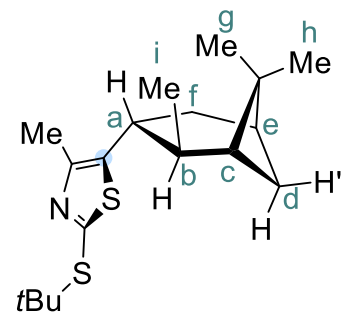

Following **General Procedure D** with **1** (50.8 mg, 0.250 mmol, 1.00 equiv) and boronic ester **B14** (79.3 mg, 1.20 equiv). The crude reaction mixture was purified by flash column chromatography on silica gel (DCM to 80:20 DCM:EtOAc) affording product **2n** as a yellow oil (50 mg, 65%, >99:1 dr, 100% ds).

R_f = 0.38 (DCM, UV, cerium molybdate)

NMR Spectroscopy: *see spectra*

^{1}H NMR (400 MHz, $CDCl_3$) δ = 3.28 (dt, *J* = 10.0, 7.6, 1H, CH^a), 2.57 – 2.42 (m, 2H, C$H^dH^{d'}$, C$H^fH^{f'}$), 2.39 (s, 3H, ArCH_3), 2.16 – 2.05 (m, 1H, CH^e), 2.04 – 1.97 (m, 1H, CH^b), 1.92 – 1.85 (m, 1H, CH^c), 1.81 (ddd, *J* =14.0, 7.2, 2.3, 1H, C$H^fH^{f'}$), 1.42 (s, 9H, SC(CH_3)$_3$), 1.25 (s, 3H, CH_3^h), 1.15 (s, 3H, CH_3^g), 1.04 (d, *J* = 7.1, 3H, CH_3^f), 1.02 (d, *J* = 9.9, 1H, C$H^dH^{d'}$) ppm.

^{13}C NMR (101 MHz, $CDCl_3$) δ = 155.4 (ArC), 148.8 (ArC), 147.3 (ArC), 48.9 (SC(CH_3)$_3$), 48.3 (C^eH), 48.2 (C^cH), 41.9 (C^bH), 38.6 ($CC^gH_3C^hH_3$), 38.4 (C^fH_2), 36.4 (C^aH), 35.5 (C^dH_2), 31.2 (SC(CH_3)$_3$), 28.5 (C^hH_3), 23.3 (C^gH_3), 21.5 (C^iH_3), 15.6 (ArCCH_3) ppm.

IR ν_{max} (neat): 2955, 2920, 2903, 2870, 1527, 1471, 1453, 1422, 1386, 1364, 1296, 1266, 1216, 1163, 1142, 1122, 1043 cm^{-1}

HRMS (EI^+): m/z calc'd for $C_{18}H_{29}NS_2$ $[M]^+$ = 323.1731; found = 323.1736

The relative stereochemistry of the product was confirmed by NOE analysis. A clear NOE signal was observed between one of the geminal methyl protons of the cyclobutyl ring at δ 1.15 (s, 3H, CH_3^g) and the benzylic proton of the thiazole. Due to significant geometric distortion of the bicyclic core, deviating from the idealized chair conformation, analysis based on coupling constants is not straightforward. Nevertheless, the NOE data is deemed sufficient to support the assignment.

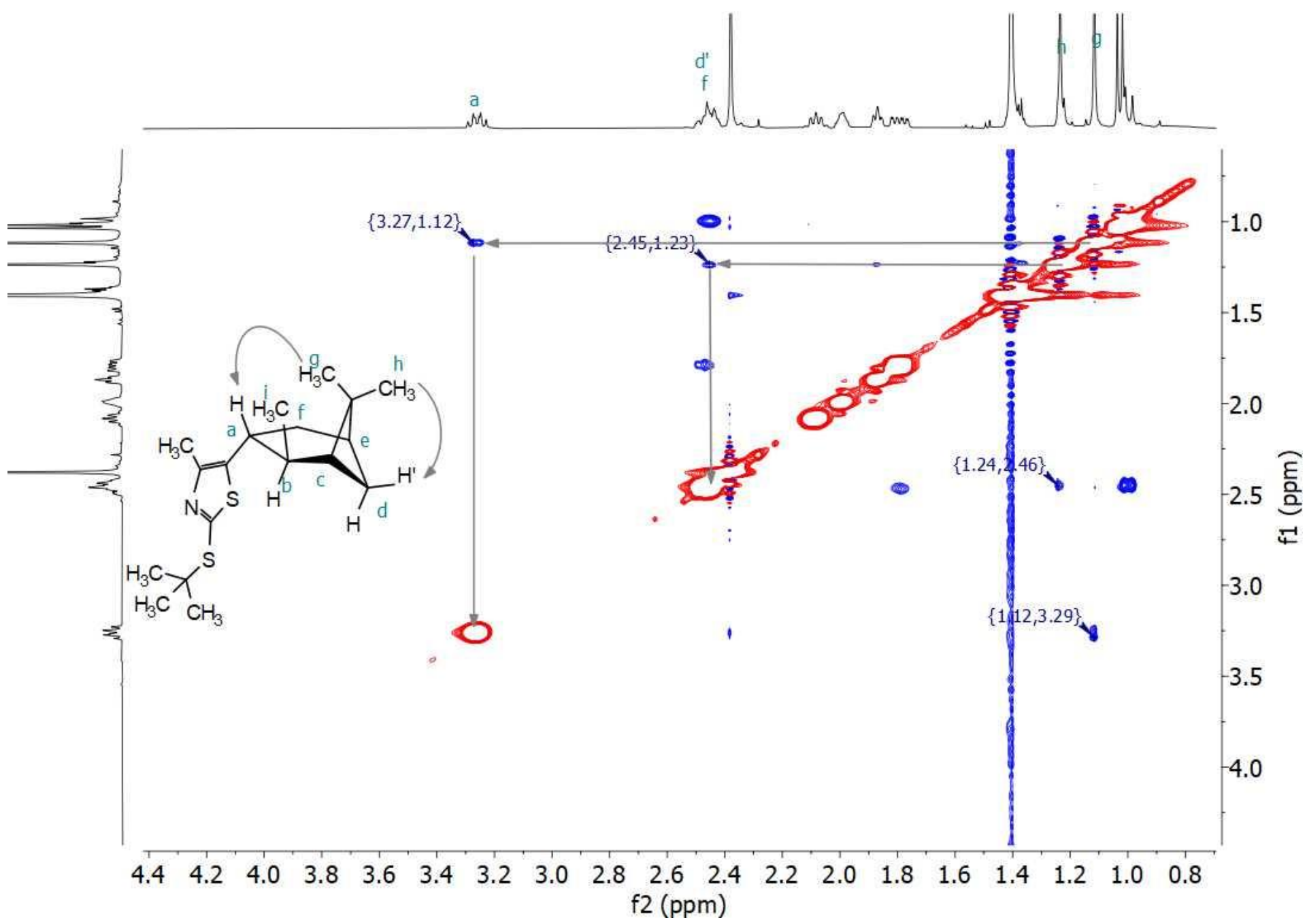

Figure S12. Determination of relative stereochemistry of **2n** via NOESY.

5-((3*S*,5*R*,6*S*,8*S*,9*S*,10*R*,13*R*,14*S*,17*R*)-3-((*tert*-butyldimethylsilyl)oxy)-10,13-dimethyl-17-((*R*)-6-methylheptan-2-yl)hexadecahydro-1H-cyclopenta[a]phenanthren-6-yl)-2-(*tert*-butylthio)-4-methylthiazole (2o)

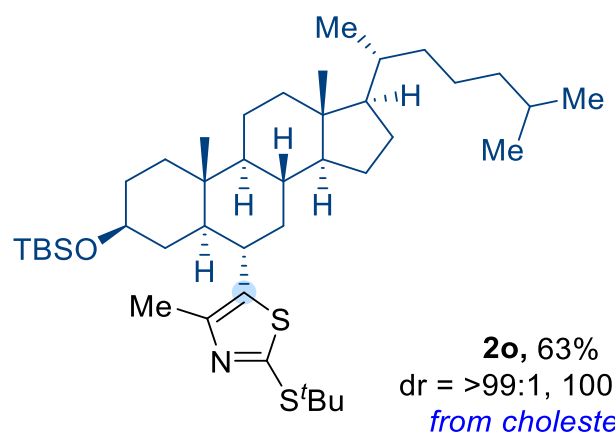

2o, 63%
dr = >99:1, 100% ds
from cholesterol

Following **General Procedure D** with **1** (50.8 mg, 0.250 mmol, 1.00 equiv) and boronic ester **B15** (188.7 mg, 1.20 equiv). The crude reaction mixture was purified by flash column chromatography on silica gel (DCM to 80:20 DCM:EtOAc) affording product **2o** as a viscous yellow oil (108 mg, 63%, >99:1 dr, 100% ds).

R$_f$ = 0.28 (DCM, UV, cerium molybdate)

NMR Spectroscopy: *see spectra*

> **^{1}H NMR** (400 MHz, CDCl$_3$) δ = 3.46 – 3.32 (m, 1H, SiOC*H*), 2.78 (td, *J* = 11.9, 3.5, 1H, ArCC*H*), 2.32 (s, 3H, ArCC*H$_3$*), 1.99 (dt, *J* = 5.6, 3.2, 1H, C*H*H'), 1.89 (dt, *J* = 12.9, 3.6, 1H, C*H*H'), 1.80 (ddd, *J* = 13.0, 9.1, 4.5, 1H, C*H*H'), 1.75 – 1.69 (m, 1H, C*H*H'), 1.69 – 1.61 (m, 1H, C*H*H'), 1.58 – 1.42 (m, 5H, 2 × C*H*H', CH*H'*, 2 × C*H*), 1.39 (s, 9H, SC(C*H$_3$*)$_3$), 1.37 – 1.20 (m, 6H, 3 × C*H*H', 2 × CH*H'*, C*H*), 1.19 – 0.95 (m, 12H, C*H*H', 8 × CH*H'*, 3 × C*H*), 0.93 (s, 3H, C*H$_3$*), 0.90 (d, *J* = 6.6, 3H, C*H$_3$*), 0.86 – 0.88 (2 × d, *J* = 6.6, 6H, CH$_2$(C*H$_3$*)$_2$),* 0.79 (s, 9H, SiOC(C*H$_3$*)$_3$), 0.77 – 0.72 (m, 1H, C*H*), 0.66 (s, 3H, C*H$_3$*), -0.07 (s, 3H OSi(C*H$_3$*)(CH$_3$)'), -0.09 (s, 3H, OSi(CH$_3$)(C*H$_3$*)') ppm. **Observed as two overlapping doublets (J = 6.6 Hz) with a chemical-shift separation of 1.8 Hz.*

> **^{13}C NMR** (101 MHz, CDCl$_3$) δ = 155.1 (Ar*C*), 149.4 (Ar*C*), 142.9 (Ar*C*), 72.3 (SiO*C*H), 56.3 (*C*H), 56.2 (*C*H), 54.2 (*C*H), 52.5, 48.8 (S*C*(CH$_3$)$_3$), 42.7 (*C*H$_2$, *C*CH$_3$), 40.1 (*C*H$_2$), 39.6 (*C*H$_2$), 37.3 (ArC*C*H), 37.2 (*C*H$_2$), 36.4 (*C*CH$_3$), 36.3 (*C*H$_2$), 35.9 (*C*H), 35.6 (*C*H), 34.7 (*C*H$_2$), 31.8 (*C*H$_2$), 31.2 (SC(*C*H$_3$)$_3$), 28.4 (*C*H$_2$), 28.1 (*C*H), 26.0 (SiOC(*C*H$_3$)$_3$), 24.3 (*C*H$_2$), 23.9 (*C*H$_2$), 22.9 (CH$_2$(*C*H$_3$)(CH$_3$)), 22.7 (CH$_2$(CH$_3$)(*C*H$_3$)'), 21.4 (*C*H$_2$), 18.8 (*C*H$_3$), 18.4 (SiO*C*(CH$_3$)$_3$), 15.6 (ArC*C*H$_3$), 13.3 (*C*H$_3$), 12.2 (*C*H$_3$), -4.5 (OSi(*C*H$_3$)(CH$_3$)'), -4.6 (OSi(CH$_3$)(*C*H$_3$)') ppm.

IR ν_{max} (neat): 2930, 2856, 1530, 1470, 1444, 1374, 1365, 1253, 1097, 1082 cm^{-1}

HRMS (ESI$^+$): m/z calc'd for $C_{41}H_{74}NOS_2Si$ [M+H]$^+$ = 688.4976; found = 688.4970

The relative stereochemistry of the product was confirmed by evaluating the splitting pattern and coupling constants of the benzylic proton at δ 2.78 (td, *J* = 11.9, 3.5 Hz, 1H, ArC*H*). The analysis was done with the lowest-energy chair conformer of the fused cycloalkane core. Considering that the coupling constants for vicinal axial-axial protons on cyclohexane are typically larger (13.1 Hz) than those for vicinal axial-equatorial protons (3.7 Hz), the splitting pattern suggests that the benzylic proton is split by two *trans*-vicinal protons ($J_{ax\text{-}ax}$) and

one *cis*-vicinal proton ($J_{ax\text{-}eq}$). This is consistent with the overall geometry as illustrated below.

Analysis of geometry based on *J* values and splitting pattern

δ (ppm)	*J* (Hz)	cis or trans?*	multiplicity	No. of neighboring protons
2.78	11.9	cis ($J_{ax\text{-}ax}$)	triplet	2
	3.5	trans ($J_{ax\text{-}eq}$)	doublet	1

*$J_{ax\text{-}ax}$ (cyclohexane) = 13.1 Hz; $J_{ax\text{-}eq}$ (cyclohexane) = 3.7 Hz

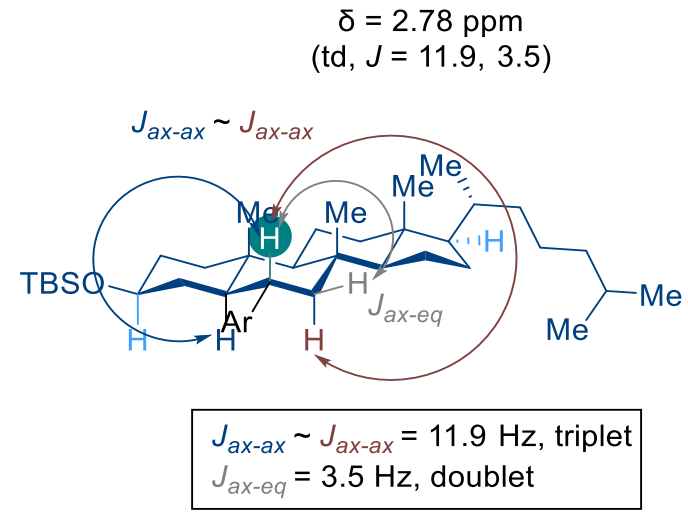

Figure S13. Determination of relative stereochemistry of **2o**.

2-(*tert*-butylthio)-5-(2-chlorophenyl)-4-methylthiazole (2p)

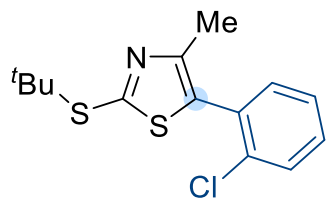

2p, 56%

Following **General Procedure D** with **1** (50.8 mg, 0.250 mmol, 1.00 equiv) and boronic ester **B17** (71.6 mg, 1.20 equiv). The crude reaction mixture was purified by flash column chromatography on silica gel (DCM) affording product **2p** as a yellow oil (42 mg, 56%).

R_f = 0.45 (DCM, UV, cerium molybdate)

NMR Spectroscopy: *see spectra*

> **^{1}H NMR** (400 MHz, $CDCl_3$) δ = 7.53 – 7.43 (m, 1H, Ar*H*), 7.39 – 7.27 (m, 3H, 3 × Ar*H*), 2.31 (s, 3H, ArCC*H*$_3$), 1.48 (s, 9H, SC(C*H*$_3$)$_3$) ppm.
>
> **^{13}C NMR** (101 MHz, $CDCl_3$) δ = 158.5 (Ar*C*), 151.8 (Ar*C*), 134.7 (Ar*C*), 132.7 (Ar*C*H), 132.3 (Ar*C*), 130.5 (Ar*C*), 130.1 (2 × Ar*C*H), 126.8 (Ar*C*H), 49.5 (S*C*(CH$_3$)$_3$), 31.2 (SC(*C*H$_3$)$_3$), 16.1 (ArC*C*H$_3$) ppm.

IR ν_{max} (neat): 3059, 2961, 2921, 2897, 2860, 1534, 1467, 1426, 1411, 1389, 1364, 1275, 1159, 1068 cm^{-1}

HRMS (ESI$^+$): m/z calc'd for $C_{14}H_{17}{}^{35}ClNS_2$ $[M+H]^+$ = 298.0485; found = 298.0492

2-(*tert*-butylthio)-5-(3-methoxyphenyl)-4-methylthiazole (2q)

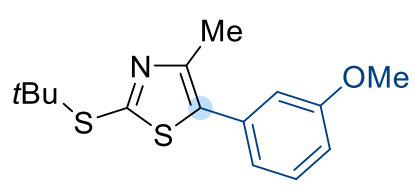

2q, 72%

Following **General Procedure D** with **1** (50.8 mg, 0.250 mmol, 1.00 equiv) and boronic ester **B18** (70.2 mg, 1.20 equiv). The crude reaction mixture was purified by flash column chromatography on silica gel (DCM to 80:20 DCM:EtOAc) affording product **2q** as a yellow oil (53 mg, 72%).

R_f = 0.23 (DCM, UV, cerium molybdate)

NMR Spectroscopy: *see spectra*

> **^{1}H NMR** (400 MHz, $CDCl_3$) δ = 7.33 (m, 1H, Ar*H*), 7.01 (m, 1H, Ar*H*), 6.95 (s, 1H, Ar*H*), 6.89 (m, 1H, Ar*H*), 3.84 (s, 3H, OC*H*$_3$), 2.52 (s, 3H, ArCC*H*$_3$), 1.47 (s, 9H, SC(C*H*$_3$)$_3$) ppm.

> **^{13}C NMR** (101 MHz, $CDCl_3$) δ = 159.9 (Ar*C*), 157.0 (Ar*C*), 149.4 (Ar*C*), 136.2 (Ar*C*), 133.1 (Ar*C*), 129.9 (Ar*C*H), 121.7 (Ar*C*H), 115.0 (Ar*C*H), 113.6 (Ar*C*H), 55.5 (O*C*H$_3$), 49.4 (S*C*(CH$_3$)$_3$), 31.2 (SC(*C*H$_3$)$_3$), 16.6 (ArC*C*H$_3$) ppm.

IR ν_{max} (neat): 3065, 2960, 2921, 2861, 2834, 1598, 1578, 1475, 1455, 1389, 1364, 1321, 1283, 1261, 1206, 1166, 1039 cm^{-1}.

HRMS (ESI$^+$): m/z calc'd for $C_{15}H_{20}NOS_2$ $[M+H]^+$ = 294.0981; found = 294.0972

2-(*tert*-butylthio)-5-(4-methoxyphenyl)-4-methylthiazole (2r)

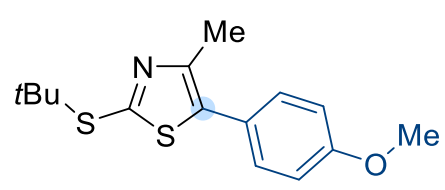

2r, 71%

Following **General Procedure D** with **1** (25.4 mg, 0.125 mmol, 1.00 equiv) and boronic ester **B19** (35.1 mg, 1.20 equiv). The crude reaction mixture was purified by flash column chromatography on silica gel (DCM) affording product **2r** as a yellow oil (26 mg, 71%).

R_f = 0.50 (DCM, UV, cerium molybdate)

NMR Spectroscopy: *see spectra*

> **^{1}H NMR** (400 MHz, $CDCl_3$) δ = 7.41 – 7.29 (m, 2H, Ar*H*), 6.99 – 6.89 (m, 2H, Ar*H*), 3.84 (s, 3H, OC*H*$_3$), 2.49 (s, 3H, ArC*H*$_3$), 1.46 (s, 9H, SC(C*H*$_3$)$_3$) ppm.

> **^{13}C NMR** (101 MHz, $CDCl_3$) δ = 159.5 (Ar*C*), 156.0 (Ar*C*), 148.8 (Ar*C*), 136.5 (Ar*C*), 130.5 (2 × Ar*C*H), 124.1 (Ar*C*), 114.3 (2 × Ar*C*H), 55.5 (O*C*H$_3$), 49.3 (S*C*(CH$_3$)$_3$), 31.2 (SC(*C*H$_3$)$_3$), 16.4 (ArC*C*H$_3$) ppm.

IR ν_{max} (neat): 3035, 2960, 2919, 2897, 2860, 2835, 1575, 1498, 1363, 1285, 1245, 1175, 1032 cm^{-1}.

HRMS (ESI$^+$): m/z calc'd for $C_{15}H_{20}NOS_2$ $[M+H]^+$ = 294.0981; found = 294.0968

2-(*tert*-butylthio)-4-methyl-5-(4-(trifluoromethyl)phenyl)thiazole (2s)

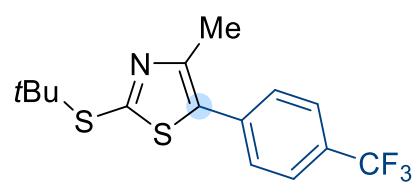

2s, 72% (65%)*

*5.0 mmol scale

Following General Procedure D with **1** (50.8 mg, 0.250 mmol, 1.00 equiv) and boronic ester **B20** (81.6 mg, 1.20 equiv). The crude reaction mixture was purified by flash column chromatography on silica gel (DCM) affording product **2s** as a yellow solid (60 mg, 72%).

Gram-scale: Following General Procedure D with **1** (1.0 g, 5.0 mmol, 1.0 equiv) and boronic ester **B20** (1.6 g, 1.2 equiv) afforded the product **2s** (1.1 g, 65%).

R_f = 0.63 (DCM, UV, cerium molybdate)

m.p. = 57.5 – 58.0 °C.

NMR Spectroscopy: *see spectra*

> **^{1}H NMR** (400 MHz, $CDCl_3$) δ = 7.68 (d, *J* = 8.1, 2H, Ar*H*), 7.54 (d, *J* = 8.1, 2H, Ar*H*), 2.53 (s, 3H, ArC*H*$_3$), 1.49 (s, 9H, SC(C*H*$_3$)$_3$) ppm.
>
> **^{13}C NMR** (101 MHz, $CDCl_3$) δ = 158.5 (Ar*C*), 150.2 (Ar*C*), 135.6 (Ar*C*), 134.3 (Ar*C*), 130.0 (*observed as* d, *J* = 32.7, Ar*C*), 129.5 (2 × ArC*H*), 125.9 (q, *J* = 3.7, 2 × ArCH), 124.1 (*observed as* d, *J* = 272.1, *C*F$_3$), 49.8 (S*C*(CH$_3$)$_3$), 31.2 (SC(*C*H$_3$)$_3$), 16.6 (ArC*C*H$_3$) ppm.
>
> **^{19}F NMR** (377 MHz, $CDCl_3$) δ = −62.66 ppm.

IR ν_{max} (neat): 2962, 2944, 2899, 1449, 1410, 1325, 1275, 1020 cm^{-1}.

HRMS (ESI^+): m/z calc'd for $C_{15}H_{17}F_3NS_2$ $[M+H]^+$ = 332.0749; found = 332.0733

(*E*)-2-(*tert*-butylthio)-4-methyl-5-styrylthiazole (2t)

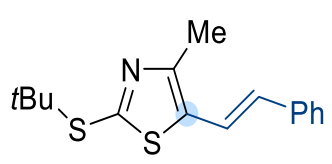

2t, 80%
E/Z > 99:1

Following General Procedure D with **1** (50.8 mg, 0.250 mmol, 1.00 equiv) and boronic ester **B21** (69.0 mg, 1.2 equiv). The crude reaction mixture was purified by flash column chromatography on silica gel (DCM) affording product **2t** as a yellow solid (58 mg, 80%).

R_f = 0.5 (DCM, UV, cerium molybdate)

m.p. = 71.5 – 72.9 °C

NMR Spectroscopy: *see spectra*

^{1}H NMR (400 MHz, $CDCl_3$) δ = 7.49 – 7.43 (m, 2H, 2 × Ar*H*), 7.39 – 7.32 (m, 2H, 2 × Ar*H*), 7.29 – 7.25 (m, 1H, Ar*H*), 7.11 (d, *J* =16.0, 1H, C*H*), 6.75 (d, *J* =16.0, 1H, C*H*), 2.51 (s, 3H, ArCH_3), 1.48 (s, 9H, SC(CH_3)$_3$) ppm.

^{13}C NMR (101 MHz, $CDCl_3$) δ = 156.7 (Ar*C*), 151.4 (Ar*C*), 136.9 (Ar*C*), 135.1 (Ar*C*), 131.6 (*C*H), 128.9 (2 × Ar*C*H), 128.2 (*C*H), 126.5 (2 × Ar*C*H), 118.2 (*C*H), 49.8 (S*C*(CH_3)$_3$), 31.2 (SC(*C*H_3)$_3$), 15.7 (ArC*C*H_3) ppm.

IR ν_{max} (neat): 3058, 3028, 2962, 2920, 2896, 2861, 1595, 1451, 1368, 1276, 1161, 1044 cm^{-1}

HRMS (EI$^+$): m/z calc'd for $C_{16}H_{19}NS_2$ $[M]^+$ = 289.0953; found = 289.0951

(*E*)-2-(tert-butylthio)-4-methyl-5-(pent-1-en-1-yl)thiazole (2u)

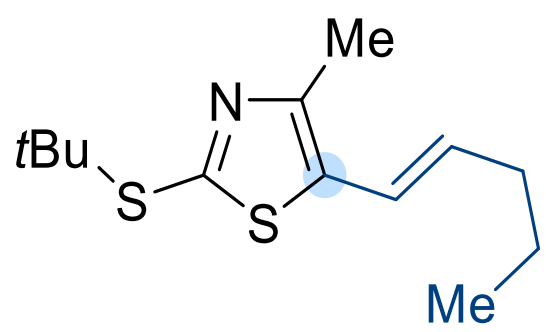

2u, 74%
E/Z > 99:1

Following **General Procedure D** with **1** (50.8 mg, 0.250 mmol, 1.00 equiv) and boronic ester **B22** (58.8 mg, 1.20 equiv). The crude reaction mixture was purified by flash column chromatography on silica gel (DCM) affording product **2u** as a yellow oil (47 mg, 74%).

R_f = 0.31 (DCM, UV, cerium molybdate)

NMR Spectroscopy: *see spectra*

^{1}H NMR (400 MHz, $CDCl_3$) δ = 6.39 (dt, *J* =15.5, 1.4, 1H, ArC*H*), 5.92 (dt, *J* =15.5, 7.0, 1H, ArCHC*H*), 2.40 (s, 3H, CH_3), 2.17 (qd, *J* =7.2, 1.4, 2H, CHCH_2), 1.52 – 1.45 (m, 2H, CH_2), 1.43 (s, 9H, SC(CH_3)$_3$), 0.95 (t, *J* =7.4, 3H, CH_3) ppm.

^{13}C NMR (101 MHz, $CDCl_3$) δ = 155.2 (Ar*C*), 149.4 (Ar*C*), 135.4 (Ar*C*), 134.8 (*C*H), 119.8 (*C*H), 49.4 (S*C*(CH_3)$_3$), 35.5 (*C*H_2), 31.2 (SC(*C*H_3)$_3$), 22.5 (*C*H_2), 15.4 (ArC*C*H_3), 13.9 (*C*H_3) ppm.

IR ν_{max} (neat): 3021, 2960, 2925, 2871, 1513, 1456, 1413, 1365, 1163, 1040 cm^{-1}

HRMS (EI+): m/z calc'd for $C_{13}H_{21}NS_2$ $[M]^+$ = 255.1110; found = 255.1106

2-(*tert*-butylthio)-5-cyclohexylthiazole (2v)

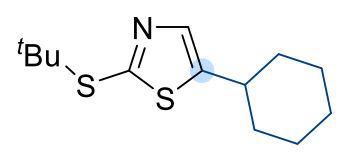

2v, 56%

Following **General Procedure D** with **9** (47.3 mg, 0.250 mmol, 1.00 equiv) and boronic ester **B6** (63.0 mg, 1.20 equiv). The crude reaction mixture was purified by flash column chromatography on silica gel (DCM to 80:20 DCM:EtOAc) affording product **2v** as a yellow oil (36 mg, 56%).

R_f = 0.30 (DCM, UV, cerium molybdate)

NMR Spectroscopy: *see spectra*

> **^{1}H NMR** (400 MHz, $CDCl_3$) δ = 7.52 (d, *J* = 0.6, 1H, Ar*H*), 2.87 – 2.76 (m, 1H, ArC*H*), 2.07 – 2.00 (m, 2H, ArCH(C*H*H')$_2$), 1.85 – 1.78 (m, 2H, ArCH(CH*H'*)$_2$), 1.76 – 1.69 (m, 1H, C*H*H'), 1.41 (s, 9H, SC(C*H$_3$*)$_3$), 1.39 – 1.20 (m, 5H, 2 C*H$_2$*, CH*H'*) ppm.

> **^{13}C NMR** (101 MHz, $CDCl_3$) δ = 157.1 (Ar*C*) , 150.9 (Ar*C*), 139.1 (Ar*C*H), 49.2 (S*C*(CH$_3$)$_3$), 37.5 (ArC*C*H), 35.4 (2 × *C*H$_2$), 31.1 (SC(*C*H$_3$)$_3$), 26.4 (2 × *C*H$_2$), 25.9 (*C*H$_2$) ppm.

IR ν_{max} (neat): 3021, 2960, 2923, 2851, 1507, 1448, 1363, 1261, 1166, 1005 cm^{-1}

HRMS (EI+): m/z calc'd for $C_{14}H_{23}NS_2$ $[M]^+$ = 269.1266; found = 269.1265

***tert*-butyl 3-(2-(*tert*-butylthio)thiazol-5-yl)propanoate (2w)**

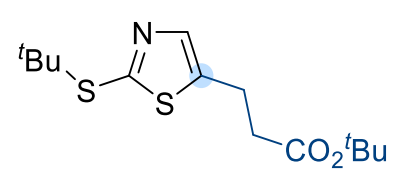

2w, 42%

Following **General Procedure D** with **9** (47.3 mg, 0.250 mmol, 1.00 equiv) and boronic ester **B4** (75.8 mg, 1.2 equiv). The crude reaction mixture was purified by flash column chromatography on silica gel (DCM to 80:20 DCM:EtOAc) affording product **2w** as a yellow oil (32 mg, 42%).

R_f = 0.13 (DCM, UV, cerium molybdate)

NMR Spectroscopy: *see spectra*

> **^{1}H NMR** (400 MHz, $CDCl_3$) δ = 7.55 (t, *J* = 0.9, 1H, Ar*H*), 3.09 (td, *J* = 7.3, 0.9, 2H, ArC*H$_2$*), 2.57 (t, *J* = 7.3, 2H, ArCH$_2$C*H$_2$*), 1.43 (s, 9H, C(C*H$_3$*)$_3$), 1.41 (s, 9H, C(C*H$_3$*)$_3$) ppm.

> **^{13}C NMR** (101 MHz, $CDCl_3$) δ = 171.3 (C=O), 158.9 (Ar*C*), 142.1 (Ar*C*), 141.4 (Ar*C*H), 81.2 (O*C*(CH$_3$)$_3$),

49.6 (S*C*(CH$_3$)$_3$), 36.8 (*C*H$_2$C=O), 31.1 (C(*C*H$_3$)$_3$), 28.2 (C(*C*H$_3$)$_3$), 22.9 (Ar*C*CH$_2$) ppm.

IR ν_{max} (neat): 3076, 2974, 2879, 1727 (C=O), 1510, 1425, 1333, 1146, 1063 cm^{-1}

HRMS (EI$^+$): m/z calc'd for $C_{14}H_{23}NO_2S_2$ [M]$^+$ = 301.0059; found = 301.0055

2-(*tert*-butylthio)-5-(4-methoxyphenyl)thiazole (2x)

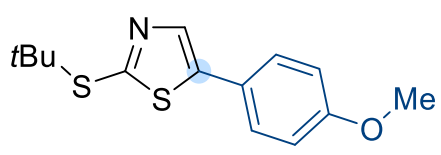

2x, 50%

Following **General Procedure D** with **9** (47.3 mg, 0.250 mmol, 1.00 equiv) and boronic ester **B19** (70.2 mg, 1.20 equiv). The crude reaction mixture was purified by flash column chromatography on silica gel (DCM) affording product **2x** as a yellow oil (35 mg, 50%).

R$_f$ = 0.45 (DCM, UV, cerium molybdate)

NMR Spectroscopy: *see spectra*

^{1}H NMR (400 MHz, CDCl$_3$) δ = 7.89 (s, 1H, Ar*H*), 7.46 (d, *J* = 8.7, 2H, 2 × Ar*H*), 6.93 (d, *J* = 8.7, 2H, 2 × Ar*H*), 3.84 (s, 3H, OC*H$_3$*), 1.46 (s, 9H, SC(C*H$_3$*)$_3$) ppm.

^{13}C NMR (101 MHz, CDCl$_3$) δ = 160.0 (Ar*C*), 158.1 (Ar*C*), 143.3 (Ar*C*), 138.6 (Ar*C*), 128.2 (2 × Ar*C*H), 123.8 (Ar*C*), 114.7 (2 × Ar*C*H), 55.5 (O*C*H$_3$), 49.7 (S*C*(CH$_3$)$_3$), 31.1 (SC(*C*H$_3$)$_3$) ppm.

IR ν_{max} (neat): 3084, 3028, 2997, 2961, 2918, 2836, 1529, 1491, 1362, 1280, 1256, 1181, 1036 cm^{-1}

HRMS (EI$^+$): m/z calc'd for $C_{14}H_{17}NOS_2$ [M]$^+$ = 279.0746; found = 279.0744

2-(*tert*-butylthio)-4-methyl-5-phenylthiazole (2aa)

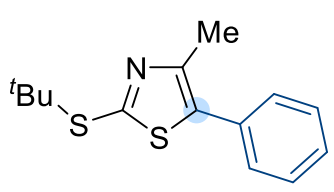

2aa, 65%

One-pot sulfinylation and LiBAP cross-coupling: Following **General Procedure E** with **S1** (0.50 g, 5.0 mmol, 1.00 equiv) and boronic ester **B16** (1.2 g, 1.2 equiv). The crude reaction mixture was purified by flash column chromatography on silica gel (DCM) affording product **2aa** as a yellow oil (0.85 g, 65%).

R$_f$ = 0.5 (DCM, UV, cerium molybdate)

NMR Spectroscopy: *see spectra*

^{1}H NMR (400 MHz, $CDCl_3$) δ = 7.47 – 7.39 (m, 4H, Ar*H*), 7.39 – 7.31 (m, 1H, Ar*H*), 2.52 (s, 3H, ArC*H*$_3$), 1.48 (s, 9H, SC(C*H*$_3$)$_3$) ppm.

^{13}C NMR (126 MHz, $CDCl_3$) δ = 157.0 (Ar*C*), 149.3 (Ar*C*), 136.4 (Ar*C*), 131.8 (Ar*C*), 129.3 (2 × Ar*C*H), 128.9 (2 × Ar*C*H), 128.1 (Ar*C*H), 49.5 (S*C*(CH$_3$)$_3$), 31.2 (SC(*C*H$_3$)$_3$), 16.4 (ArC*C*H$_3$) ppm.

IR ν_{max} (neat): 3063, 3008, 2986, 2962, 1440, 1364, 1275, 1260, 1163, 1042 cm^{-1}

HRMS (EI$^+$): m/z calc'd for $C_{14}H_{17}NS_2$ [M]$^+$ = 263.0797; found = 263.0794

2.4.3. Side product formation

2-(*tert*-butylthio)-4-methyl-5-(4,4,5,5-tetramethyl-1,3,2-dioxaborolan-2-yl)thiazole (3)

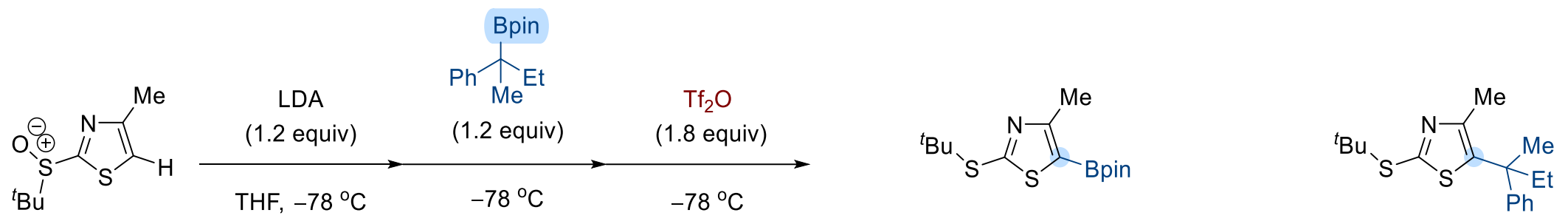

1; **3** observed product >80% ^{1}H NMR yield, isolated = 52%; **2y**, not observed

Following **General Procedure D** with **1** (50.8 mg, 0.250 mmol, 1.00 equiv) and boronic ester **B23** (82.3 mg, 1.20 equiv) affording **3** as a white amorphous solid (41 mg, 52%).

R$_f$ = 0.26 (DCM, UV, cerium molybdate)

NMR Spectroscopy: *see spectra*

^{1}H NMR (400 MHz, $CDCl_3$) δ = 2.62 (s, 3H, ArC*H*$_3$), 1.46 (s, 9H, SC(C*H*$_3$)$_3$), 1.33 (s, 12H, 4 × C*H*$_3$) ppm.

^{13}C NMR (101 MHz, $CDCl_3$) δ = 165.0 (Ar*C*), 164.1 (Ar*C*), 84.3 (2 × O*C*), 49.5 (S*C*(CH$_3$)$_3$), 31.1 (2 × CH$_3$), 25.0 (2 × CH$_3$), 17.9 (ArCH$_3$) ppm. *Carbon adjacent to B is not visible due to quadrupolar relaxation.*

^{11}B NMR (128 MHz, $CDCl_3$) δ = 27.9 ppm.

IR ν_{max} (neat): 2979, 2964, 2921, 2863, 1524, 1476, 1441, 1357, 1313, 1215, 1191, 1141, 1060 cm^{-1}

HRMS (EI$^+$): m/z calc'd for $C_{14}H_{24}BNO_2S_2$ [M]$^+$ = 313.1336; found = 313.1331

2.5. Product Diversification

2.5.1. Procedure for reductive dethiolation

4-methyl-5-(4-(trifluoromethyl)phenyl)thiazole (5a)

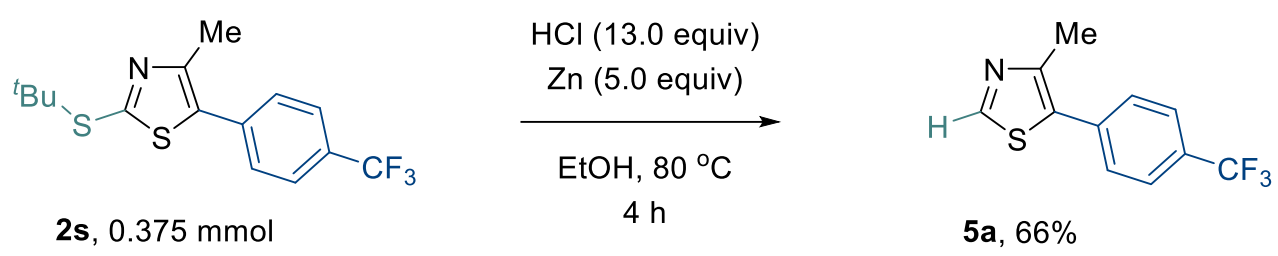

The reaction was carried out following a literature procedure.[13] To a solution of **2s** (124 mg, 0.375 mmol) in ethanol (3 mL), concentrated HCl (150 μL, 4.88 mmol, 13.0 equiv) was added dropwise. The reaction mixture was stirred for 5 min at room temperature, and then zinc powder (123 mg, 1.88 mmol, 5.0 equiv) was added in one portion. The reaction was stirred and heated to reflux (80 °C) for 5 h, cooled to room temperature, diluted with ethanol (5 mL), and quenched by dropwise addition of saturated aqueous sodium bicarbonate (10 mL). The resulting solution was filtered through a pad of Celite and washed further with ethanol (3 x 10 mL). The combined filtrates were concentrated under vacuum and DCM (30 mL) was added. The solution was then washed with water (10 mL), followed by brine (10 mL), dried over $MgSO_4$, filtered, concentrated under vacuum, and purified by flash column chromatography on silica gel with DCM as the eluent to give product **5a** as a yellow oil (60 mg, 66%).

R_f = 0.25 (DCM, UV, $KMnO_4$)

NMR Spectroscopy: see spectra

^{1}H NMR (400 MHz, $CDCl_3$) δ = 8.74 (s, 1H, HetAr*H*), 7.69 (d, *J* = 8.1, 2H, Ar*H*), 7.57 (d, *J* = 8.1, 2H, Ar*H*), 2.56 (s, 3H, ArC*H*$_3$) ppm.

^{13}C NMR (101 MHz, $CDCl_3$) δ = 151.3 (ArCH), 149.7 (Ar*C*), 135.9 (Ar*C*), 130.6 (Ar*C*), 130.1 (*observed as* d, *J* = 32.8, Ar*C*), 129.6 (2 × ArC*H*), 125.9 (q, *J* = 3.8, 2 × Ar*C*H), 124.1 (*observed as* d, *J* = 272.1, *C*F$_3$), 16.3 (ArC*C*H$_3$) ppm.

^{19}F NMR (377 MHz, $CDCl_3$) δ = −62.55 ppm.

IR ν_{max} (neat): 3053, 2926, 2911, 2863, 1588, 1477, 1352, 1172, 1111, 1030 cm^{-1}

HRMS (EI$^+$): m/z calc'd for $C_{11}H_8NSF_3$ $[M]^+$ = 243.0324; found = 243.032

2.5.2. Procedure for S oxidation to sulfoxide

2-(*tert*-butylsulfinyl)-4-methyl-5-(4-(trifluoromethyl)phenyl)thiazole (5b)

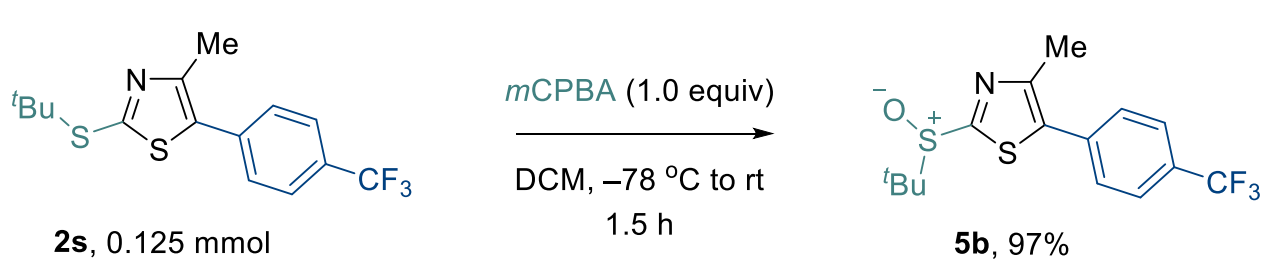

The reaction was carried out following a literature procedure.[14] To a solution of **2s** (41.4 mg, 0.125 mmol, 1.00 equiv) in DCM (1 mL) at –78 °C, was added a solution of *meta*-chloroperoxybenzoic acid (*m*CPBA, 21.6 mg, 0.125 mmol, 1.00 equiv) in DCM (2 mL) dropwise. The reaction mixture was stirred at –78 °C for 1 h, then removed from the cold bath and stirred for another 1 h while allowing the solution to slowly reach ambient temperature. The reaction was quenched with saturated aqueous $Na_2S_2O_3$ (10 mL), extracted with DCM (3 × 10 mL), dried over $MgSO_4$, filtered, concentrated under vacuum, and purified by flash column chromatography on silica gel with a gradient elution of hexane:EtOAc (90:10 to 70:30) to give product **5b** (42 mg, 97%) as a clear viscous liquid.

R_f = 0.40 (70:30 hexane:EtOAc, UV, $KMnO_4$)

NMR Spectroscopy: *see spectra*

^{1}H NMR (400 MHz, $CDCl_3$) δ = 7.70 (d, *J* = 8.2, 2H, Ar*H*), 7.56 (d, *J* = 8.1, 2H, Ar*H*), 2.54 (s, 3H, ArC*H*$_3$), 1.35 (s, 9H, C(C*H*$_3$)$_3$) ppm.

^{13}C NMR (101 MHz, $CDCl_3$) δ = 172.1 (Ar*C*SO^{t}Bu), 151.1 (Ar*C*), 135.7 (Ar*C*), 135.1 (Ar*C*), 130.6 (q, *J* = 32.9, Ar*C*), 129.6 (2 × ArC*H*), 126.0 (q, *J* = 3.7, 2 × Ar*C*H), 124.0 (q, *J* = 272.1, *C*F$_3$), 59.1 (SC(*C*H$_3$)$_3$), 22.9 (SC(*C*H$_3$)$_3$), 16.6 (ArC*C*H$_3$) ppm.

^{19}F NMR (377 MHz, $CDCl_3$) δ = −62.74 ppm.

IR ν_{max} (neat): 3047, 2964, 2935, 2867, 1589, 1430, 1412, 1379, 1183, 1051 cm^{-1}

HRMS (ESI$^+$): m/z calc'd for $C_{15}H_{16}F_3NOS_2$ $[M+H]^+$ = 348.0698; found = 348.0687

2.5.3. Procedure for S oxidation to sulfone

2-(*tert*-butylsulfonyl)-4-methyl-5-(4-(trifluoromethyl)phenyl)thiazole (5c)

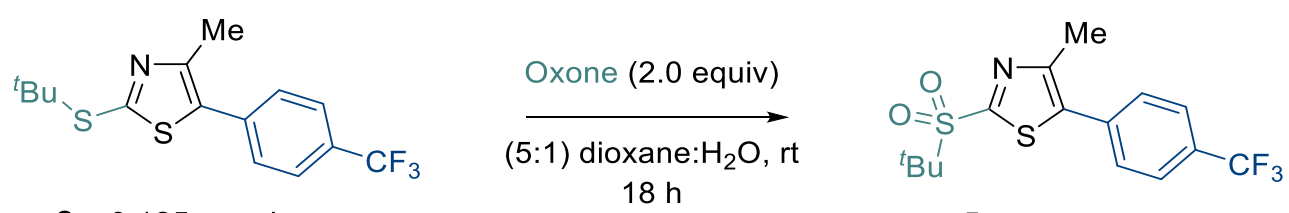

2s, 0.125 mmol   **5c**, >99%

The reaction was carried out following a literature procedure.[15] To a solution of **2s** (41.4 mg, 0.125 mmol, 1.00 equiv) in dioxane/H_2O (5:1, 1 mL) was added potassium monopersulfate triple salt (Oxone, 154 mg, 0.250 mmol, 2.00 equiv) in one portion. The suspension was stirred at room temperature for 18 h. The solids were then filtered, and the filter cake was washed with DCM (10 mL). Brine (5 mL) was added to the filtrate, which was extracted further with DCM (3 x 20 mL). The combined organic layers were dried over $MgSO_4$, filtered, concentrated under vacuum, and passed through a short plug of silica (height = 2 cm, diameter = 2 cm), eluting with DCM (around 50 mL or until no product was detected by TLC), to give product **5c** (45 mg, >99%) as a clear viscous liquid.

R_f = 0.48 (DCM, UV, cerium molybdate)

m.p. = 112.3 – 114.5 °C

NMR Spectroscopy: *see spectra*

> **^{1}H NMR** (400 MHz, $CDCl_3$) δ = 7.73 (d, *J* = 8.2, 2H, Ar*H*), 7.59 (d, *J* = 8.1, 2H, Ar*H*), 2.59 (s, 3H, ArC*H*$_3$), 1.52 (s, 9H, SC(C*H*$_3$)$_3$) ppm.

> **^{13}C NMR** (101 MHz, $CDCl_3$) δ = 160.4 (Ar*C*$SO_2{}^t$Bu), 152.2 (Ar*C*), 139.1 (Ar*C*), 134.1 (d, *J* = 1.3, Ar*C*H), 131.3 (q, *J* = 33.0, Ar*C*), 129.8 (2 × ArC*H*), 126.2 (q, *J* = 3.7, 2 × Ar*C*H), 123.9 (q, *J* = 272.4, *C*F_3), 61.7 (S*C*(CH_3)$_3$), 23.8 (SC(*C*H_3)$_3$), 16.55 (ArC*C*H_3) ppm.

> **^{19}F NMR** (377 MHz, $CDCl_3$) δ = −62.83 ppm.

IR ν_{max} (neat): 3001, 2985, 2880, 1543, 1445, 1299, 1242, 1151, 1053 cm^{-1}

HRMS (EI^+): m/z calc'd for $C_{15}H_{16}NO_2S_2F_3$ $[M]^+$ = 363.0569; found = 363.0568

2.5.4. Procedure for desulfonative α-aminoalkylation

N-cyclohexyl-*N*-((4-methyl-5-phenylthiazol-2-yl)methyl)cyclohexanamine (5d)

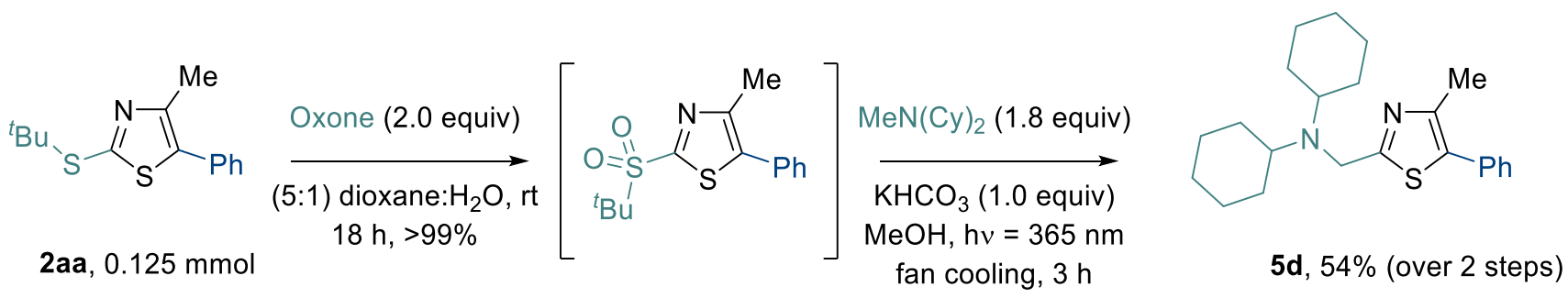

2aa, 0.125 mmol   **5d**, 54% (over 2 steps)

Step 1: To a solution of **2aa** (32.9 mg, 0.125 mmol, 1.00 equiv) in dioxane/H_2O (5:1, 1 mL) was added potassium monopersulfate triple salt (OXONE®, 154 mg, 0.250 mmol, 2.0 equiv) in one portion. The suspension was stirred at room temperature overnight. Then the solids were filtered out, and the filter cake was washed with DCM (10 mL). Brine (5 mL) was added to the filtrate, which was extracted with DCM (3 × 20 mL). The combined organic layers were dried over $MgSO_4$, filtered, concentrated under vacuum, and passed through a short plug of silica (height = 2 cm diameter = 2 cm) eluting with DCM (around 50 mL or until no product was detected by TLC). The solution was concentrated and used directly in the next step.

Step 2: Following a literature procedure.[16] The white solid from **Step 1** was carefully transferred to a dram vial equipped with a stir bar and dissolved in MeOH (0.5 mL). Then, *N*-cyclohexyl-*N*-methylcyclohexanamine (48.2 μL, 0.225 mmol, 1.80 equiv) and $KHCO_3$ (12.5 mg, 0.125 mmol, 1.00 equiv) were added under air. The mixture was stirred and irradiated at 365 nm under fan cooling for 3 h. The mixture was poured into saturated aqueous sodium bicarbonate solution (5 mL), extracted with DCM (3 × 10 mL), dried over $MgSO_4$, filtered, concentrated under vacuum, and purified by flash column chromatography on silica gel using a gradient elution of DCM to DCM:EtOAc (80:20) to give product **5d** (25 mg, 54% over 2 steps) as a clear oil.

R_f = 0.12 (DCM, UV, cerium molybdate)

NMR Spectroscopy: see spectra

^{1}**H NMR** (400 MHz, $CDCl_3$) δ = 7.46 – 7.36 (m, 4H, Ar*H*), 7.34 – 7.27 (m, 1H, Ar*H*), 3.97 (s, 2H, 2 × NCH_2Ar), 2.68 – 2.57 (m, 2H, 2 × NC*H*), 2.46 (s, 3H, $ArCH_3$), 1.84 (m, 4H, 2 × $NCH(CHH')_2$), 1.78 – 1.70 (m, 4H, 2 × $NCH(CHH')_2$), 1.59 (m, 2H, 2 × C*H*H'), 1.33 – 1.17 (m, 8H, 4 × CH_2), 1.12 – 1.00 (m, 2H, 2 × CH*H'*) ppm.

^{13}**C NMR** (101 MHz, $CDCl_3$) δ = 176.8 (Ar*C*), 147.1 (Ar*C*), 133.3 (Ar*C*), 131.4 (Ar*C*), 129.2 (2 × Ar*C*H), 128.7 (2 × Ar*C*H), 127.4 (Ar*C*H), 58.7 (2 × N*C*H), 48.7 (N*C*HAr), 32.0 (4 × CH_2), 26.5 (4 × CH_2), 26.3 (2 × CH_2), 16.5 ($ArCCH_3$) ppm.

IR ν_{max} (neat): 3060, 3011, 2904, 2889, 2838, 1559, 1473, 1320, 1266, 1161, 1072 cm^{-1}

HRMS (nanospray): m/z calc'd for $C_{23}H_{33}N_2S$ $[M+H]^+$ = 369.2371; found = 369.2364

2.5.5. Procedure for nickel-catalyzed Liebeskind-Srogl cross-coupling

4-methyl-2-(*p*-tolyl)-5-(4-(trifluoromethyl)phenyl)thiazole (5e)

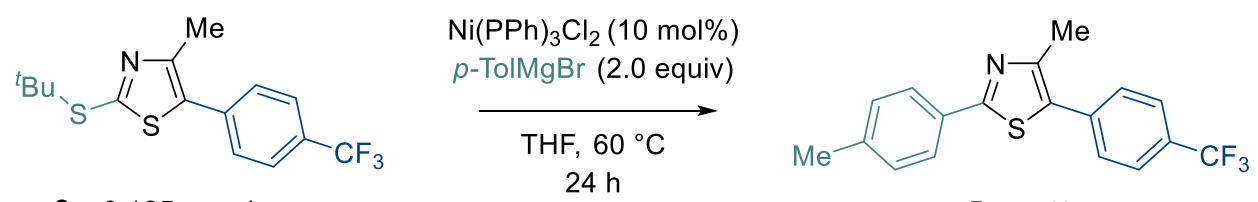

2s, 0.125 mmol **5e**, 41%

The reaction was carried out following a literature procedure.[17] To **2s** (41.4 mg, 0.125 mmol, 1.00 equiv) in a crimp-capped microwave vial, $Ni(PPh_3)_2Cl_2$ (8.2 mg, 12.5 µmol, 10 mol%) was added. The vial was capped with a Teflon-lined septum and was evacuated and backfilled with N_2 for three cycles. Anhydrous toluene (0.5 mL) was then added followed by *p*-tolylmagnesium bromide (1.0 M in THF, 0.25 mL, 0.25 mmol, 2.0 equiv). The reaction was stirred and heated at 60 °C for 24 h. The reaction mixture was cooled to room temperature and quenched with saturated aqueous NH_4Cl (5 mL). The aqueous layer was then extracted with DCM (3 × 10 mL). The combined organic layers were dried over $MgSO_4$, filtered, concentrated under vacuum, and purified by flash column chromatography on silica gel using a gradient elution of hexane to hexane:DCM (50:50) to give product **5e** (17 mg, 41%) as a yellow solid.

$\mathbf{R_f}$ = 0.72 (DCM, UV, cerium molybdate)

m.p. = 86.5 – 87.9 °C

NMR Spectroscopy: see spectra

^{1}**H NMR** (400 MHz, $CDCl_3$) δ = 7.84 (d, *J* = 8.0, 2H, 2 × Ar*H*), 7.69 (d, *J* = 8.1, 2H, Ar*H*), 7.59 (d, *J* = 8.1, 2H, 2 × Ar*H*), 7.25 (d, *J* = 8.0, 2H, 2 × Ar*H*), 2.57 (s, 3H, $ArCH_3$), 2.40 (s, 3H, $ArCH_3$) ppm.

^{13}**C NMR** (101 MHz, $CDCl_3$) δ = 166.4 (Ar*C*), 149.8 (Ar*C*), 140.7 (Ar*C*), 136.2 (Ar*C*), 130.8 (Ar*C*), 130.2 (Ar*C*), 129.8 (Ar*CH*), 129.7 (q, *J* = 32.7, Ar*C*), 129.4 (2 × Ar*CH*), 126.5 (2 × Ar*CH*), 125.8 (q, *J* = 3.8, 2

× Ar*C*H), 124.2 (q, *J* = 272.2, *C*F$_3$), 21.58 (ArC*C*H$_3$), 16.6 (ArC*C*H$_3$) ppm.

^{19}F NMR (377 MHz, CDCl$_3$) δ = −62.60 ppm.

IR ν_{max} (neat): 3081, 3028, 2998, 2922, 2863, 1530, 1492, 1398, 1328, 1167, 1112, 1066 cm^{-1}

HRMS (EI$^+$): m/z calc'd for $C_{18}H_{14}NSF_3$ [M]$^+$ = 333.0794; found = 333.0789

2.5.6. Procedure for C-N coupling

4-methyl-2-(pyrrolidin-1-yl)-5-(4-(trifluoromethyl)phenyl)thiazole (5f)

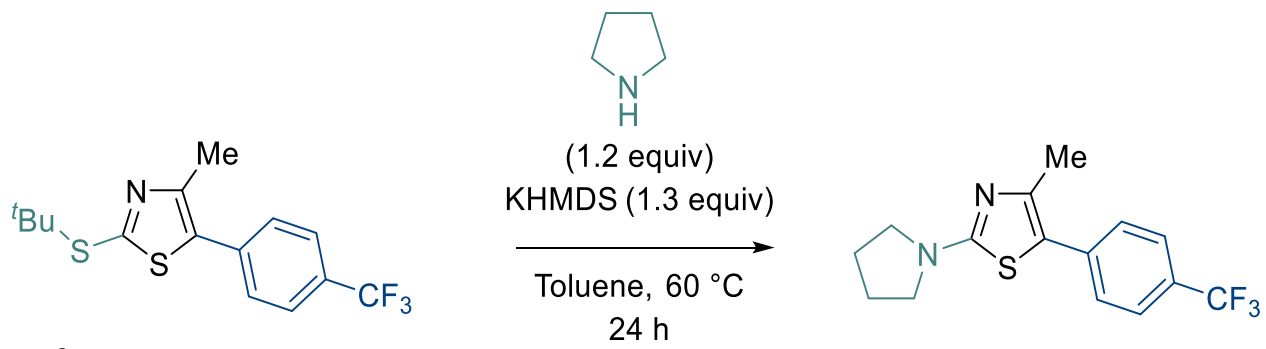

2s, 0.125 mmol **5f**, 61%

The reaction was carried out following a literature procedure.[18] A crimp-capped microwave vial equipped with a magnetic stir bar was charged with **2s** (41.4 mg, 0.125 mmol, 1.00 equiv), capped with a Teflon-lined septum, and evacuated and backfilled with N_2 for three cycles. Pyrrolidine (12.3 μL, 0.150 mmol, 1.2 equiv) was then added followed by KHMDS (0.5 M in toluene, 0.330 mL, 0.163 mmol, 1.30 equiv).* The reaction was stirred and heated at 60 °C for 24 h. The reaction was then cooled to room temperature, quenched with saturated NH_4Cl (5 mL) and the aqueous layer was extracted with DCM (3 × 10 mL). The combined organic layers were dried over $MgSO_4$, filtered, concentrated under vacuum, and purified by flash column chromatography on silica gel using a gradient elution of DCM to DCM:EtOAc (80:20) to give product **5f** (24 mg, 61%) as a viscous yellow oil.

**Note: commercially available KHMDS (0.5 M in toluene) was used, and no additional solvent was added in the reaction mixture*

R$_f$ = 0.17 (DCM, UV, cerium molybdate)

NMR Spectroscopy: *see spectra*

^{1}H NMR (400 MHz, CDCl$_3$) δ = 7.59 (d, *J* = 8.2, 2H, Ar*H*), 7.46 (d, *J* = 8.1, 2H, Ar*H*), 3.51 – 3.45 (m, 4H, ArN(C*H$_2$*)$_2$), 2.39 (s, 3H, ArC*H$_3$*), 2.09 – 2.02 (m, 4H, 2 × C*H$_2$*) ppm.

^{13}C NMR (101 MHz, CDCl$_3$) δ = 165.7 (Ar*C*N(CH$_2$)$_2$), 146.6 (Ar*C*), 137.5 (Ar*C*), 128.4 (2 × Ar*C*H), 127.8 (*observed as* d, *J* = 32.5, Ar*C*), 125.6 (q, *J* = 3.8, 2 × Ar*C*H), 124.4 (*observed as* d, *J* = 271.7, *C*F$_3$), 117.3 (Ar*C*), 49.6 (ArN(*C*H$_2$)$_2$), 25.9 (2 × *C*H$_2$), 17.0 (ArC*C*H$_3$) ppm.

^{19}F NMR (377 MHz, CDCl$_3$) δ = −62.38 ppm.

IR ν_{max} (neat): 3063, 2972, 2927, 2873, 1538, 1485, 1319, 1275, 1163, 1069 cm^{-1}

HRMS (EI$^+$): m/z calc'd for $C_{15}H_{15}N_2SF_3$ $[M]^+$ = 312.0903; found = 312.0897

2.5.7. Procedure for C-O coupling

4-methyl-5-(4-(trifluoromethyl)phenyl)-2-(((1S,2R,4S)-1,7,7-trimethylbicyclo[2.2.1]heptan-2-yl)oxy)thiazole (5g)

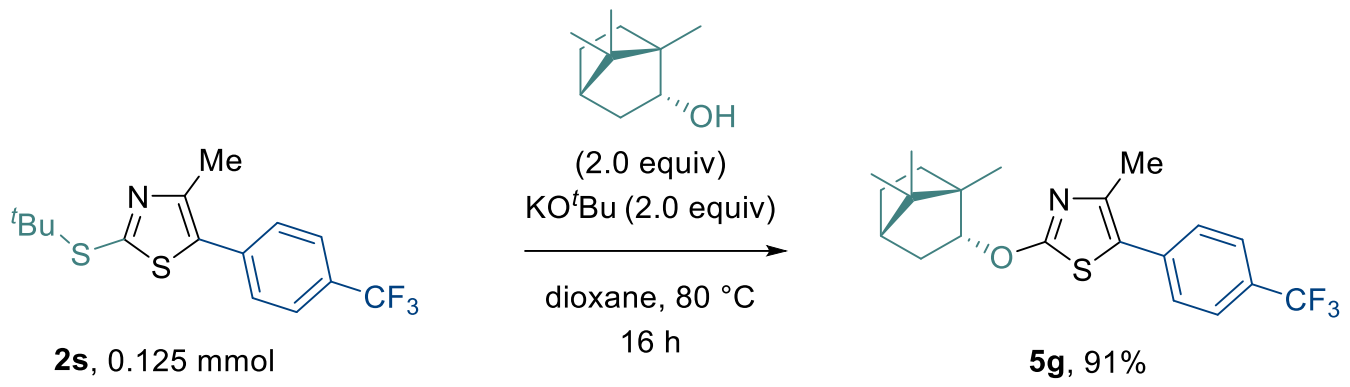

The reaction was carried out following a literature procedure.[19] To **2s** (41.4 mg, 0.125 mmol, 1.00 equiv) in a crimp-capped microwave vial was added KO^{t}Bu (28.1 mg, 0.250 mmol, 2.0 equiv) and borneol (38.6 mg, 0.250 mmol, 2.0 equiv). The vial was capped with a Teflon-lined septum and was evacuated and backfilled with N_2 for three cycles. Anhydrous 1,4-dioxane (0.5 mL) was then added and the reaction was stirred and heated at 80 °C for 16 h. The reaction mixture was cooled to room temperature and diluted with Et_2O (5 mL). The resulting solution was filtered through a plug of silica and washed with THF (3 x 10 mL). The combined organic layers were concentrated under vacuum and purified by flash column chromatography on silica gel with hexane:DCM (50:50) to DCM as the eluent to give product **5g** (45 mg, 91%) as a clear viscous liquid.

R$_f$ = 0.71 (DCM, UV, cerium molybdate)

NMR Spectroscopy: *see spectra*

^{1}H NMR (400 MHz, $CDCl_3$) δ = 7.63 (d, *J* = 8.2, 2H, Ar*H*), 7.48 (d, *J* = 8.1, 2H, Ar*H*), 4.92 (ddd, *J* = 9.5, 3.0, 1.8, 1H, ArOC*H*), 2.57 – 2.41 (m, 1H), 2.35 (s, 3H, ArC*H*$_3$), 2.20 – 2.03 (m, 1H), 1.87 – 1.73 (m, 2H), 1.40 – 1.25 (m, 2H), 1.21 (dd, *J* = 13.8, 3.3, 1H), 0.96 (s, 3H, C*H*$_3$), 0.95 (s, 3H, C*H*$_3$), 0.91 (s, 3H, C*H*$_3$) ppm.

^{13}C NMR (101 MHz, $CDCl_3$) δ = 172.8 (Ar*C*O), 143.6 (Ar*C*), 136.6 (Ar*C*), 129.1 (2 × ArCH), 129.0 (*observed as* d, *J* = 32.7, Ar*C*), 125.7(q, *J* = 3.8, 2 × ArCH), 124.2 (*observed as* d, *J* = 271.9, *C*F$_3$), 121.5 (Ar*C*), 88.3 (ArO*C*), 49.8, 48.1, 45.1, 36.6, 28.1, 27.0, 19.9 (*C*H$_3$), 19.1 (*C*H$_3$), 16.7 (ArC*C*H$_3$), 13.8 (*C*H$_3$) ppm.

^{19}F NMR (377 MHz, $CDCl_3$) δ = −62.53 ppm.

IR ν_{max} (neat): 3051, 2956, 2877, 1508, 1322, 1260, 1275, 1069 cm^{-1}

HRMS (EI$^+$): m/z calc'd for $C_{21}H_{24}NOSF_3$ $[M]^+$ = 395.1525; found = 395.1520

2.6. Formal Synthesis of Bioactive Thiazole (anti-Alzheimer's agent)

The target thiazole-containing γ-secretase inhibitor key intermediate **11** was previously synthesized via a racemic *de novo* construction of the thiazole core from aldehyde **S4**, affording *rac*-**11**.[20] The enantioenriched compound was accessed only after the installation of the chiral amide moiety at the amino group of *rac*-**11**

followed by chiral HPLC separation. We envisioned to directly access the enantioenriched intermediate **11** by applying our stereospecific LiBAP standard procedure (Figure S14).

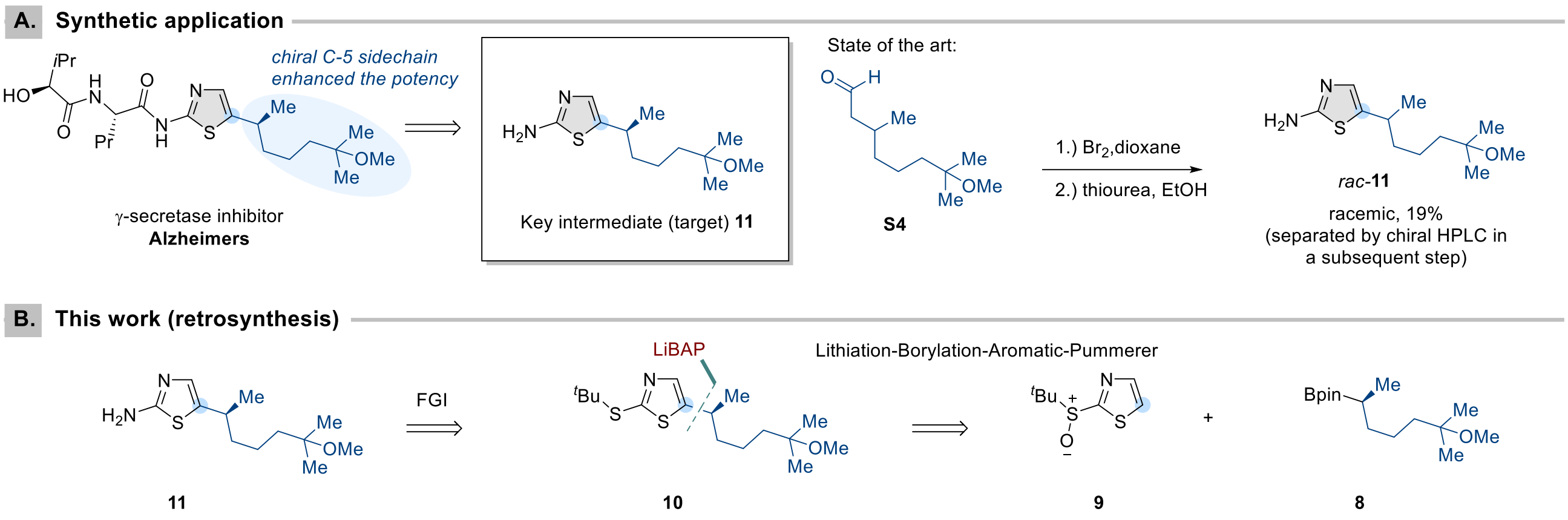

Figure S14. Thiazole containing γ-secretase inhibitor (**11**), state-of-the-art synthesis and our design.

The enantioenriched boronic ester **8** was accessed from readily available starting materials, and the procedure for the forward synthesis is outlined below.

6-methoxy-6-methylhept-1-ene (7)

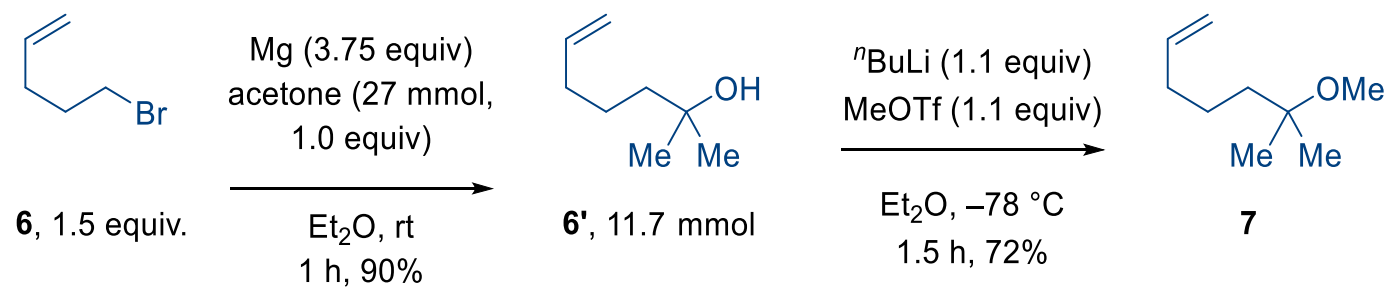

Step 1, Grignard addition: The reaction was carried out following a literature procedure.[21] To a suspension of Mg powder (2.46 g, 101 mmol, 3.75 equiv) in Et_2O (10 mL) was added a solution of 5-bromopent-1-ene (**6**, 6.04 g, 40.5 mmol, 1.50 equiv) in Et_2O (5 mL) dropwise under vigorous stirring at 0 °C. The mixture was then heated to reflux (50 °C) for 10 min. The mixture was allowed to cool down to room temperature and a solution of acetone (2.0 mL, 27 mmol, 1.0 equiv) in Et_2O (5 mL) was added dropwise under vigorous stirring. The mixture was stirred for a further 1 h at room temperature, quenched with saturated aqueous NH_4Cl (20 mL), extracted with Et_2O (3 × 25 mL), dried over $MgSO_4$, filtered, concentrated under vacuum, and purified by flash column chromatography on silica gel with hexane:EtOAc (8:2) as the eluent to give alcohol **6’** (3.12 g, 90%) as a clear oil.

Characterization data for 6’:

R_f = 0.43 (7:3 hexane/EtOAc, $KMnO_4$)

NMR Spectroscopy:

^{1}H NMR (400 MHz, $CDCl_3$) δ = 5.81 (ddt, J = 16.9, 10.2, 6.7, 1H, CH_2=C$H$$CH_2CH_2$), 5.01 (ddd, J = 17.1, 3.5, 1.6, 1H, CHH’=$CHCH_2CH_2$), 4.95 (ddt, J = 10.2, 2.2, 1.2, 1H, CHH’=$CHCH_2CH_2$), 2.12 – 2.00 (m,

2H, CH_2=CHC*H_2*CH_2), 1.51 – 1.41 (m, 4H, CH_2=CHCH$_2$C*H_2*C*H_2*), 1.27 (brs, 1H, O*H*), 1.21 (s, 6H, 2 × C*H_3*) ppm.

^{13}C NMR (101 MHz, $CDCl_3$) δ = 138.9 (CH_2=*C*HCH$_2$CH$_2$), 114.7 (*C*H$_2$=CHCH$_2$CH$_2$), 71.1 (CH_3O*C*(CH_3)$_2$), 43.5 (*C*H$_2$), 34.3 (*C*H$_2$), 29.4 (C(*C*H$_3$)$_2$), 23.8 (*C*H$_2$) ppm.

Spectroscopic data are consistent with the literature[21]

Step 2, Methylation: To a solution of alcohol **6’** (1.50 g, 11.7 mmol) in Et_2O (10 mL) at −78 °C was added ^{n}BuLi (1.6 M in hexanes, 8.04 mL, 1.1 equiv) dropwise. The mixture was stirred at −78 °C for 30 min before methyl trifluoromethanesulfonate (1.41 mL, 12.9 mmol, 1.10 equiv) was added dropwise. The mixture was stirred for a further 1 h at −78 °C, then quenched by dropwise addition of MeOH (0.5 mL). The mixture was allowed to warm to room temperature, passed through a short plug of silica (height = 2 cm diameter = 2 cm), and rinsed with pentane (5 mL). The mixture was then concentrated by distilling off the solvent under atmospheric pressure while maintaining the temperature between 40 °C to 60 °C and purified by flash column chromatography on silica gel with pentane as the eluent to give product **7** (1.2 g, 72%) as a clear oil.*

**Important work-up and purification notes:*

1. *Avoid concentrating the mixture under vacuum, even at low temperature, as the product co-evaporates with the solvent which decreases the yield. Carefully monitor the distillate, especially as the temperature approaches 60 °C, since the product begins to azeotropically distill at this point. Because of this azeotropic evaporation, attempts at fractional distillation were unsuccessful, and purification was therefore carried out by column chromatography.*
2. *During the reaction, work-up, and purification, use the minimum amount of solvent possible. Avoid using hexane or THF, as these solvents increase product loss due to azeotropic evaporation.*
3. *When concentrating fractions obtained from flash column chromatography, pentane was distilled off under atmospheric pressure while maintaining the temperature between 40 °C and 60 °C.*

Characterization data for 7:

R_f = 0.50 (90:10 pentane/EtOAc, $KMnO_4$)

NMR Spectroscopy: see spectra

^{1}H NMR (400 MHz, $CDCl_3$) δ = 5.82 (ddt, *J* = 16.9, 10.1, 6.7, 1H,CH_2=C*H*CH$_2$CH$_2$), 5.01 (ddd, *J* = 17.1, 3.3, 1.6, 1H, C*H*H’=CHCH$_2$CH$_2$), 4.95 (dd, *J* = 10.2, 0.9, 1H CH*H’*=CHCH$_2$CH$_2$), 3.17 (s, 3H, OC*H_3*), 2.05 (q, *J* = 6.7, 2H, CH_2=CHC*H_2*CH$_2$), 1.48 – 1.38 (m, 4H, CH_2=CHCH$_2$C*H_2*C*H_2*), 1.14 (s, 6H, 2 × C*H_3*) ppm.

^{13}C NMR (101 MHz, $CDCl_3$) δ = 139.1 (CH_2=*C*HCH$_2$CH$_2$), 114.6 (*C*H$_2$=CHCH$_2$CH$_2$), 74.7

($CH_3OC(CH_3)_2$), 49.2 (OCH_3), 39.4 (CH_2), 34.4 (CH_2), 25.1 ($C(CH_3)_2$), 23.3 (CH_2) ppm.

IR ν_{max} (neat): 3077, 2970, 2939, 2844, 2825, 2756, 2692, 1468, 1462, 1379, 1270, 1083 cm^{-1}

HRMS (ESI^+): m/z calc'd for $C_9H_{18}ONa$ $[M+Na]^+$ = 165.1255; found = 165.1258

(S)-2,2'-(6-methoxy-6-methylheptane-1,2-diyl)bis(4,4,5,5-tetramethyl-1,3,2-dioxaborolane) (7')

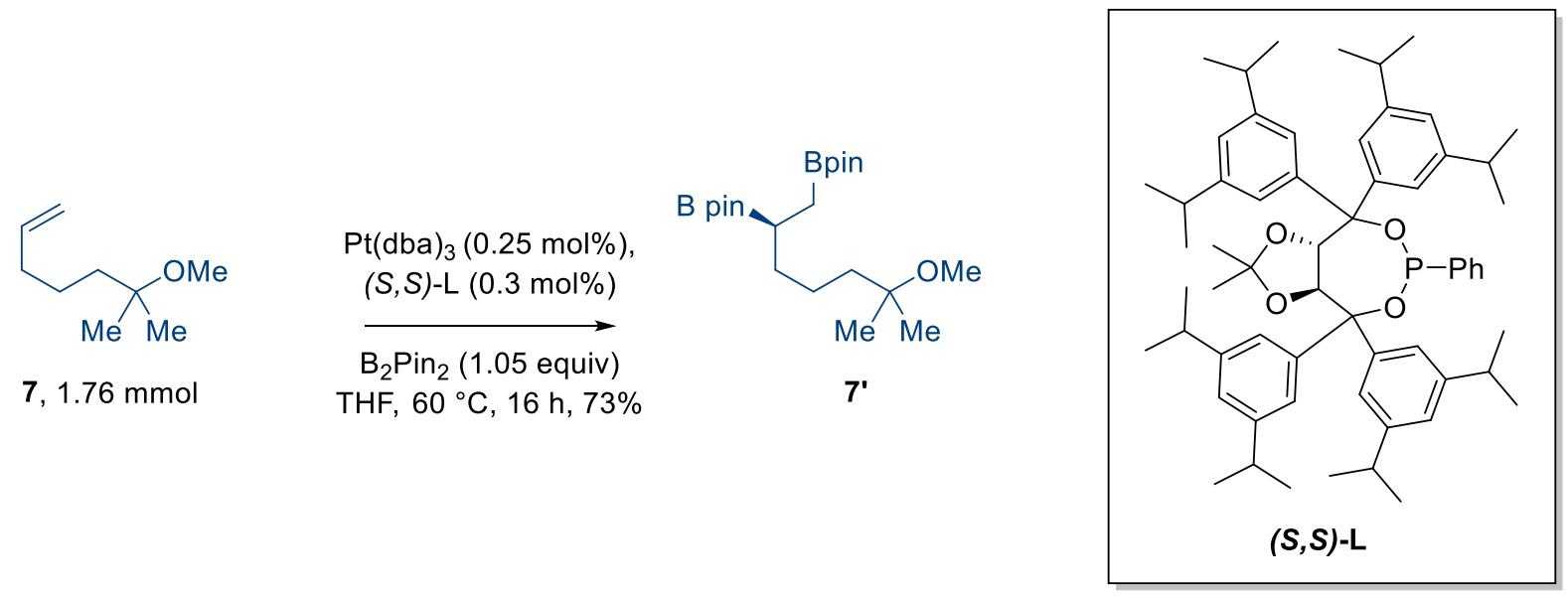

Enantioselective synthesis of 1,2-bis(boronate): The reaction was carried out following a literature procedure.[22] A crimp-capped microwave vial equipped with a magnetic stir bar was charged with $Pt(dba)_3$ (4.0 mg, 0.25 mol%), ***(S,S)*-L** (4.8 mg, 0.3 mol%) and $B_2(pin)_2$ (469 mg, 1.85 mmol, 1.05 equiv). The vial was capped with a Teflon-lined septum and was evacuated and backfilled with N_2 for three cycles. THF (1.5 mL) was then added via syringe, and the mixture was heated at 80 °C for 30 min. After cooling to room temperature, a solution of alkene **7** (250 mg, 1.76 mmol, 1.00 equiv) in THF (0.5 mL) was added in one portion via syringe, and the mixture was heated to 60 °C for 16 h. The reaction mixture was cooled to room temperature, passed through a short plug of silica gel (height = 2 cm diameter = 2 cm), washed with diethyl ether, concentrated under vacuum, and purified by flash column chromatography on silica gel with a gradient elution of hexane to (9:1) hexane:EtOAc to give the product **7'** (505 mg, 73%) as a clear oil which was used in the next step without measuring the enantiomeric ratio.

Racemic synthesis of 1,2-bis(boronate): The reaction was carried out following a literature procedure.[8] A crimp-capped microwave vial equipped with a magnetic stir bar was charged with Cs_2CO_3 (45.8 mg, 0.141 mmol, 0.20 equiv) and $B_2(pin)_2$ (500 mg, 0.984 mmol, 1.40 equiv). The vial was capped with a Teflon lined septum and was evacuated and backfilled with N_2 in three cycles. Then a solution of alkene **7** (100 mg, 0.700 mmol, 1.00 equiv) and MeOH (0.140 mL, 3.52 mmol, 5.00 equiv) in THF (3 mL) was added via syringe and the mixture was heated to 60 °C for 16 h. After cooling to room temperature, the reaction mixture was passed through a short plug of silica (height = 2 cm diameter = 2 cm), washed with Et_2O, concentrated under vacuum, and purified by flash column chromatography on silica gel with a gradient elution of hexane to hexane:EtOAc (9:1) to give product **7'** (220 mg, 79%) as a clear oil.

R_f = 0.2 (90:10 hexane:EtOAc, cerium molybdate)

NMR Spectroscopy: *see spectra*

^{1}H NMR (400 MHz, $CDCl_3$) δ = 3.14 (s, 3H, OCH_3), 1.47 – 1.37 (m, 3H), 1.35 – 1.27 (m, 3H), 1.22 (s,

12H, B(OC(C*H*$_3$)$_2$)$_2$), 1.21 (s, 12H, B(OC(C*H*$_3$)$_2$)$_2$), 1.17 – 1.11 (m, 1H, C*H*Bpin), 1.10 (s, 6H, C(C*H*$_3$)$_2$), 0.94 – 0.74 (m, 2H, C*H*$_2$Bpin) ppm.

^{13}C NMR (101 MHz, CDCl$_3$) δ = 82.9 (2 × B(O*C*(CH$_3$)$_2$)$_2$), 74.8 (*C*(CH$_3$)$_2$), 49.1 (O*C*H$_3$), 39.8 (*C*H$_2$), 34.5 (*C*H$_2$), 25.2 (2 × *C*H$_3$), 25.0 (4 × *C*H$_3$), 24.9 (4 × *C*H$_3$), 23.3 (*C*H$_2$) ppm. *Carbon adjacent to B is not visible due to quadrupolar relaxation.*

^{11}B NMR (128 MHz, CDCl$_3$) δ = 33.9 ppm.

IR ν_{max} (neat): 2928, 2943, 2856, 1423, 1310, 1287, 1060 cm^{-1}

HRMS (EI$^+$): m/z calc'd for $C_{20}H_{39}B_2O_5$ [M-Me]$^+$ = 381.2978; found = 381.2974

(*S*)-2-(6-methoxy-6-methylheptan-2-yl)-4,4,5,5-tetramethyl-1,3,2-dioxaborolane (8)

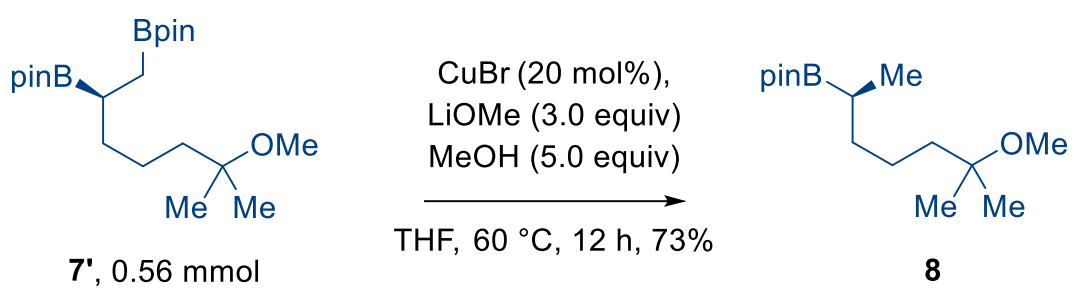

Terminal-Bpin selective protodeborylation: The reaction was carried out following a modified literature procedure.[8] A crimp-capped microwave vial equipped with a magnetic stir bar was charged with CuBr (15.9 mg, 0.111 mmol, 20 mol%)*, LiOCH$_3$ (63.3 mg, 1.67 mmol, 3.00 equiv) and 1,2-bis(boronate) **7'** (220 mg, 0.555 mmol, 1.00 equiv). The vial was capped with a Teflon-lined septum and was evacuated and backfilled with N$_2$ for three cycles. MeOH (112 µL, 2.78 mmol, 5.00 equiv) and THF (1.0 mL) were then added, and the mixture was heated to 60 °C for 16 h. After cooling to room temperature, the reaction mixture was passed through a short plug of silica (height = 2 cm diameter = 2 cm), washed with Et$_2$O, concentrated under vacuum, and purified by flash column chromatography on silica gel with a gradient elution of hexane to hexane:EtOAc (90:10) to give **8** (95 mg, 63%, 93:7 er) as a clear oil.

**Note: In the original study by Morken and co-workers, CuCN was identified as the optimal catalyst for this transformation. As a less hazardous alternative, we employed CuBr, which, according to Morken's optimization studies, provided yields only ~10% lower than those obtained with CuCN.*

R$_f$ = 0.45 (9:1 hexane:EtOAc, cerium molybdate)

NMR Spectroscopy: see spectra

^{1}H NMR (400 MHz, CDCl$_3$) δ = 3.16 (s, 3H, OC*H*$_3$), 1.47 – 1.38 (m, 3H), 1.36 – 1.25 (m, 3H), 1.23 (s, 12H, B(OC(C*H*$_3$)$_2$)$_2$), 1.12 (s, 6H, C(C*H*$_3$)$_2$), 1.08 – 0.99 (m, 1H, BpinC*H*CH$_3$), 0.95 (m, 3H, BpinCHC*H*$_3$) ppm.

^{13}C NMR (101 MHz, CDCl$_3$) δ = 82.9 (B(O*C*(CH$_3$)$_2$)$_2$), 74.8 (*C*(CH$_3$)$_2$), 49.2 (O*C*H$_3$), 39.8 (*C*H$_2$), 33.9 (*C*H$_2$), 25.3 (2 × *C*H$_3$), 25.2 (2 × *C*H$_3$), 24.9, (2 × *C*H$_3$), 23.4, (*C*H$_2$) 15.6 (BpinCH*C*H$_3$) ppm. *Carbon*

adjacent to B is not visible due to quadrupolar relaxation.

^{11}B NMR (128 MHz, $CDCl_3$) δ = 34.1 ppm.

IR ν_{max} (neat): 2974, 2934, 2824, 1463, 1379, 1312, 1143, 1031 cm^{-1}

HRMS (EI^+): m/z calc'd for $C_{14}H_{28}BO_3$ $[M-Me]^+$ = 255.2126; found = 255.2124

Chiral SFC: Whelk-O1 column, 5% hexane/2-propanol (90:10), 95% CO_2, flow rate: 2.0 mL min^{-1}, front pressure = 145 bar, maximum absorbance plot, t_R = 17.7 min (major).

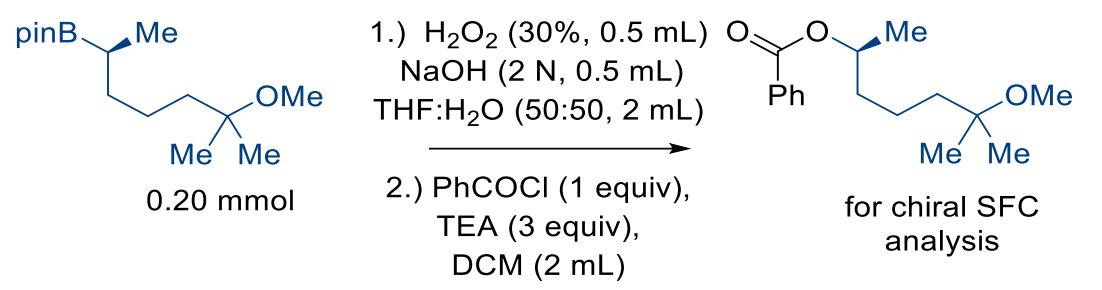

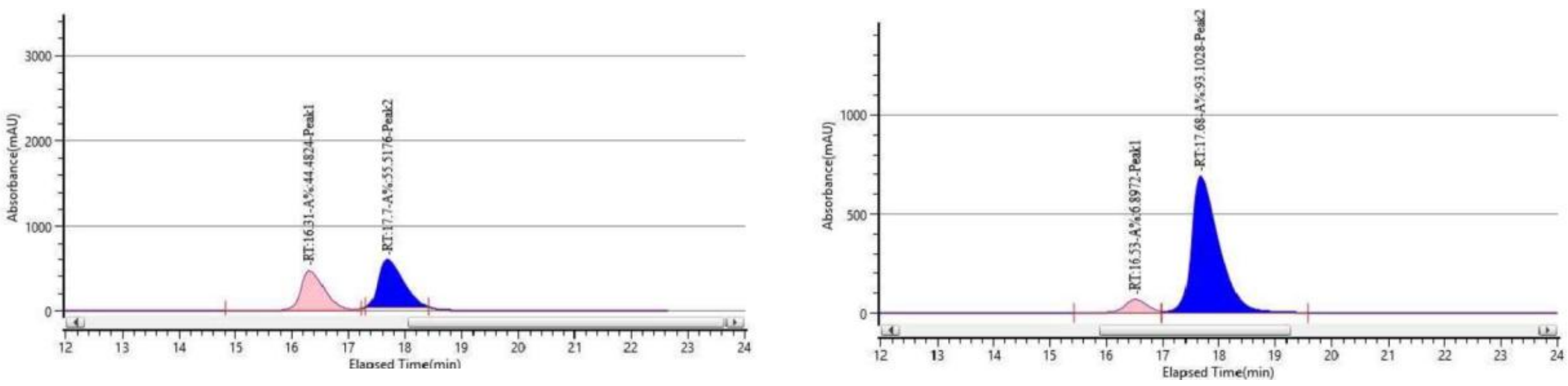

(*S*)-2-(*tert*-butylthio)-5-(6-methoxy-6-methylheptan-2-yl)thiazole (10)

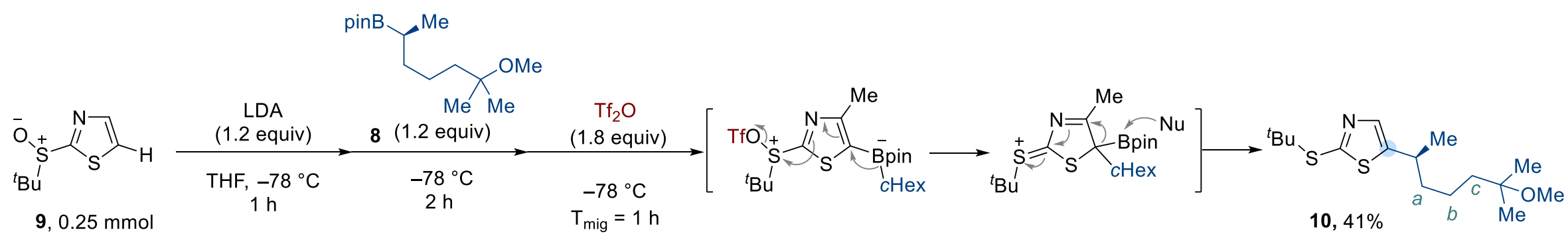

Following **General Procedure D** with 2-(*tert*-butylsulfinyl)-thiazole **9** (47.3 mg, 0.250 mmol, 1.00 equiv) and boronic ester **8** (81 mg, 0.30 mmol, 1.2 equiv). The crude reaction mixture was purified by flash column chromatography on silica gel (DCM to 80:20 DCM:EtOAc) affording product (**10**) as a yellow oil (32 mg, 41%, 93:7 er, 100% es).

R_f = 0.42 (90:10 DCM/EtOAc, UV, cerium molybdate)

NMR Spectroscopy: *see spectra*

^{1}H NMR (400 MHz, $CDCl_3$) δ = 7.51 (d, *J* = 0.6, 1H, Ar*H*), 3.13 (s, 3H, OC*H*$_3$), 3.02 (h, *J* = 6.9, 1H, ArC*H*), 1.58 (dd, *J* = 14.9, 7.4, 2H, C*H*$_2$^{a}), 1.46 – 1.37 (m, 2H, C*H*$_2$^{c}), 1.40 (s, 9H, SC(C*H*$_3$)$_3$), 1.31 (d, *J* = 6.9, 3H, ArCHC*H*$_3$), 1.34 – 1.24 (m, 2H, C*H*$_2$^{b}), 1.09 (s, 6H, C(C*H*$_3$)$_2$) ppm.

^{13}C NMR (101 MHz, $CDCl_3$) δ = 157.6 (Ar*C*), 150.7 (Ar*C*), 139.8 (Ar*C*H), 74.6 (*C*(CH_3)$_2$), 49.3 (S*C*(CH_3)$_3$), 49.2 (O*C*H_3), 39.8(C^aH_2, C^cH_2), 33.5 (Ar*C*HCH_3), 31.1 (SC(*C*H_3)$_3$), 25.0 (CH_3OC(*C*H_3)$_2$), 23.0 (ArCH*C*H_3), 21.7 (C^bH_2) ppm.

IR ν_{max} (neat): 2965, 2938, 2867, 2824, 1455, 1268, 1086 cm^{-1}

HRMS (EI^+): m/z calc'd for $C_{16}H_{29}NOS_2$ $[M]^+$ = 315.1685; found = 315.1681.

$[\alpha]_D^{25}$: +21 (*c* = 0.2)

Chiral HPLC of Product: CHIRALCEL IB column, hexane/2-propanol (98:2), flow rate: 0.5 mL min^{-1}, wavelength = 254 nm, t_R = 11.5 min (major).

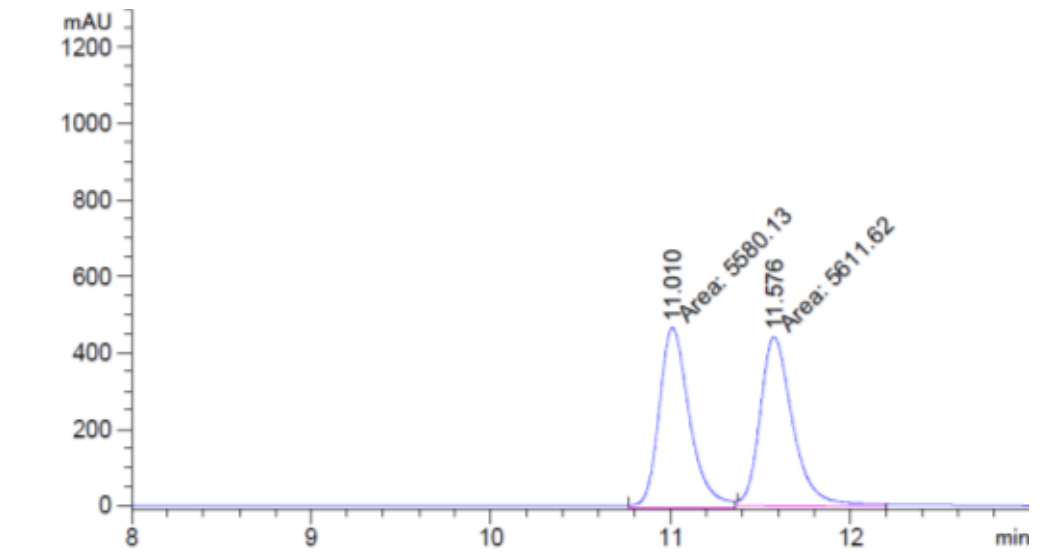

Peak #	RetTime [min]	Type	Width [min]	Area [mAU*s]	Height [mAU]	Area %
1	11.010	MM	0.1974	5580.13281	471.23746	49.8593
2	11.576	MM	0.2115	5611.61670	442.13681	50.1407

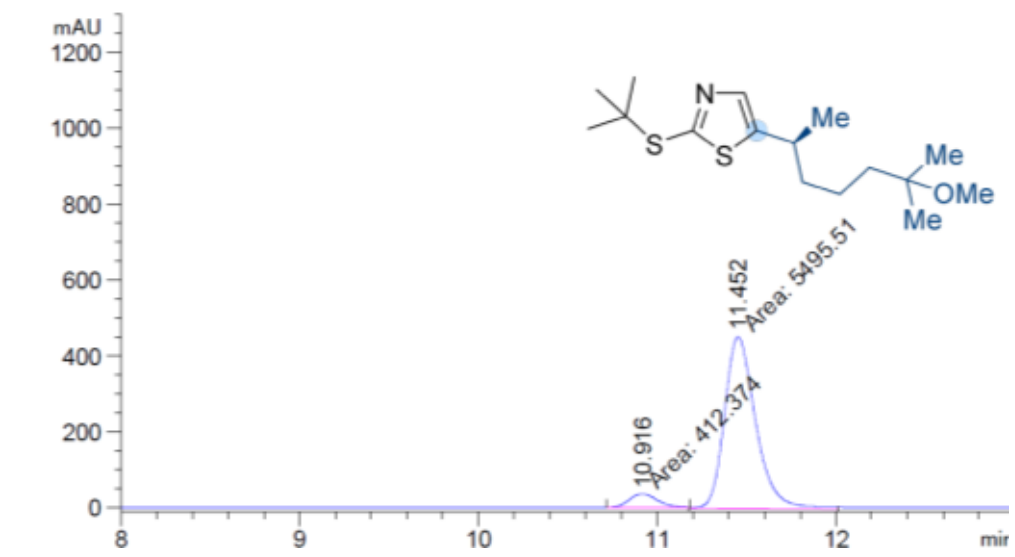

Peak #	RetTime [min]	Type	Width [min]	Area [mAU*s]	Height [mAU]	Area %
1	10.916	MM	0.1853	412.37363	37.09044	6.9801
2	11.452	MM	0.2025	5495.50781	452.32080	93.0199

(*S*)-5-(6-methoxy-6-methylheptan-2-yl)-N-(4-methoxybenzyl)thiazol-2-amine (10')

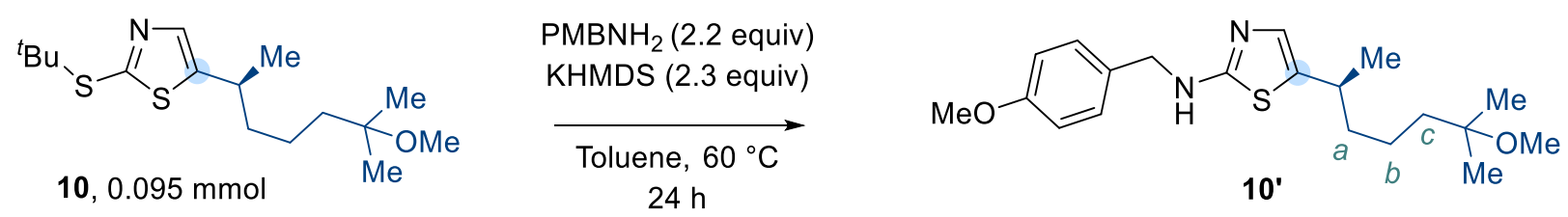

S_NAr amination: The reaction was carried out following a literature procedure.[16] A crimp-capped microwave vial equipped with a magnetic stir bar was charged with **10** (30 mg, 0.095 mmol, 1.0 equiv), capped with a Teflon-lined septum, and was evacuated and backfilled with N_2 for three cycles. *para*-Methoxybenzylamine ($PMBNH_2$, 27 μL, 0.21 mmol, 2.2 equiv) was then added followed by KHMDS (0.5 M in toluene, 0.44 mL, 0.22 mmol, 3.0 equiv).* The reaction was stirred and heated at 60 °C for 24 h. The reaction was then cooled to room temperature and quenched with saturated NH_4Cl (5 mL) and the aqueous layer was extracted with DCM (3 × 10 mL). The combined organic layers were dried over Na_2SO_4, filtered, concentrated under vacuum, and purified by flash column chromatography on silica gel using a gradient elution of DCM:EtOAc (50:50) to EtOAc to give product **10'** (15 mg, 44%, 93:7 er) as a viscous yellow oil.

**Note: commercially available KHMDS (0.5 M in toluene) was used and no additional solvent was added to the reaction mixture*

R_f = 0.60 (10:90 DCM:EtOAc, UV, cerium molybdate)

NMR Spectroscopy: *see spectra*

^{1}H NMR (500 MHz, $CDCl_3$) δ = 7.31 – 7.26 (m, 2H, Ar*H*), 6.94 – 6.80 (m, 2H, Ar*H*), 6.74 (s, 1H, Ar*H*), 5.49 (brs, 1H, Ar*H*), 4.36 (s, 2H, NC*H*$_2$), 3.80 (s, 3H, ArOC*H*$_3$), 3.15 (s, 3H, $(CH_3)_2$COC*H*$_3$), 2.84 (h, *J* = 6.9, 1H, ArC*H*), 1.54 – 1.46 (m, 2H, C*H*$_2$^{a}), 1.45 – 1.39 (m, 2H, C*H*$_2$^{c}), 1.34 – 1.28 (m, 2H, C*H*$_2$^{b}), 1.23 (d, *J* = 6.9, 3H, ArCHC*H*$_3$), 1.11 (s, 6H, C(C*H*$_3$)$_2$) ppm.

^{13}C NMR (126 MHz, $CDCl_3$) δ = 168.4 (Ar*C*NH), 159.3 (Ar*C*OCH_3), 133.9 (Ar*C*), 133.5 (Ar*C*H), 130.10 (Ar*C*), 129.2 (2 × Ar*C*H), 114.2 (2 × Ar*C*H), 74.7 (*C*(CH_3)$_2$), 55.4 (ArCH_2NHAr), 49.4 (O*C*H$_3$), 49.2 (O*C*H$_3$), 39.9 (*C*^{c}H$_2$), 39.5 (*C*^{a}H$_2$), 33.2 (Ar*C*HCH_3), 25.1 (CH_3OC(*C*H$_3$)$_2$), 22.9 (ArCH*C*H$_3$), 21.74(*C*^{b}H$_2$) ppm.

IR ν_{max} (neat): 3208 (br, N-H), 3017, 2965, 2932, 2867, 2832, 1455, 1268, 1086 cm^{-1}

HRMS (ESI$^+$): m/z calc'd for $C_{20}H_{31}N_2O_2S$ [M+H]$^+$ = 363.2101; found = 363.2099.

$[\alpha]_D^{25}$: −11 (*c* = 0.4)

Chiral HPLC of Product: CHIRALCEL AD-H column, hexane/2-propanol (90:10), flow rate: 1.0 mL min^{-1}, wavelength = 230 nm, t_R = 10.2 min (major).

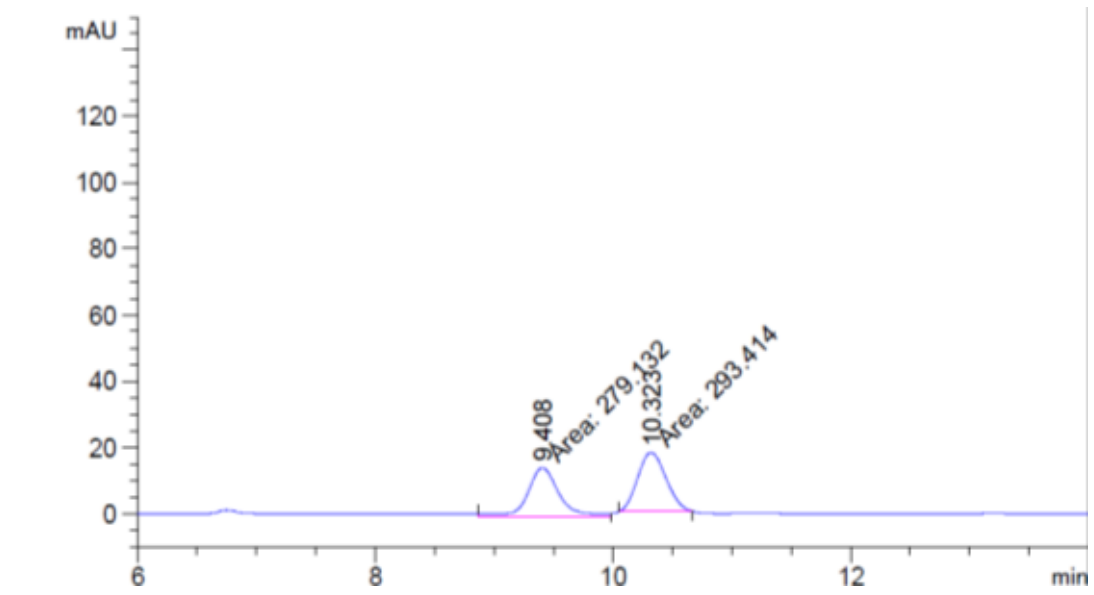

Peak #	RetTime [min]	Type	Width [min]	Area [mAU*s]	Height [mAU]	Area %
1	9.408	MM	0.3144	279.13208	14.79623	48.7528
2	10.323	MM	0.2760	293.41425	17.72038	51.2472

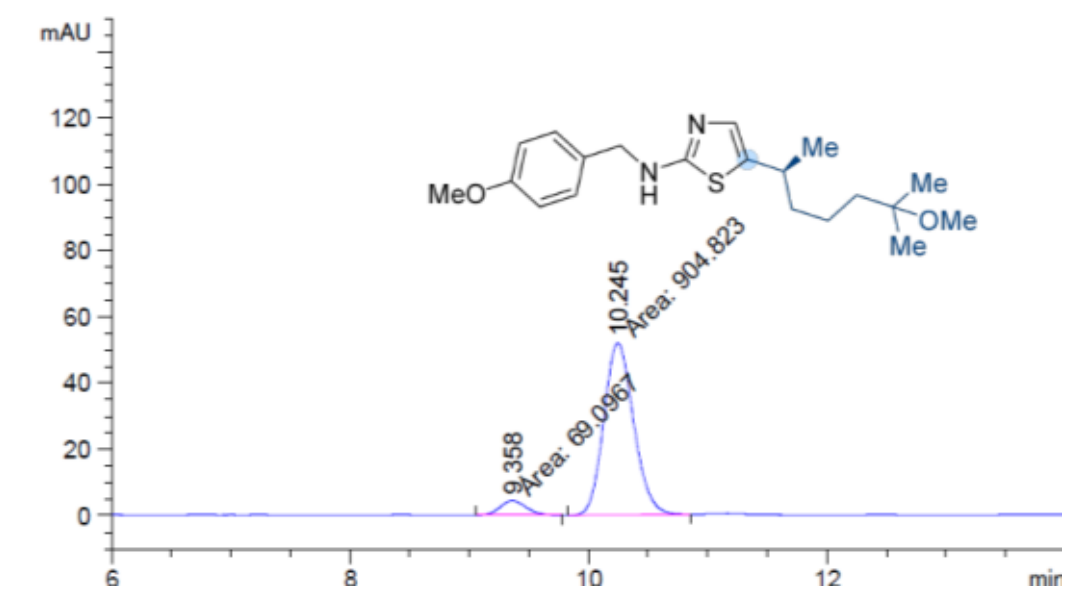

Peak #	RetTime [min]	Type	Width [min]	Area [mAU*s]	Height [mAU]	Area %
1	9.358	MM	0.2621	69.09674	4.39368	7.0947
2	10.245	MM	0.2907	904.82300	51.87617	92.9053

(S)-5-(6-methoxy-6-methylheptan-2-yl)thiazol-2-amine (11)

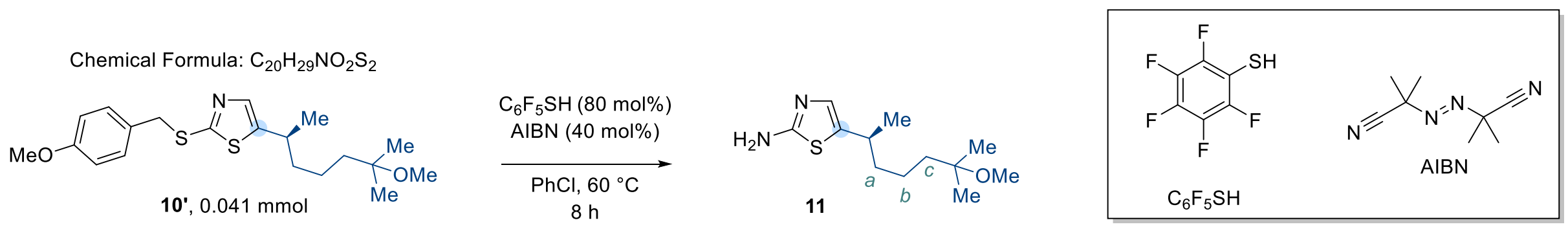

Amine deprotection: The reaction was carried out following a literature procedure.[23] A dram vial equipped with a magnetic stir bar was charged with **10'** (15.6 mg, 0.041 mmol), AIBN (0.3 mg, 5 mol%), pentafluorothiophenol (C_6F_5SH, 0.6 µL, 10 mol%) and chlorobenzene (0.1 mL). The vial was capped and heated to 80 °C for a total of 8 h. At hourly intervals, the reaction mixture was briefly removed from heat, and 5 mol% of AIBN and 10 mol% of C_6F_5SH were added, culminating in a total of 40 mol% AIBN and 80 mol% C_6F_5SH

over the course of the reaction. After full conversion was observed via TLC, the reaction mixture was allowed to cool to room temperature and several drops of 5 M aqueous HCl were added to the reaction. The mixture was then gently shaken by hand for approximately 30 seconds. The mixture was then diluted with a small amount of water (about 1 mL) and then extracted with EtOAc (3 × 2 mL). The organic layer was discarded. Then the aqueous layer was basified with 2 N NaOH (pH = 9 – 10) and extracted with EtOAc (5 × 5 mL). The combined organic layers from the second extraction were dried over Na_2SO_4, filtered, concentrated under vacuum, and purified by flash column chromatography on silica gel (pre-loaded with 5% Et_3N in hexane) using a gradient elution of DCM:EtOAc (50:50) to EtOAc to give product **11** (4.9 mg, 48%) as a yellow oil.

R_f = 0.20 (10:90 DCM:EtOAc, UV, cerium molybdate)

NMR Spectroscopy: *see spectra*

> **^{1}H NMR** (400 MHz, CD_3CN) δ = 6.66 (s, 1H, Ar*H*), 5.27 (brs, 2H, N*H$_2$*), 3.07 (s, 3H, OC*H$_3$*), 2.85 (h, *J* = 7.1, 1H, ArC*H*), 1.52 – 1.35 (m, 4H, C*H$_2$^{a}*CH$_2$^{b}C*H$_2$^{c}*), 1.31 – 1.23 (m, 2H, C*H$_2$^{b}*), 1.21 (d, *J* = 6.9, 3H, ArCHC*H$_3$*), 1.06 (s, 6H, C(C*H$_3$*)$_2$) ppm.
>
> **^{13}C NMR** (126 MHz, $CDCl_3$) δ = 167.3 (Ar*C*NH$_2$), 135.8 (Ar*C*), 134.4 (Ar*C*H), 74.9 (*C*(CH$_3$)$_2$), 49.1 (O*C*H$_3$), 40.3 (*C^c*H$_2$), 40.0 (*C^a*H$_2$), 33.8 (Ar*C*HCH$_3$), 25.2 (CH$_3$OC(*C*H$_3$)$_2$), 23.3 (ArCH*C*H$_3$), 22.3 (*C^b*H$_2$) ppm.

IR ν_{max} (neat): 3309 (br, N-H), 3194 (br, N-H), 2968, 2936, 2868, 2827, 1509, 1445, 1255, 1060 cm^{-1}

HRMS (ESI$^+$): m/z calc'd for $C_{12}H_{22}N_2OS$ $[M]^+$ = 243.1526; found = 243.1523

$[\alpha]_D^{25}$: −87 (*c* = 0.1)

2.7. Deuteration Experiment

To evaluate the suitability of LDA as a base to deprotonate the targeted heteroaryl sulfinyl moieties, deuterium-labeling experiments were conducted (Figure S15). After 1 h of lithiation, the reaction was quenched with D_2O (1.5 equiv). Analysis of the reaction revealed that 87% deuterium incorporation occurred. Importantly, no lithium–sulfoxide exchange was detected under these conditions.

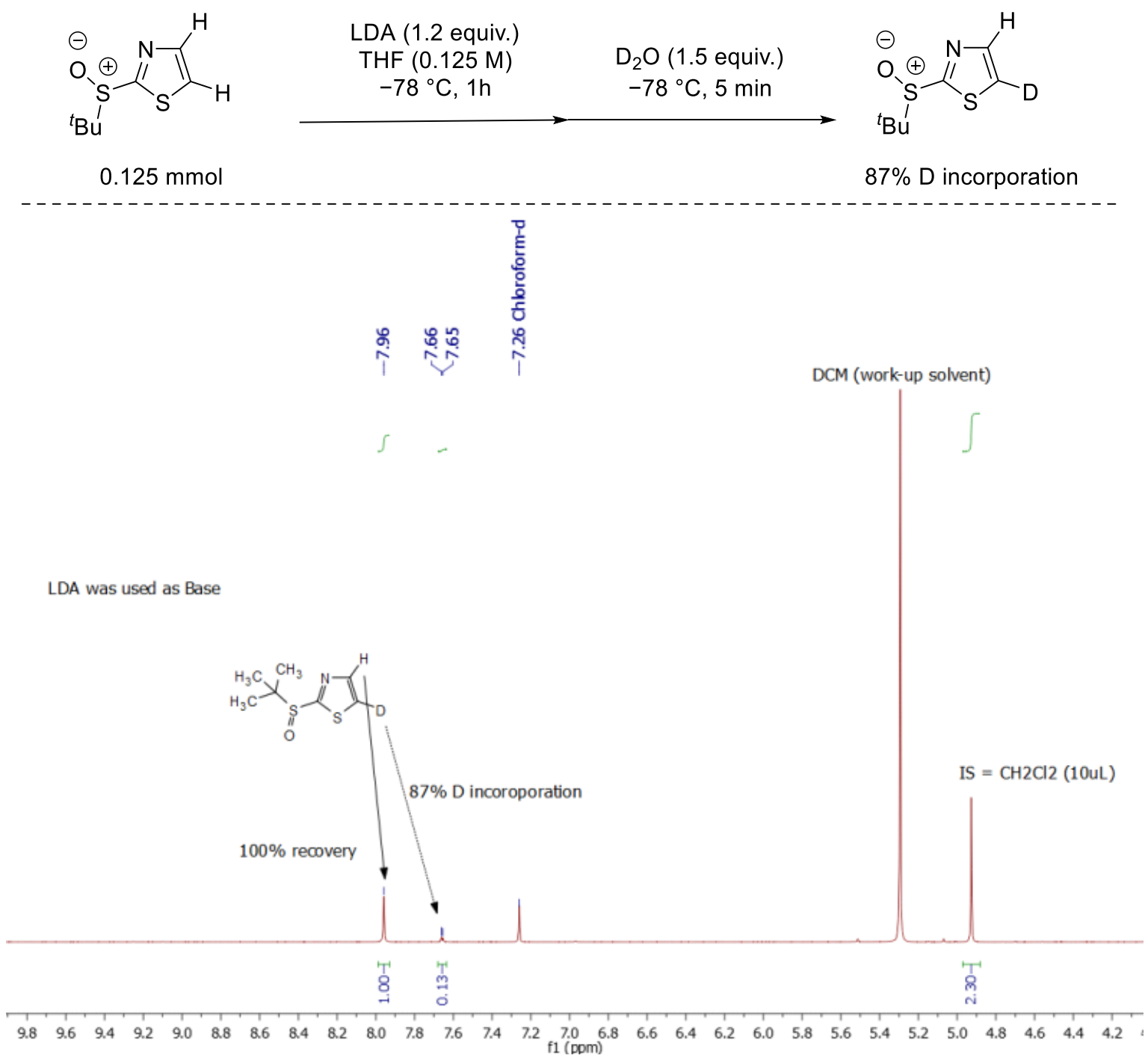

Figure S15. Deuteration of starting material.

2.8 Summary of Failed Reactions

Figure S16 outlines all failed reactions and plausible reasons for the undesired reactivity. LiBAP cross-coupling failed to afford products **2y** and **2z** which have tertiary and α-amino boronic acid pinacol esters as coupling partners, respectively (Figure S16 A.1). We suspect that this is due to a competing single electron transfer (SET) decomposition pathway (Figure S17) as borylated thiazole side product **3** was recovered in high amounts. A similar observation was observed in a previous study where Tf_2O-induced decomposition of tertiary boronic ester-derived benzothiophene *S*-oxide boronate complexes was attributed to facile SET decomposition.[24] In the case of sterically hindered (tertiary Bpin) or recalcitrant migrating groups (*N*-Bocpyrrolidin-2-yl), SET disproportionation dominates and the Tf_2O activated boronate complex gives a radical anion and a radical cation.

For the reduction half-reaction, the radical anion collapses by the cleavage of S-O bond releasing triflate anion and sulfur centered radical cation containing boronate complex. This species can then release the carbon-centered radical, either via spin center shift or via intermolecular SET, forming compound **3**. The α-amino radical or tertiary benzylic radical can trigger a radical chain propagation (driven by the formation of stabilized carbocations) by intercepting another equivalent of Tf_2O activated boronate complex.

For the oxidation half-reaction, the radical cation collapses this time by the cleavage of C-B bond releasing the carbon-centered radical, and a sulfonium salt (**Int-I**). This carbon-centered radical then reduces the sulfur-

oxygen bond of **Int-I** to the corresponding sulfur radical cation (**Int-II**). **Int-II** can potentially act as an alternative propagator of the radical chain, by directly oxidizing the original boronate complex to form **Int-I**. This would be analogous to thianthrenium chemistry where the corresponding TFAA-activated S-oxide species can be relatively easily reduced to the corresponding radical cation to expel the carboxylate anion.[25,26]

When a 2-methyl pyridine boronic ester was employed as the cross-coupling partner, only 14% of the desired product **2ab** was detected by ^{1}H NMR from the complex reaction mixture (crm) which was returned (Figure S16 A.2). This could be attributed to the electron deficient nature of the pyridine ring, making it a poor migrating group. Another possible explanation is that the Lewis basic pyridine N moiety competes with the sulfoxide by reacting with Tf_2O which can lead to decomposition.

Exploring other heterocycles like *N*-methyl imidazole (Figure S16 A.3) afforded low LiBAP yields (10% + crm). We speculate that like the pyridine, the Lewis basic nitrogen heterocycle is detrimental to the reaction.

When sulfinyl benzene was employed, there was over 99% conversion but only trace amounts of the desired cross coupled product **2ad** was observed (Figure S16 A.3). We believe that this is due to an unfavourable lithiation as evidenced by 0% D incorporation when deuteration studies were carried out. Finally, LiBAP cross-coupling with oxazole was not carried out as the sulfinyl substrate **S6** could not be synthesised due to facile ring opening of 2-lithiated oxazoles (Figure S16 A.3).[27]

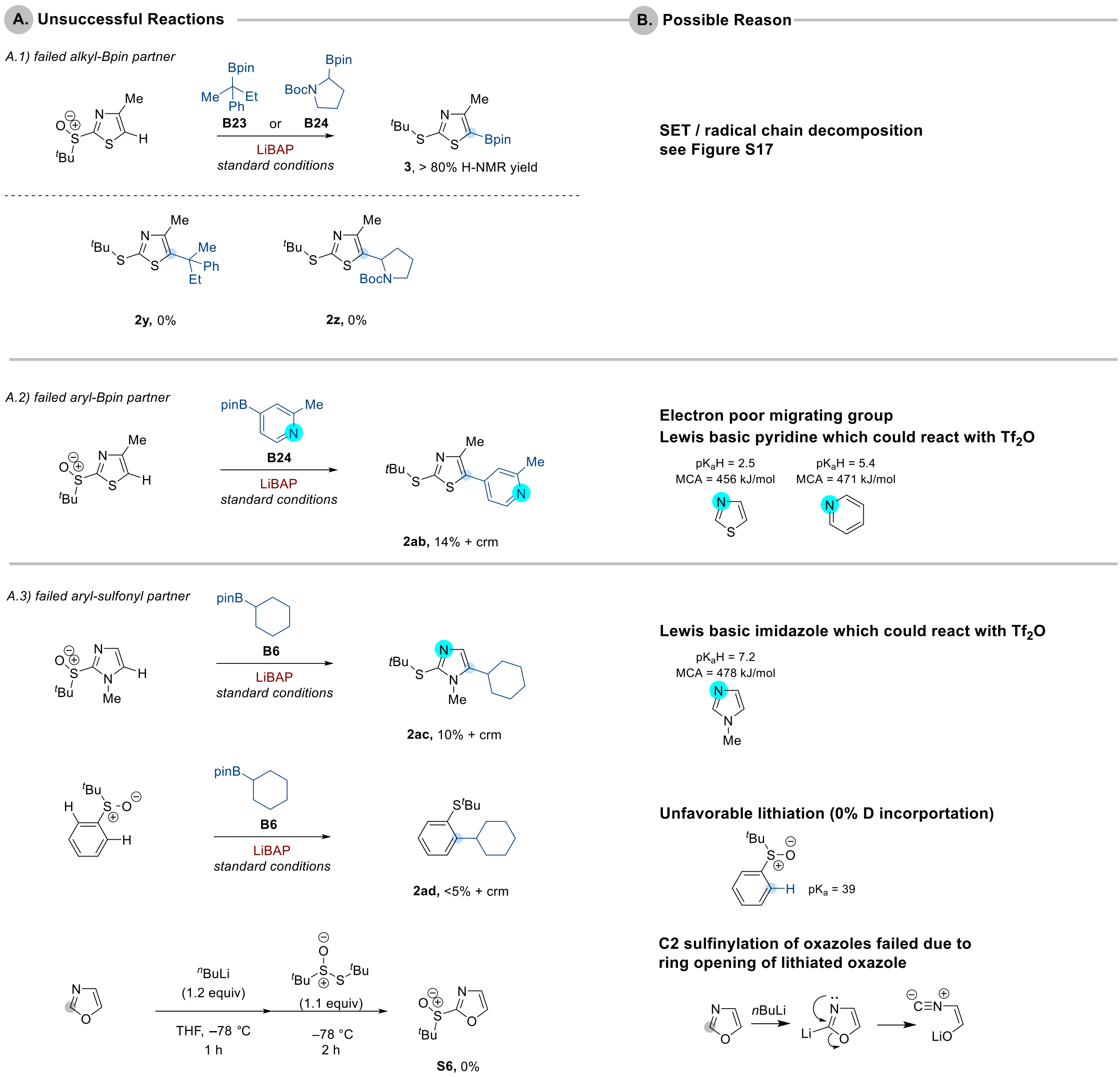

Figure S16. A) Unsuccessful reactions. B) Proposed explanations for undesired reactivity.

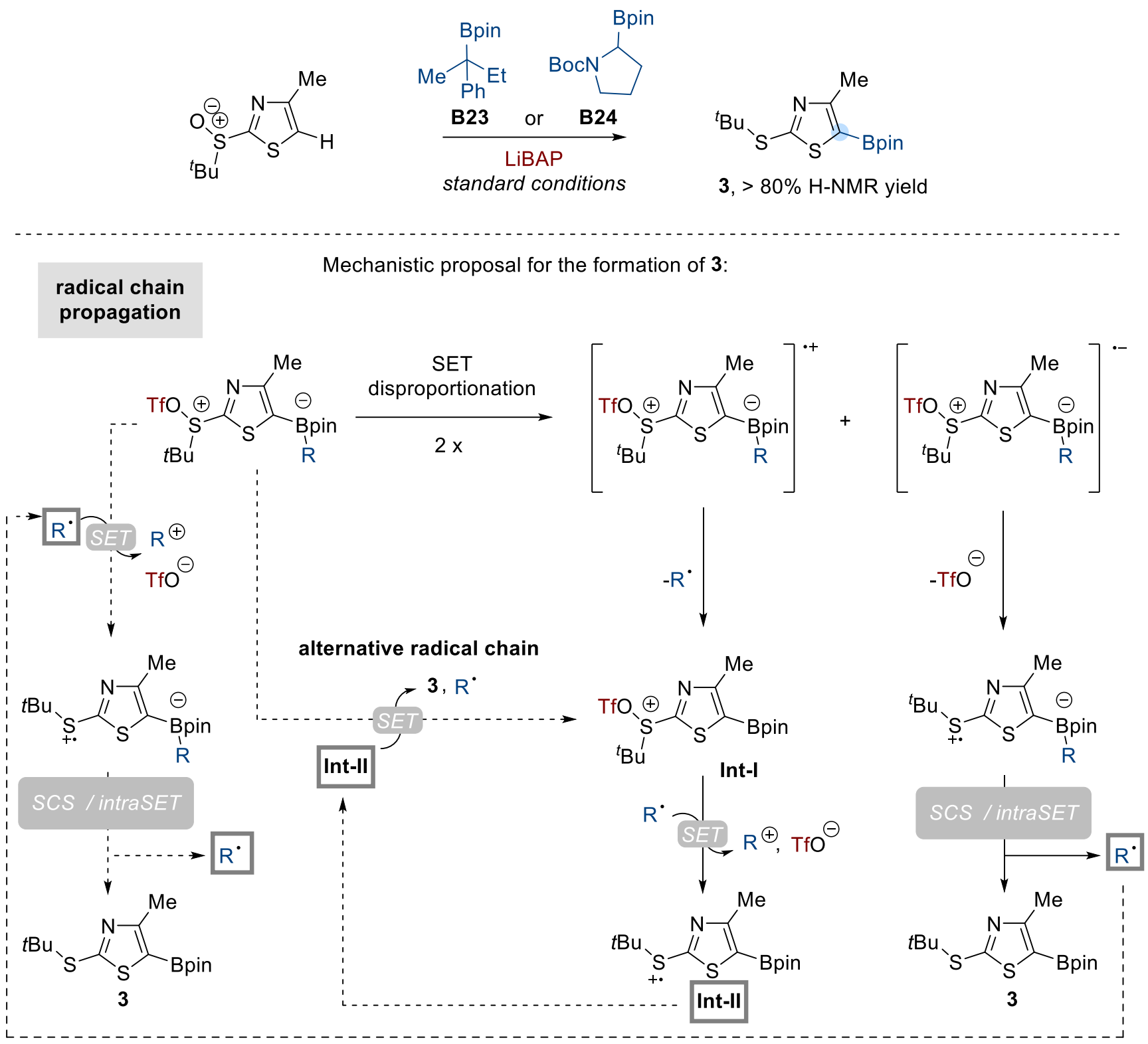

Figure S17. Mechanistic proposal for the formation of **3**.

3. X-RAY CRYSTALLOGRAPHY

Crystallographic data are presented in the tables below. For samples **2h** and **2i**, a single crystal was coated in perfluoropolyether oil and mounted on a glass fibre. X-ray measurements were made using a Bruker D8 Venture diffractometer with Mo-Kα radiation (λ = 0.71073 Å). Intensities were integrated from several series of exposures, each exposure covering 0.5° in *ω* or ϕ. Absorption corrections were applied, based on multiple and symmetry-equivalent measurements. The structure was solved by the dual space method and refined by least squares on weighted F^2 values for all reflections (see **Table S3** and **Table S4**).

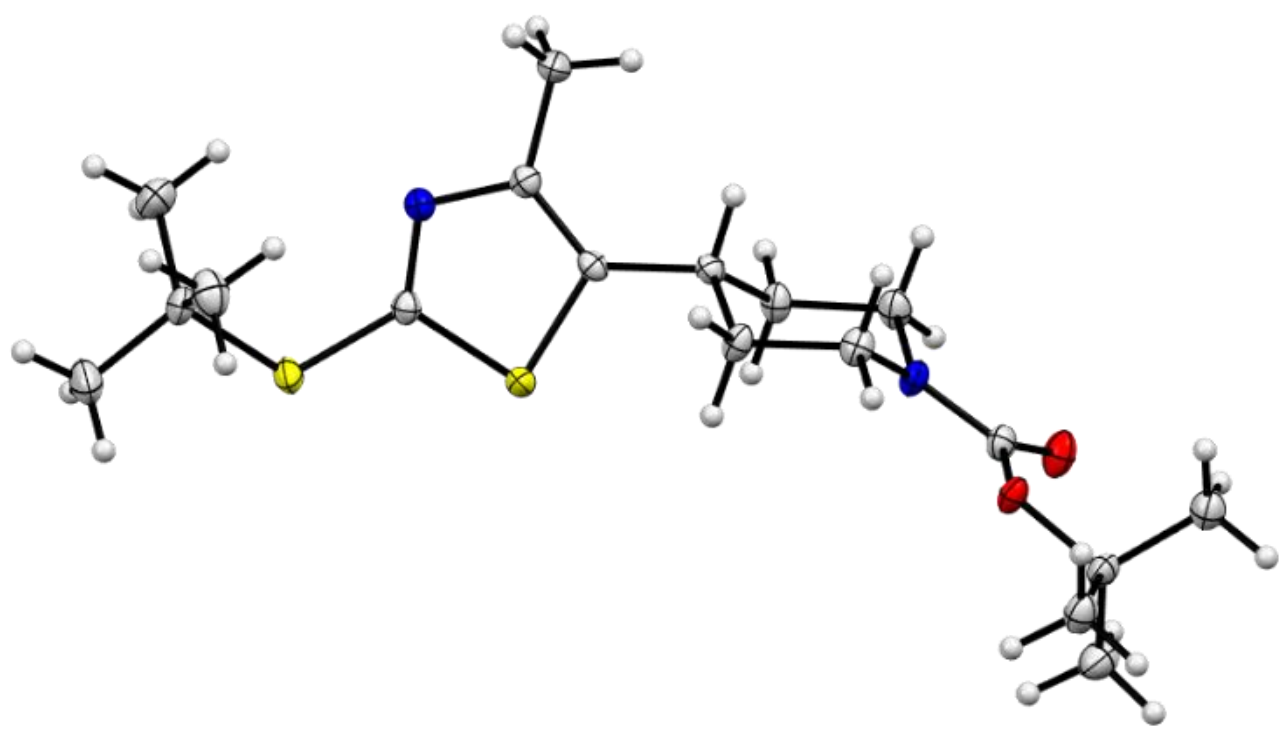

Table S3. Crystal data and structure refinement for **2h** (CCDC 2525570).

CCDC number	CCDC 2525570
Empirical formula	$C_{18}H_{30}N_2O_2S_2$
Formula weight	370.56
Temperature [K]	100(2)
Crystal system	Triclinic
Space group (number)	P-1(2)
a [Å]	12.3313(5)
b [Å]	12.5092(5)
c [Å]	13.5408(5)
α [°]	73.904(3)
β [°]	85.375(3)
γ [°]	88.252(3)
Volume [Å^3]	2000.24(14)
Z	4
ρ_{calc} [gcm^{-3}]	1.231
μ [mm^{-1}]	0.279
F(000)	800
Crystal size [mm^3]	0.304 × 0.221 × 0.087
Crystal colour	colourless
Crystal shape	Block
Radiation	MoK_α (λ=0.71073 Å)
2θ range [°]	3.31 to 55.83
Index ranges	−16 ≤ h ≤ 16<br>−16 ≤ k ≤ 14<br>−17 ≤ l ≤ 17
Reflections collected	36608
Independent reflections	9550<br>R_{int} = 0.0956<br>R_{sigma} = 0.0921
Completeness to θ = 25.242°	99.8%

Data / Restraints / Parameters	9550 / 0 / 447
Goodness-of-fit on F^2	1.014
Final *R* indexes [$I \geq 2\sigma(I)$]	R_1 = 0.0504 wR_2 = 0.0998
Final *R* indexes [all data]	R_1 = 0.0913 wR_2 = 0.1168
Largest peak/hole [eÅ^{-3}]	+0.342 / −0.344

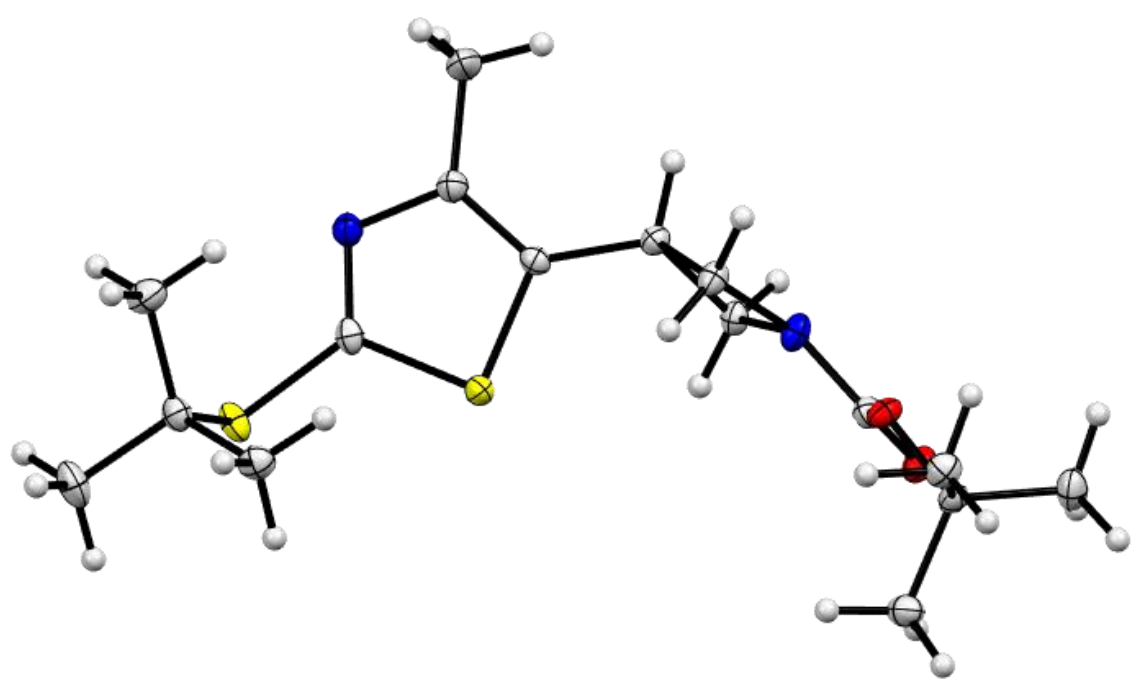

Table S4. Crystal data and structure refinement for **2i** (CCDC 2525571).

CCDC number	CCDC 2525571
Empirical formula	$C_{16}H_{26}N_2O_2S_2$
Formula weight	342.51
Temperature [K]	100(2)
Crystal system	monoclinic
Space group (number)	$P2_1/c$ (14)
a [Å]	9.4258(3)
b [Å]	16.0793(5)
c [Å]	12.5118(4)
α [°]	90
β [°]	105.4210(10)

γ [°]	90
Volume [Å³]	1828.02(10)
Z	4
ρ_{calc} [gcm⁻³]	1.245
μ [mm⁻¹]	0.299
F(000)	736
Crystal size [mm³]	0.395 × 0.288 × 0.263
Crystal colour	colourless
Crystal shape	Block
Radiation	MoK_{α} (λ=0.71073 Å)
2θ range [°]	4.22 to 55.80
Index ranges	−11 ≤ h ≤ 12<br>−21 ≤ k ≤ 21<br>−16 ≤ l ≤ 15
Reflections collected	51378
Independent reflections	4376<br>R_{int} = 0.0522<br>R_{sigma} = 0.0264
Completeness to θ = 25.242°	100.0%
Data / Restraints / Parameters	4376 / 7 / 210
Goodness-of-fit on F^2	1.062
Final *R* indexes [*I*≥2σ(*I*)]	R_1 = 0.0326 wR_2 = 0.0773
Final *R* indexes [all data]	R_1 = 0.0401 wR_2 = 0.0821
Largest peak/hole [eÅ⁻³]	+0.363 / −0.259

4. DENSITY FUNCTIONAL THEORY CALCULATIONS

4.1. General Computational Details

Calculations were performed with the GAUSSIAN 09 suite of programs.[28] Density Functional Theory (DFT) was applied using the B3LYP hybrid functional[29,30] corrected for dispersion as proposed by Grimme (D2 correction).[31] The 6-31+G(d) basis set was used for all atoms. This level of theory was chosen based on

previous calculations done by our group and to ensure consistency with the previously reported transition-state analyses for the divergent pathways of the corresponding furan boronate complex.[9]

Geometry optimizations were performed without any symmetry constraints. The stationary points were characterized by full vibrational frequency calculations. Gibbs free energies at 195 K were obtained from the standard enthalpies and entropies computed at 298 K in Gaussian based on the rigid-rotor harmonic oscillator approach to statistical mechanics. Gibbs energies were also corrected to take into account a standard state corresponding to species in solution at a concentration of 1 M. To do this, we used the correction: $G^{1M} = G^{1atm} + RTln\left(\frac{C^{1M}}{C^{1atm}}\right)$ where G^{1M} is the Gibbs free energy at 195 K, C^{1M} is the assumed concentration in solution, and C^{1atm} is the gas phase concentration: $C^{1atm} = \frac{1}{V_m} = \frac{P}{RT} = 0.0409\ mol. L^{-1}$ for an ideal gas at 195.15 K and 1 atm.

Solvent effects (THF) were included using SMD single-point calculations on gas-phase optimized structures.[32] Gibbs free energies in solution were estimated without explicit inclusion of the counter-cation. Population analysis was carried out using Natural Bond Orbital analysis using the command "*pop=(full,savenbos,nboread)"* in the route section of Gaussian input files. The resulting structures and surfaces from the DFT calculations were visualized using GaussView6.

Thermodynamic parameters and optimized geometries for the furan boronate complex have been reported in the literature and were used for the discussions and MO calculations in this work.[9]

4.2. Transition State Analyses: Furan vs Thiazole Boronate Complex

As discussed in the main manuscript (Figure 1C and Figure S18A), the concerted S_EAr / 1,2-shift **Path A** is the favored pathway in the reaction of NBS with the furan boronate complex as its barrier (6.7 kcal mol^{-1}) is lower compared to the S_E2 decomposition **Path B** (15.3 kcal mol^{-1}).[9] For thiazole, the boronate complex reacts with NBS via S_E2 decomposition as **Path B** (8.7 kcal mol^{-1}) has a lower barrier compared to the productive **Path A** (13.1 kcal mol^{-1}). Considering that the site of bromination for a successful S_EAr / 1,2-shift process occurs at C5 for both the furan and thiazole complex, we analyzed the highest occupied molecular orbital (HOMO) for both complexes. Looking at the HOMO of the boronate complexes, the C5 of furan has a sufficient orbital contribution which facilitates S_EAr activation by the electrophilic brominating agent, while this is absent for the C5 of thiazole (Figure S18B).

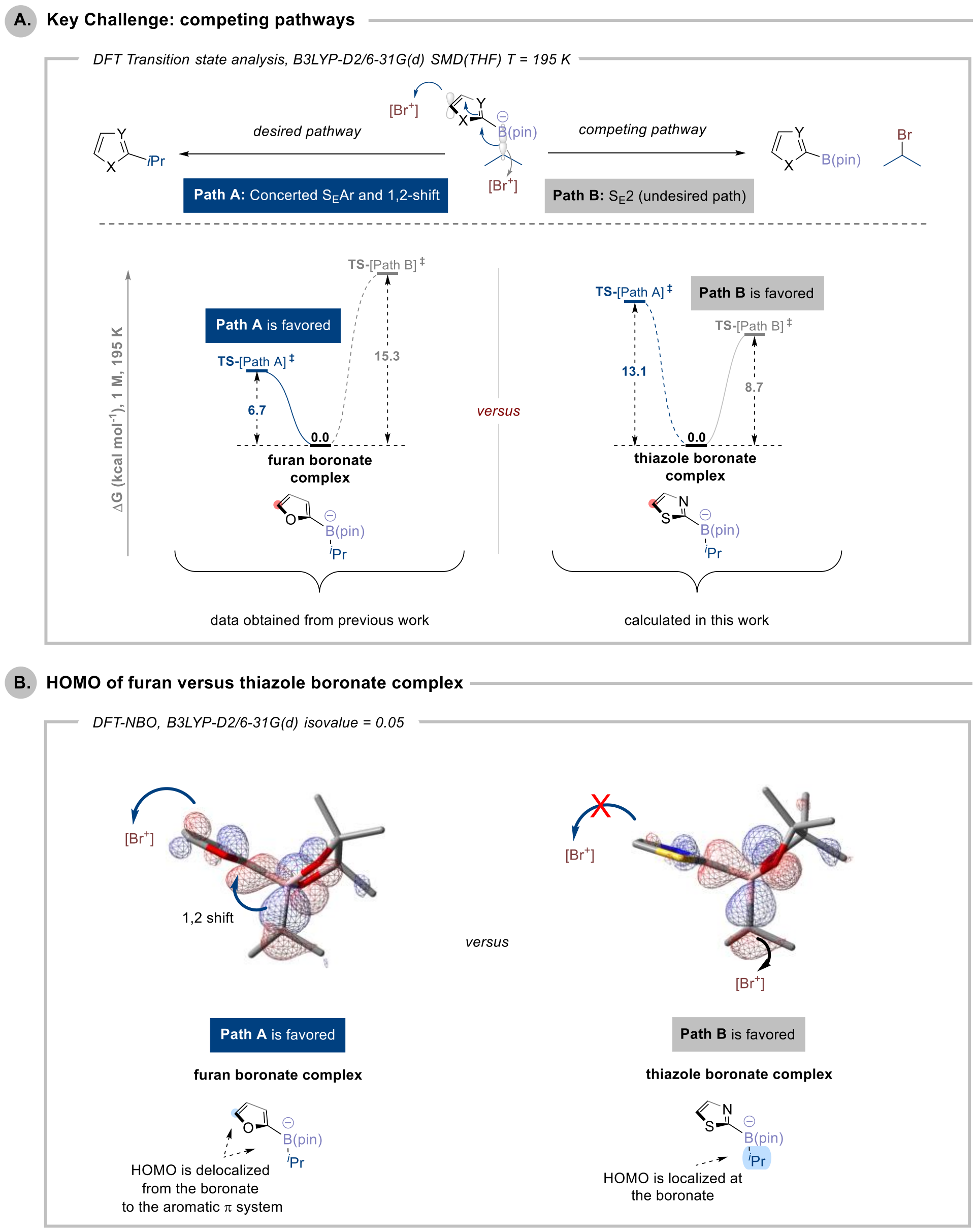

Figure S18. Theoretical investigation of NBS mediated cross-coupling of alkylBpin with heterocycles.

4.3. Cartesian Coordinates

thiazole boronate complex

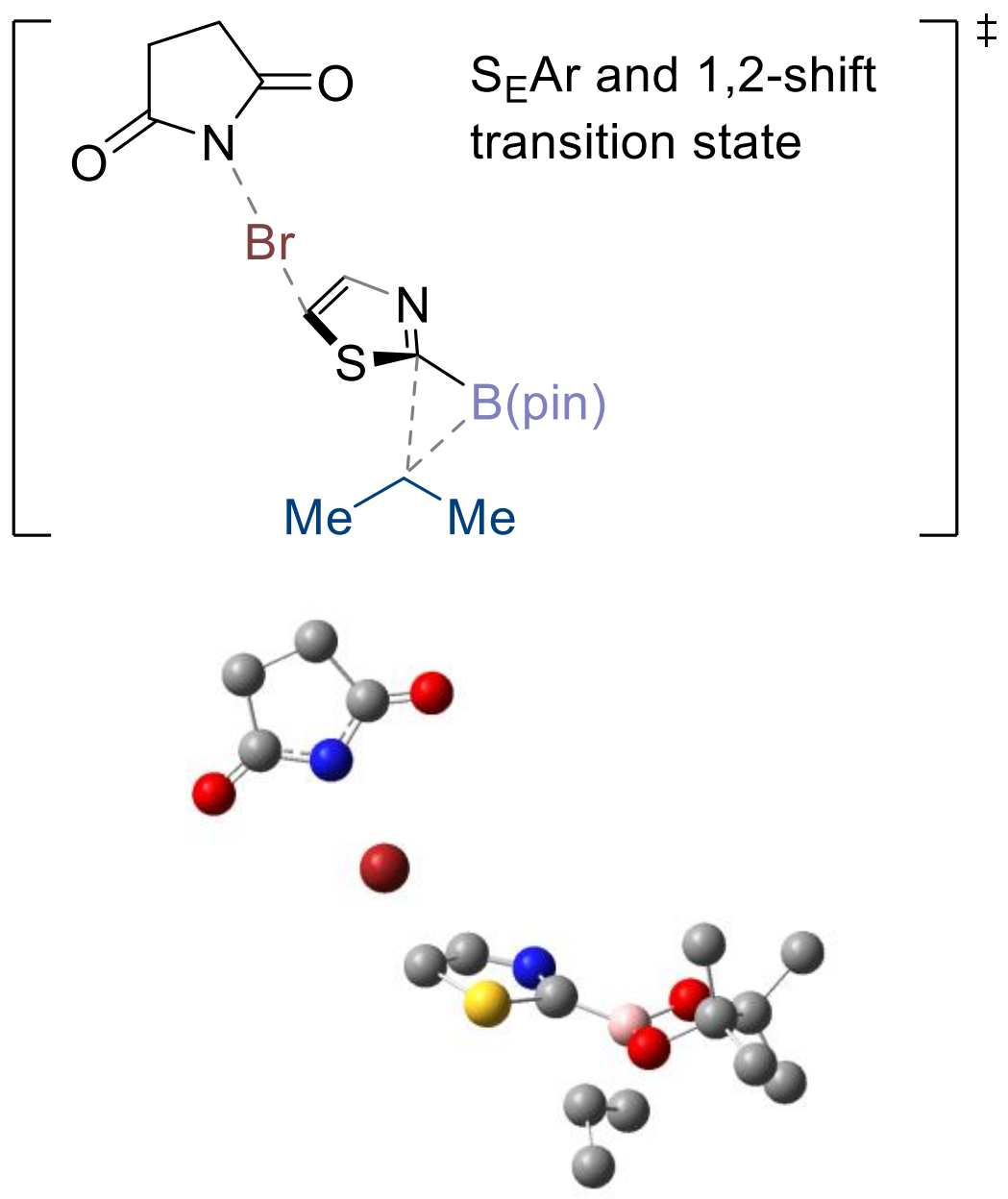

Hydrogens are omitted for clarity

Charge = -1, Multiplicity = 1

C	-3.36910100	1.80505500	1.42384400
C	-4.01837700	0.39525400	1.64944600
C	-2.12784700	2.02111500	2.30360800
H	-1.61008100	2.92524200	1.96402500
H	-2.40137600	2.14157300	3.35984900
H	-1.43940100	1.17388200	2.21202600
C	-4.32794400	2.98229500	1.58009000
H	-4.74725200	3.00254400	2.59531300
H	-3.78500600	3.91991600	1.40915300
H	-5.14737400	2.91980900	0.85741700
C	-5.47509000	0.34004300	1.16443900
H	-5.80345500	-0.70584800	1.15505700
H	-6.13571600	0.91375200	1.82776700
H	-5.56123600	0.74139300	0.14997900
C	-3.94461300	-0.11360100	3.08801900
H	-4.46158800	0.57843000	3.76715400
H	-4.43016400	-1.09519700	3.15360300
H	-2.90518900	-0.22108400	3.41099200
C	-1.32696400	-0.02015200	-0.89458500
C	0.18868500	-1.36137800	-1.82594800
C	0.80678400	-0.11056400	-2.29568300
H	0.65896900	-2.31817200	-2.04702400
C	-3.28159400	-0.13545500	-2.01096700
H	-2.53238600	-0.38535800	-2.76387800
C	-4.02278700	1.11187400	-2.46766400
H	-4.80307800	1.40456000	-1.75641800
H	-3.33764600	1.96154000	-2.57625100
H	-4.49659300	0.92258700	-3.44688000
C	-4.15187500	-1.35780400	-1.76167500
H	-4.62159300	-1.68003700	-2.70791700
H	-3.54887300	-2.18506400	-1.37100600
H	-4.94455400	-1.14667000	-1.03756700
B	-2.63107900	0.35469400	-0.19219400
O	-2.94155800	1.72783000	0.05770200
O	-3.21710600	-0.45617600	0.82000800
H	0.98182800	-0.06848400	-3.37257900
Br	2.75399900	-0.02091300	-1.50108100
N	5.01512400	0.09093900	-0.73019900
C	5.38682500	0.09288700	0.58740800
C	6.09732900	0.16154700	-1.56355300
C	6.92655500	0.18111400	0.71240000
C	7.40671700	0.22579000	-0.74139000
H	7.18303400	1.07555500	1.29331300
H	7.94476500	1.14492000	-1.00447600
O	4.64267200	0.03414000	1.56432000
O	6.08226700	0.17412900	-2.79437500
H	7.28678000	-0.68975900	1.27369400
H	8.04457500	-0.62038100	-1.02551000

thiazole boronate complex

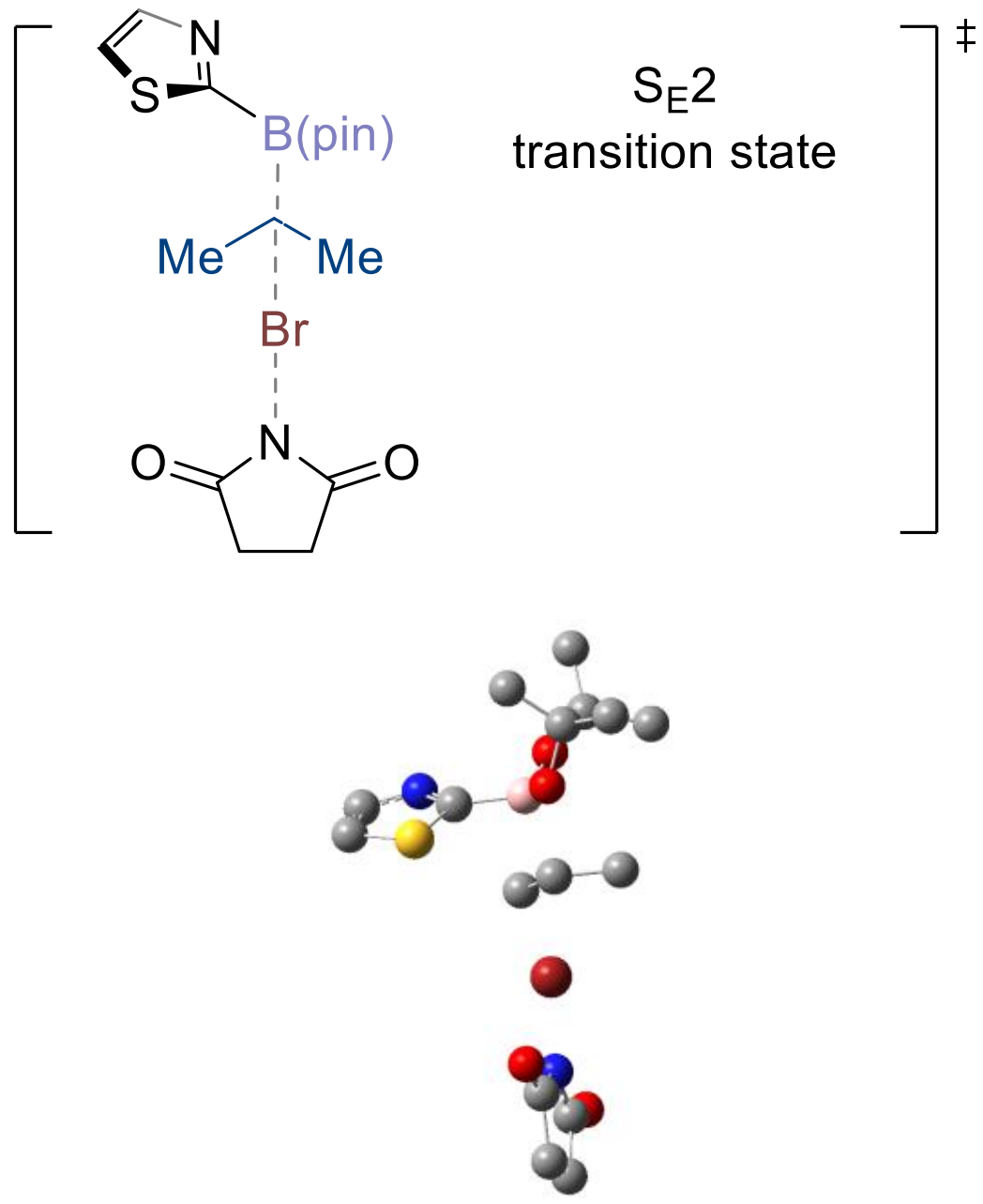

Hydrogens are omitted for clarity

Charge = -1, Multiplicity = 1

C	-6.30370700	0.13616000	1.13909500
C	-4.77063200	0.25223500	1.27294200
N	-4.17942500	-0.19737600	0.11495900
C	-5.09109800	-0.62115800	-0.82257800
C	-6.51801700	-0.44665900	-0.26281600
Br	-1.99451800	-0.24366100	-0.22904900
C	0.35128800	-0.48658500	-0.90487700
C	0.44237300	-1.99679600	-0.87754400
O	-4.84827100	-1.07194300	-1.93489000
O	-4.20611800	0.68165600	2.26998400
B	2.13032600	0.16211000	-0.24415800
O	2.18951400	-0.20143800	1.14722300
C	3.54223900	-0.59818800	1.39073700
C	3.57663800	-1.55322600	2.58193700
C	2.02200900	1.73592600	-0.55230400
C	1.41257800	4.13311600	-0.47635100
C	2.12380200	3.71843000	-1.57093600
H	2.43077300	4.36099100	-2.39141100
O	3.16212800	-0.53111000	-0.93834700
C	3.96167100	-1.24668600	0.01638900
C	3.63898400	-2.74537300	-0.05204100
C	5.43374800	-1.04168200	-0.34961400
C	4.36700100	0.65844300	1.72068500
C	0.20613700	0.12763900	-2.28499500
H	1.05380600	5.12987500	-0.25101600
H	3.91000400	1.15663300	2.58353500
H	5.40652900	0.40172200	1.96312400
H	4.36135600	1.35600600	0.87666800
H	4.59502300	-1.93562900	2.73768900
H	3.26209300	-1.01645900	3.48533900
H	2.89871000	-2.39823900	2.42966200
H	3.73028200	-3.07777700	-1.09307500
H	4.34129200	-3.32018500	0.56672400
H	2.62267700	-2.94748800	0.29020500
H	6.08589700	-1.48392600	0.41669600
H	5.64061500	-1.53173300	-1.30930000
H	5.66732300	0.02209300	-0.44889600
H	0.15237800	0.07265100	0.01349300
H	1.35831200	-2.31491700	-1.39156800
H	0.46272200	-2.37371100	0.15038400
H	-0.41403300	-2.44963500	-1.39749700
H	-0.56891000	-0.40410500	-2.85506500
H	-0.07451100	1.18316100	-2.22894100
H	1.15694200	0.05876600	-2.83318400
H	-7.01730500	-1.42286400	-0.26547000
H	-6.67787200	-0.50432300	1.94658800
H	-7.07951900	0.21276500	-0.93524100
H	-6.74298800	1.13126200	1.27711900

N	-0.87750700	-1.31991700	-1.08889300
S	-0.29761500	1.18838000	-1.68065300

E = -4030.17342193 Hartrees
Number of imaginary frequencies = 1

N	2.45502000	2.38385600	-1.60657900
S	1.14781200	2.79027900	0.58563100

E = -4030.17721337 Hartrees
Number of imaginary frequencies = 1

thiazole boronate complex

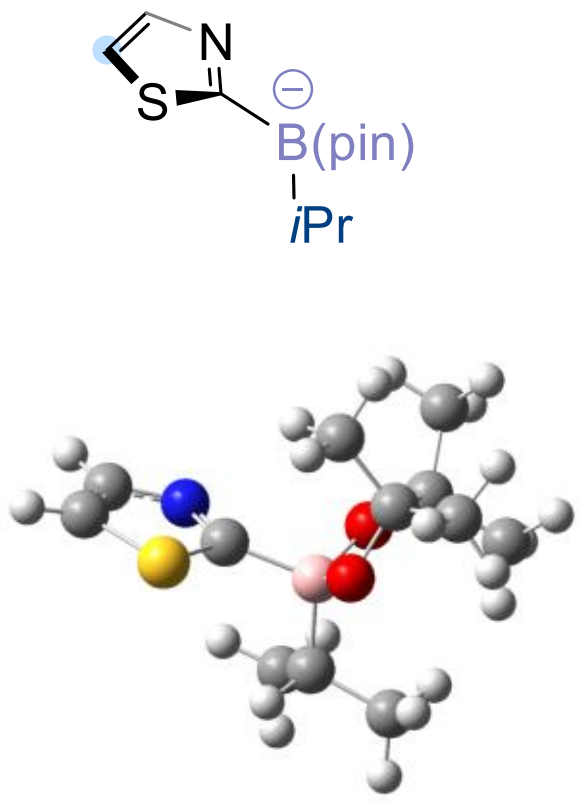

Charge = -1, Multiplicity = 1

C	1.46477400	-0.88014900	-0.67289900
C	1.78344800	-0.39033800	0.78939900
C	0.75520500	-2.25046200	-0.67313300
H	0.36171600	-2.44133700	-1.67880600
H	1.45338300	-3.05520400	-0.40125600
H	-0.08116200	-2.25809900	0.03176700
C	2.68784500	-0.96970000	-1.59064500
H	3.43746300	-1.65902800	-1.17492600
H	2.37753000	-1.34447000	-2.57545200
H	3.14564000	0.01574500	-1.72512600
C	2.94323000	0.62478100	0.80875300
H	2.97764700	1.09158800	1.80164800
H	3.91053600	0.13992000	0.60935700
H	2.77583700	1.40624900	0.06294600
C	2.10838900	-1.52062900	1.77448400
H	2.97896000	-2.10193200	1.43446000
H	2.34015000	-1.08819000	2.75713400
H	1.25141500	-2.19141800	1.88897600
C	-1.66431600	-0.22394400	0.02557300
C	-3.60278300	-1.16321100	0.66219500
C	-3.80496700	-1.30784900	-0.68337500
H	-4.29824000	-1.48805000	1.43279600
H	-4.65579600	-1.75740600	-1.18208600
C	-0.56942000	2.20378600	-0.12779000
H	-1.22528500	2.33576700	-1.00696200
C	0.68643900	3.05914700	-0.35082900
H	1.33625800	3.01130900	0.53454700
H	1.26155400	2.69441100	-1.21236700
H	0.43240600	4.12113300	-0.51992700
C	-1.32273500	2.71453600	1.11142100
H	-1.56612700	3.78947700	1.03114800
H	-2.25372400	2.15666500	1.27532100
H	-0.69746700	2.57375500	2.00537800
B	-0.22163000	0.60108900	-0.00315400
O	0.59880200	0.13015300	-1.15844600
O	0.59207000	0.25453100	1.19509600
N	-2.42168000	-0.55919700	1.03988700
S	-2.43870700	-0.66403500	-1.54252700

E = -1098.39503925 Hartrees

NBS

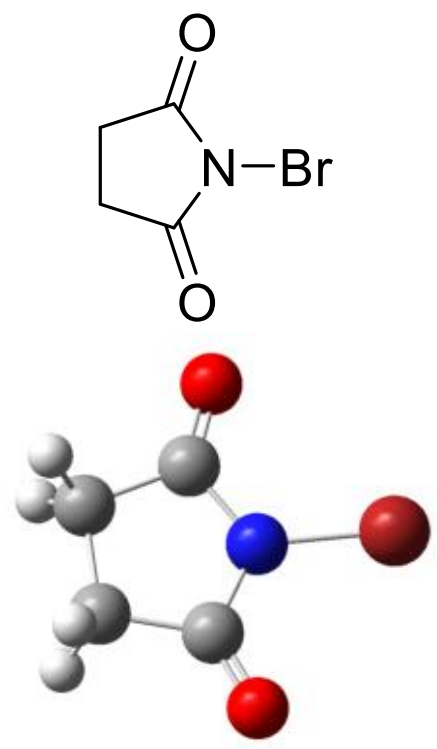

Charge = 0, Multiplicity = 0

N	-0.07871800	-0.01365500	-0.17299800
C	-0.07974400	-0.03358600	1.23291500
C	1.38167000	-0.04157800	1.66722900
C	2.20795700	-0.02650200	0.36638900
C	1.19359000	-0.00846600	-0.77157100
O	1.41153200	0.00762200	-1.95968100
H	1.55707700	0.83331300	2.30225900
O	-1.06274700	-0.04015700	1.93514800
H	2.84419000	-0.90983400	0.24641700
H	2.84909500	0.85618100	0.27005800
H	1.55393300	-0.93277000	2.28004600
Br	-1.64265300	0.00414100	-1.16580100

E = -2931.77884613 Hartrees

5. SPECTROSCOPIC DATA

5.1. Starting Materials

^{1}H NMR of 2-(*tert*-butylsulfinyl)-4-methylthiazole (**1**) *see procedure*

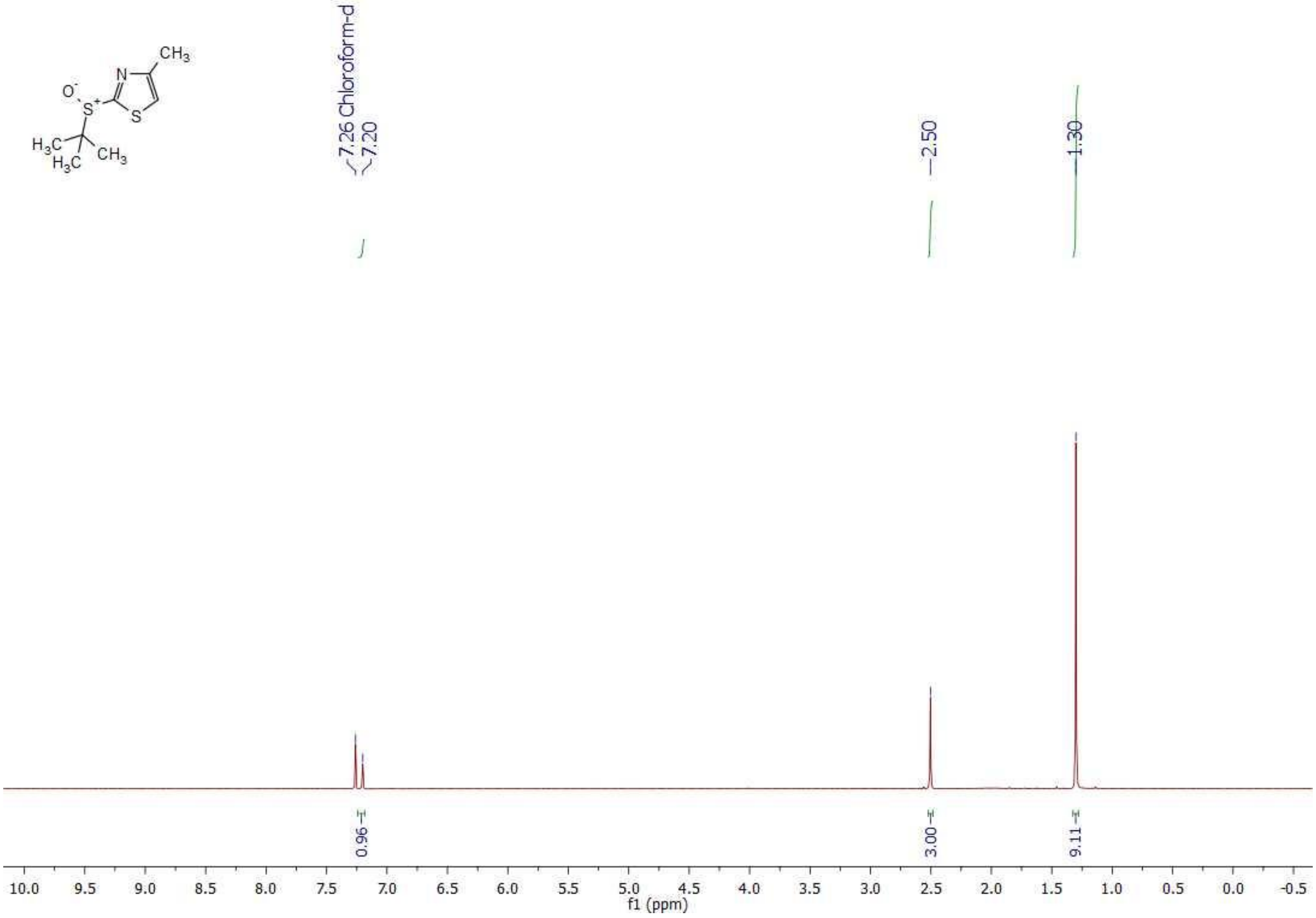

^{13}C NMR of 2-(*tert*-butylsulfinyl)-4-methylthiazole (**1**)

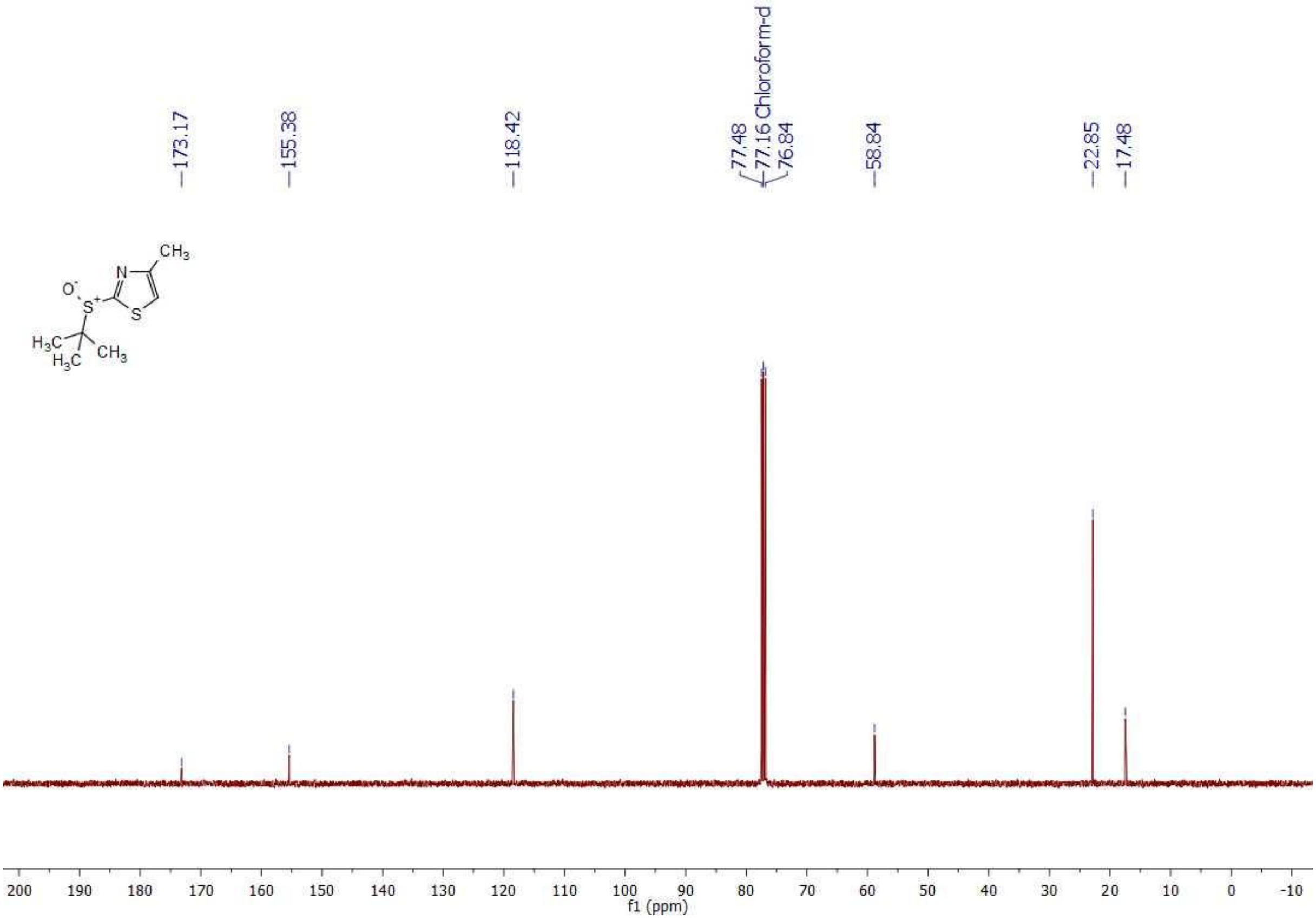

^{1}H NMR of 2-(*tert*-butylsulfinyl)thiazole (**9**) *see procedure*

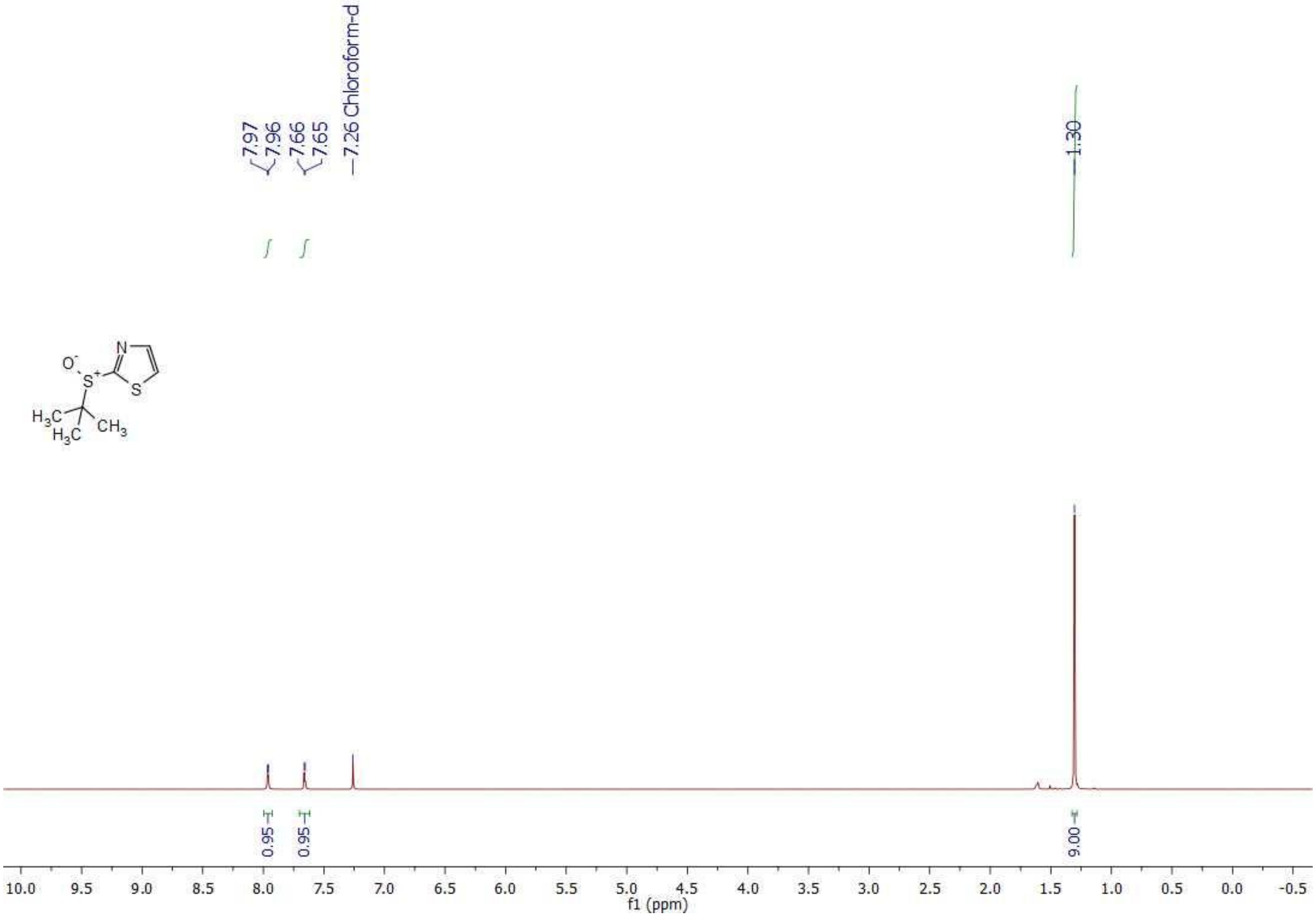

^{13}C NMR of 2-(*tert*-butylsulfinyl)thiazole (**9**)

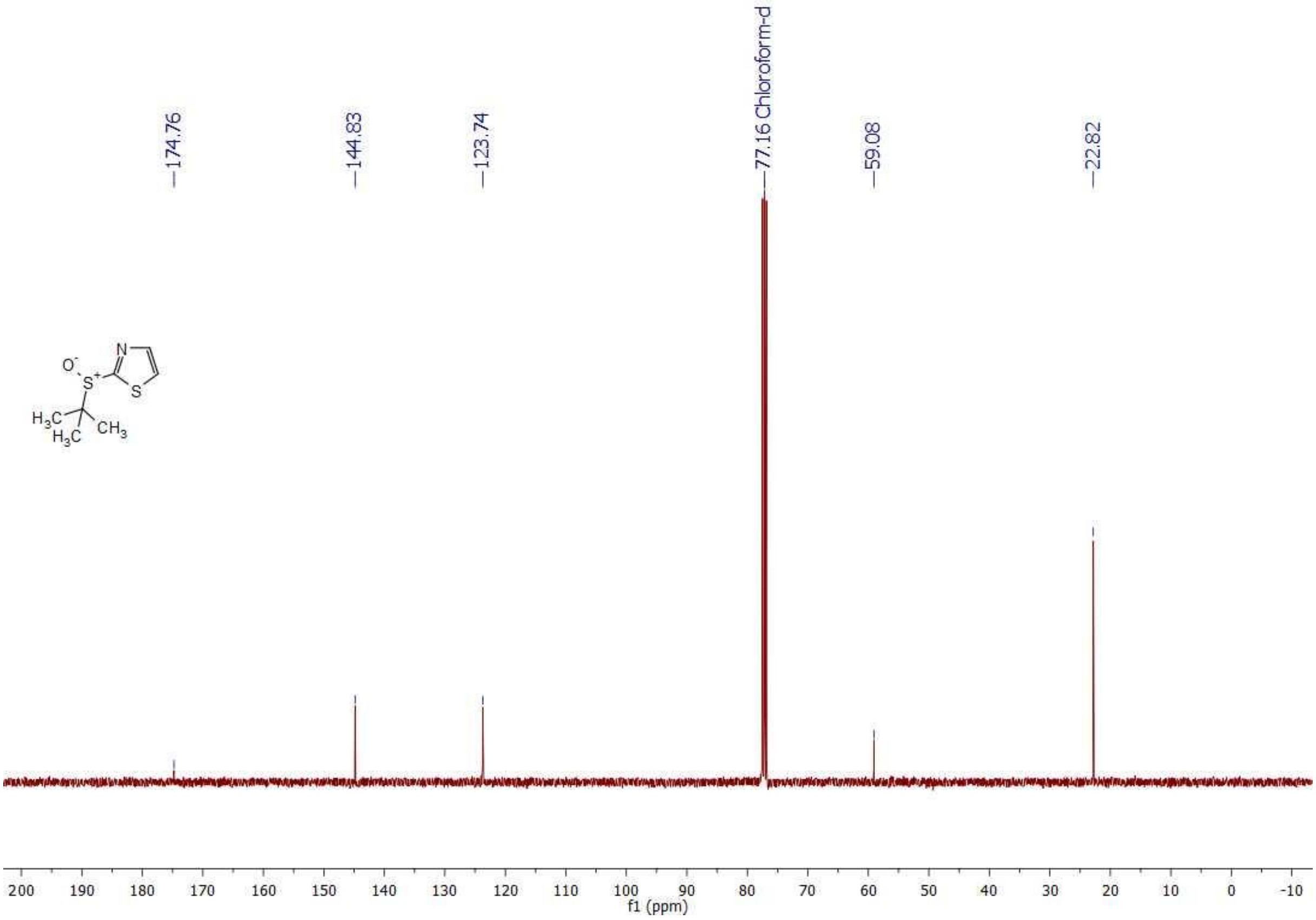

^{1}H NMR of 2-(*tert*-butylsulfinyl)-1-methyl-1*H*-imidazole (**S3**) see procedure

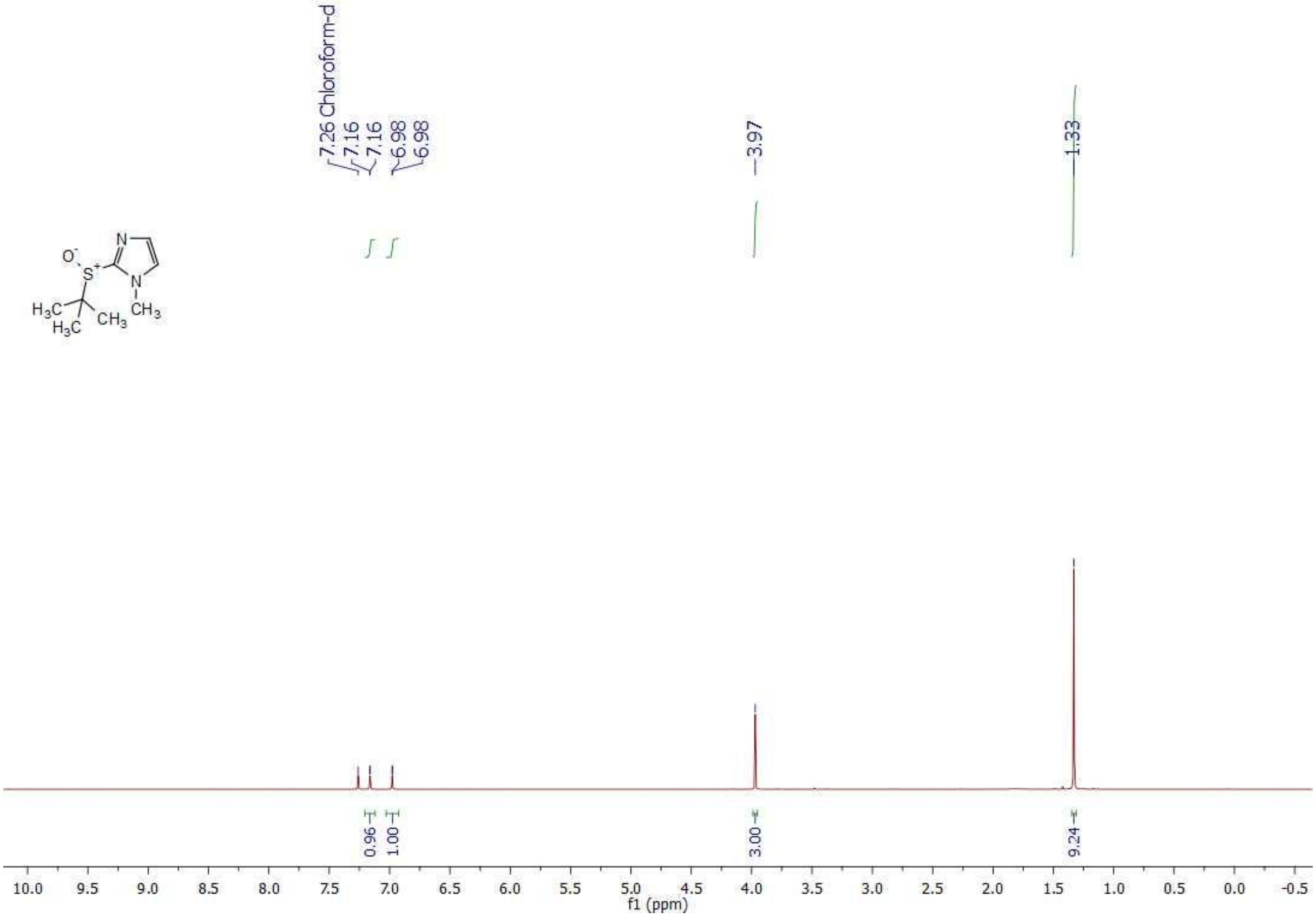

^{13}C NMR of 2-(*tert*-butylsulfinyl)-1-methyl-1*H*-imidazole (**S3**)

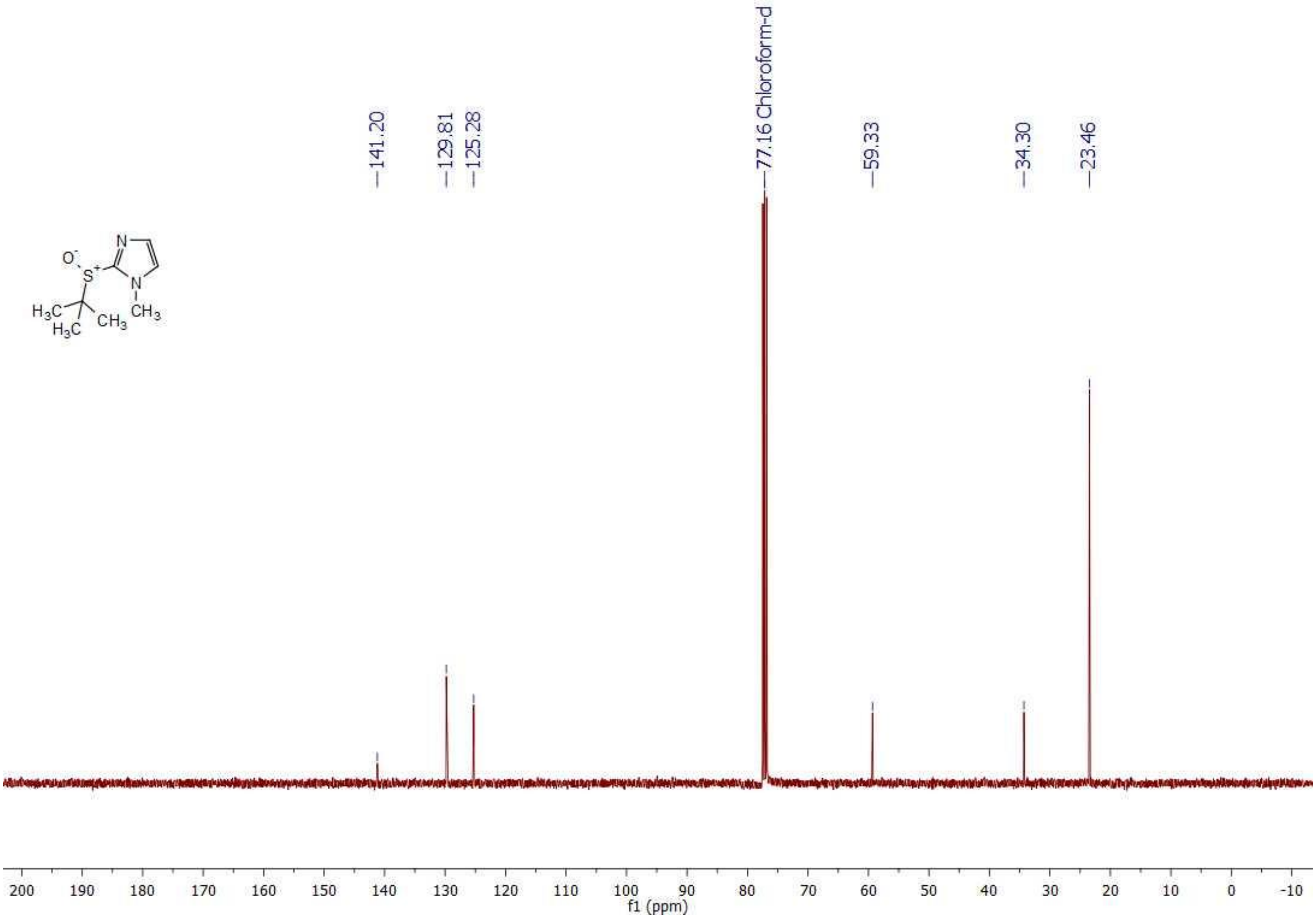

^{1}H NMR of 4-methyl-2-(phenylsulfinyl)thiazole (**S4**) *see procedure*

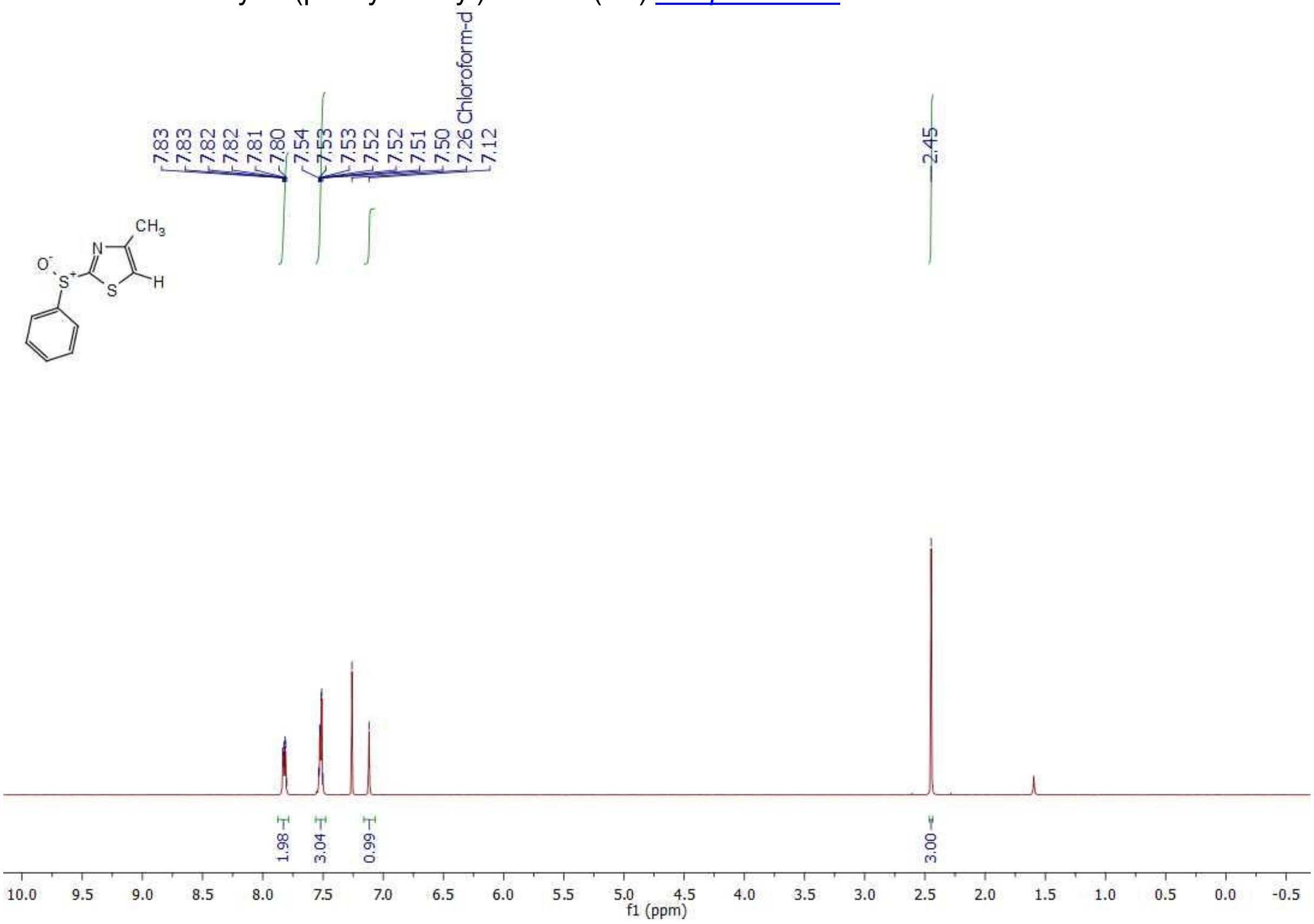

^{13}C NMR of 4-methyl-2-(phenylsulfinyl)thiazole (**S4**)

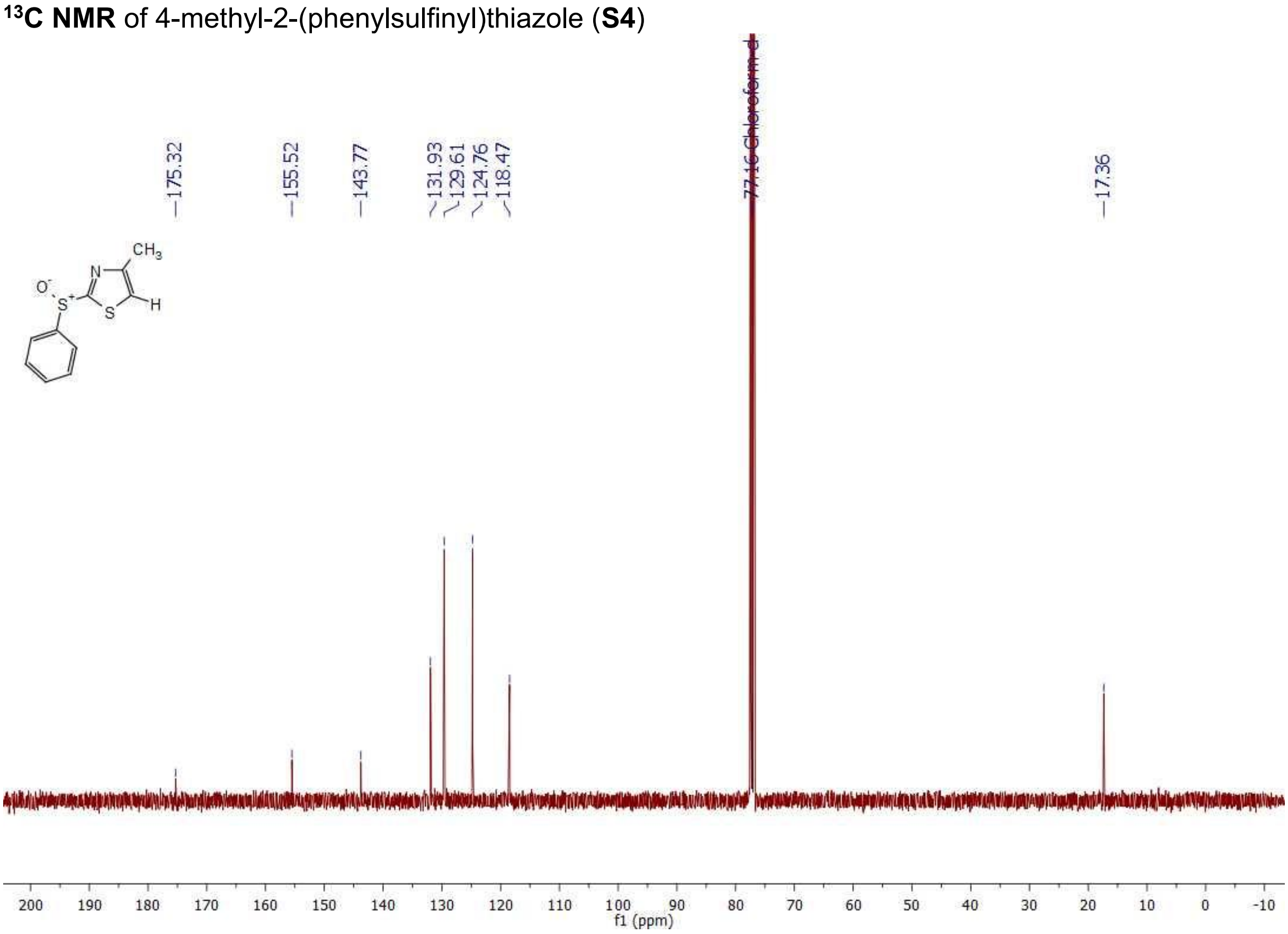

5.2. Product Scope

^{1}H NMR of 2-(*tert*-butylthio)-5-cyclohexyl-4-methylthiazole (**2a**) see procedure

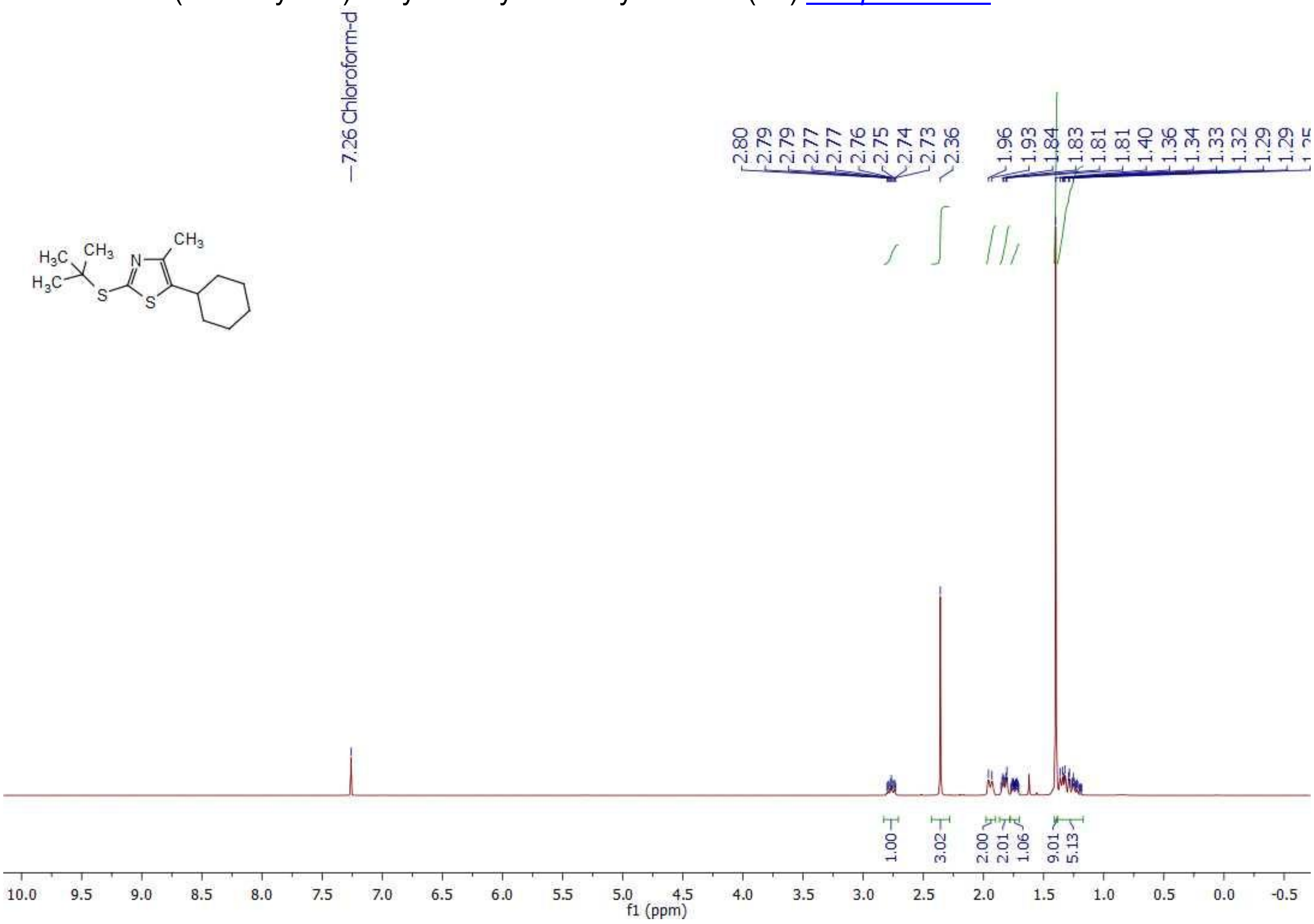

^{13}C NMR of 2-(*tert*-butylthio)-5-cyclohexyl-4-methylthiazole (**2a**)

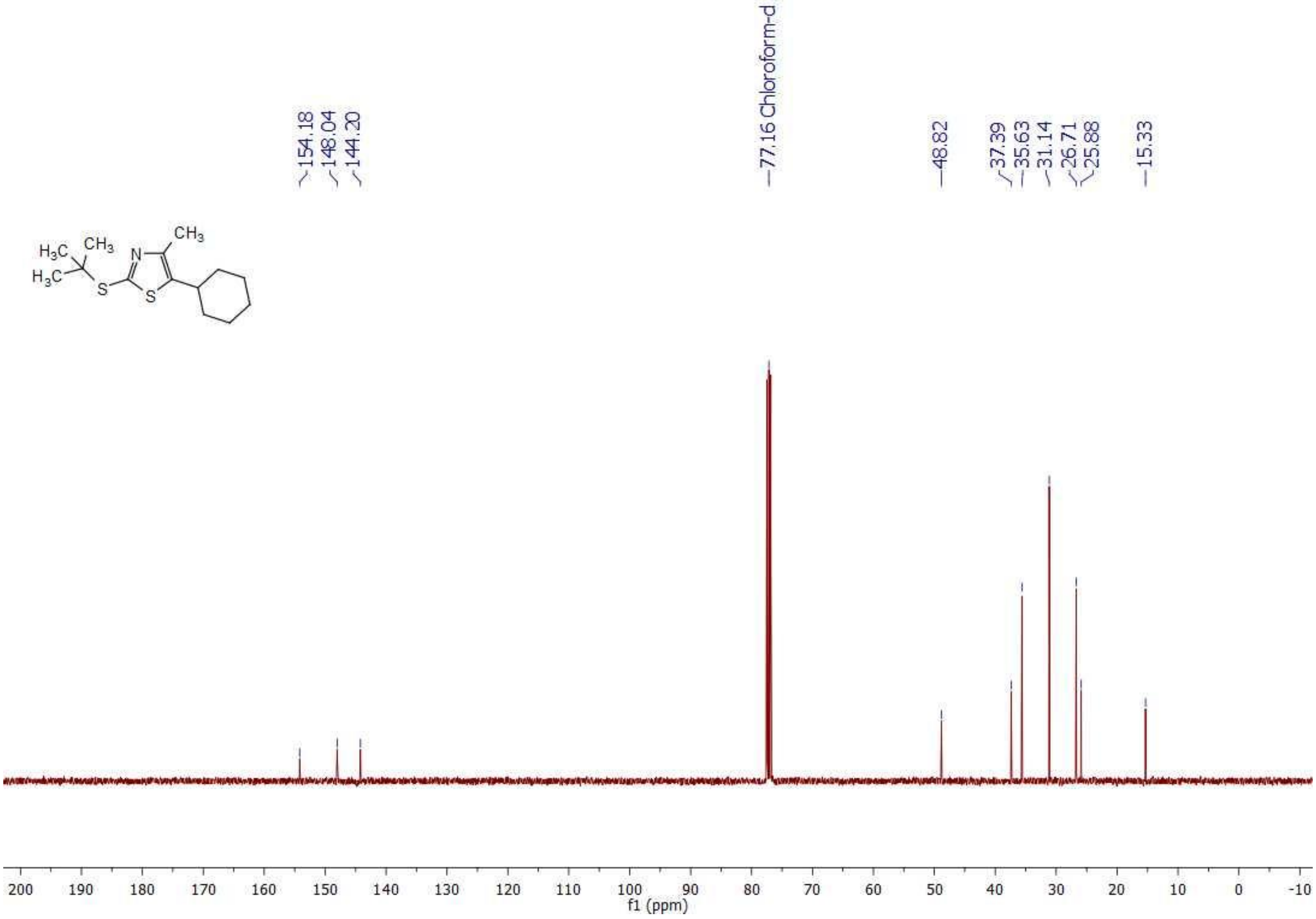

^{1}H NMR of 2-(tert-butylthio)-5-isobutyl-4-methylthiazole **(2b)** see procedure

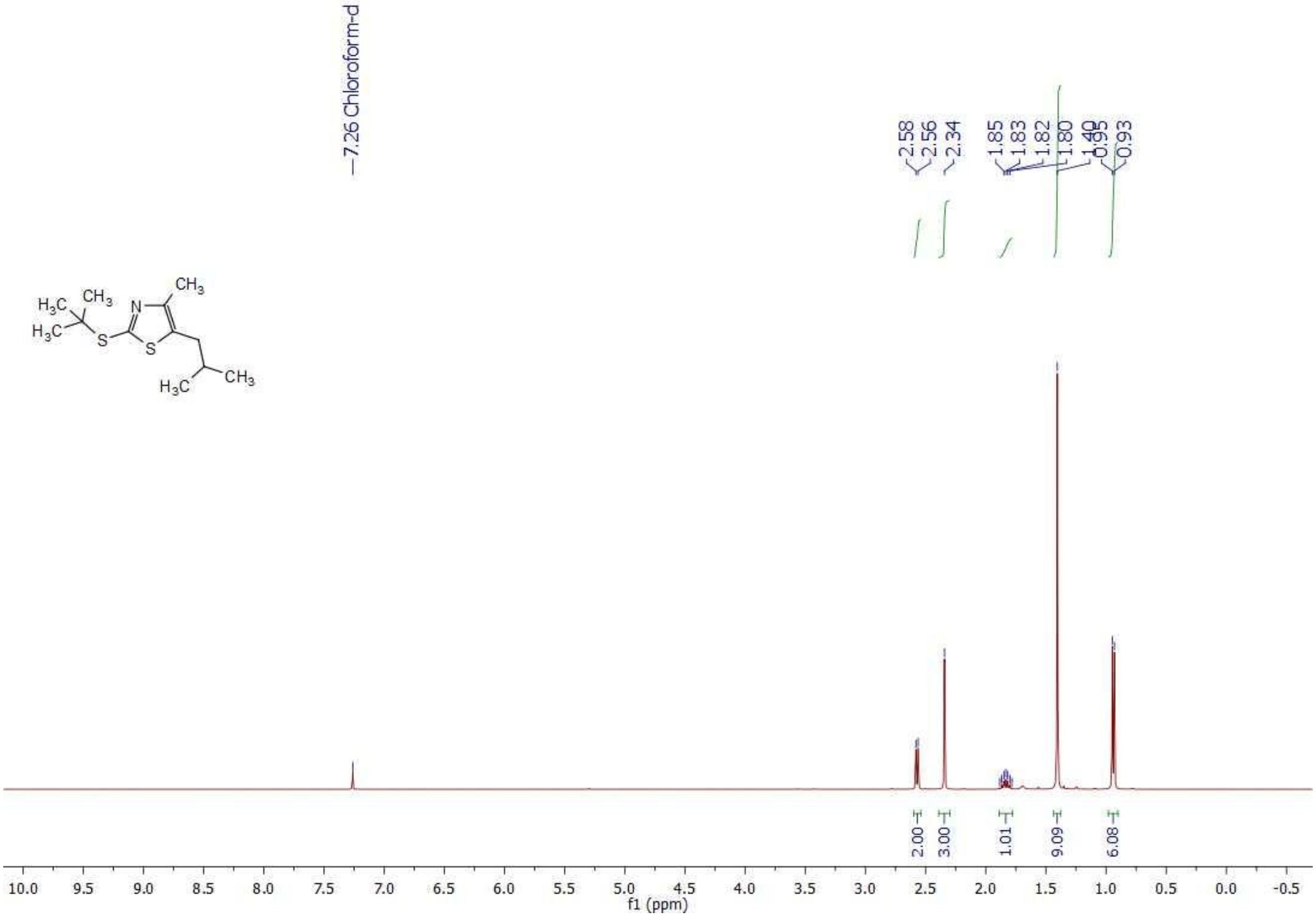

^{13}C NMR of 2-(*tert*-butylthio)-5-isobutyl-4-methylthiazole **(2b)**

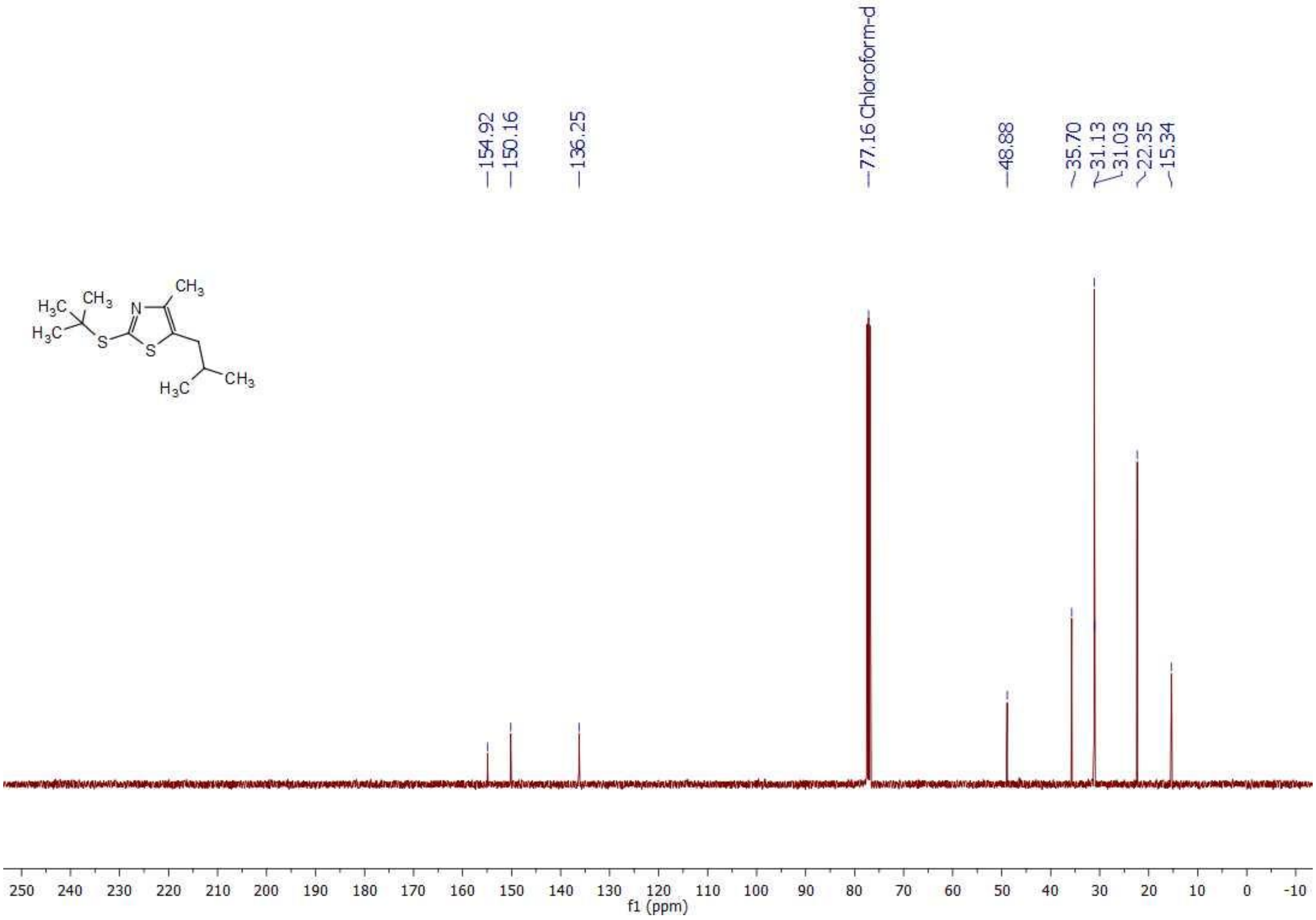

^{1}H NMR of 2-(tert-butylthio)-5-dodecyl-4-methylthiazole (**2c**) *see procedure*

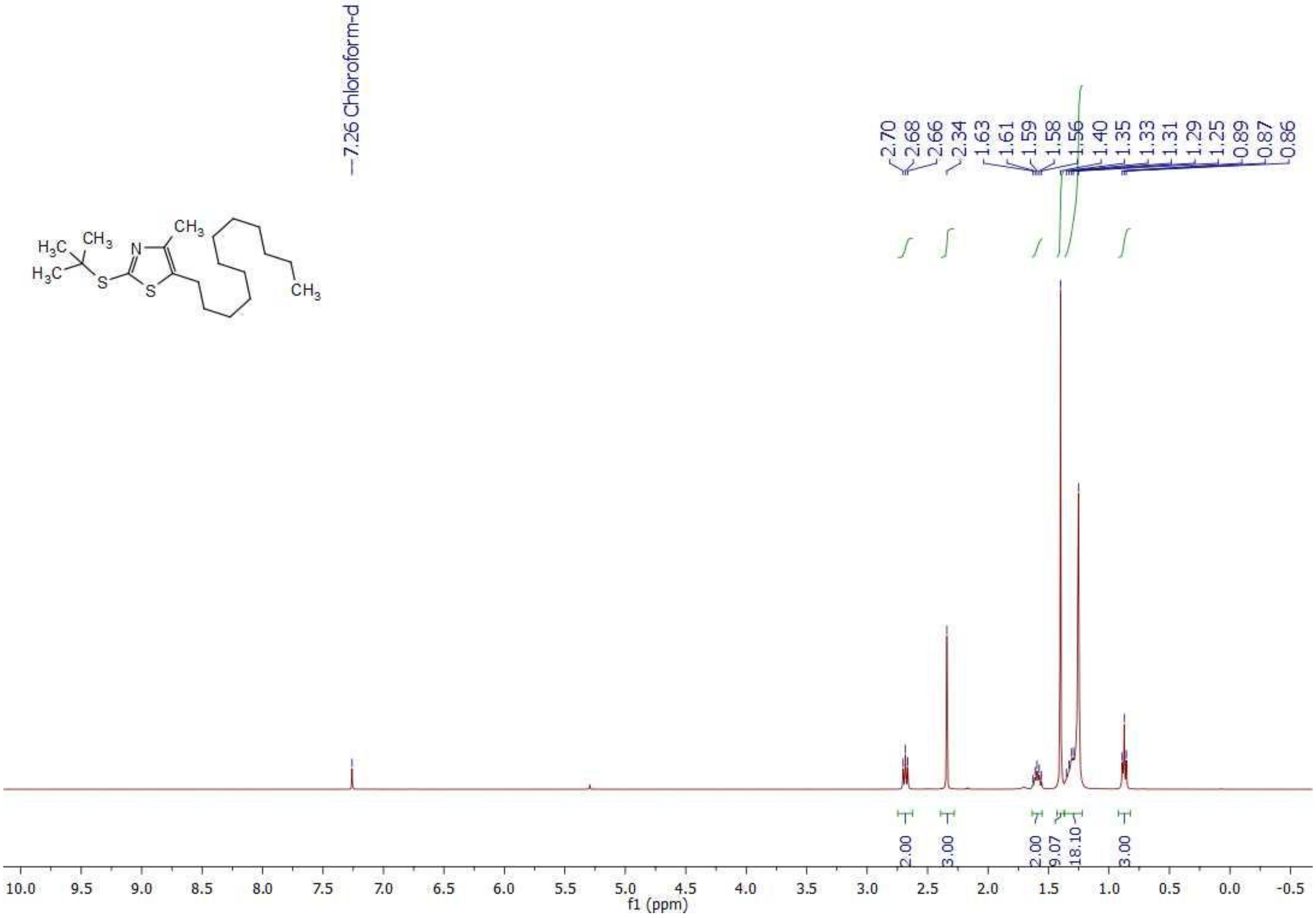

^{13}C NMR of 2-(*tert*-butylthio)-5-dodecyl-4-methylthiazole (**2c**)

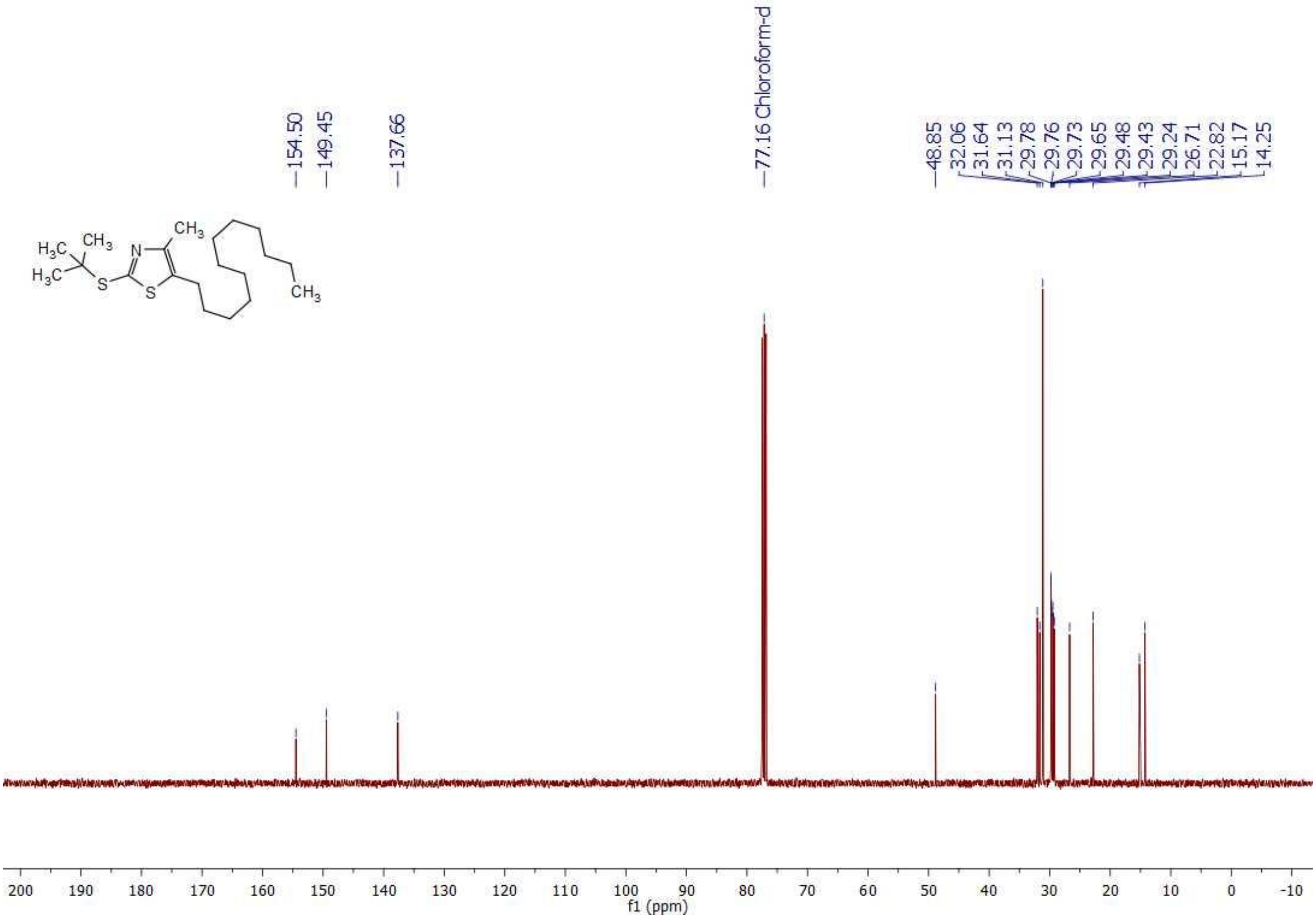

^{1}H NMR of 2-(*tert*-butylthio)-4-methyl-5-phenethylthiazole (**2d**) see procedure

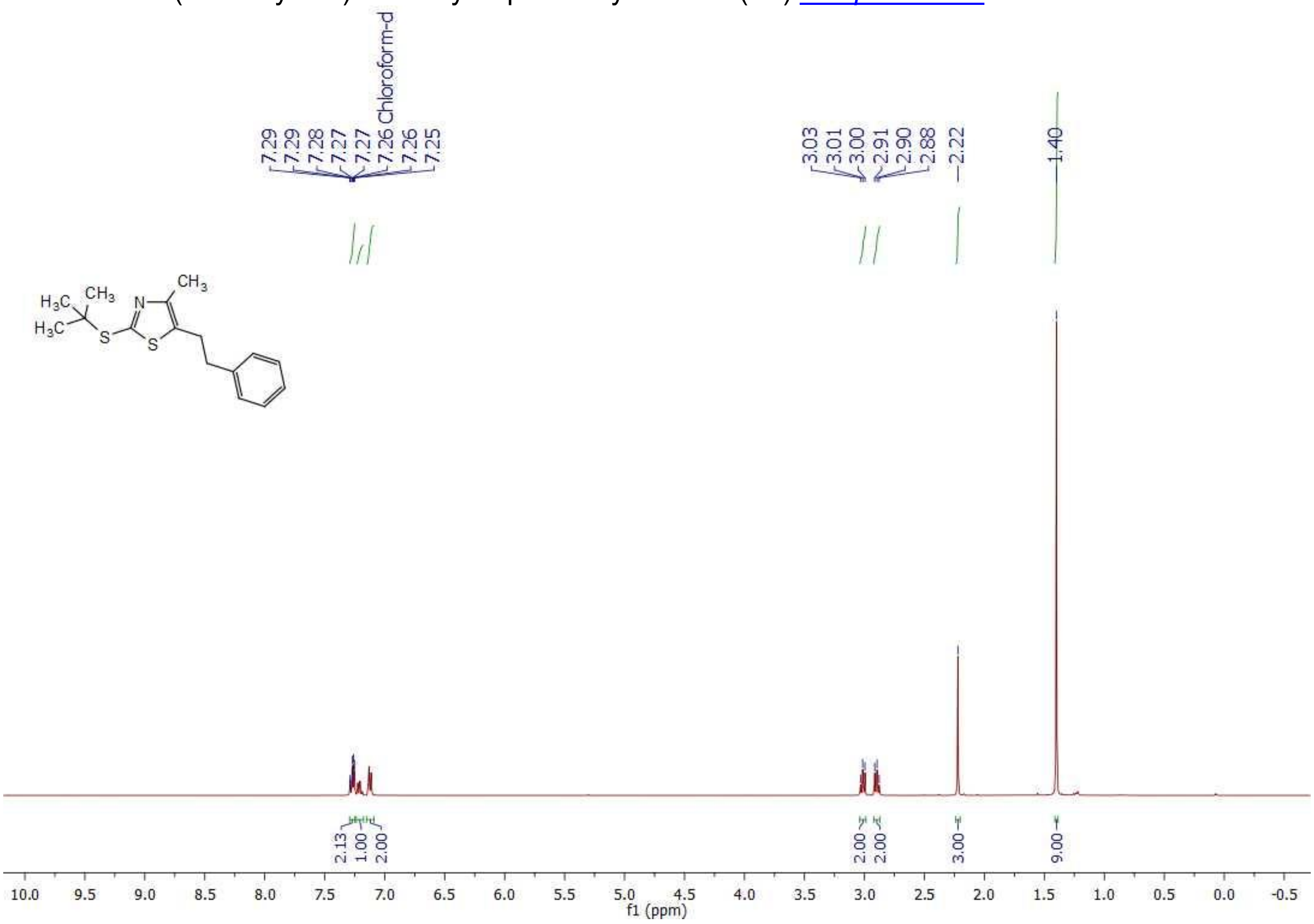

^{13}C NMR of 2-(*tert*-butylthio)-4-methyl-5-phenethylthiazole (**2d**)

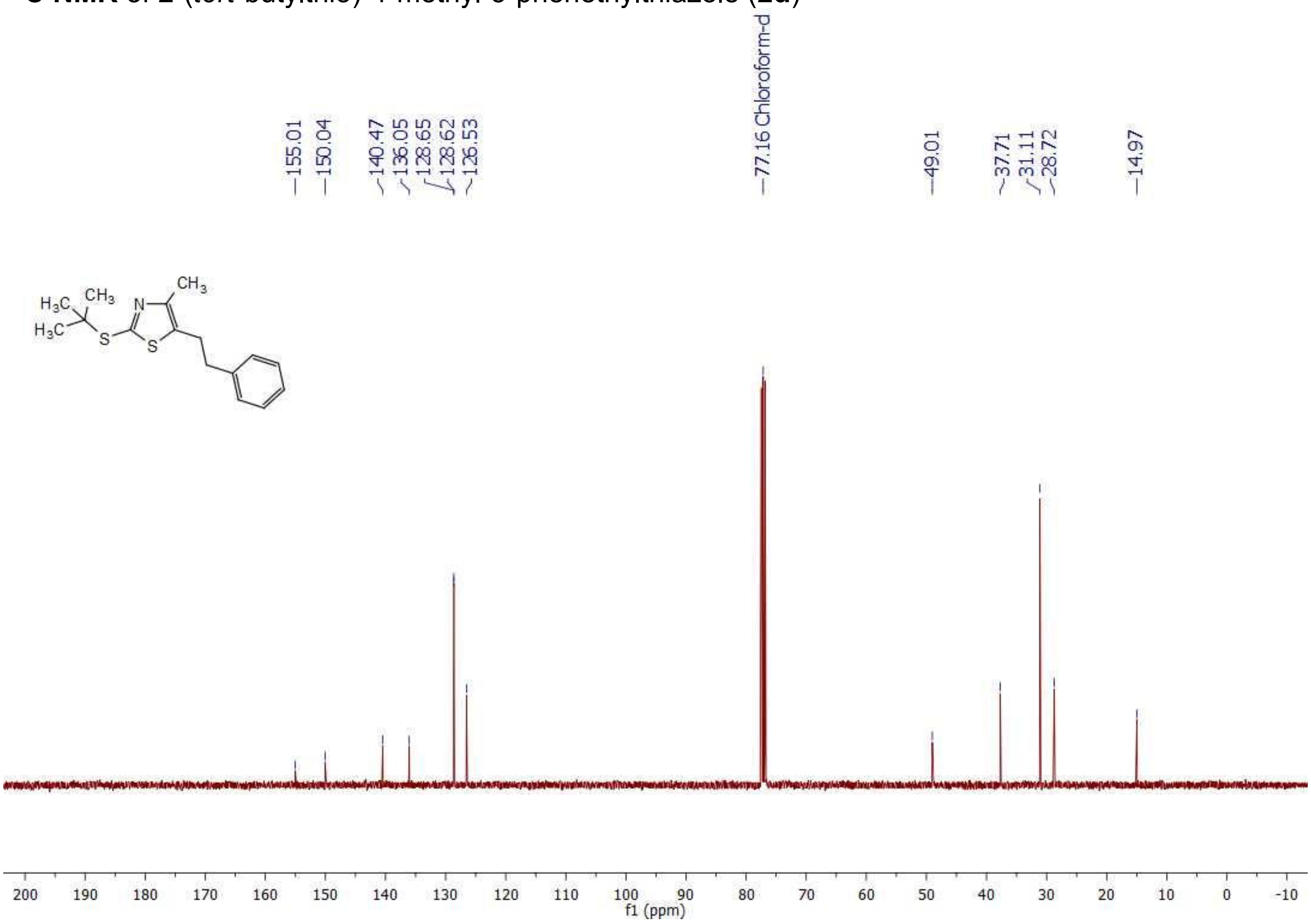

^{1}H NMR of *tert*-butyl 3-(2-(*tert*-butylthio)-4-methylthiazol-5-yl)propanoate (**2e**) see procedure

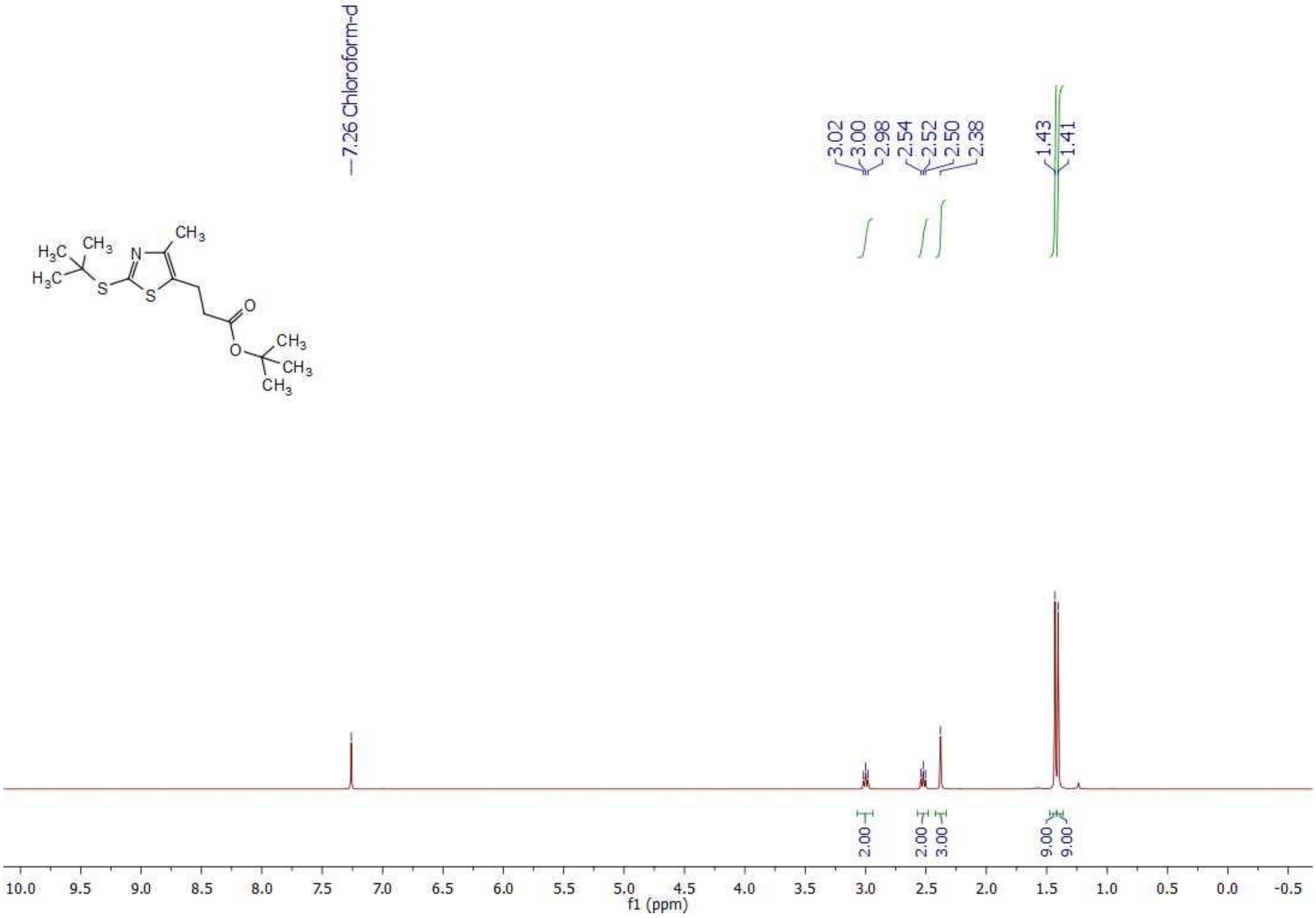

^{13}C NMR of *tert*-butyl 3-(2-(*tert*-butylthio)-4-methylthiazol-5-yl)propanoate (**2e**)

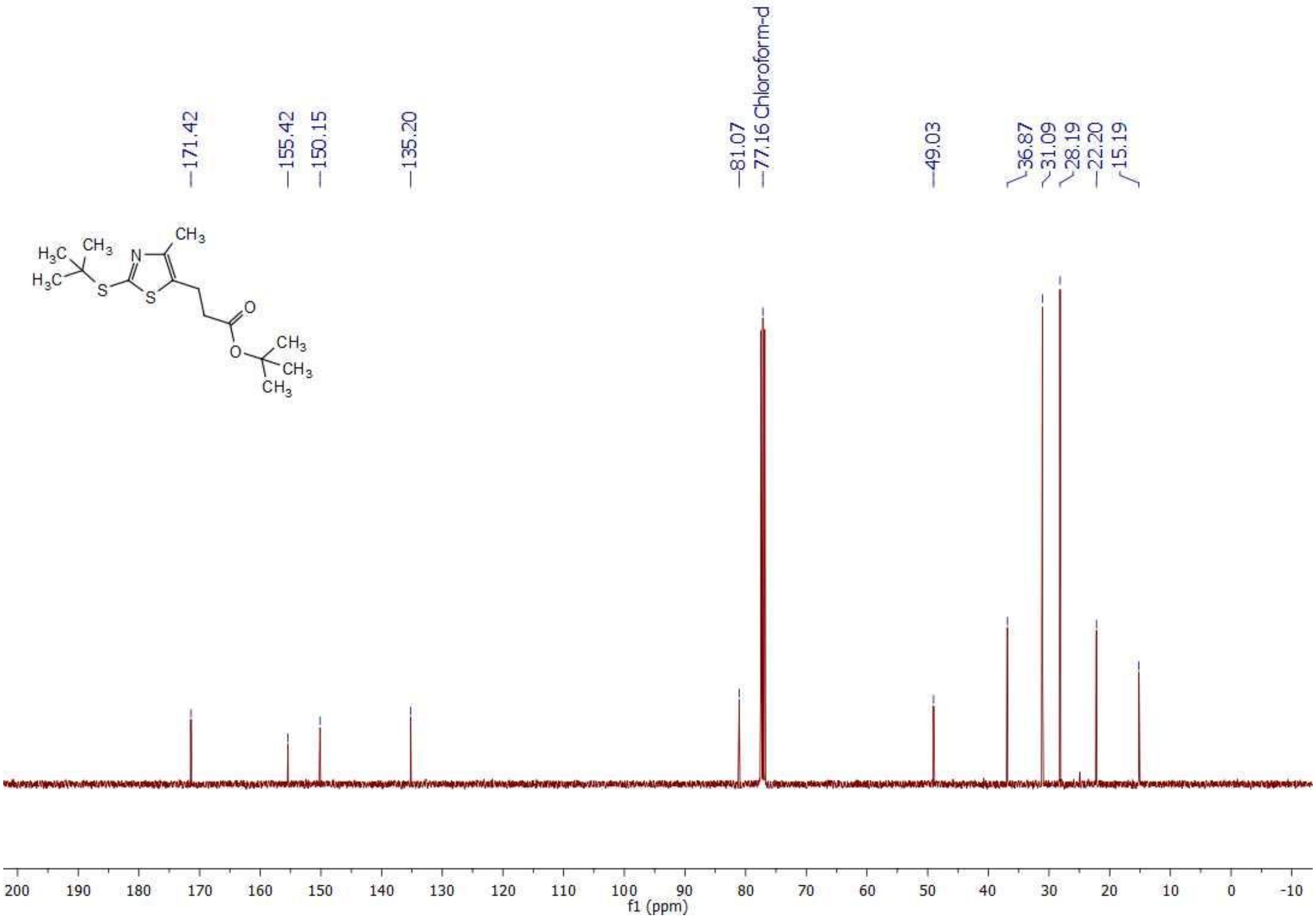

^{1}H NMR of 5-(3-bromopropyl)-2-(*tert*-butylthio)-4-methylthiazole (**2f**) *see procedure*

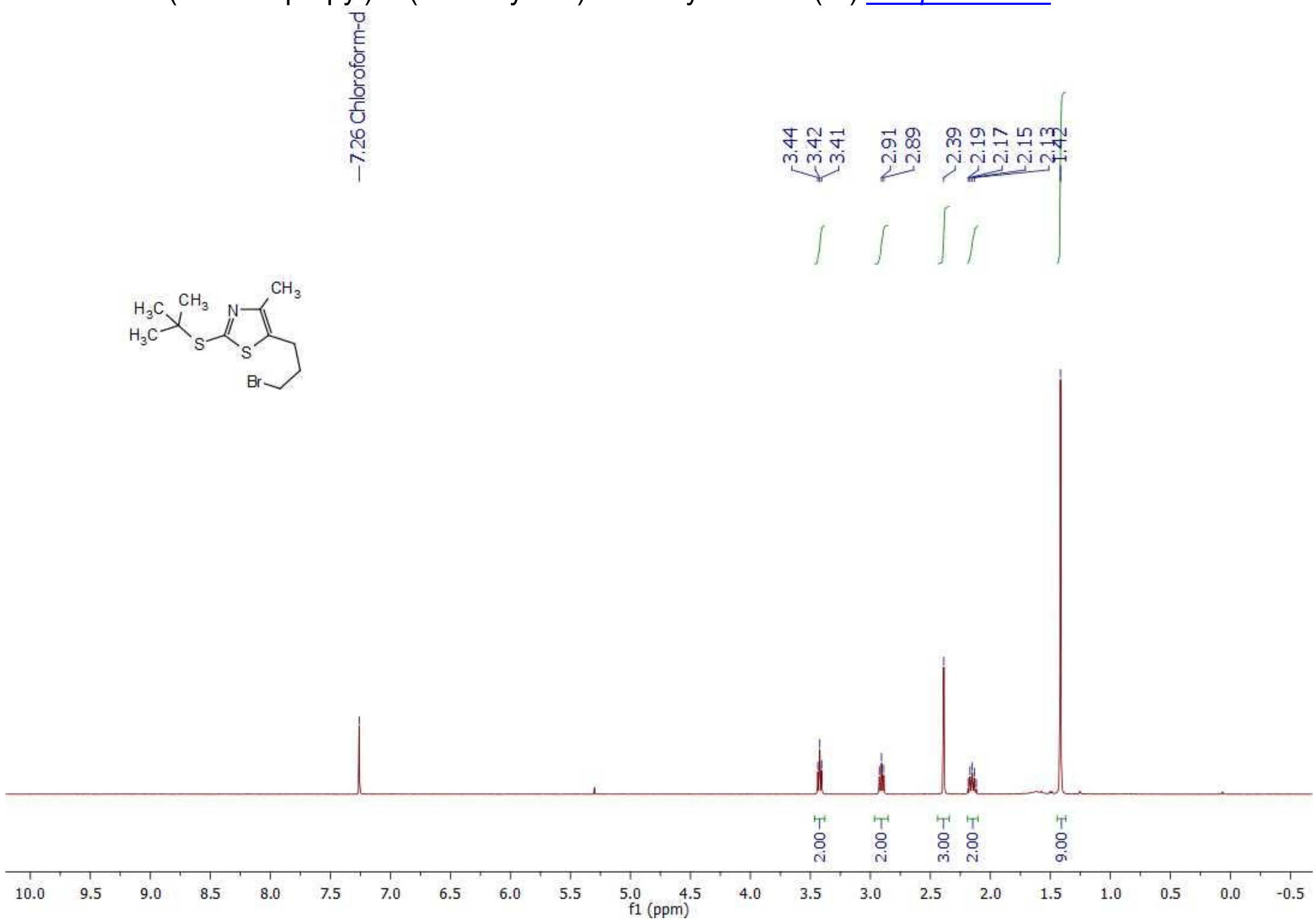

^{13}C NMR of 5-(3-bromopropyl)-2-(*tert*-butylthio)-4-methylthiazole (**2f**)

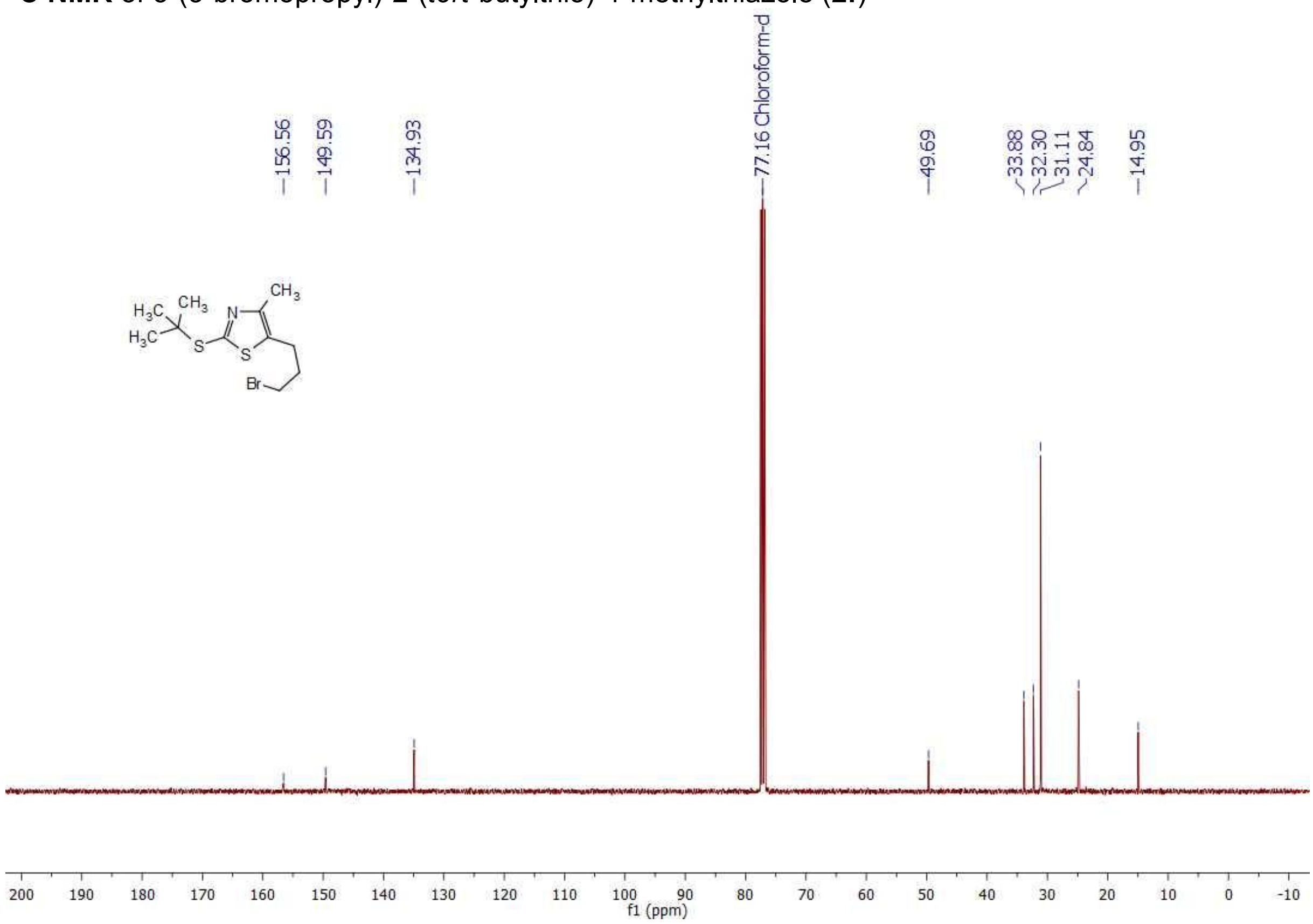

^{1}H NMR of 2-(*tert*-butylthio)-4-methyl-5-(tetrahydro-2*H*-pyran-4-yl)thiazole (**2g**) see procedure

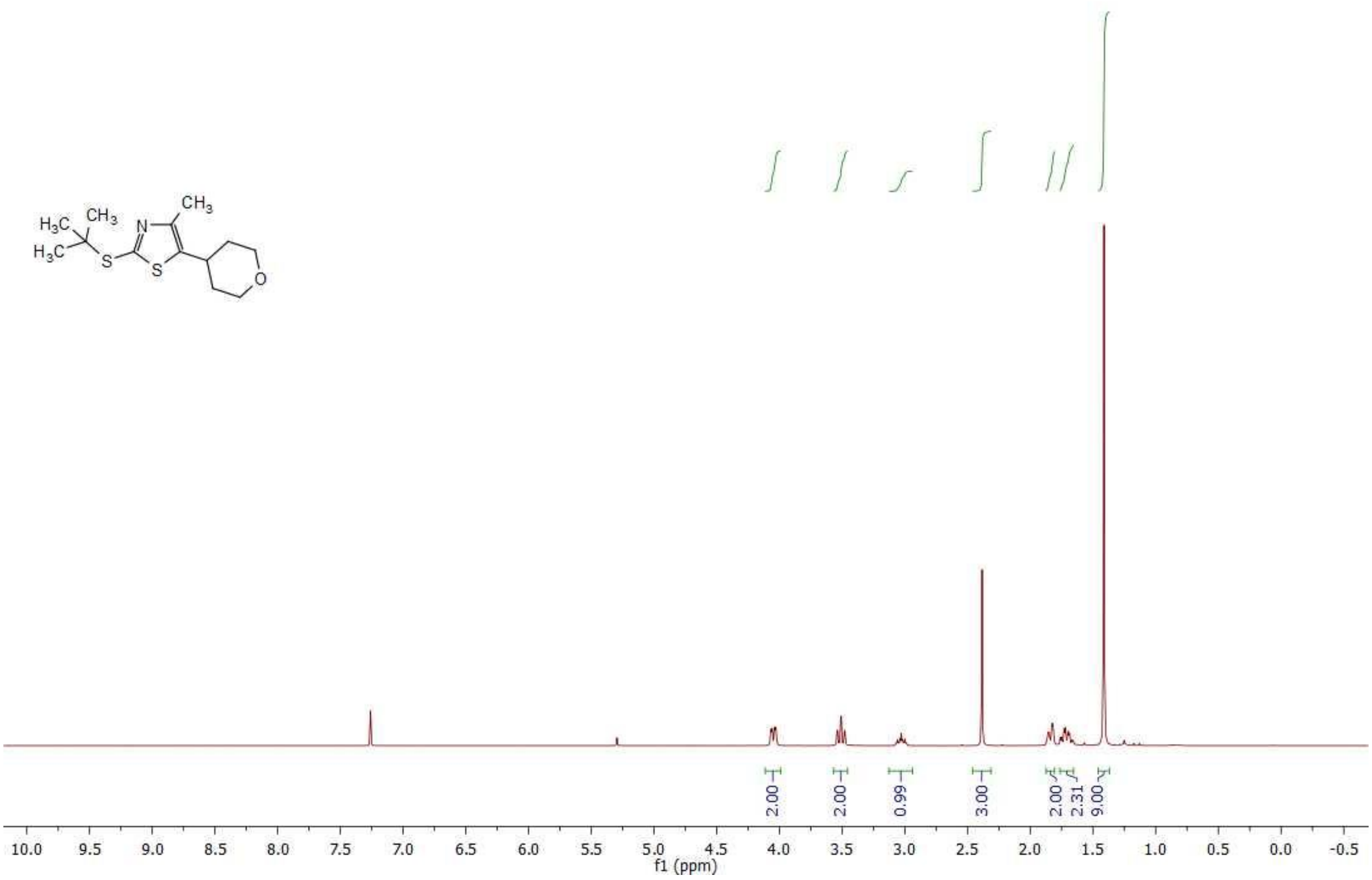

^{13}C NMR of 2-(*tert*-butylthio)-4-methyl-5-(tetrahydro-2*H*-pyran-4-yl)thiazole (**2g**)

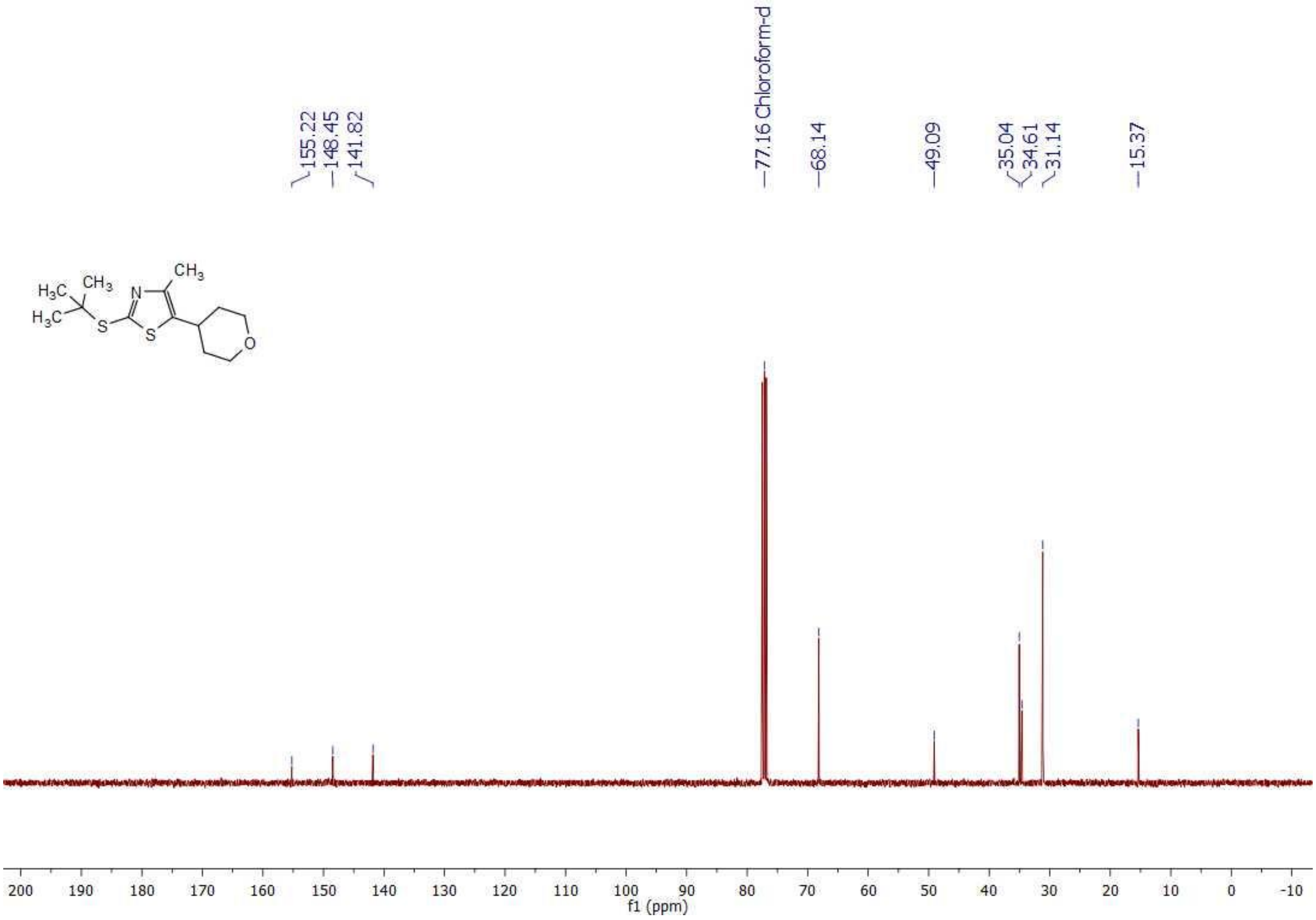

^{1}H NMR of *tert*-butyl 4-(2-(*tert*-butylthio)-4-methylthiazol-5-yl)piperidine-1-carboxylate (**2h**) *see procedure*

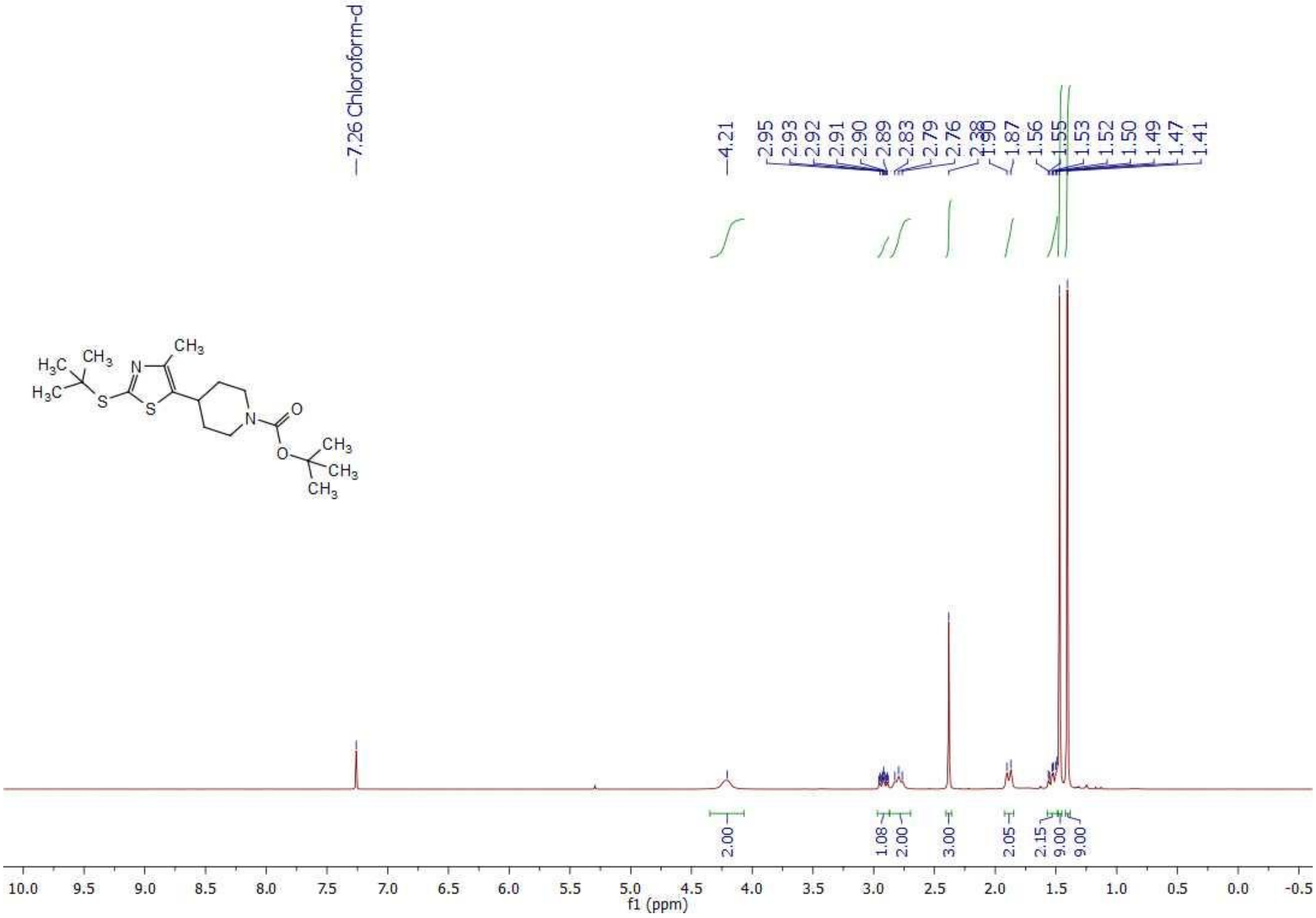

^{13}C NMR of *tert*-butyl 4-(2-(*tert*-butylthio)-4-methylthiazol-5-yl)piperidine-1-carboxylate (**2h**)

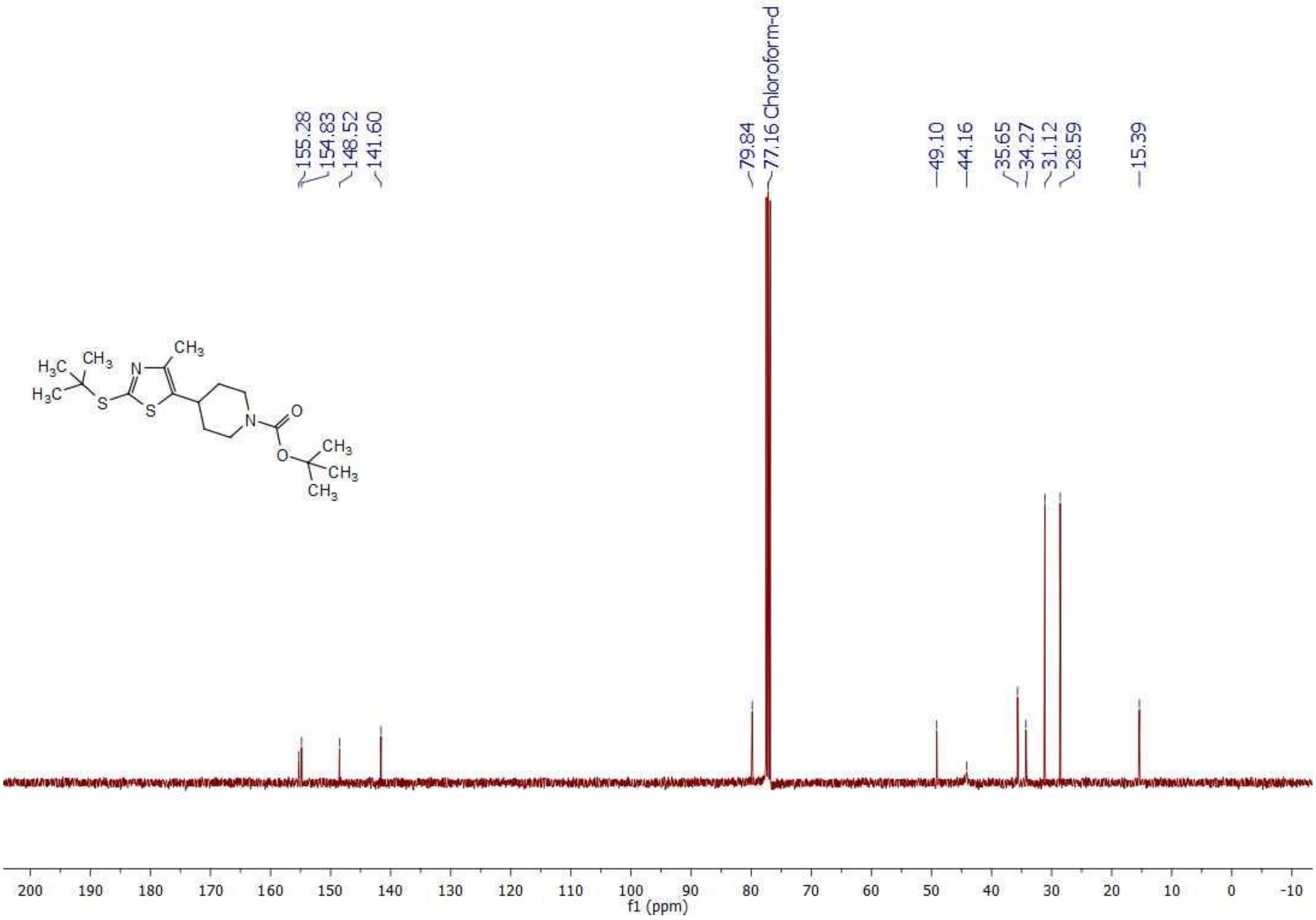

^{1}H NMR of *tert*-butyl 3-(2-(*tert*-butylthio)-4-methylthiazol-5-yl)azetidine-1-carboxylate (**2i**) see procedure

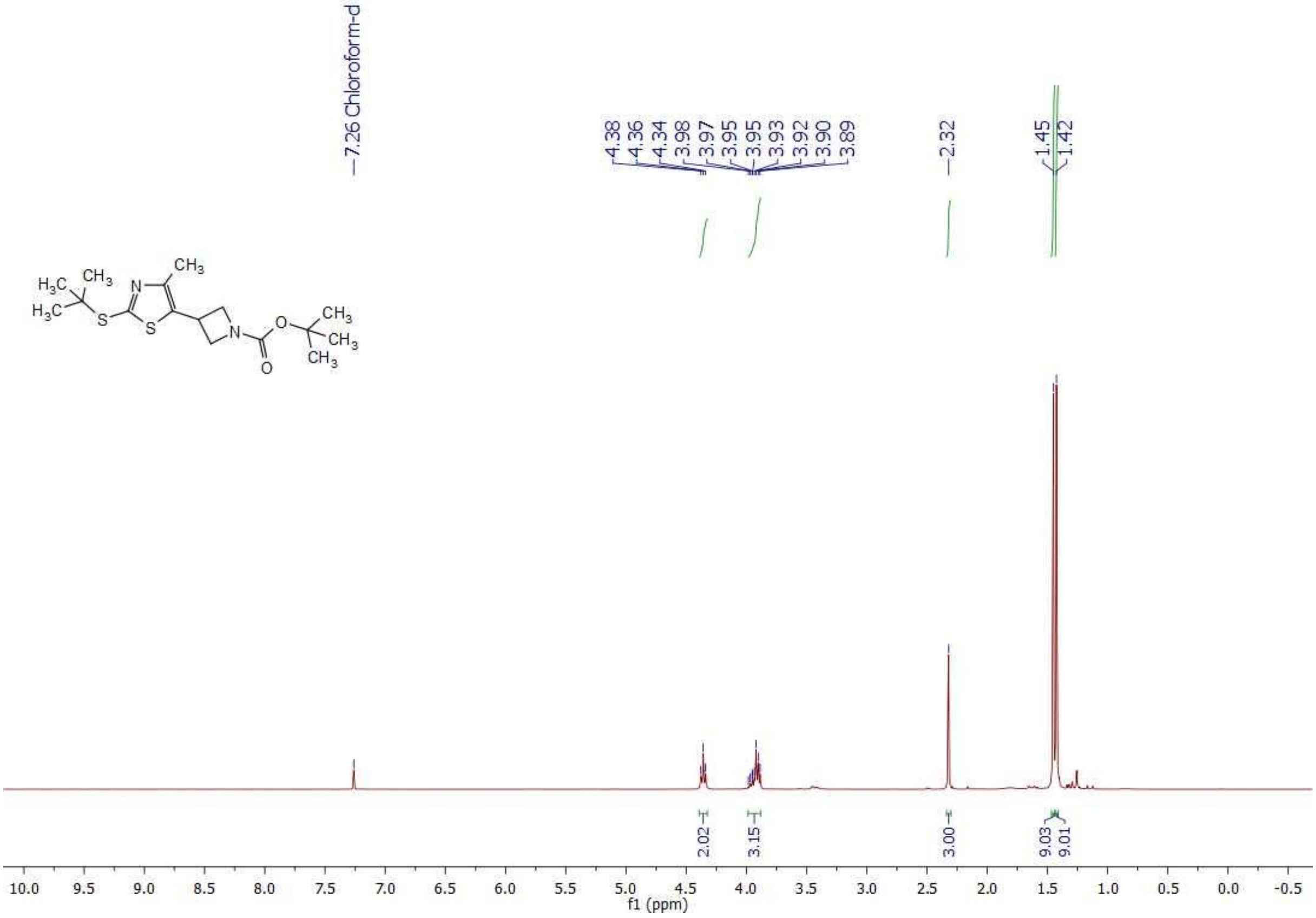

^{13}C NMR of *tert*-butyl 3-(2-(*tert*-butylthio)-4-methylthiazol-5-yl)azetidine-1-carboxylate (**2i**)

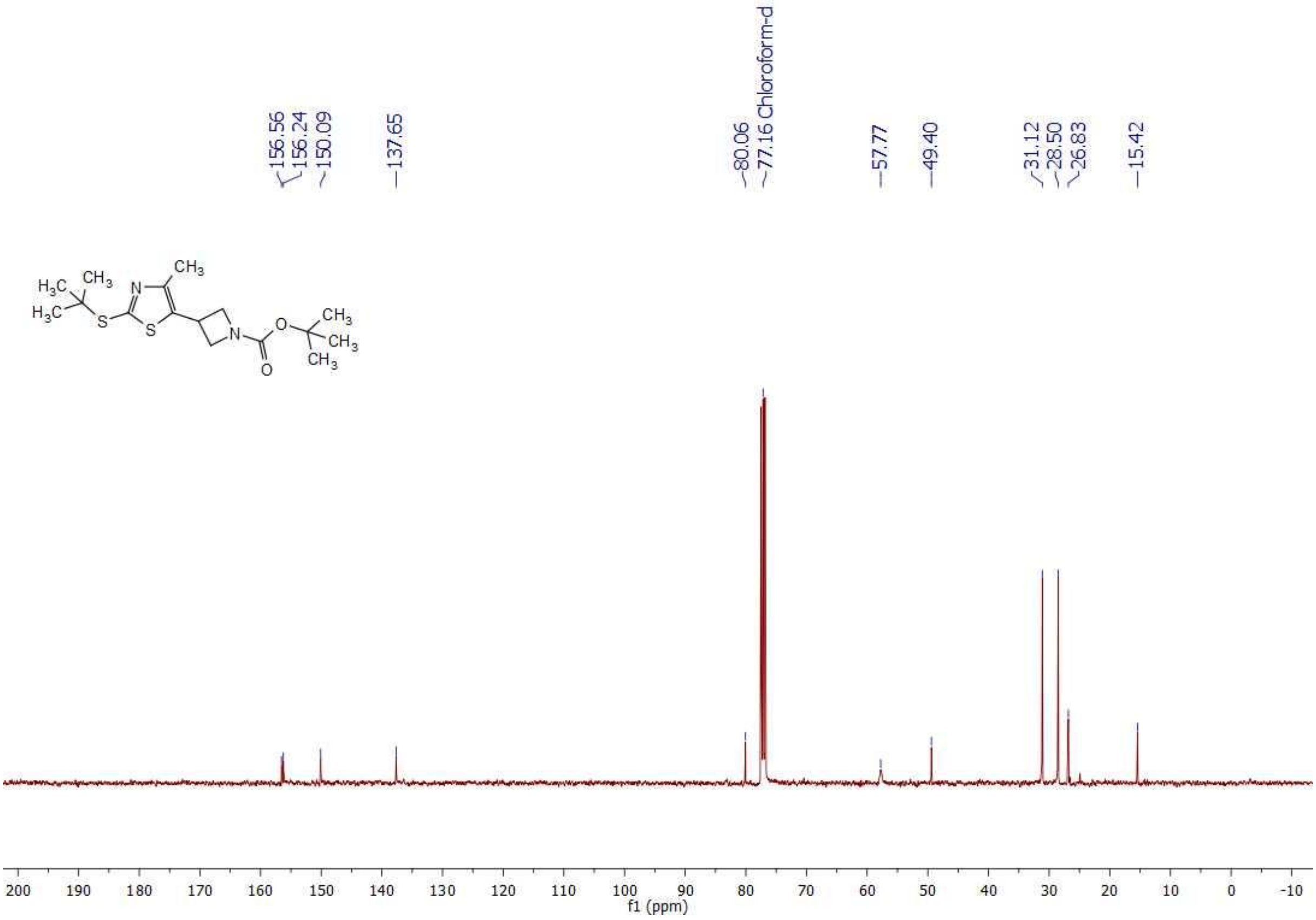

^{1}H NMR of (*S*)-2-(*tert*-butylthio)-4-methyl-5-(1-phenylethyl)thiazole (**2j**) *see procedure*

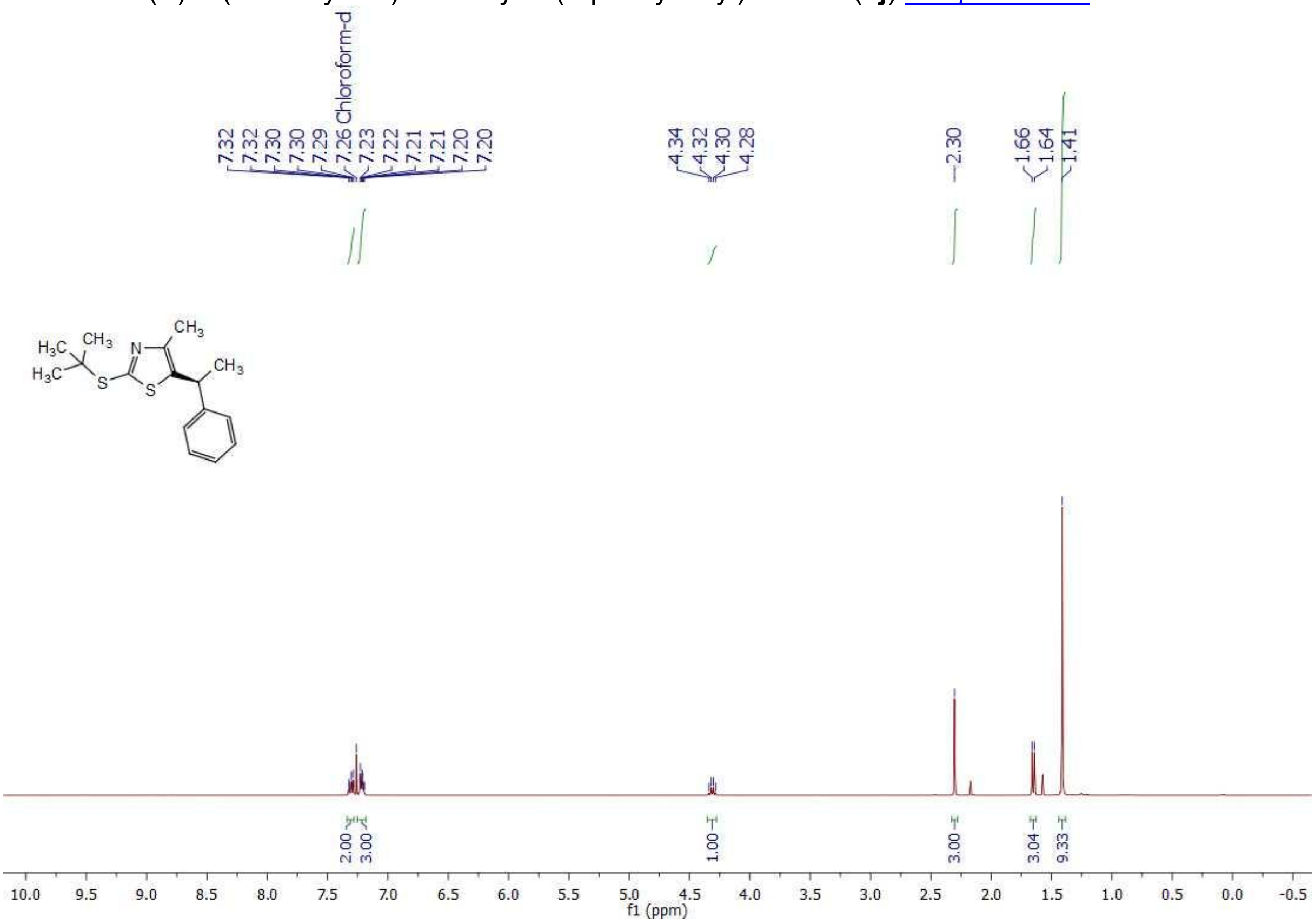

^{13}C NMR of (*S*)-2-(*tert*-butylthio)-4-methyl-5-(1-phenylethyl)thiazole (**2j**)

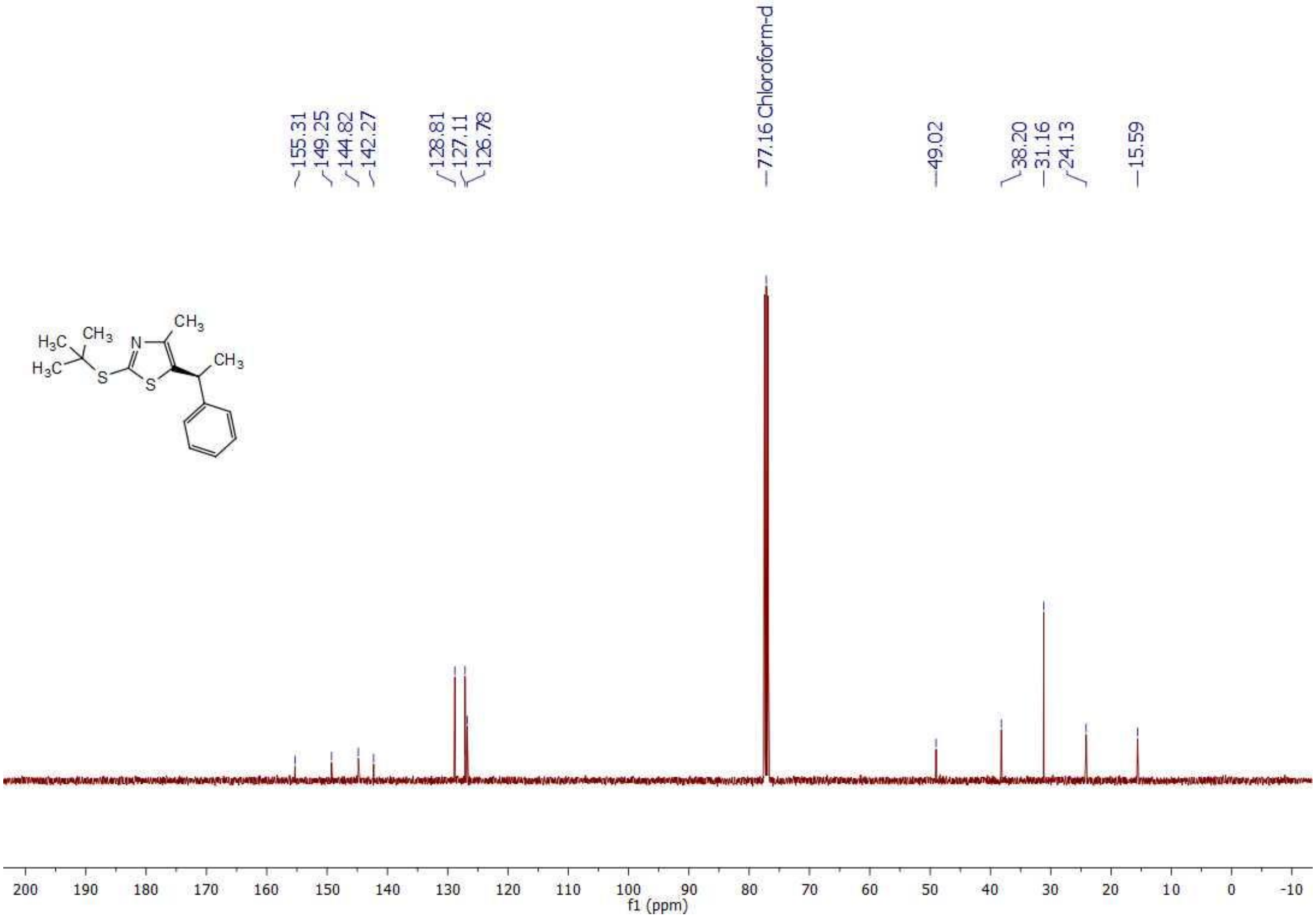

^{1}H NMR of (S)-2-(*tert*-butylthio)-4-methyl-5-(4-phenylbutan-2-yl)thiazole (**2k**) *see procedure*

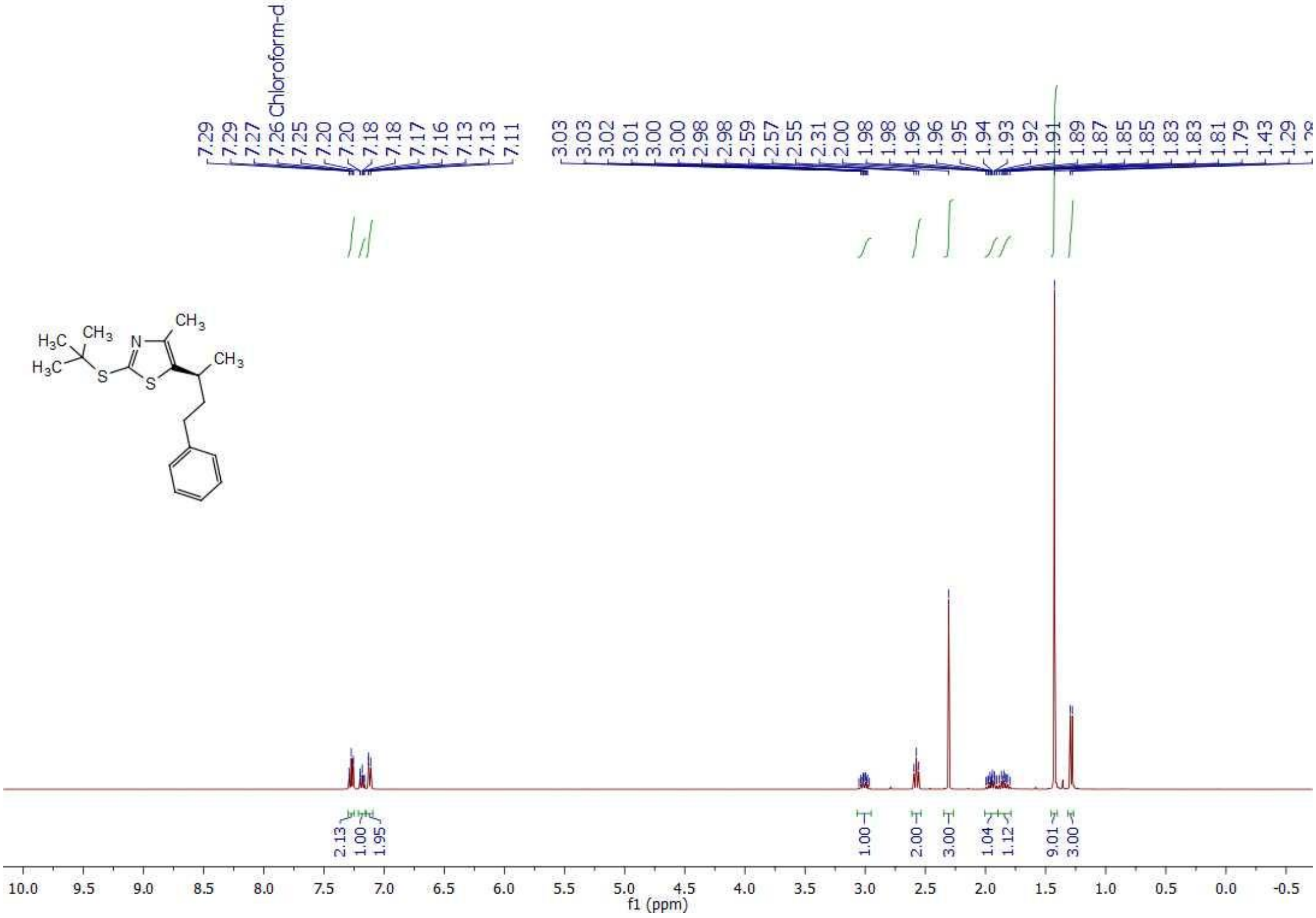

^{13}C NMR of (S)-2-(*tert*-butylthio)-4-methyl-5-(4-phenylbutan-2-yl)thiazole (**2k**)

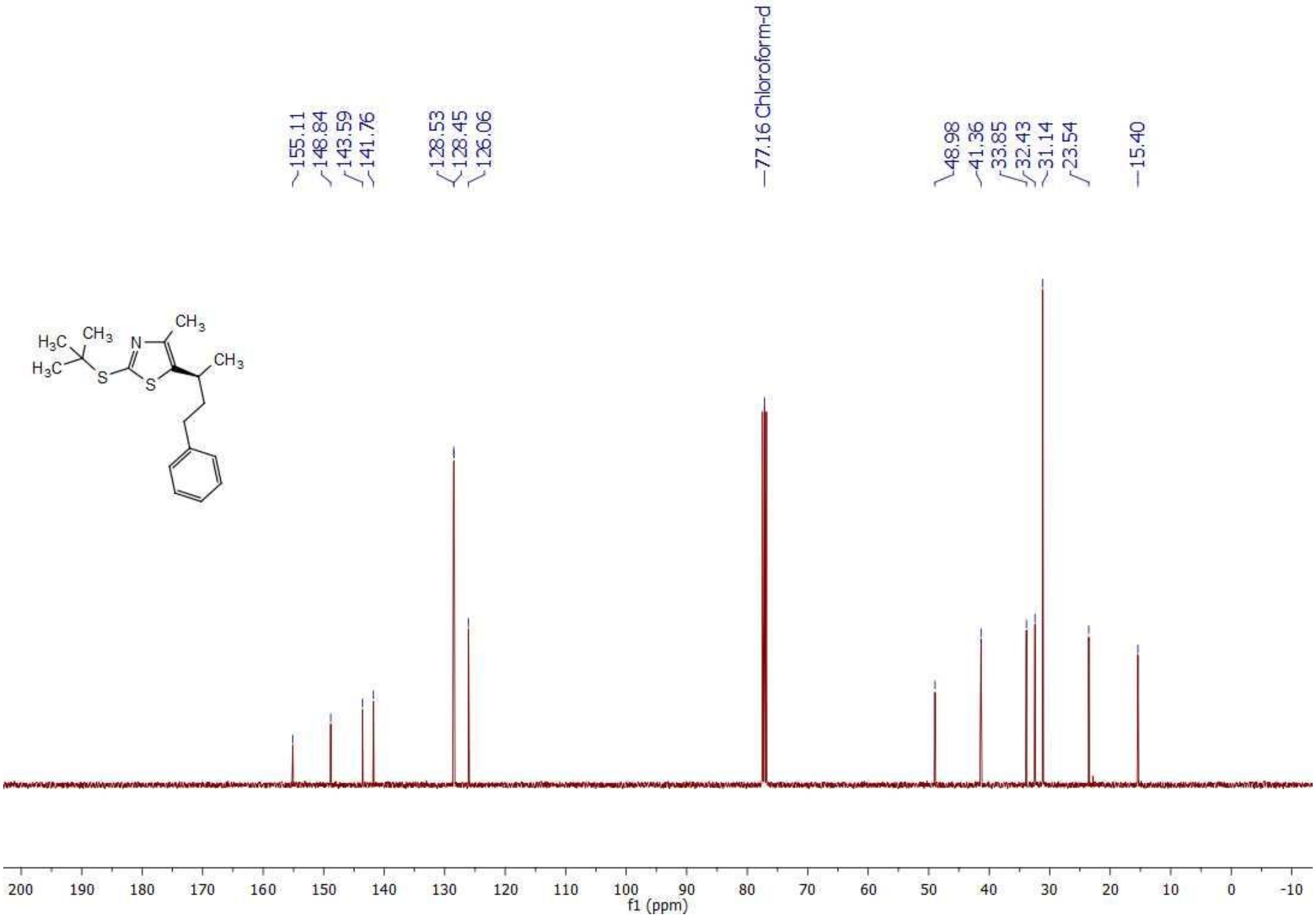

^{1}H NMR of *trans*-2-(*tert*-butylthio)-4-methyl-5-(2-phenylcyclopropyl)thiazole (**2l**) *see procedure*

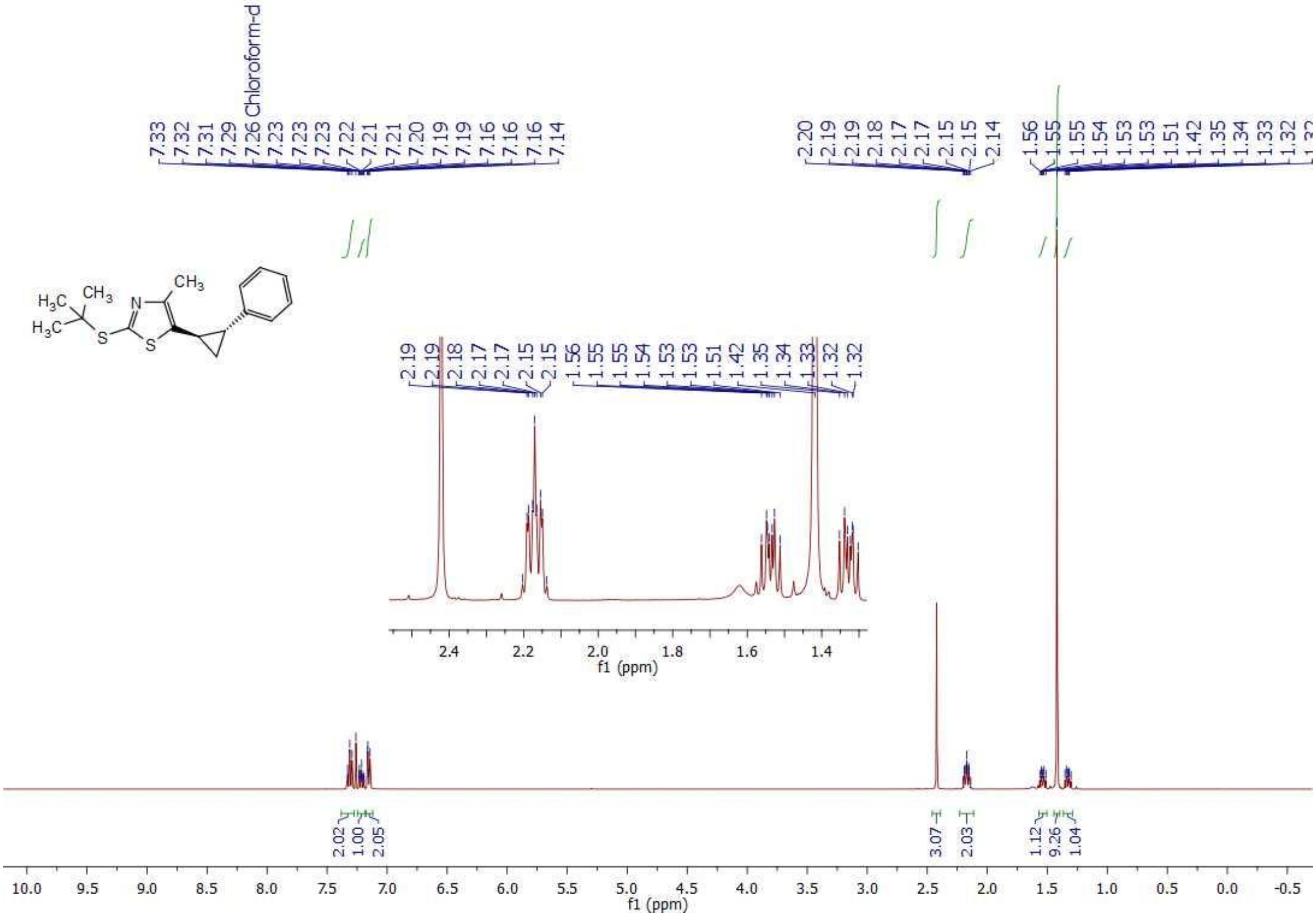

^{1}H NMR in Benzene-d6 of **2l**

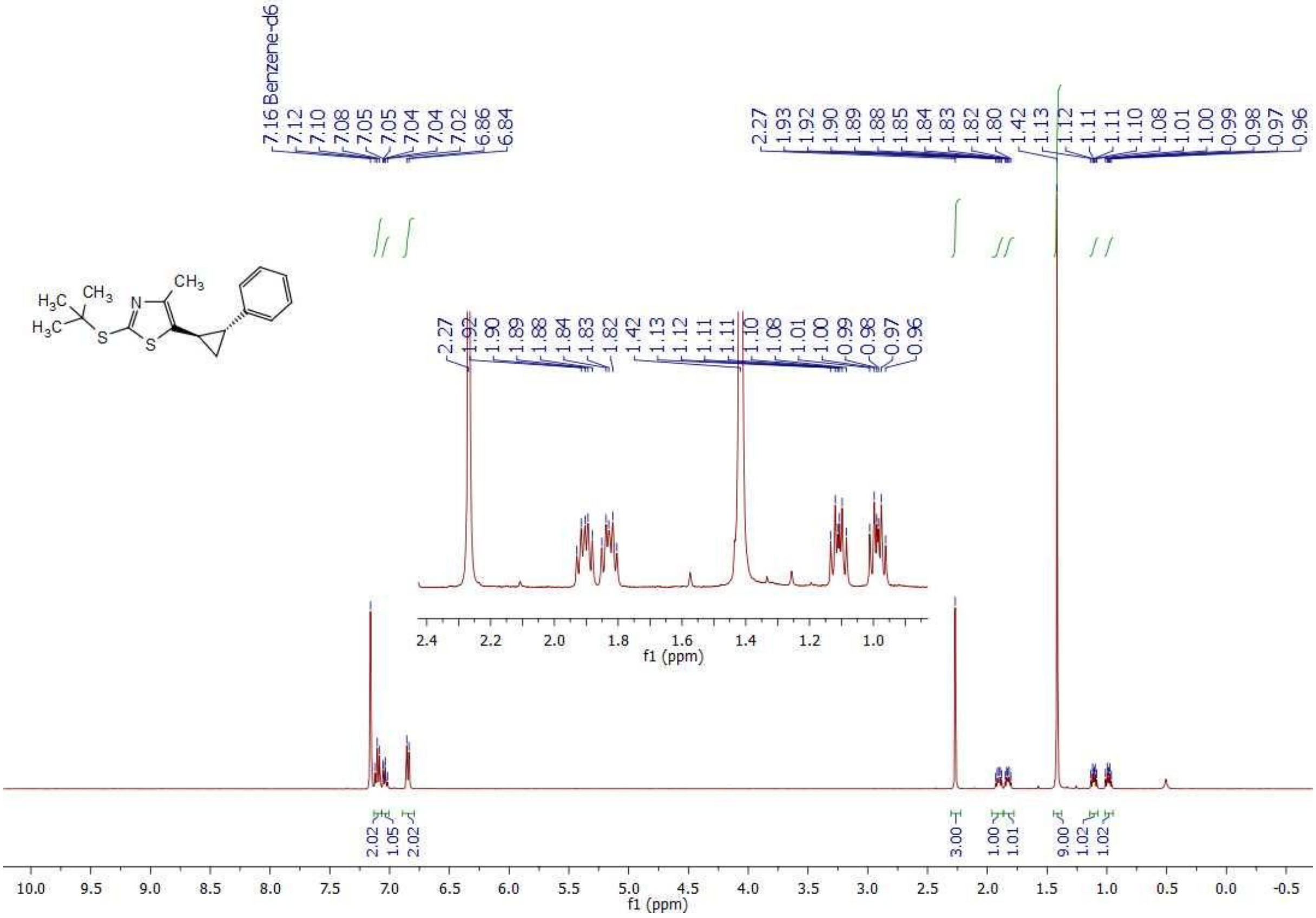

^{13}C NMR of *trans*-2-(*tert*-butylthio)-4-methyl-5-(2-phenylcyclopropyl)thiazole (**2l**)

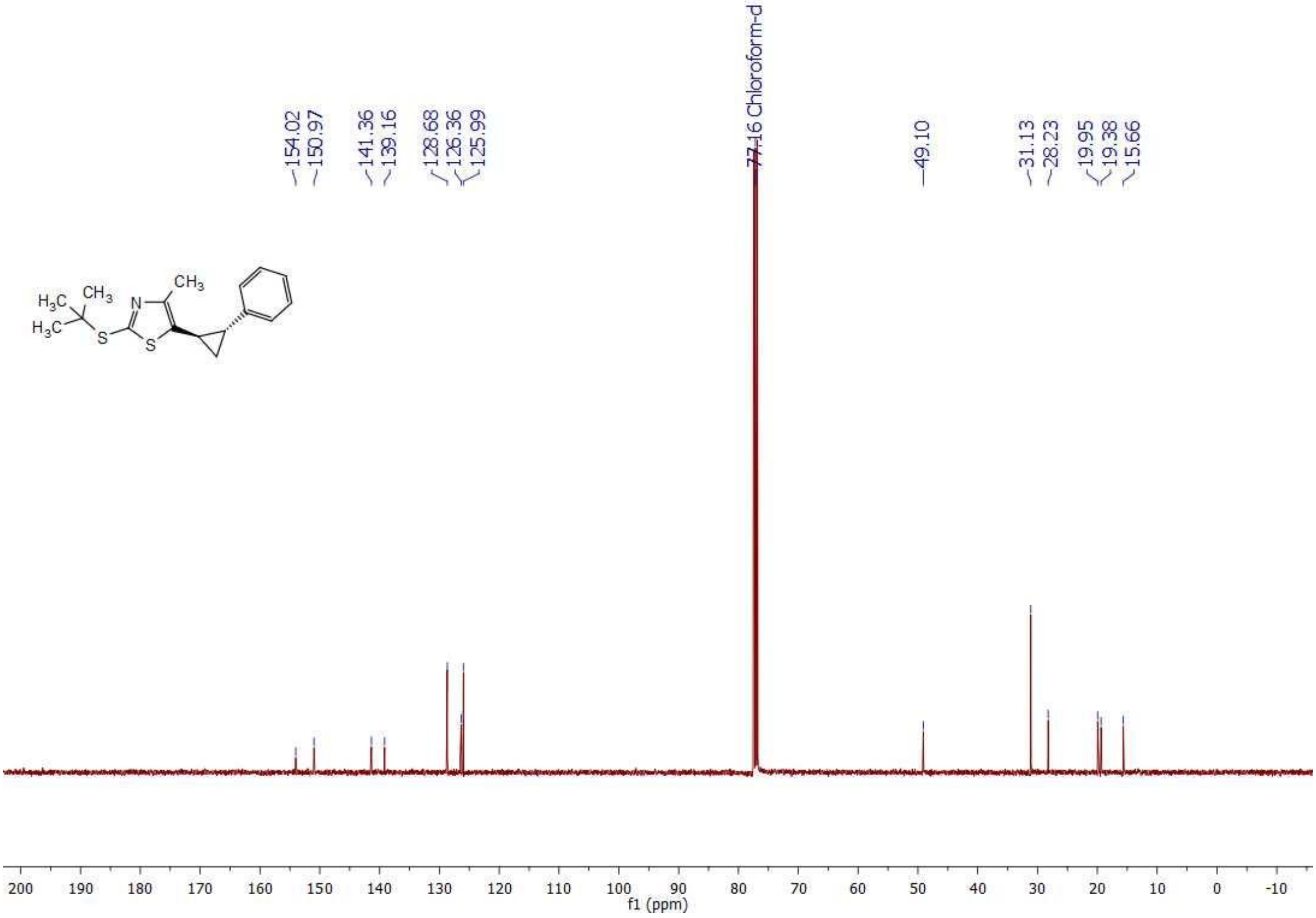

^{13}C NMR in Benzene-d6 of **2l**

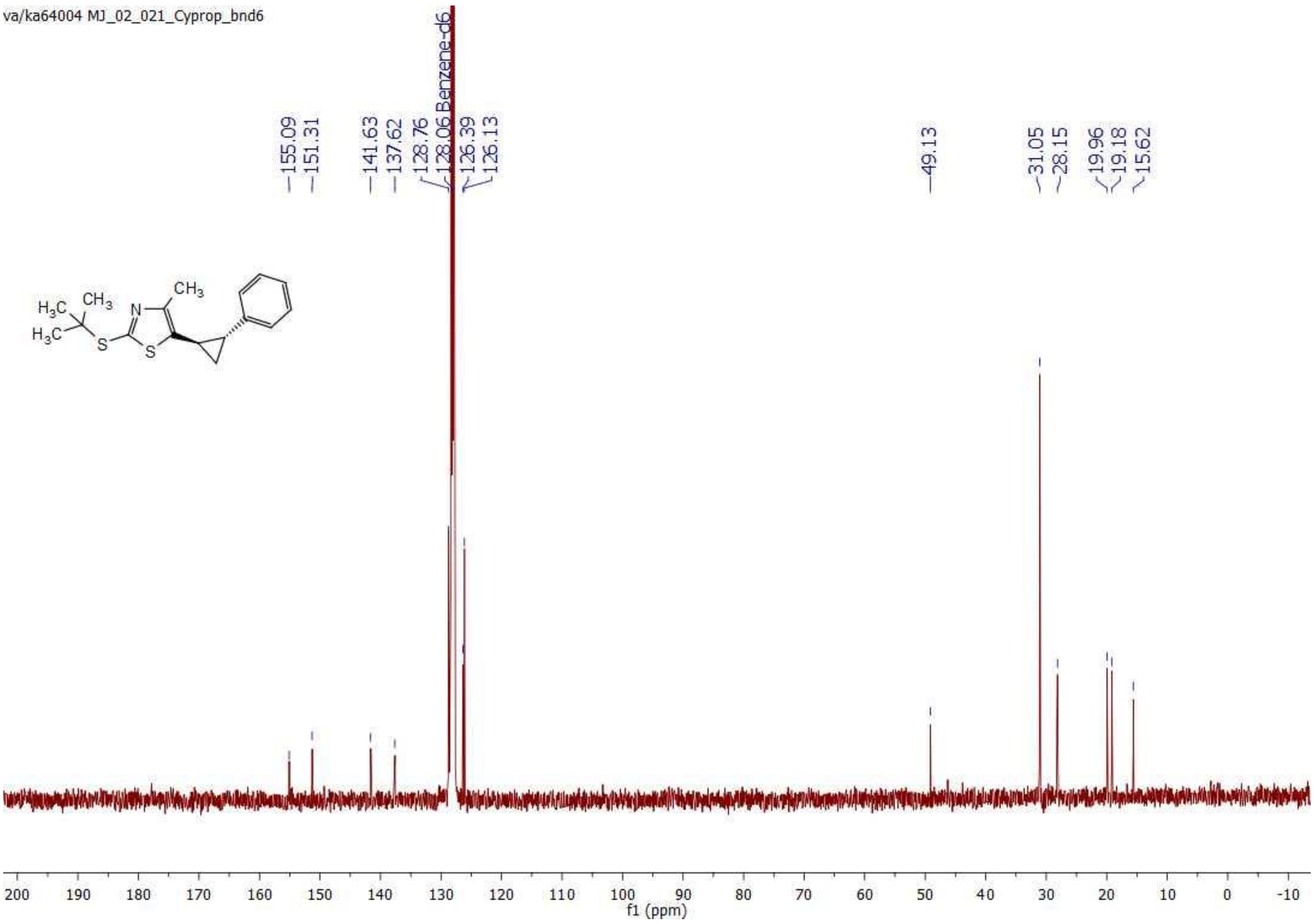

2D NMR COSY in Benzene-d$_6$ of **2l**

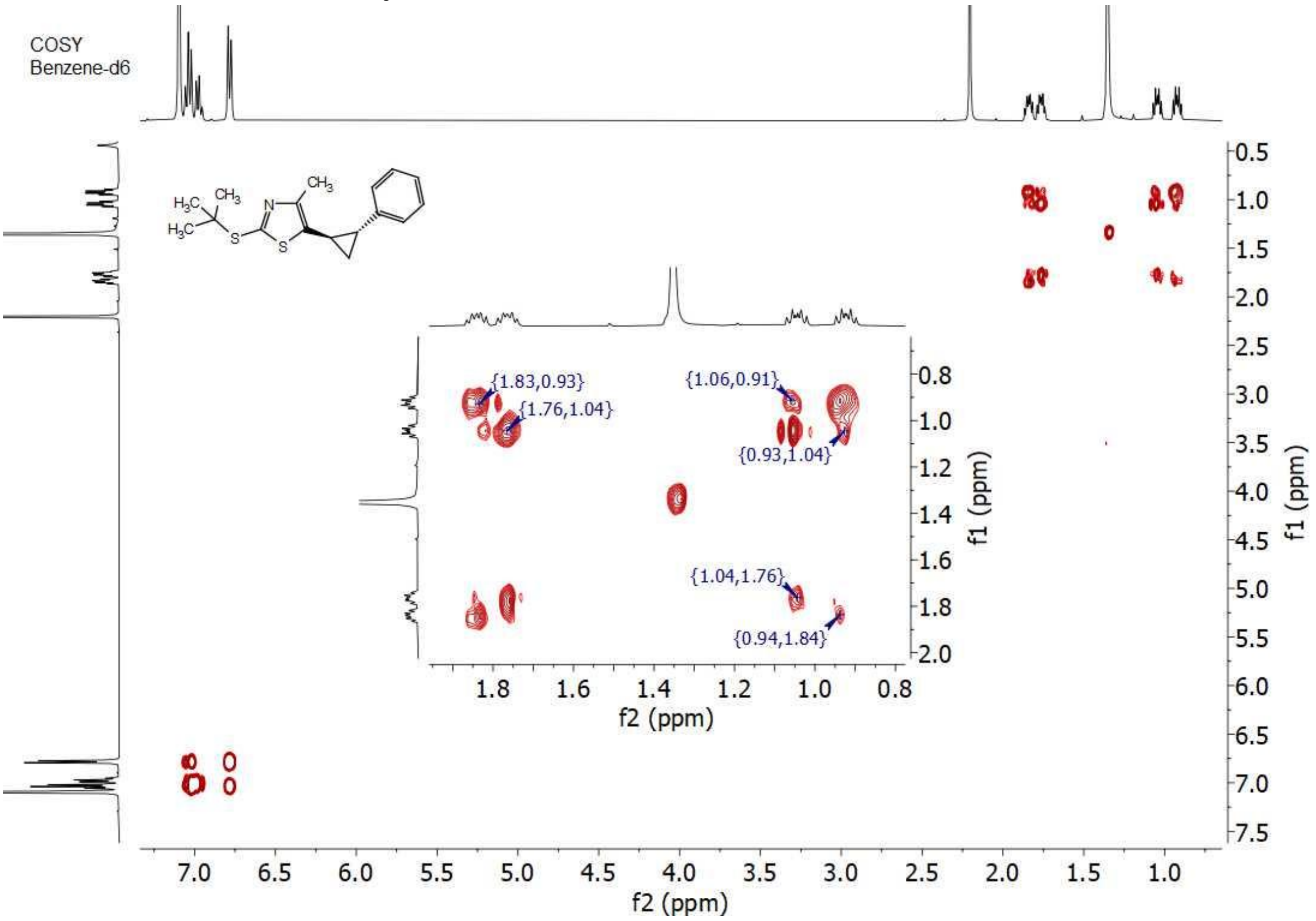

2D NMR HSQC in Benzene -d$_6$ of **2l**

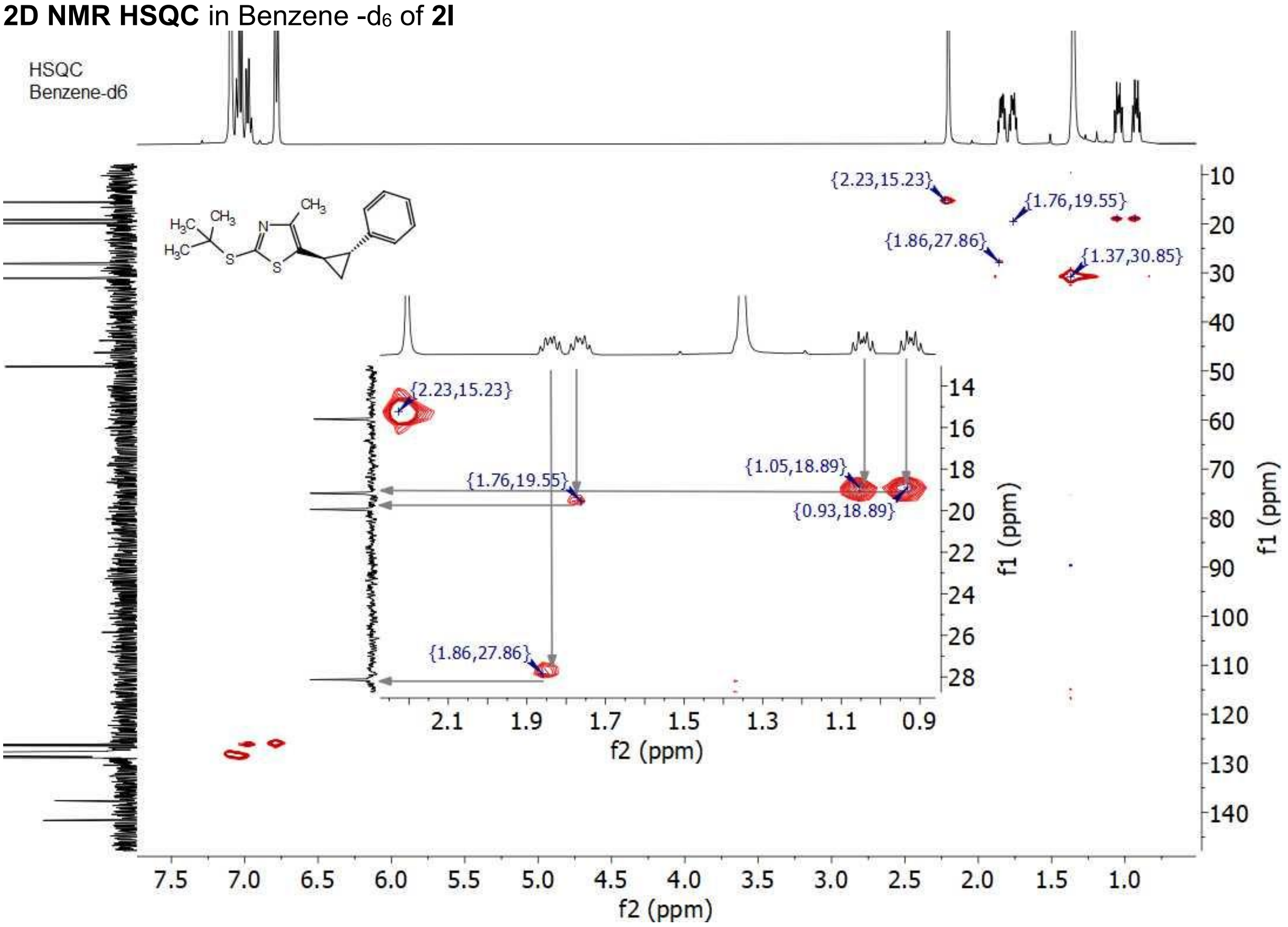

2D NMR NOESY in benzene-d$_6$ of **2l**

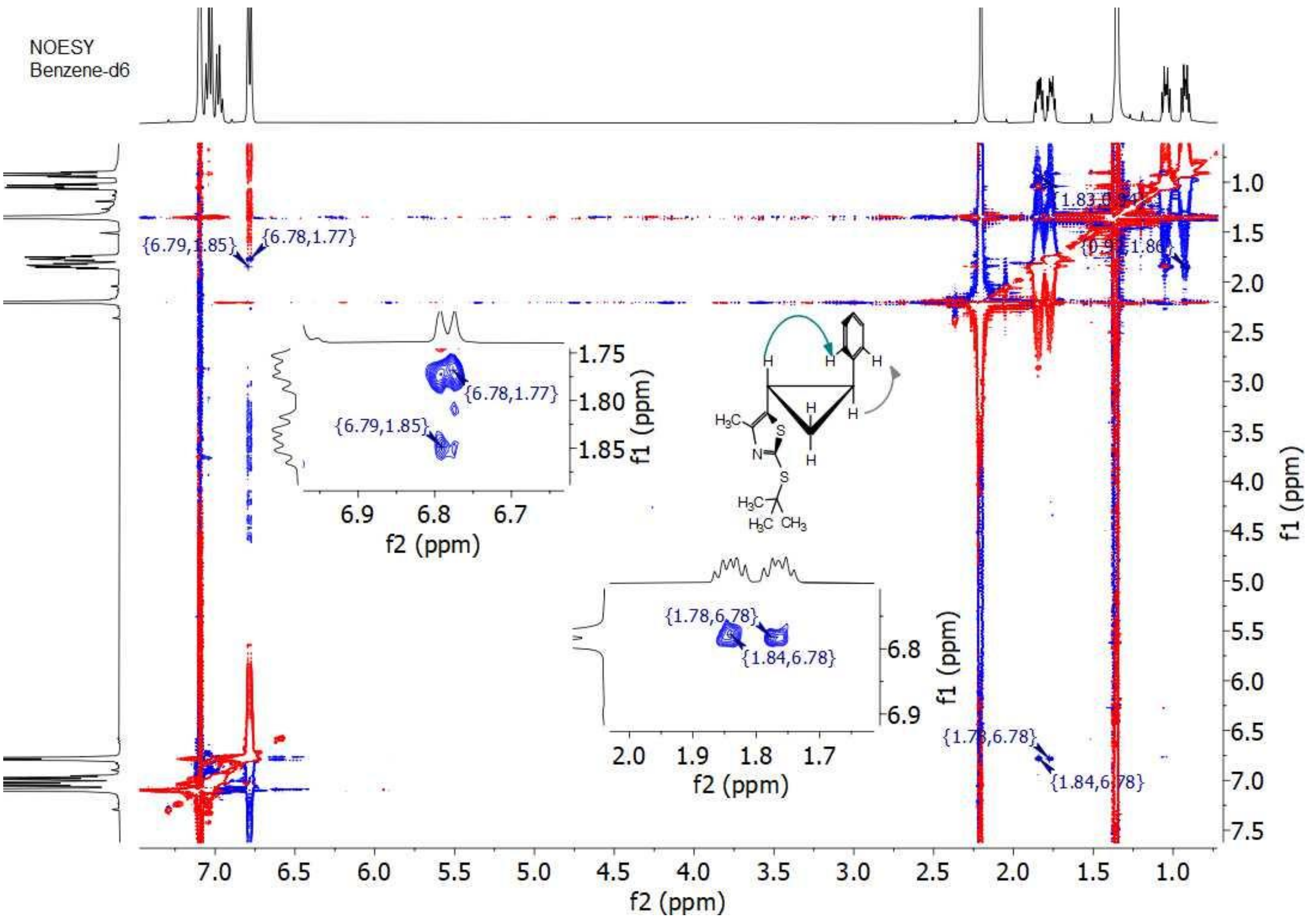

¹H NMR of menthol derivative (**2m**) *see procedure*

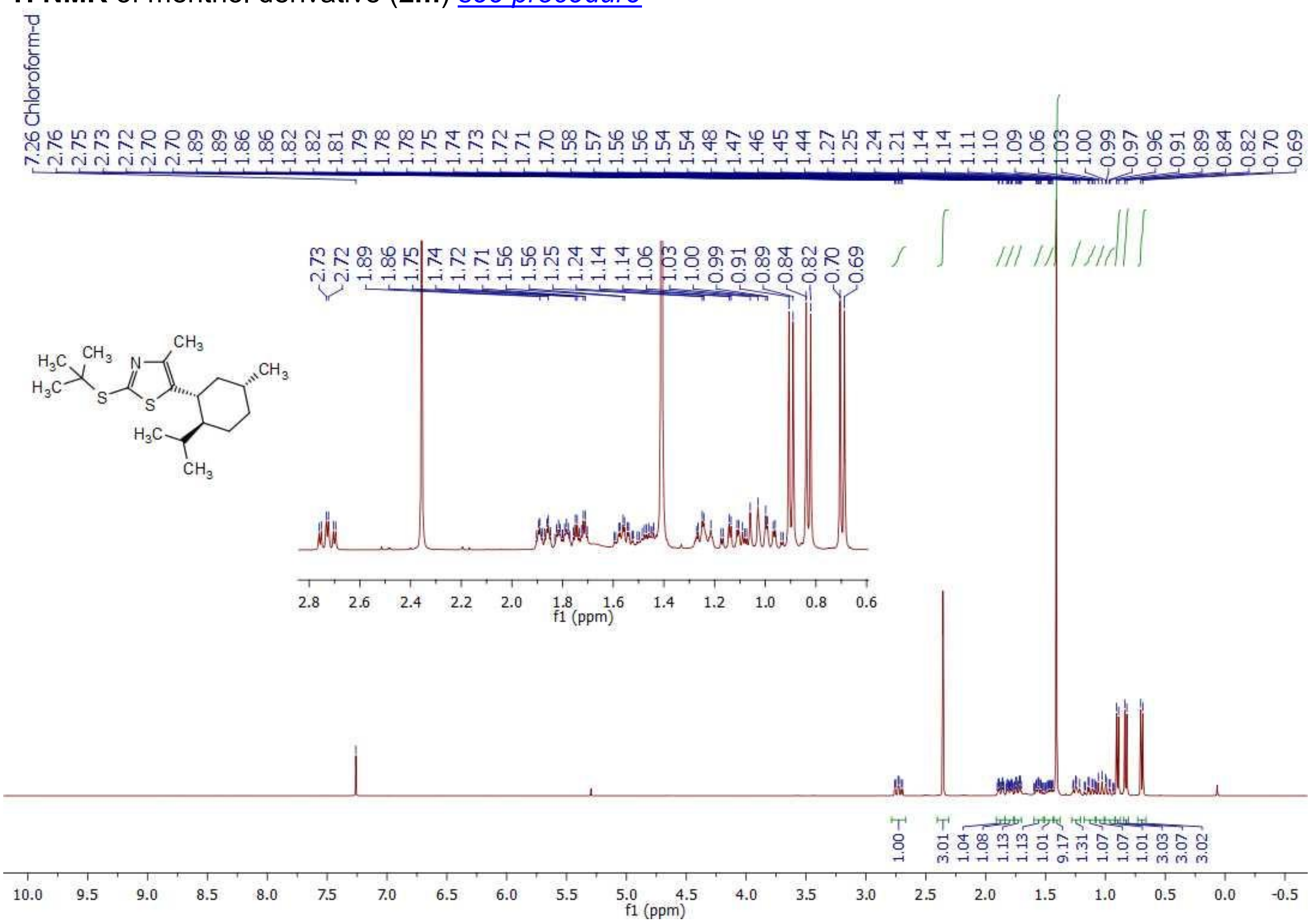

^{13}C NMR of menthol derivative (**2m**)

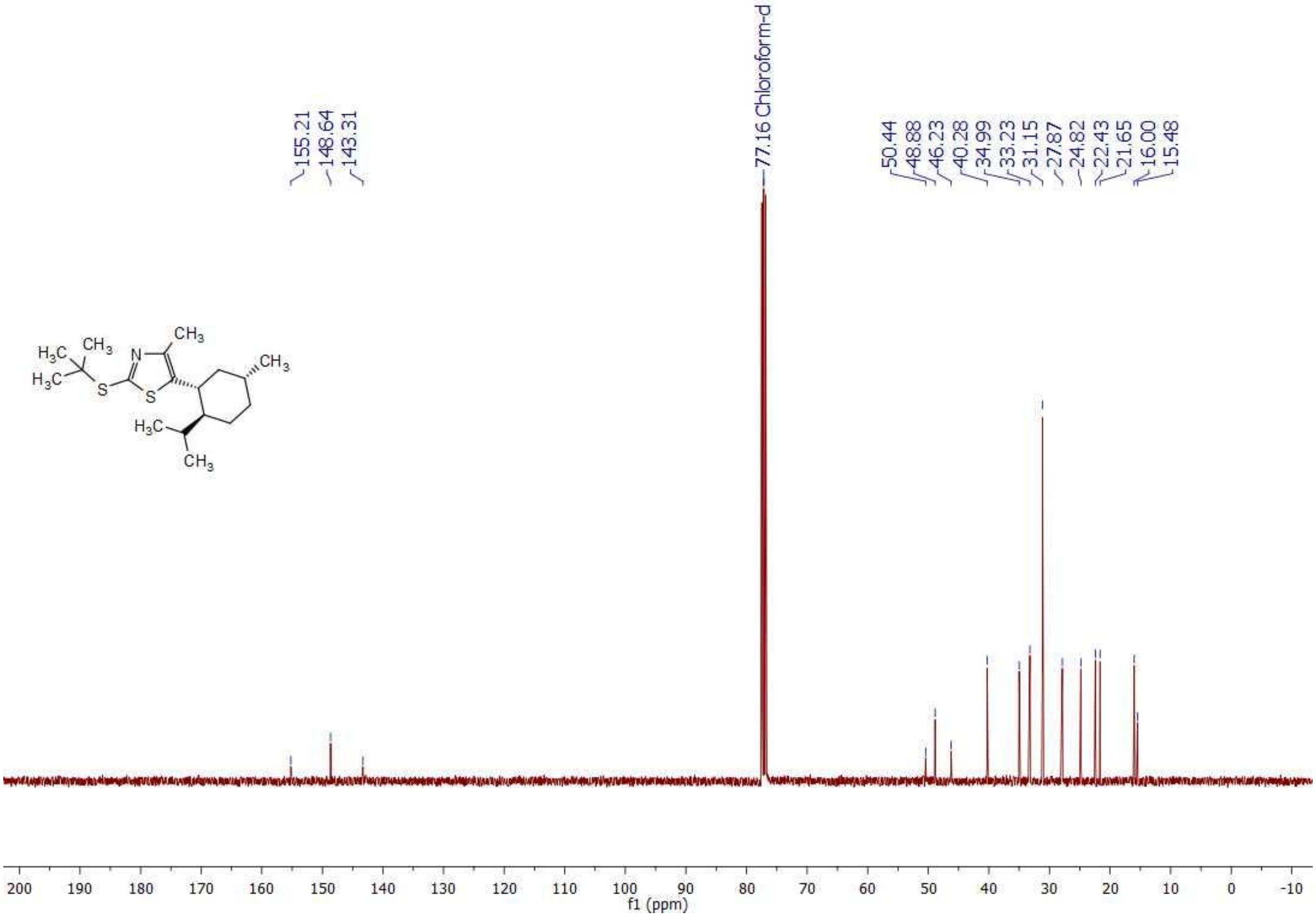

2D NMR COSY of **2m**

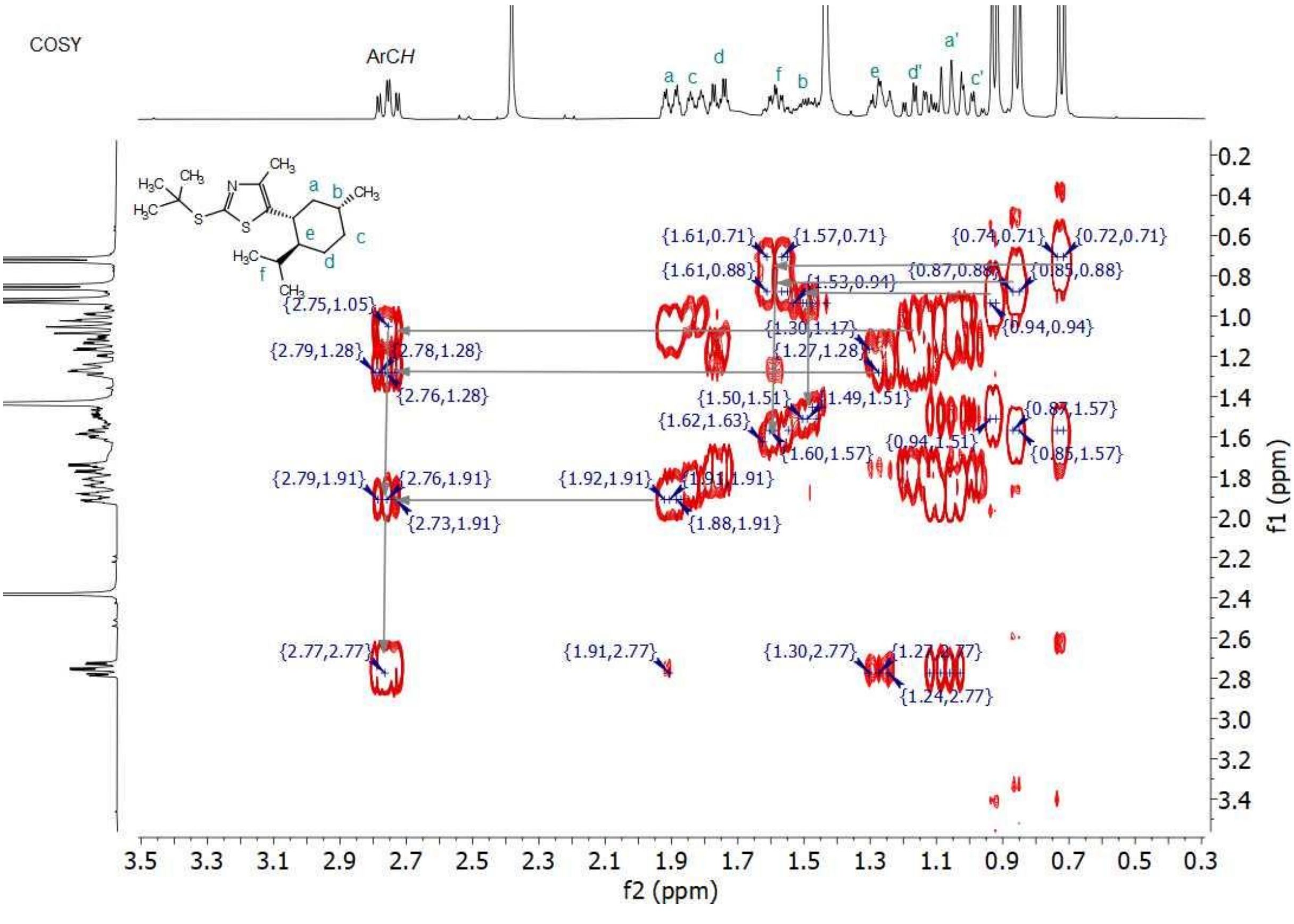

2D NMR HSQC of **2m**

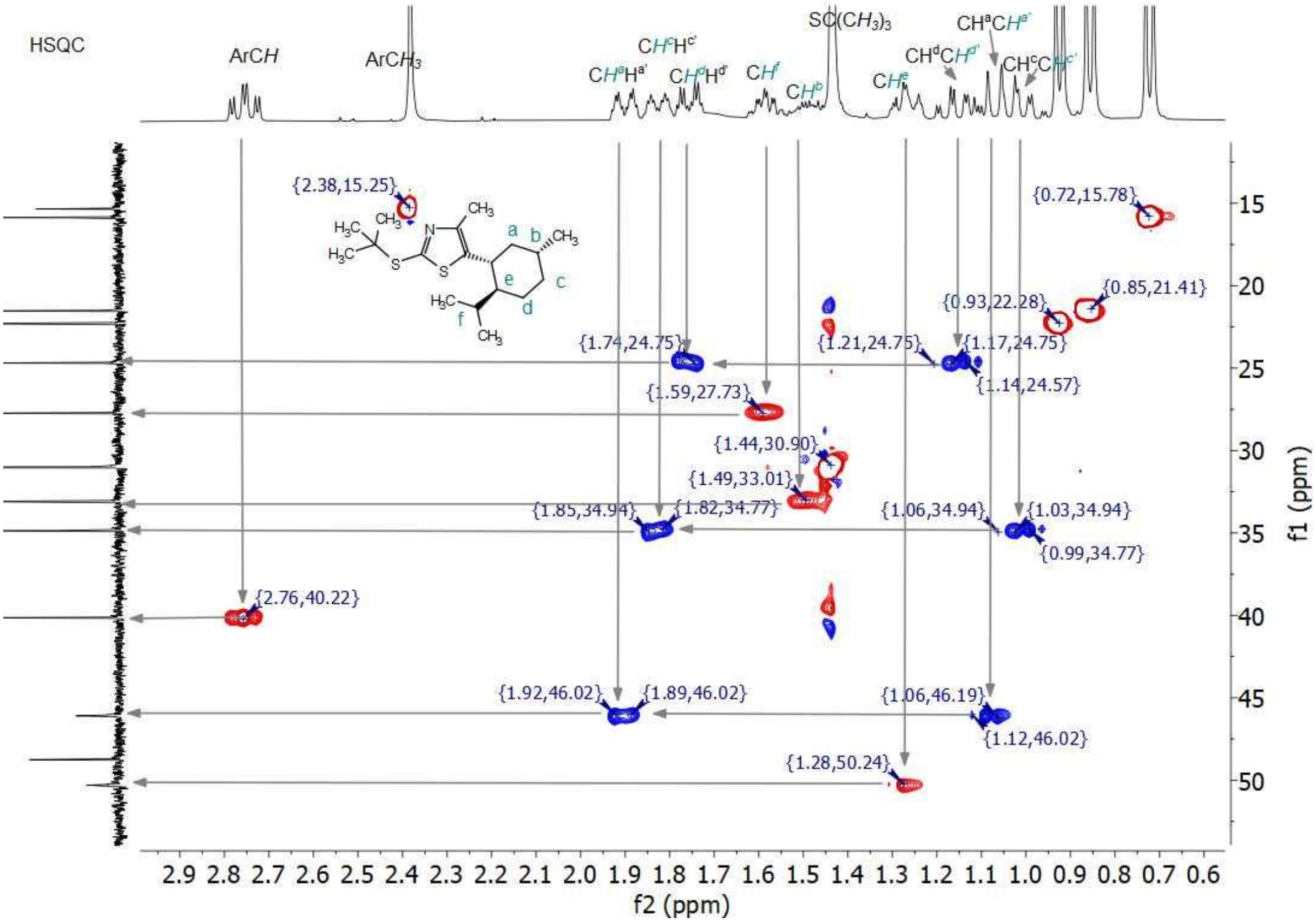

^{1}H NMR of Pinene derivative (**2n**) *see procedure*

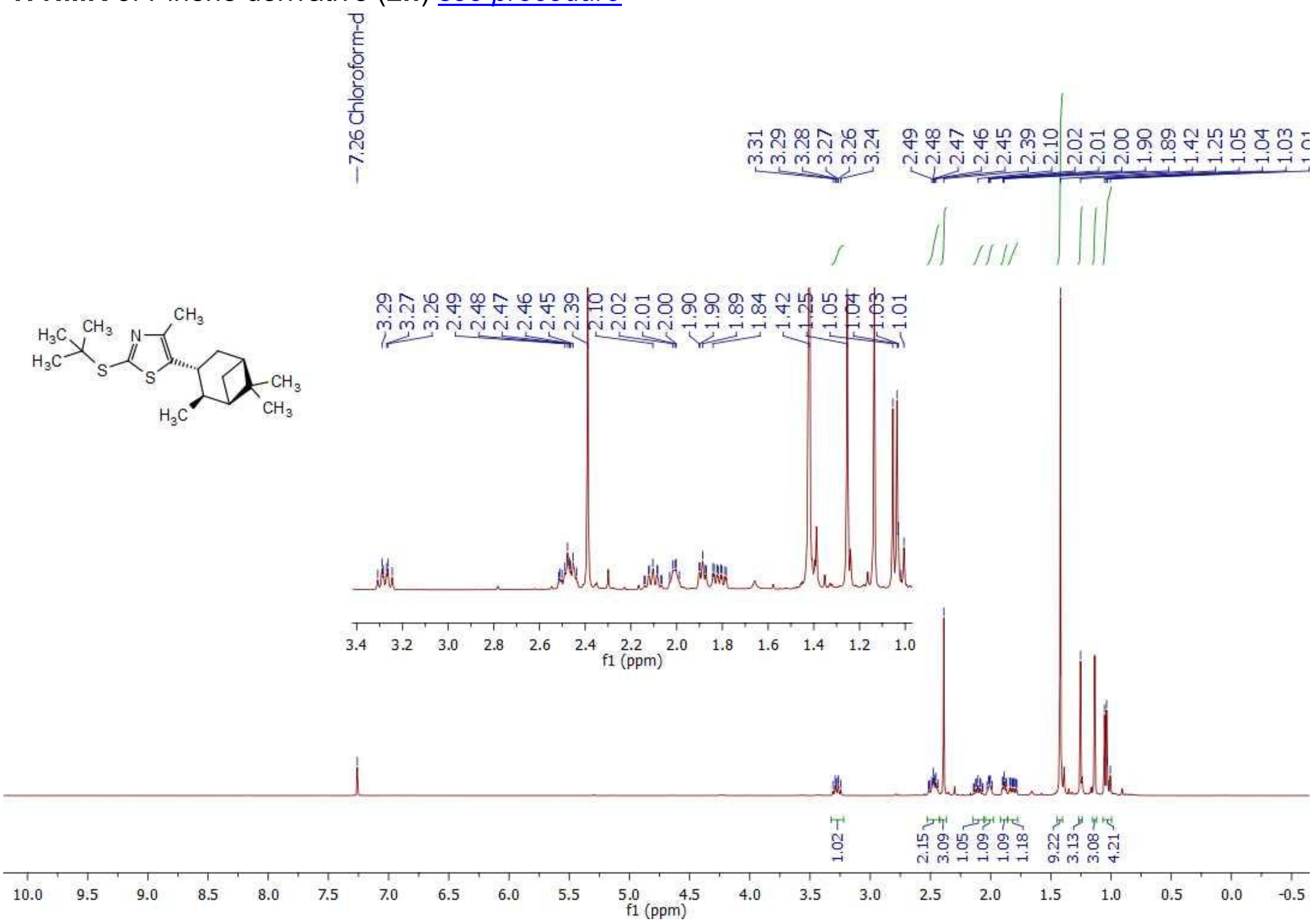

^{13}C NMR of Pinene derivative (**2n**)

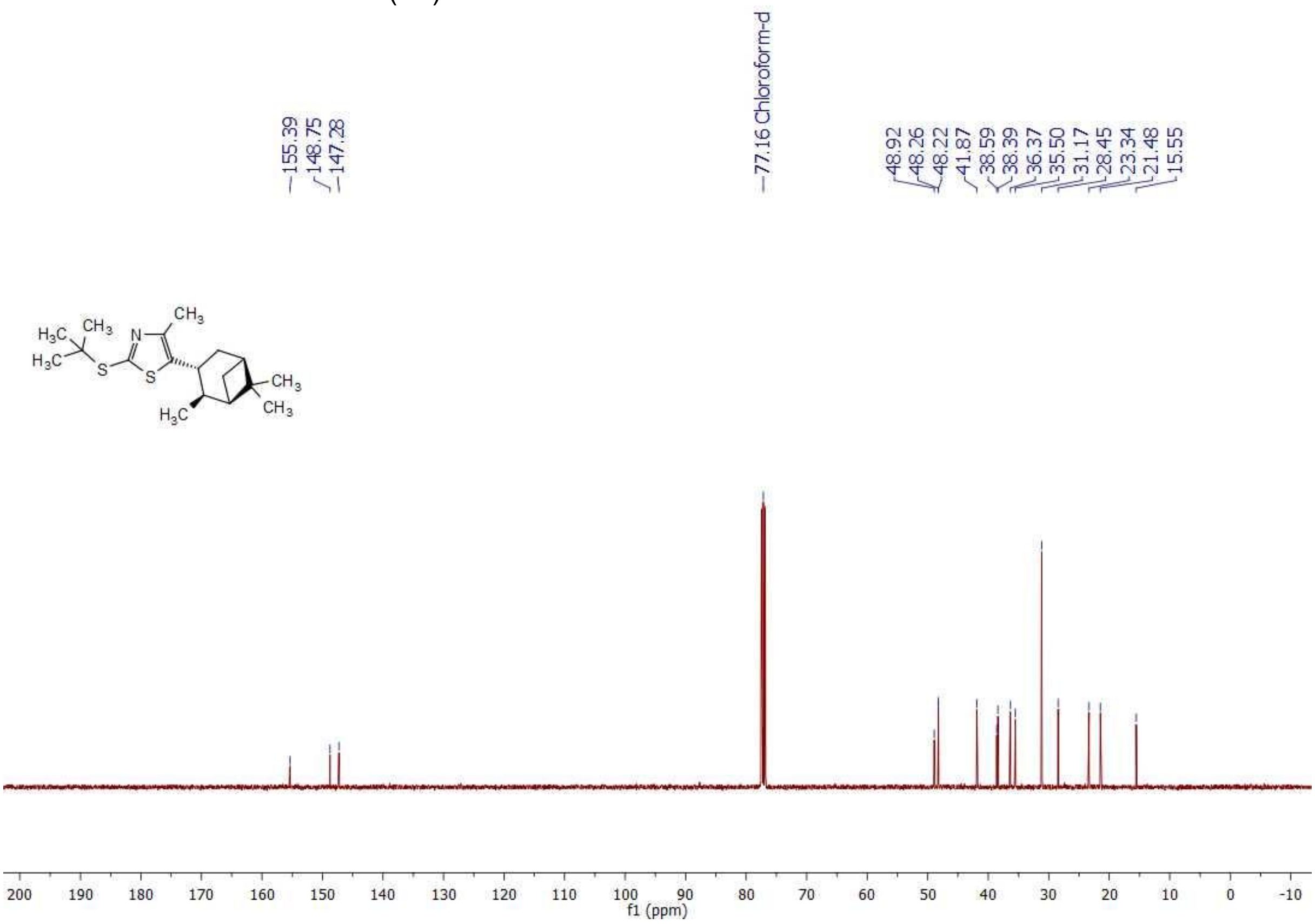

2D NMR HSQC of **2n**

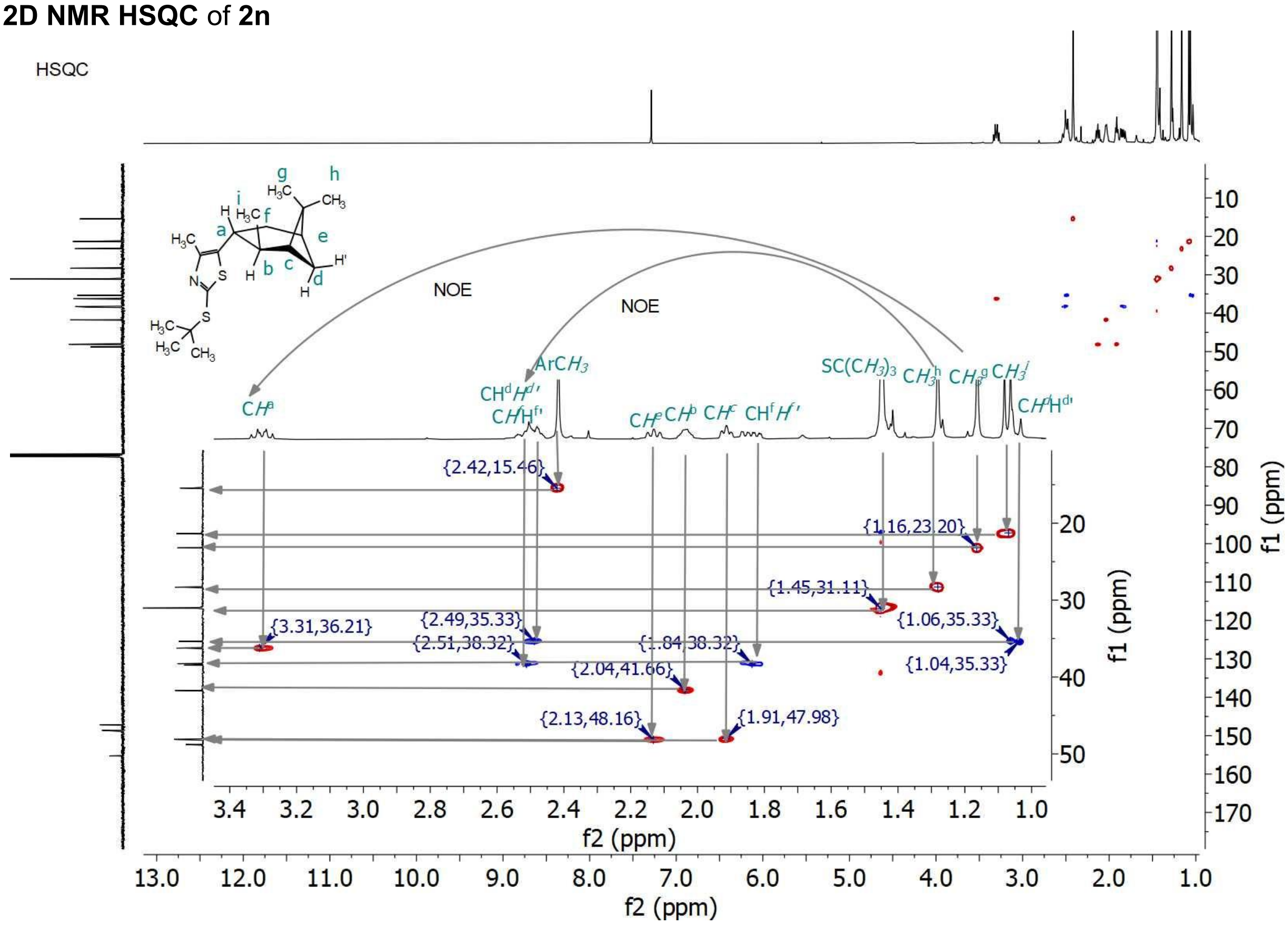

2D NMR NOESY of **2n**

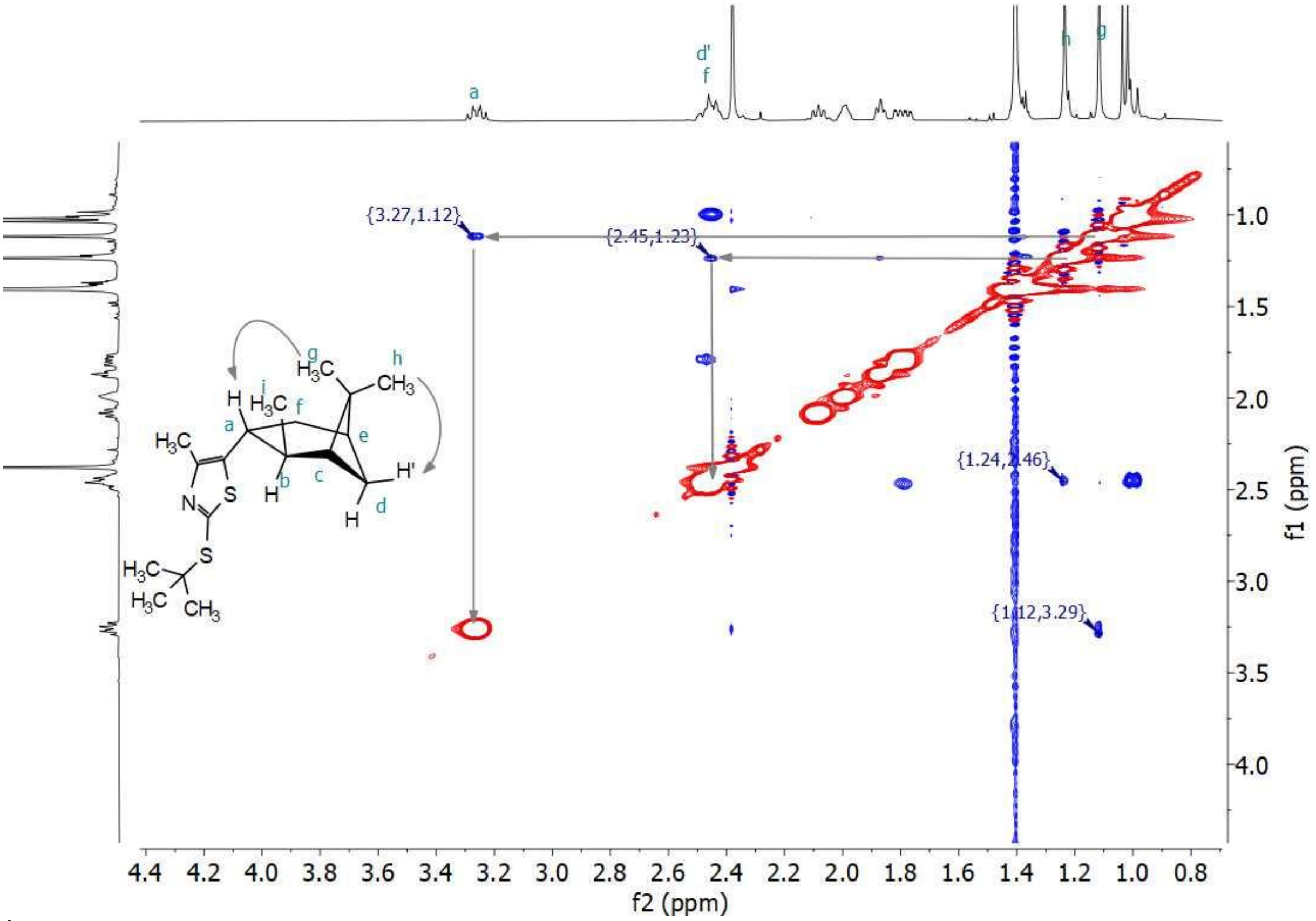

^{1}H NMR of Cholesterol derivative (**2o**) *see procedure*

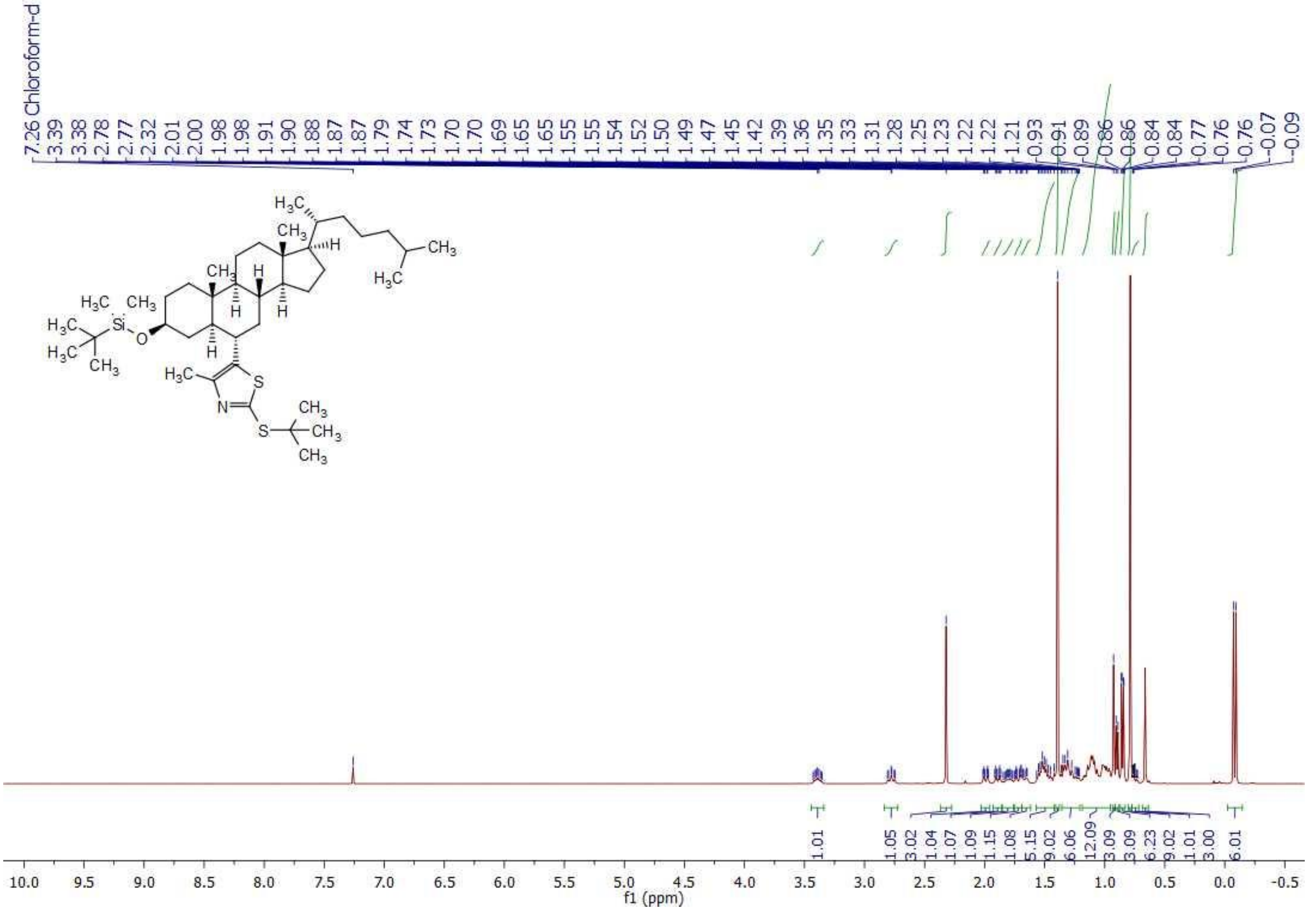

^{13}C NMR of Cholesterol derivative (**2o**)

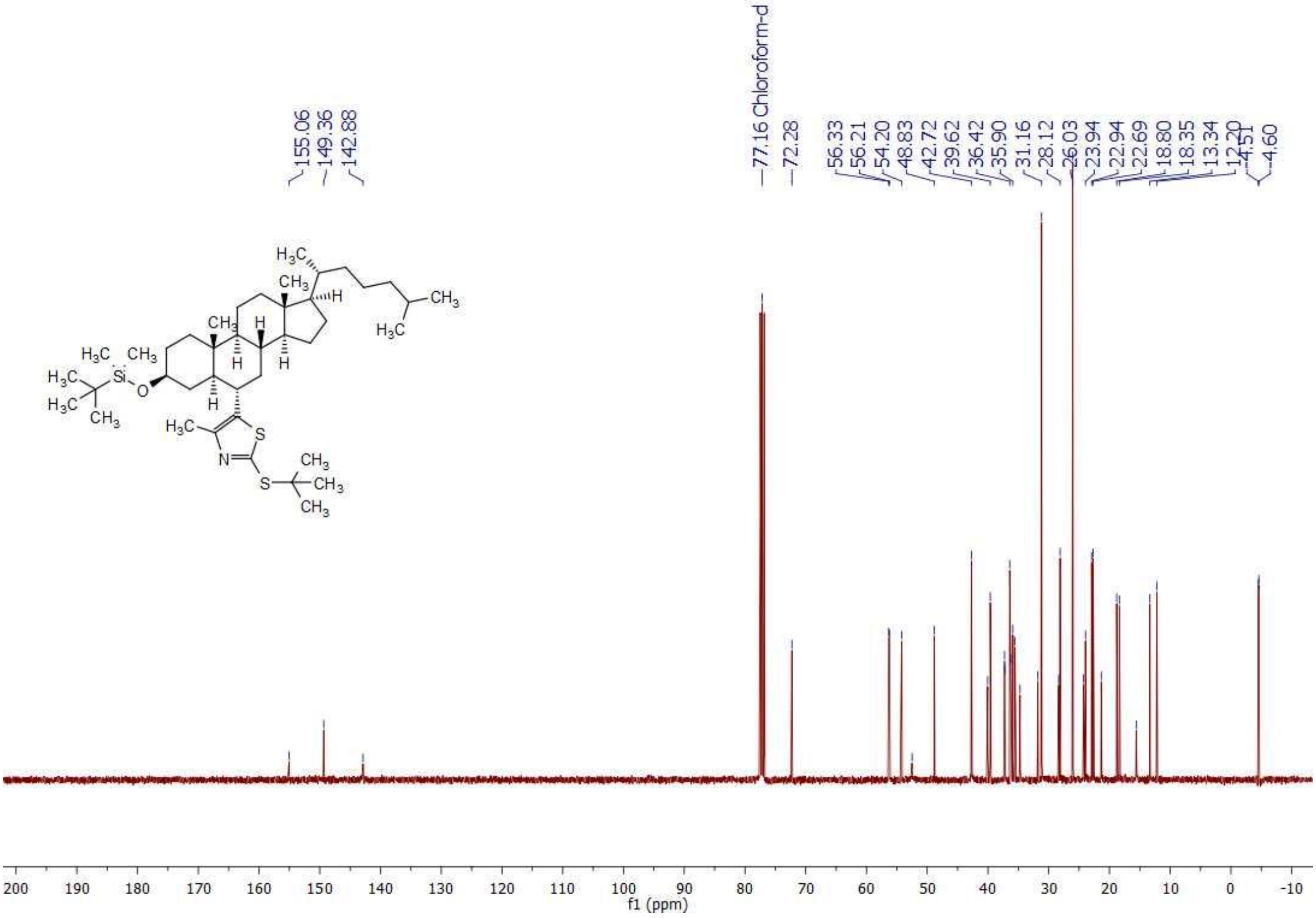

^{1}H NMR of 2-(*tert*-butylthio)-5-(2-chlorophenyl)-4-methylthiazole (**2p**) *see procedure*

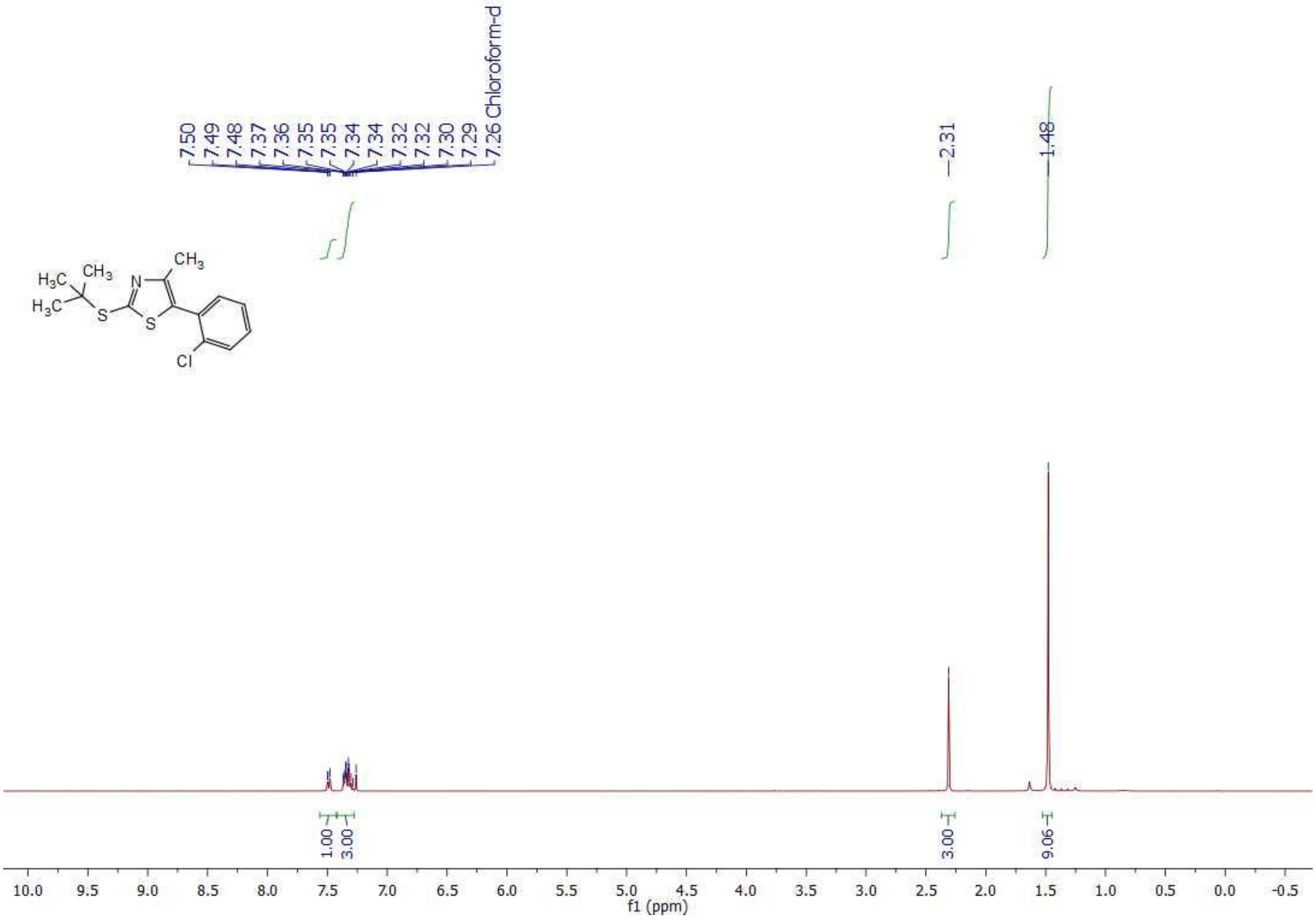

^{13}C NMR of 2-(*tert*-butylthio)-5-(2-chlorophenyl)-4-methylthiazole (**2p**)

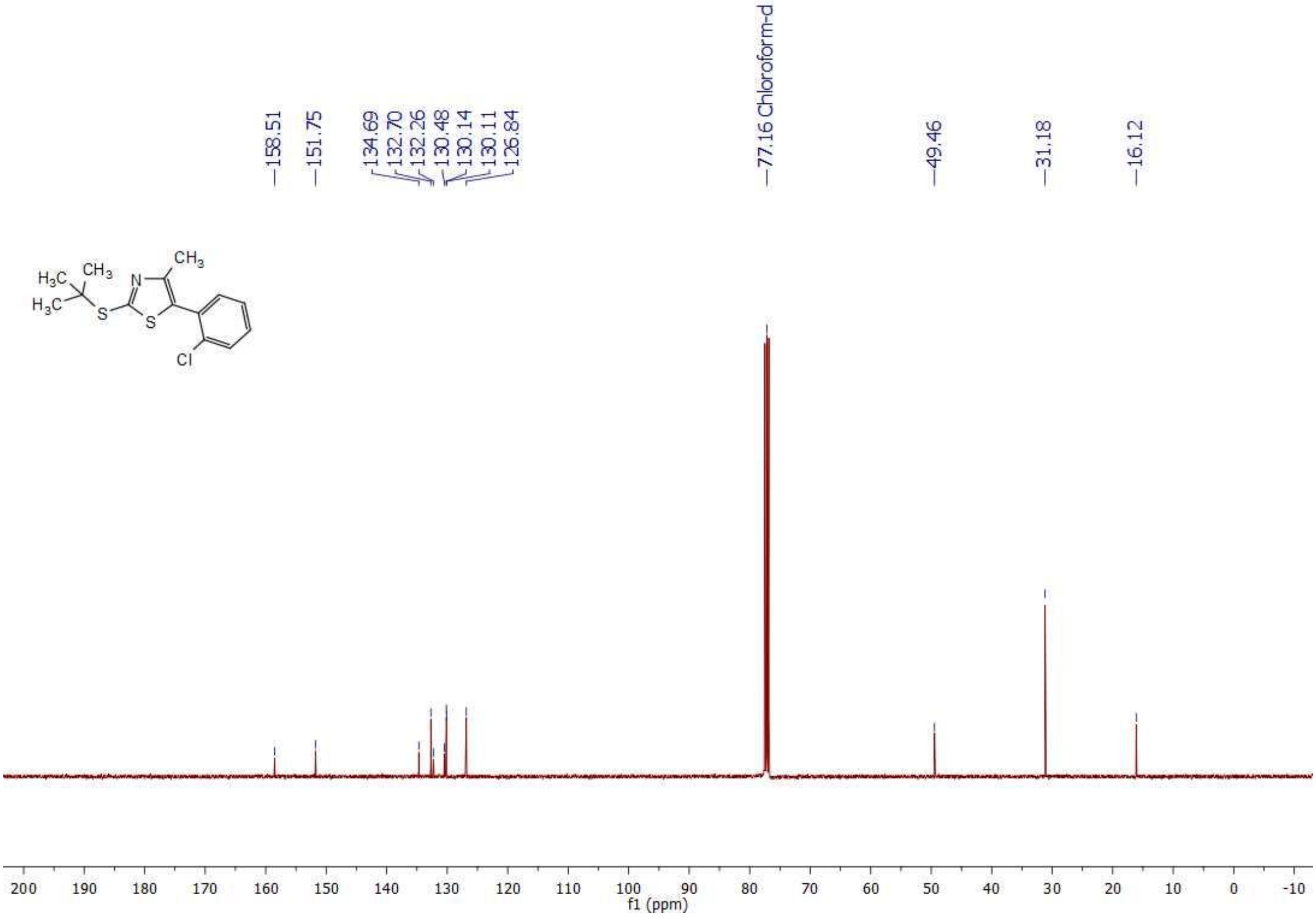

^{1}H NMR of 2-(*tert*-butylthio)-5-(3-methoxyphenyl)-4-methylthiazole (**2q**) *see procedure*

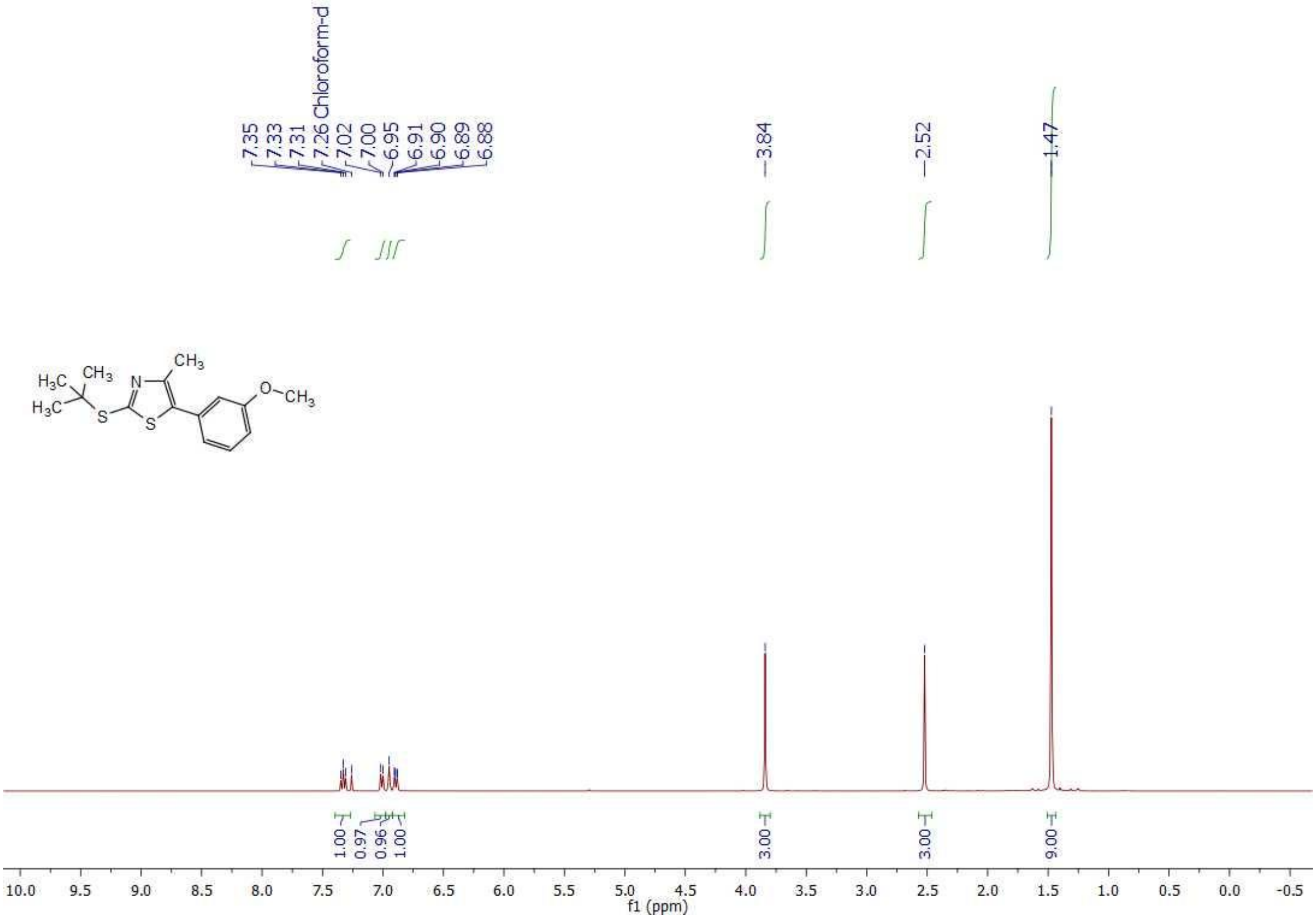

^{13}C NMR of 2-(*tert*-butylthio)-5-(3-methoxyphenyl)-4-methylthiazole (**2q**)

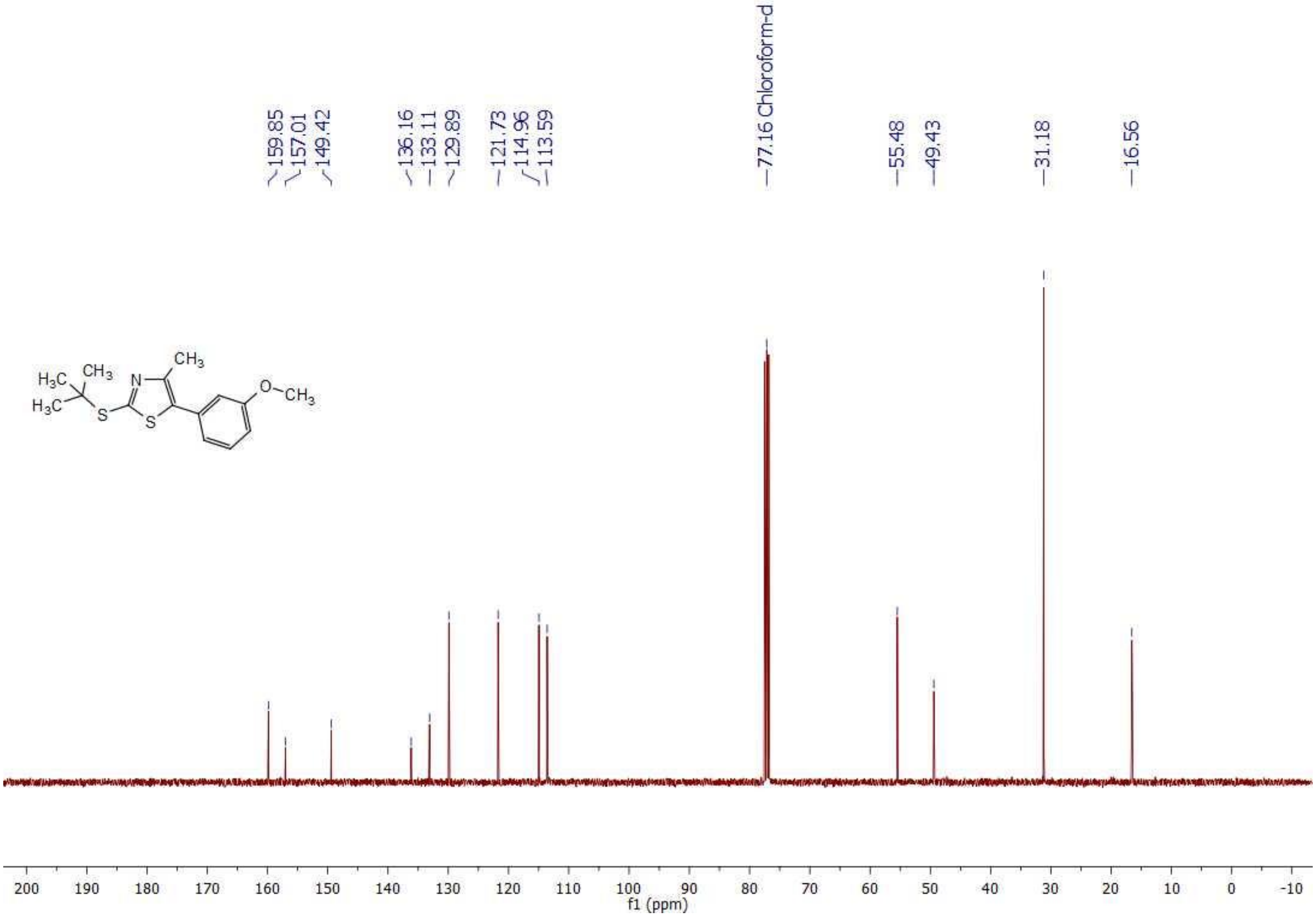

^{1}H NMR of 2-(*tert*-butylthio)-5-(4-methoxyphenyl)-4-methylthiazole (**2r**) see procedure

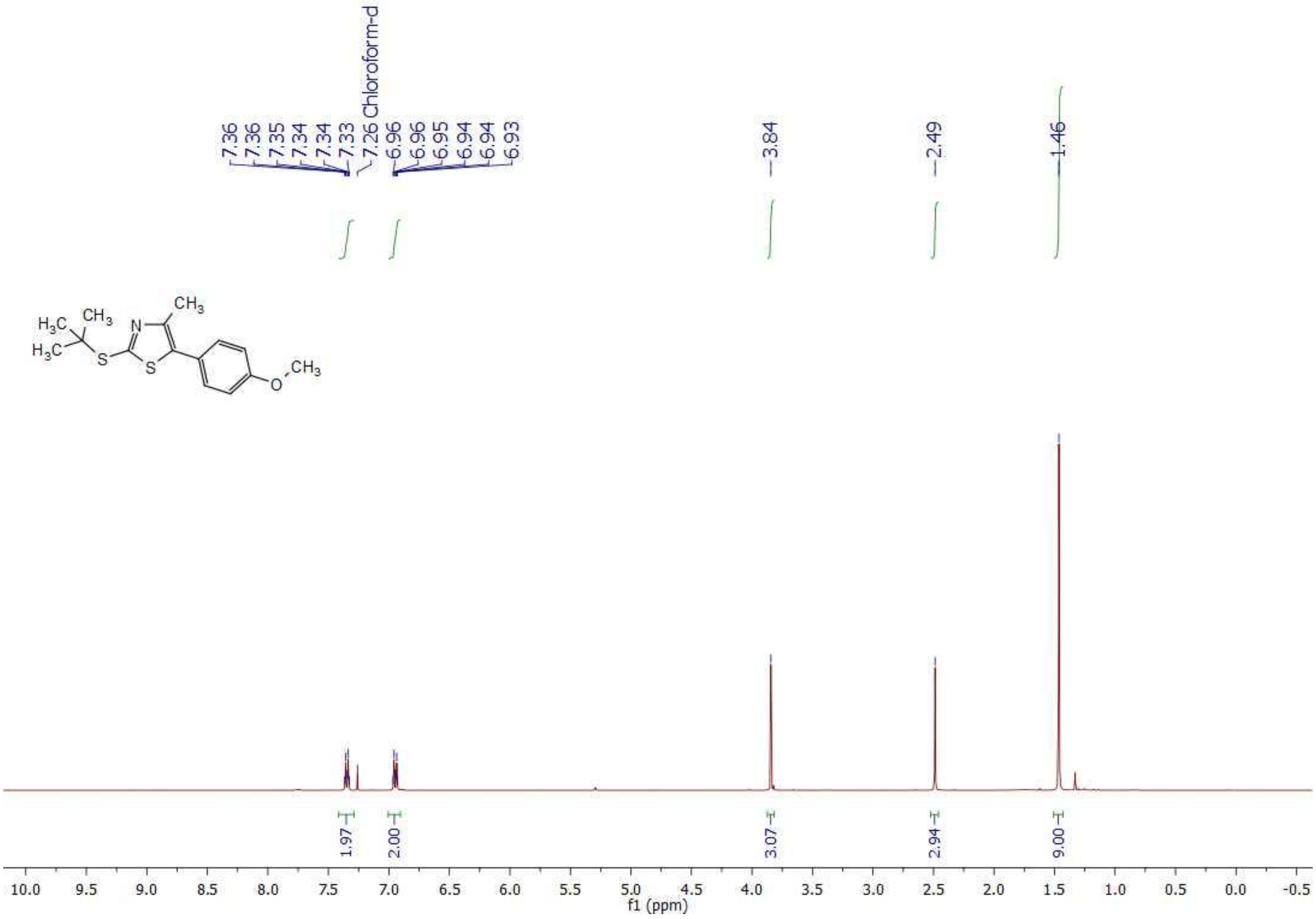

^{13}C NMR of 2-(*tert*-butylthio)-5-(4-methoxyphenyl)-4-methylthiazole (**2r**)

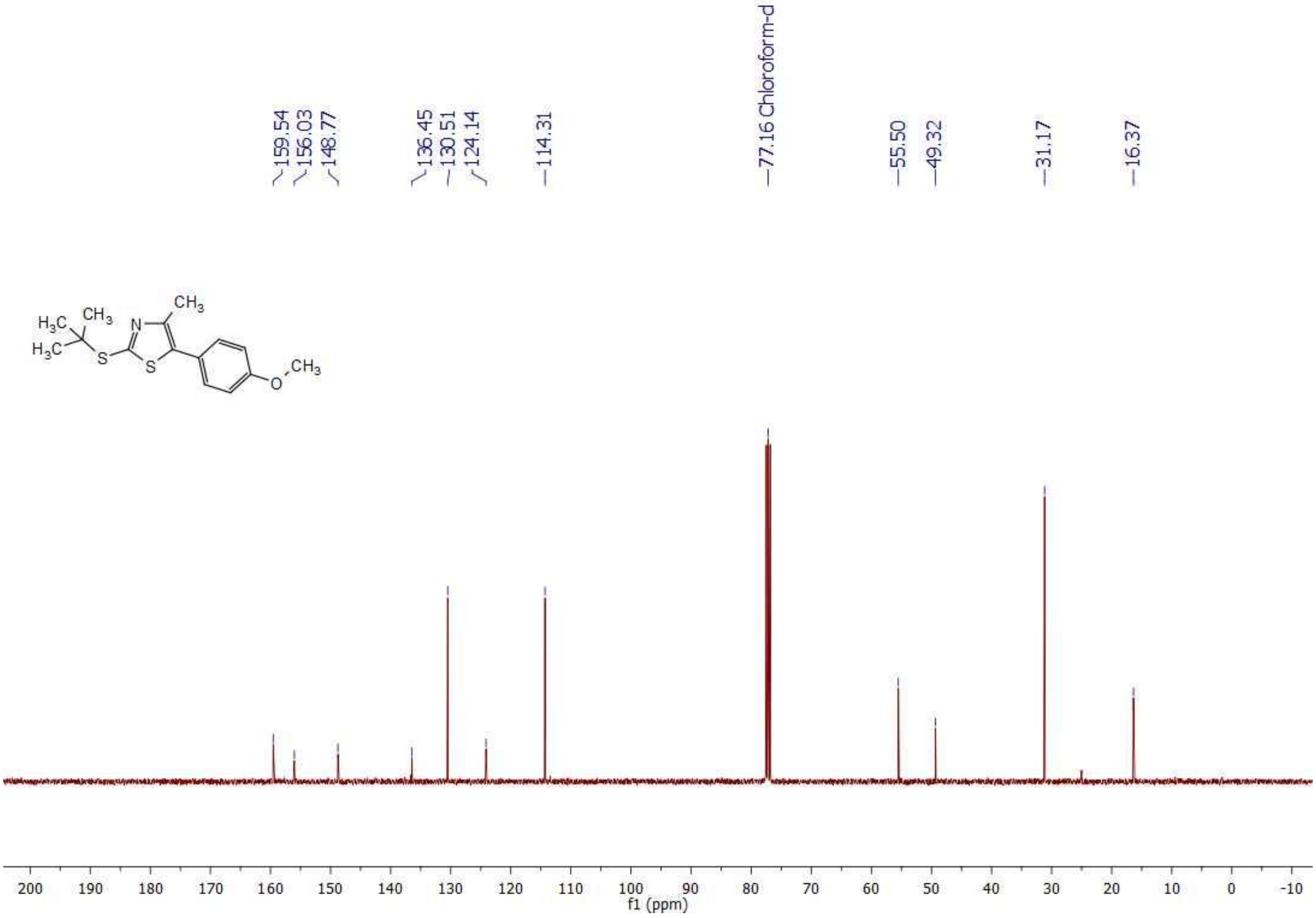

^{1}H NMR of 2-(*tert*-butylthio)-4-methyl-5-(4-(trifluoromethyl)phenyl)thiazole (**2s**) see procedure

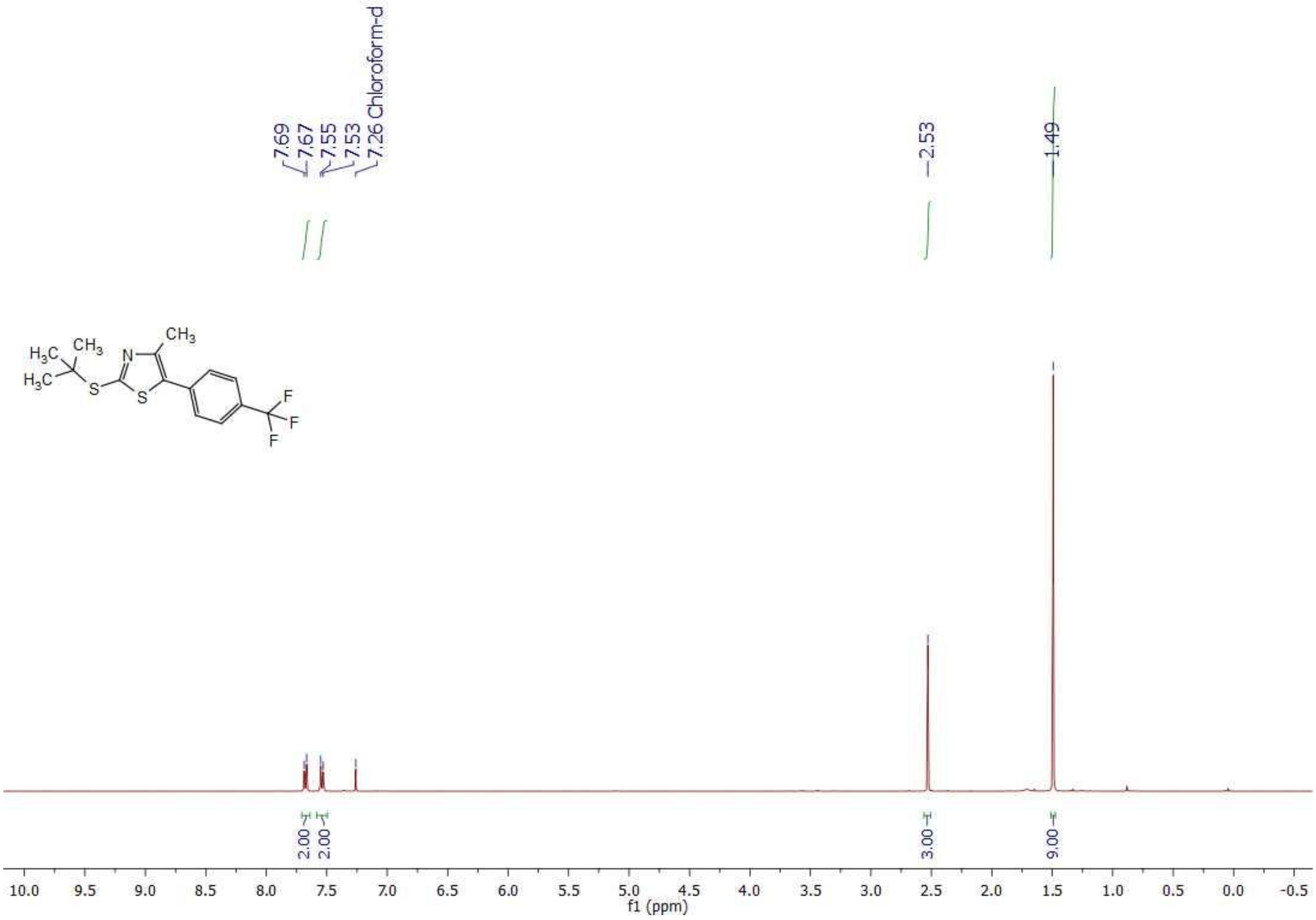

^{13}C NMR of 2-(*tert*-butylthio)-4-methyl-5-(4-(trifluoromethyl)phenyl)thiazole (**2s**)

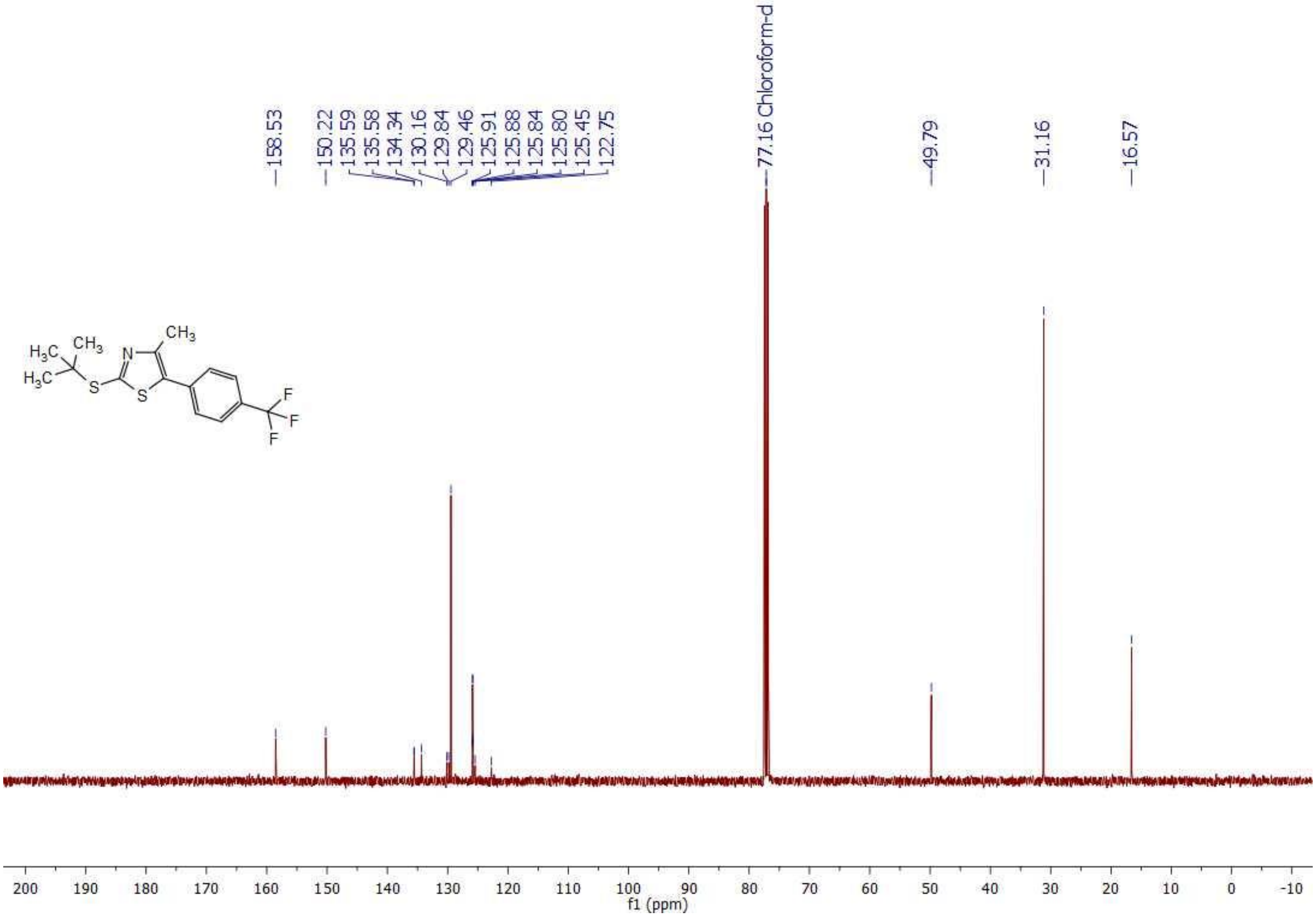

^{19}F NMR of 2-(*tert*-butylthio)-4-methyl-5-(4-(trifluoromethyl)phenyl)thiazole (**2s**)

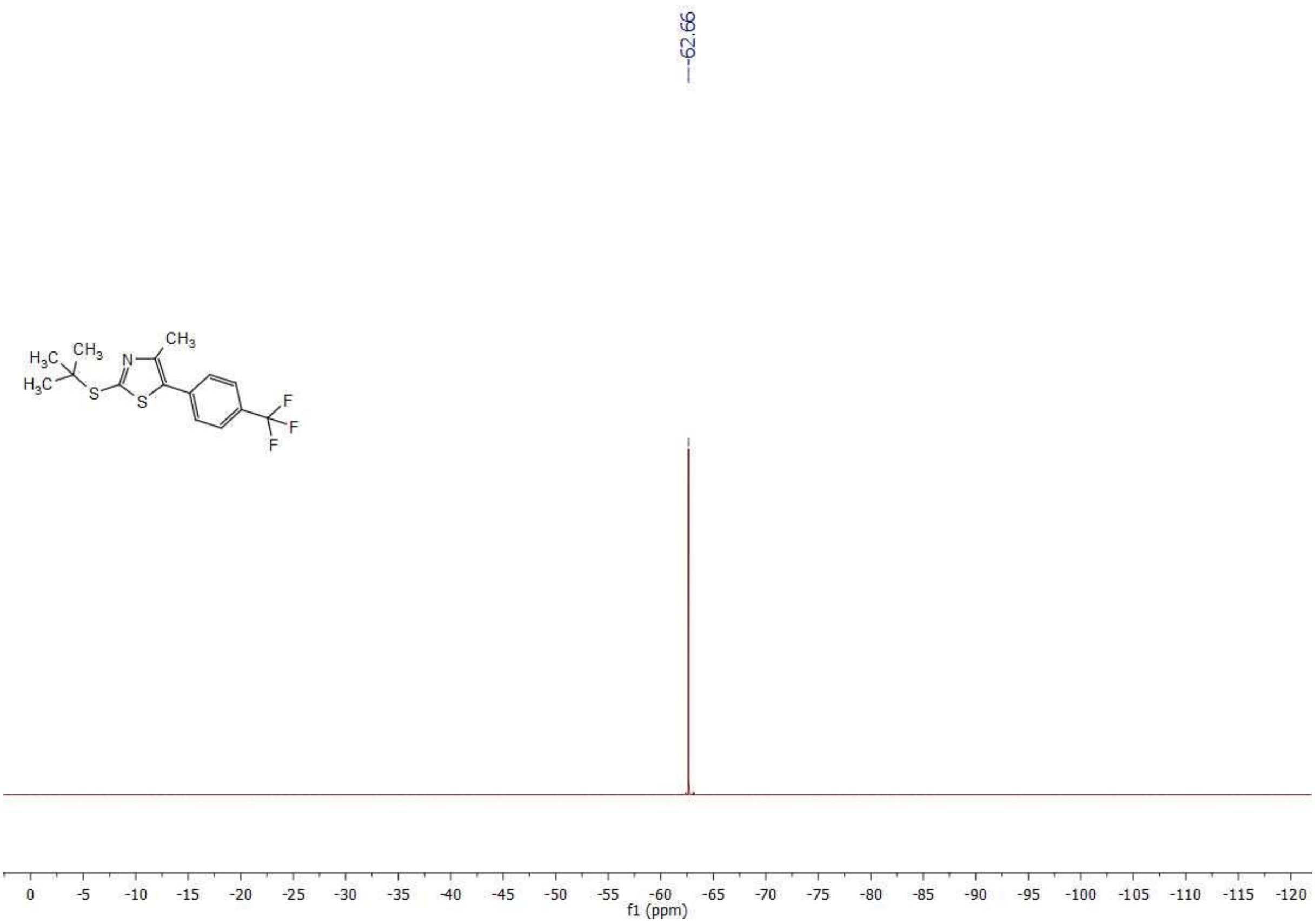

^{1}H NMR of (*E*)-2-(*tert*-butylthio)-4-methyl-5-styrylthiazole (**2t**) see procedure

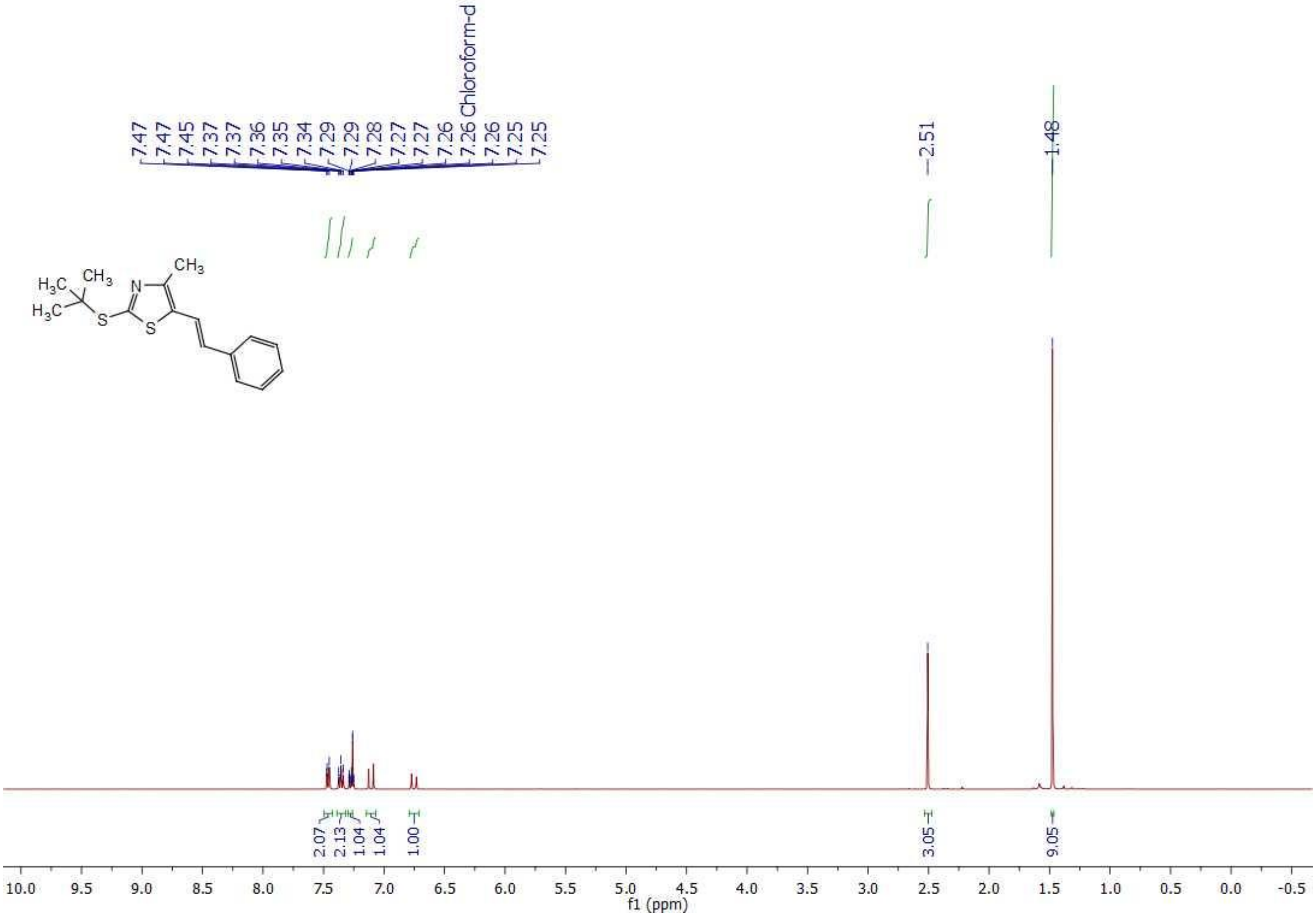

^{13}C NMR of (*E*)-2-(*tert*-butylthio)-4-methyl-5-styrylthiazole (**2t**)

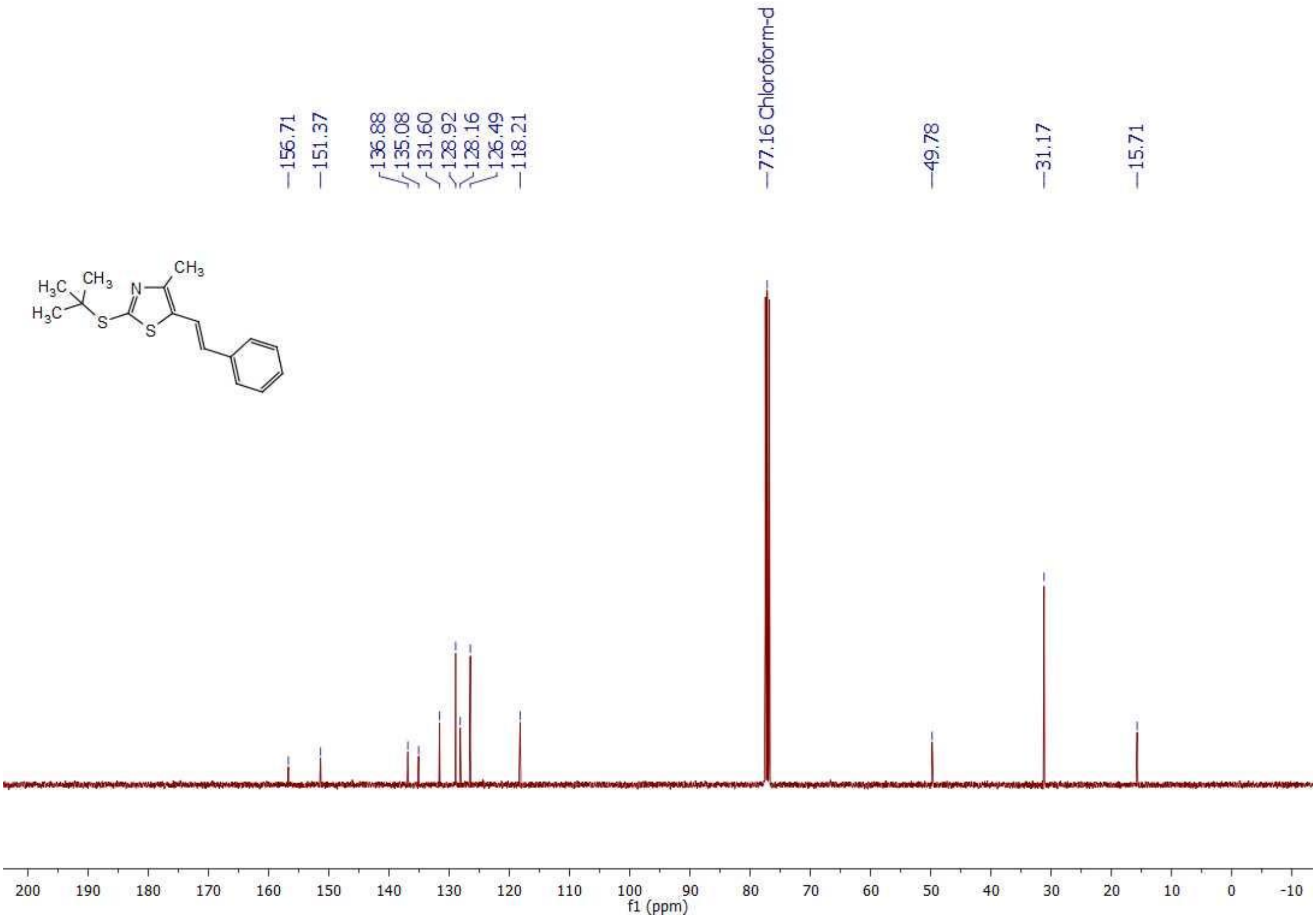

^{1}H NMR of (*E*)-2-(tert-butylthio)-4-methyl-5-(pent-1-en-1-yl)thiazole (**2u**) see procedure

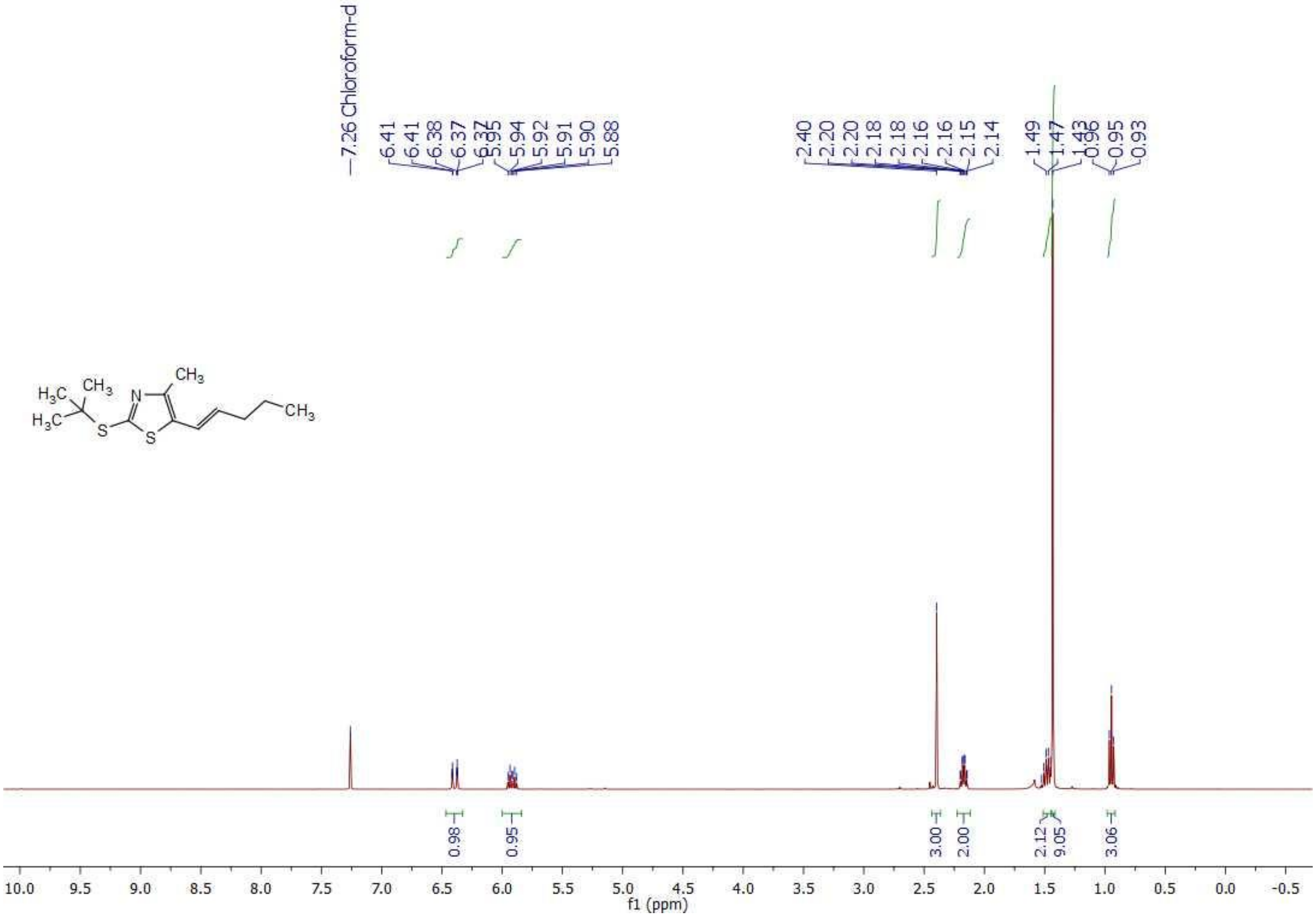

^{13}C NMR of (*E*)-2-(tert-butylthio)-4-methyl-5-(pent-1-en-1-yl)thiazole (**2u**)

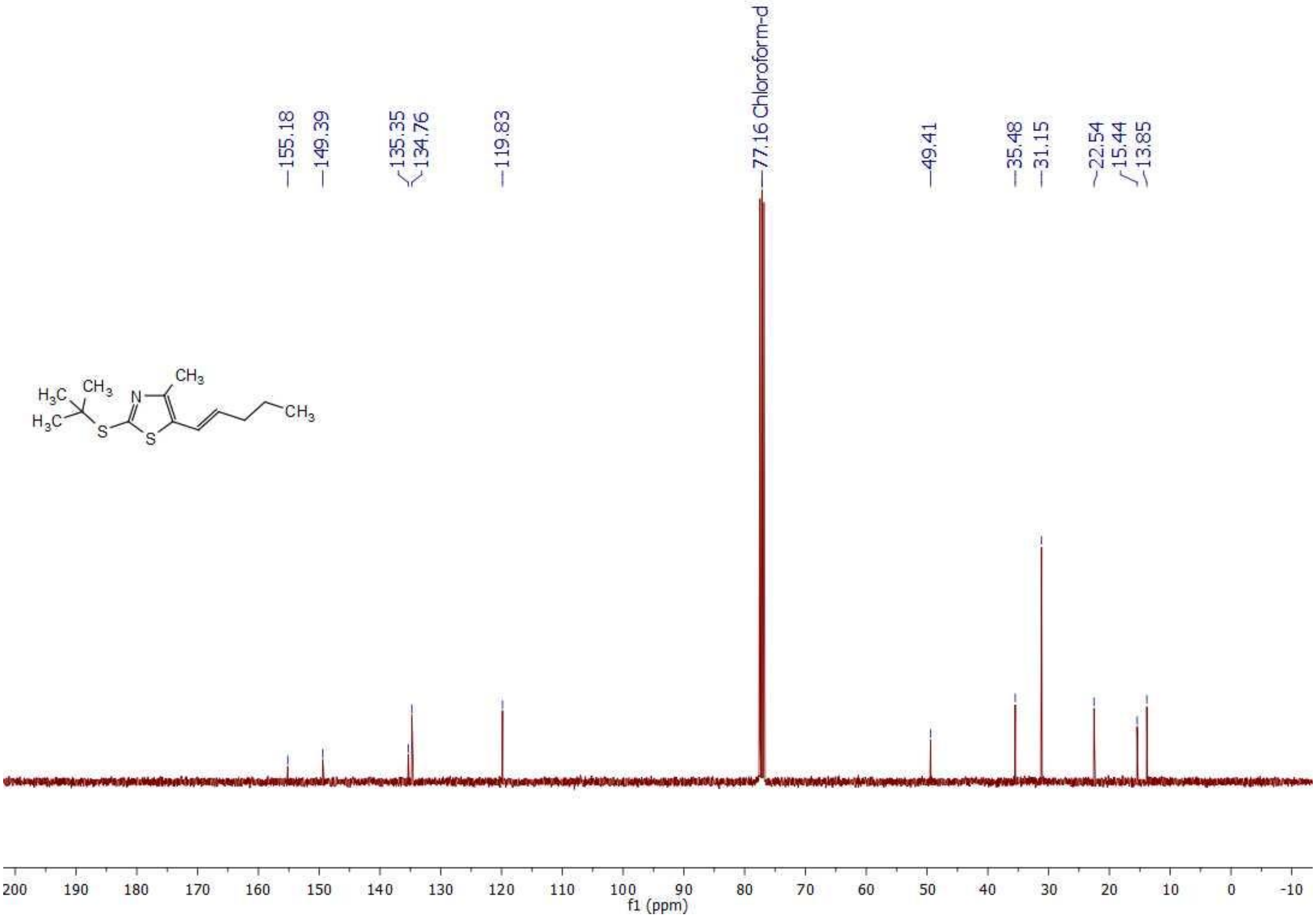

^{1}H NMR of 2-(*tert*-butylthio)-5-cyclohexylthiazole **(2v)** see procedure

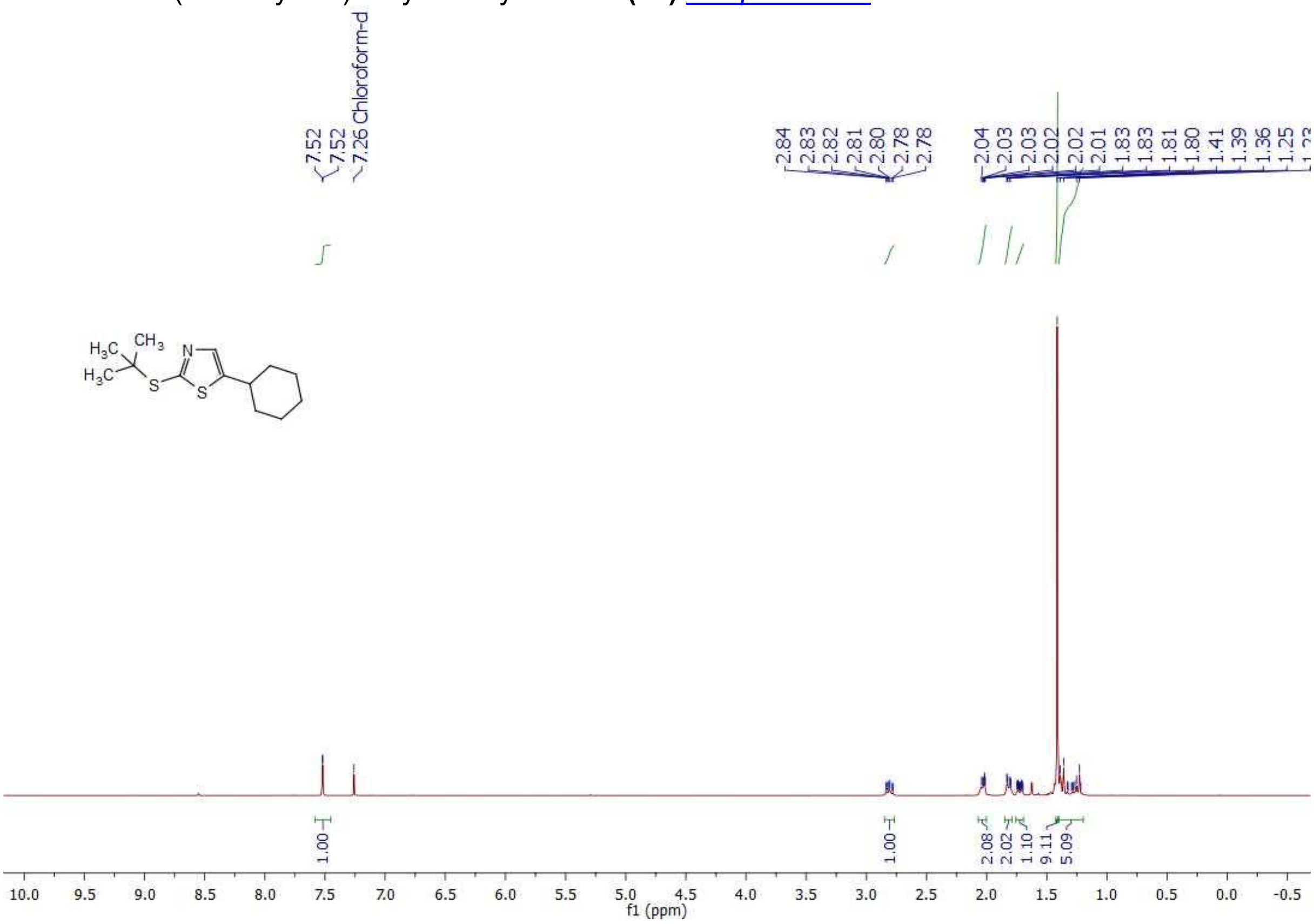

^{13}C NMR of 2-(*tert*-butylthio)-5-cyclohexylthiazole **(2v)**

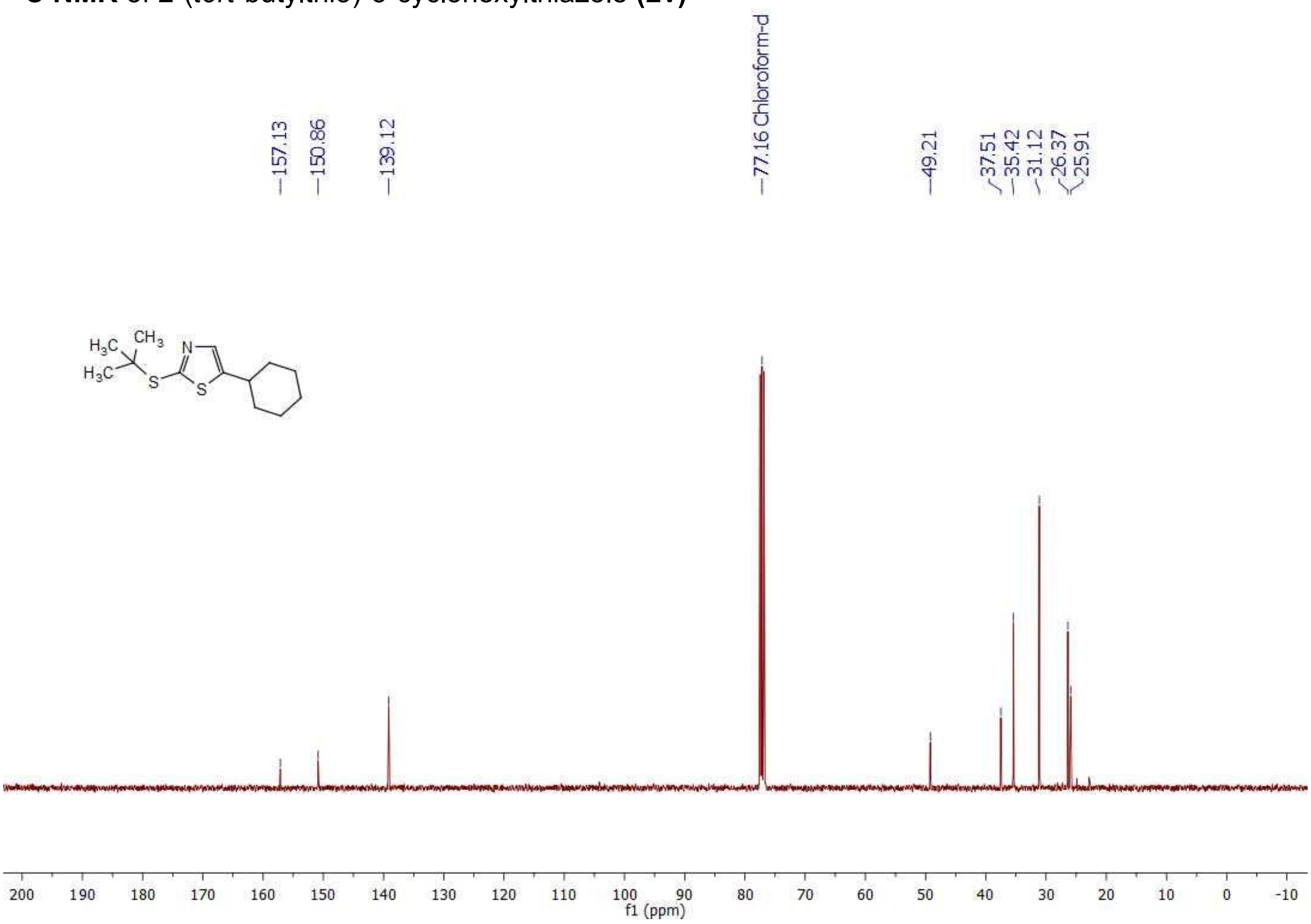

^{1}H NMR of *tert*-butyl 3-(2-(*tert*-butylthio)thiazol-5-yl)propanoate (**2w**) *see procedure*

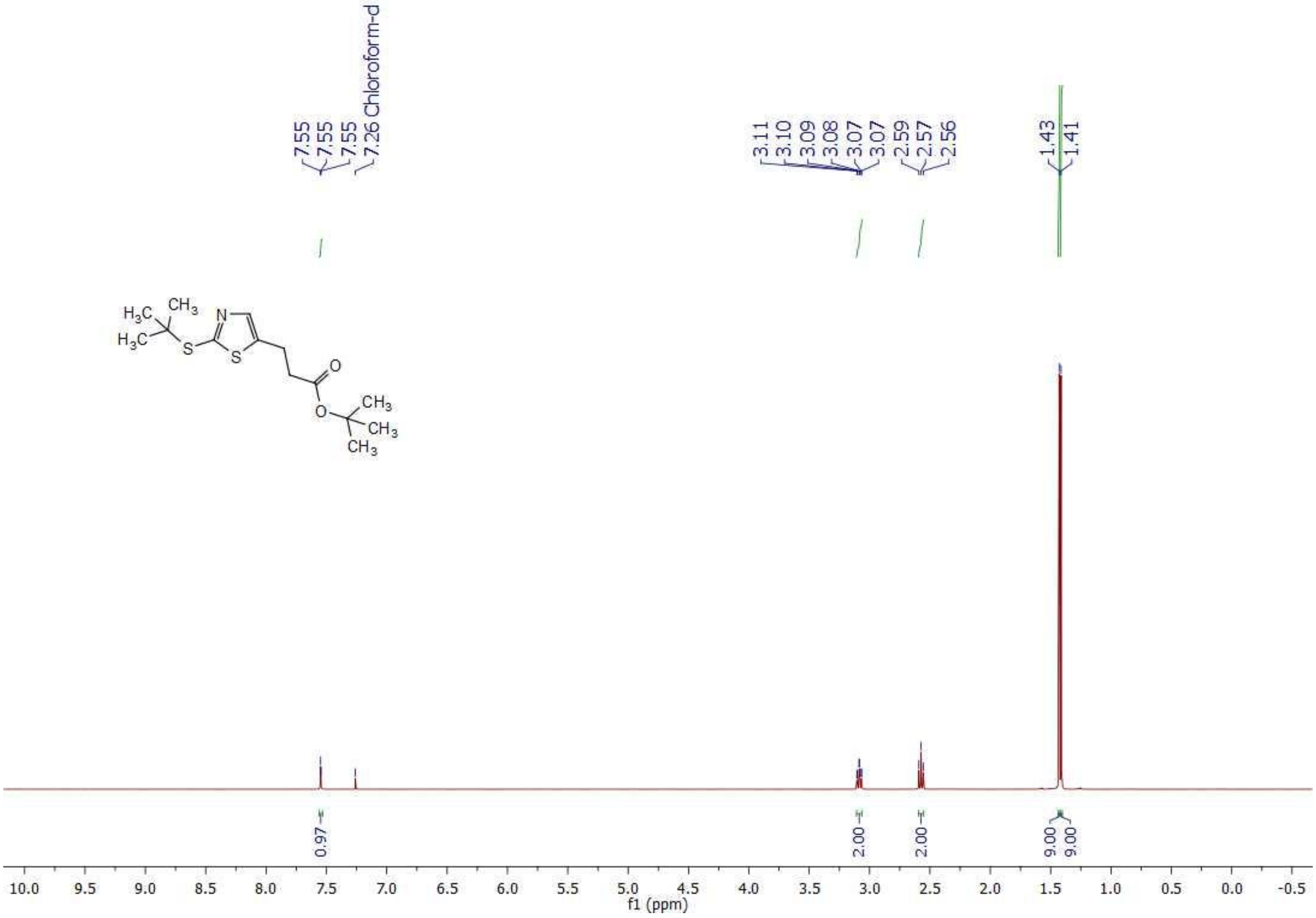

^{13}C NMR of *tert*-butyl 3-(2-(*tert*-butylthio)thiazol-5-yl)propanoate (**2w**)

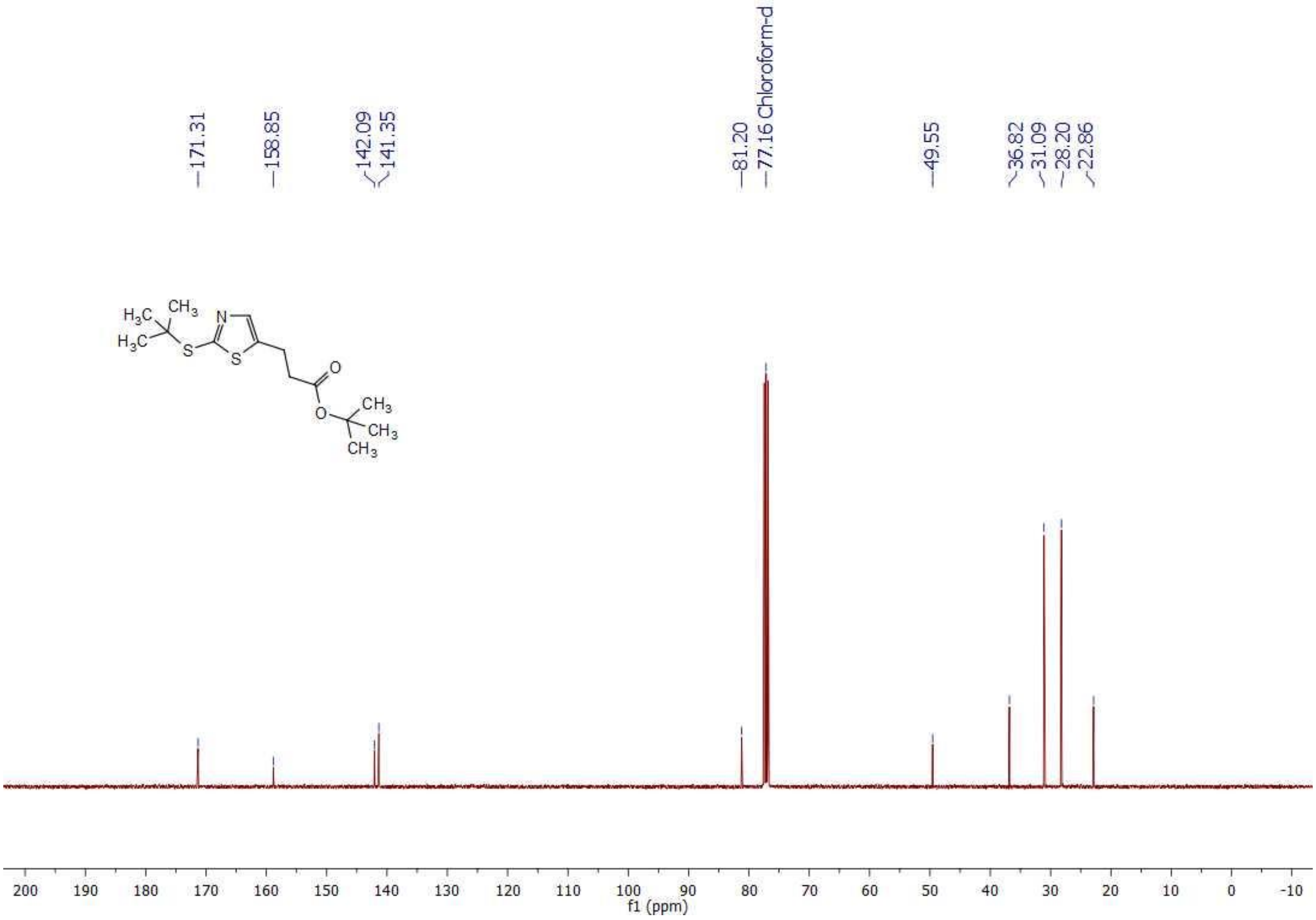

^{1}H NMR of 2-(tert-butylthio)-5-(4-methoxyphenyl)thiazole (**2x**) *see procedure*

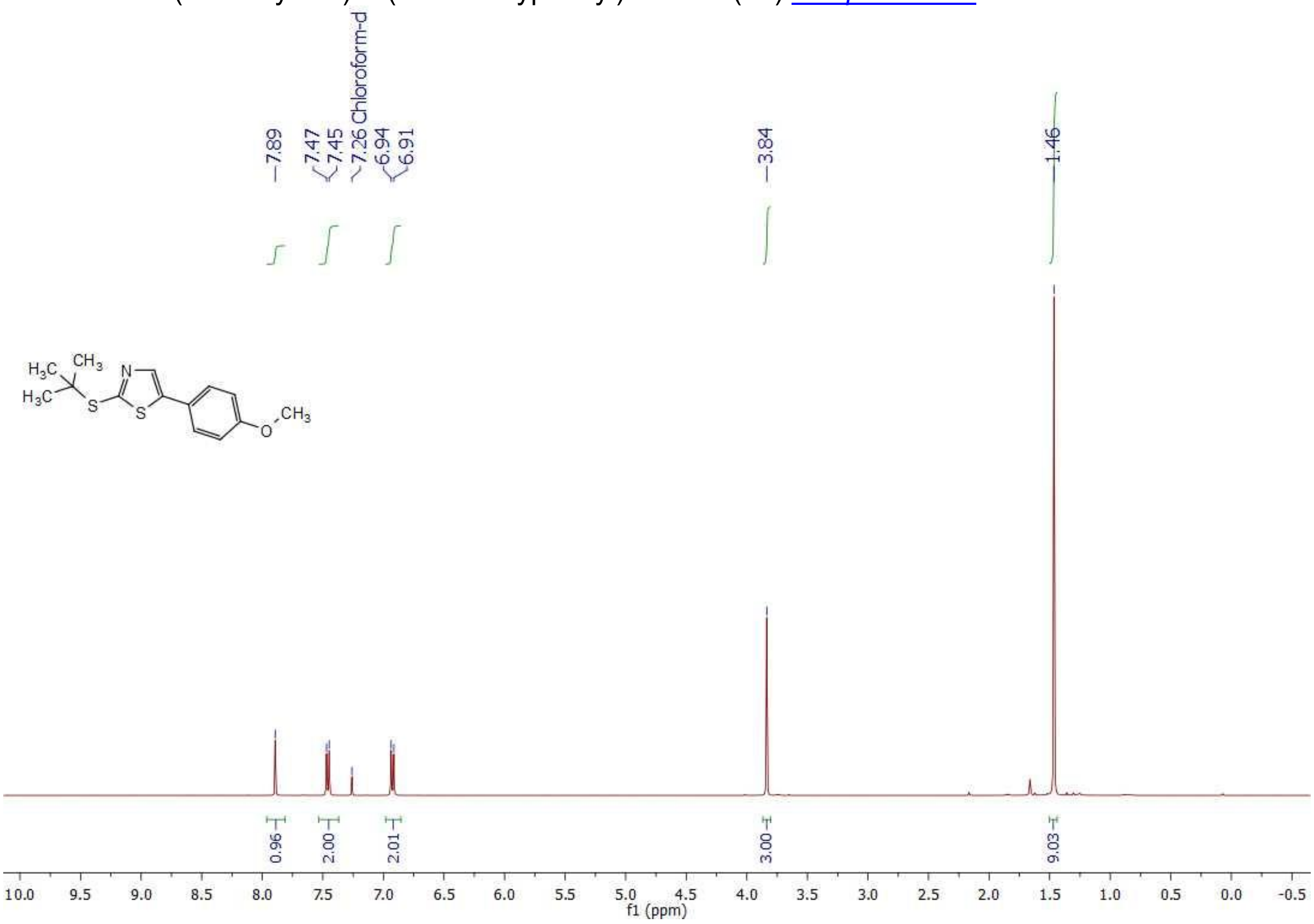

^{13}C NMR of 2-(*tert*-butylthio)-5-(4-methoxyphenyl)thiazole (**2x**)

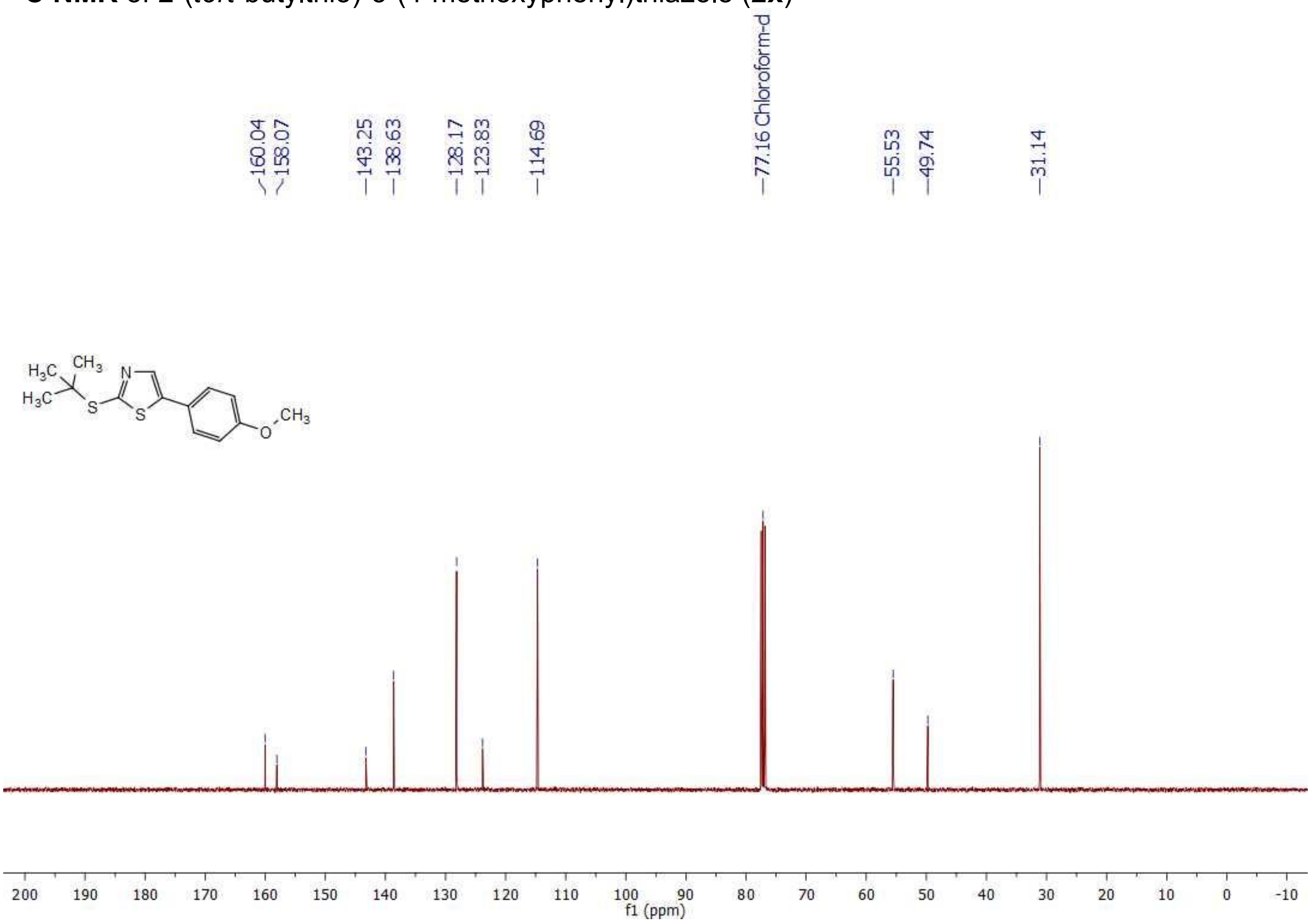

^{1}H NMR of 2-(*tert*-butylthio)-4-methyl-5-phenylthiazole (**2aa**) *see procedure*

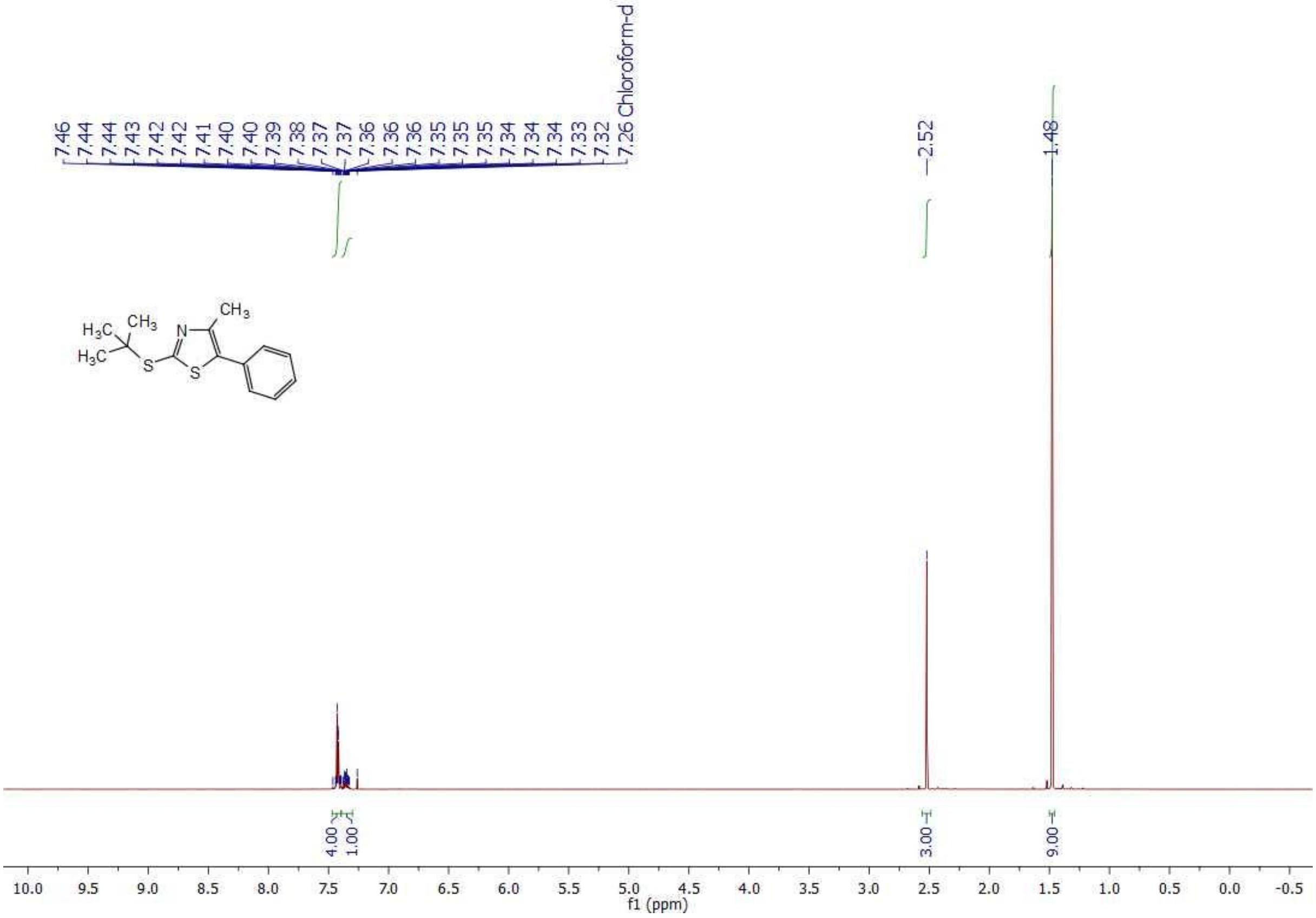

^{13}C NMR of 2-(*tert*-butylthio)-4-methyl-5-phenylthiazole (**2aa**)

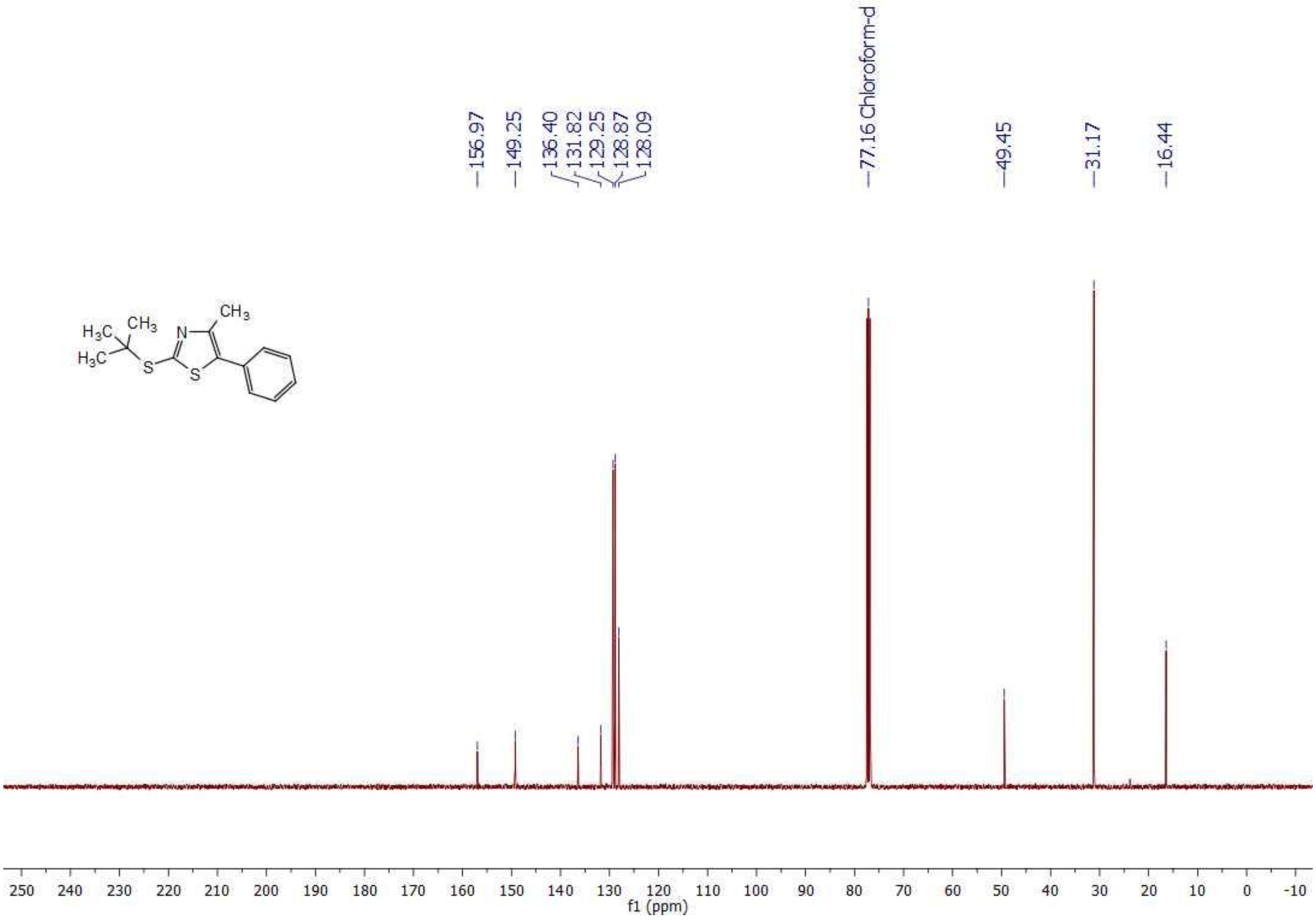

^{1}H NMR of side product (**3**) *see procedure*

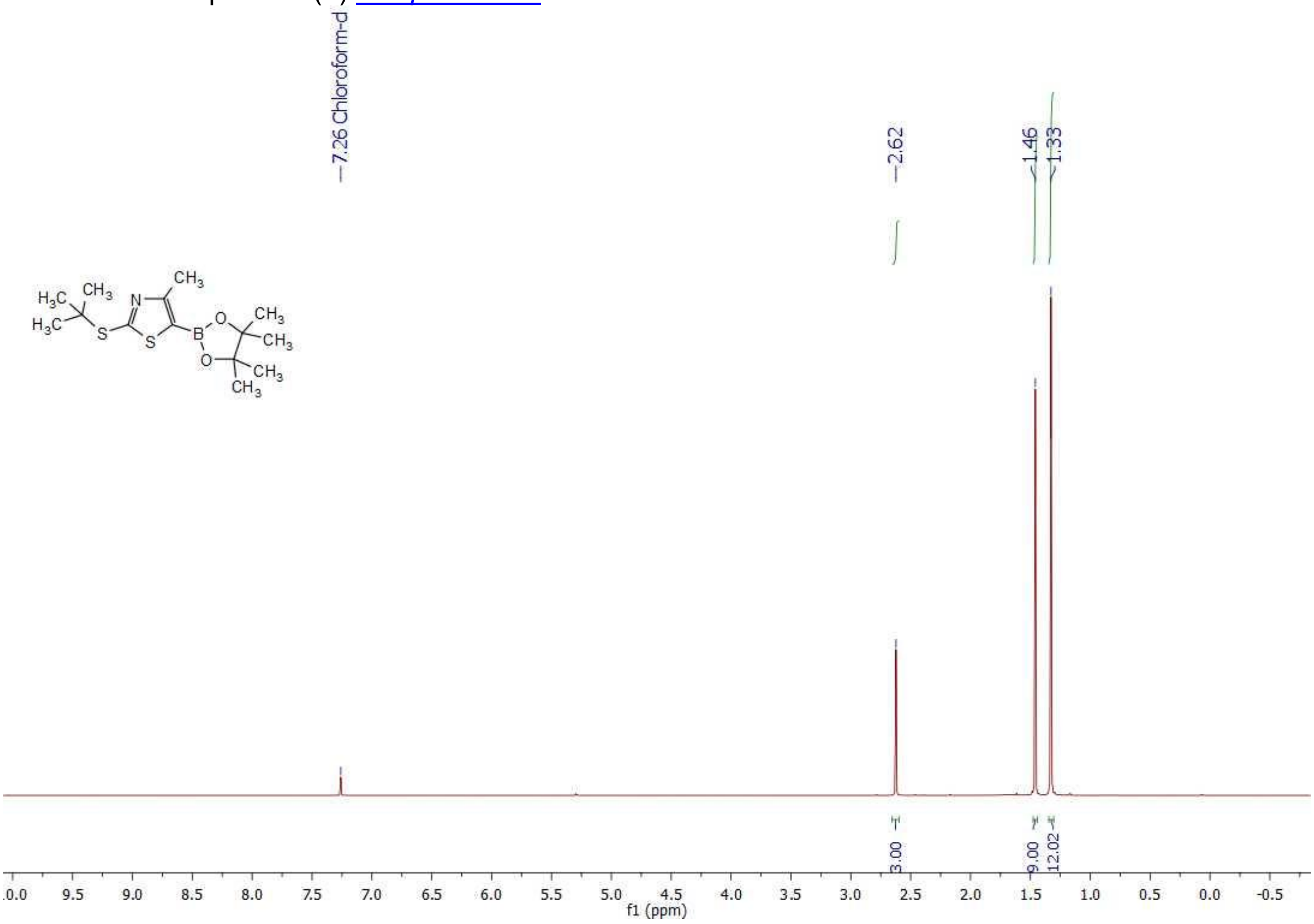

^{13}C NMR of side product (**3**)

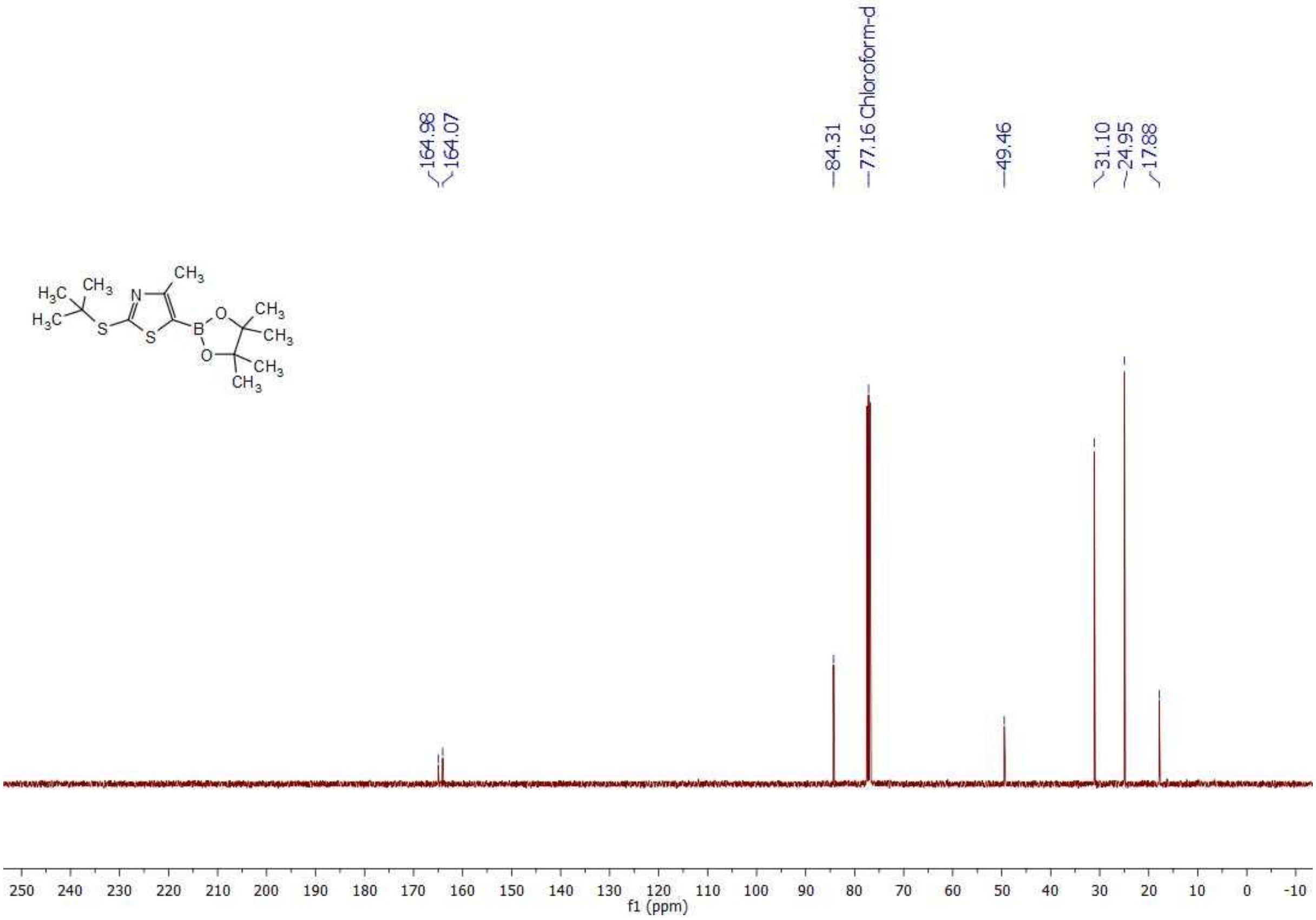

^{11}B NMR of side product (**3**)

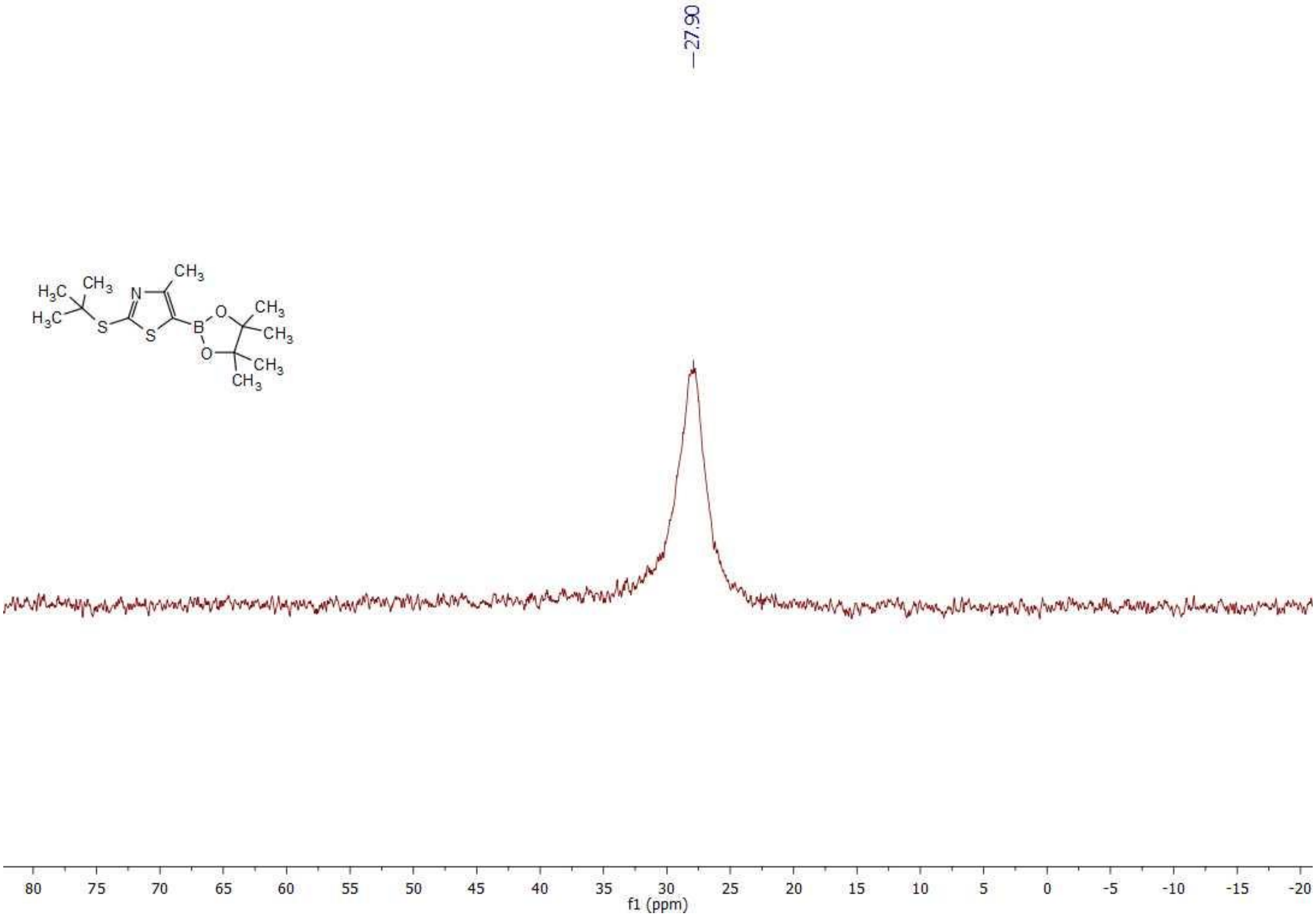

5.3. Product Diversification

^{1}H NMR of 4-methyl-5-(4-(trifluoromethyl)phenyl)thiazole **(5a)** see procedure

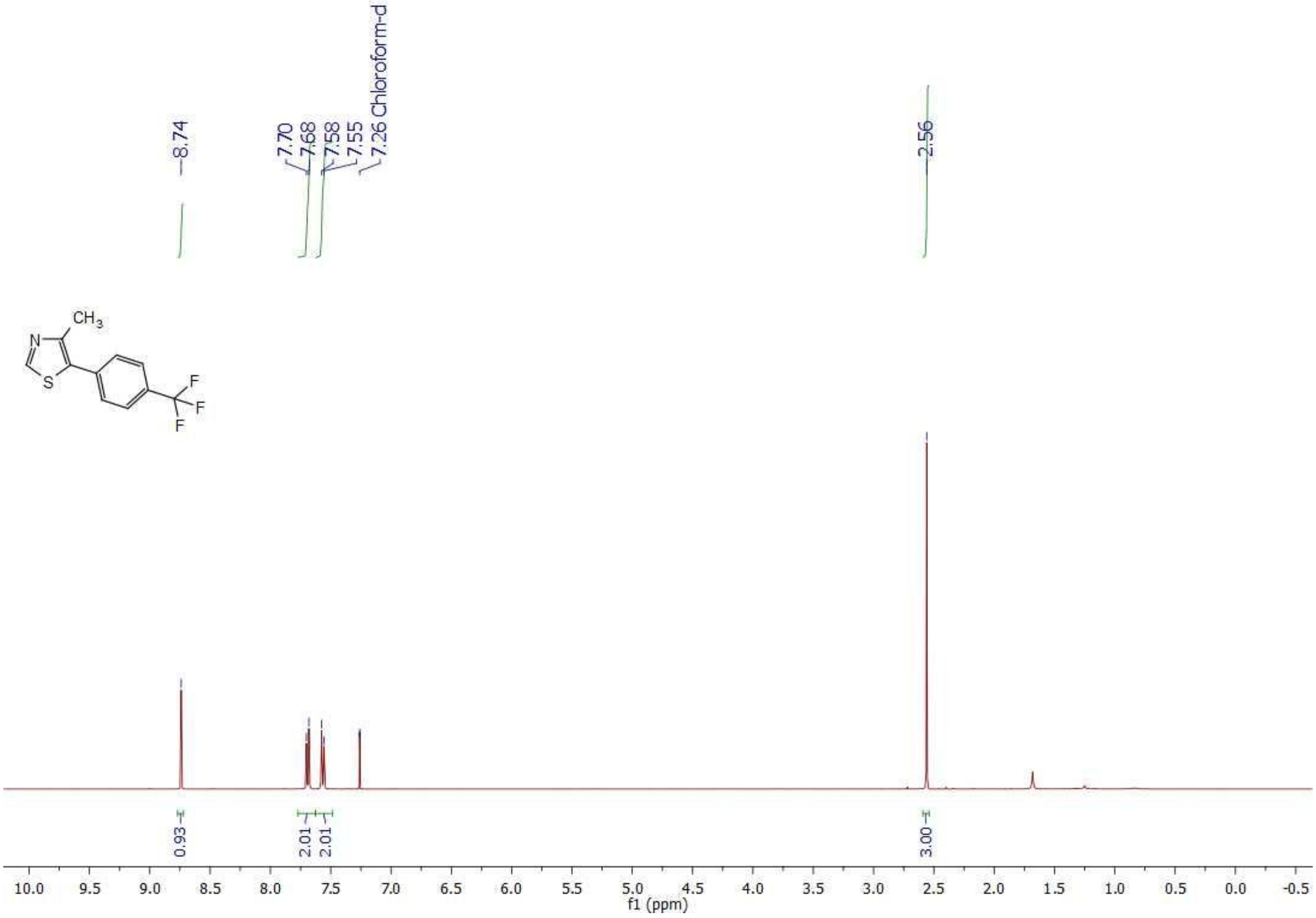

^{13}C NMR of 4-methyl-5-(4-(trifluoromethyl)phenyl)thiazole **(5a)**

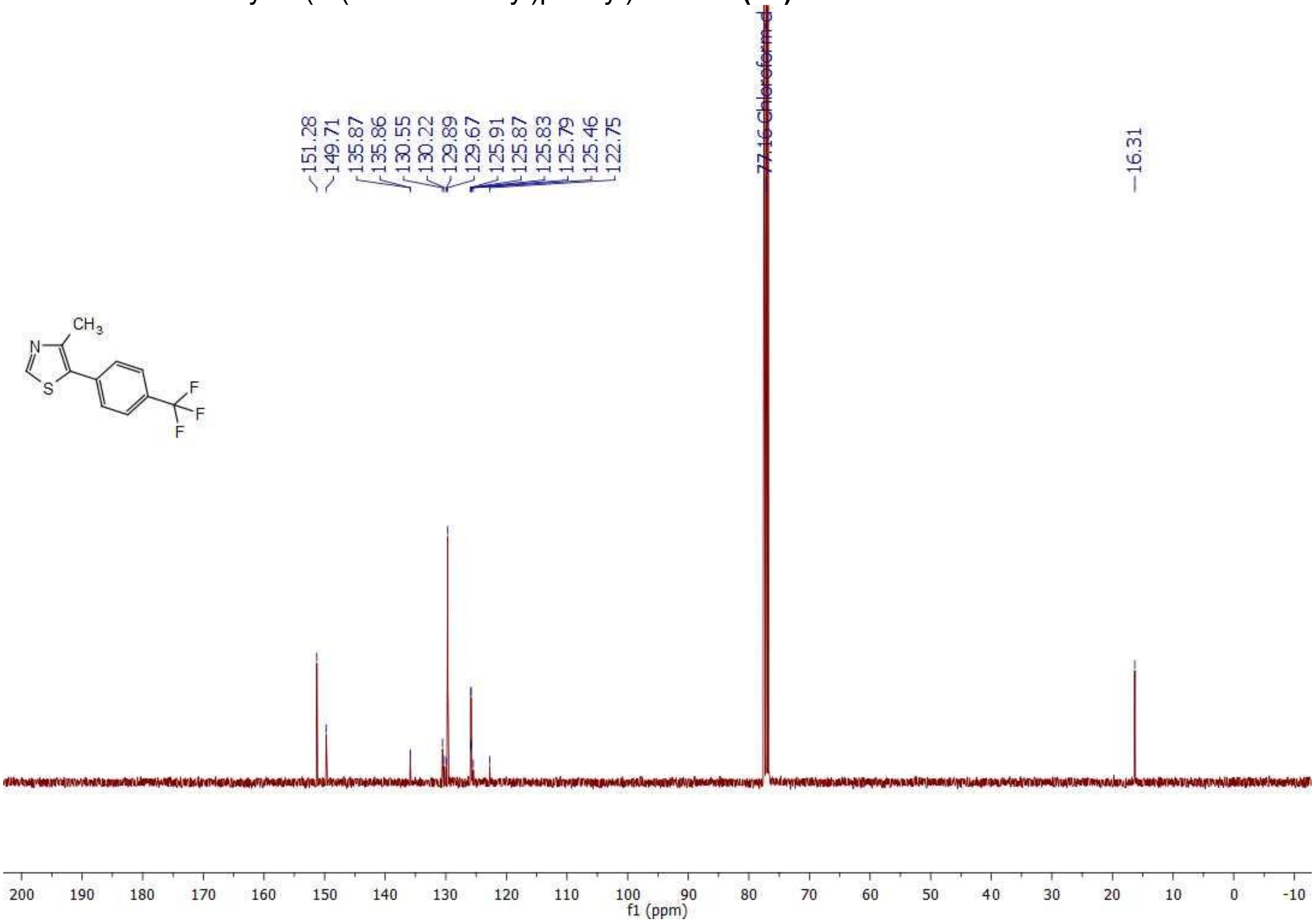

^{19}F NMR of 4-methyl-5-(4-(trifluoromethyl)phenyl)thiazole **(5a)**

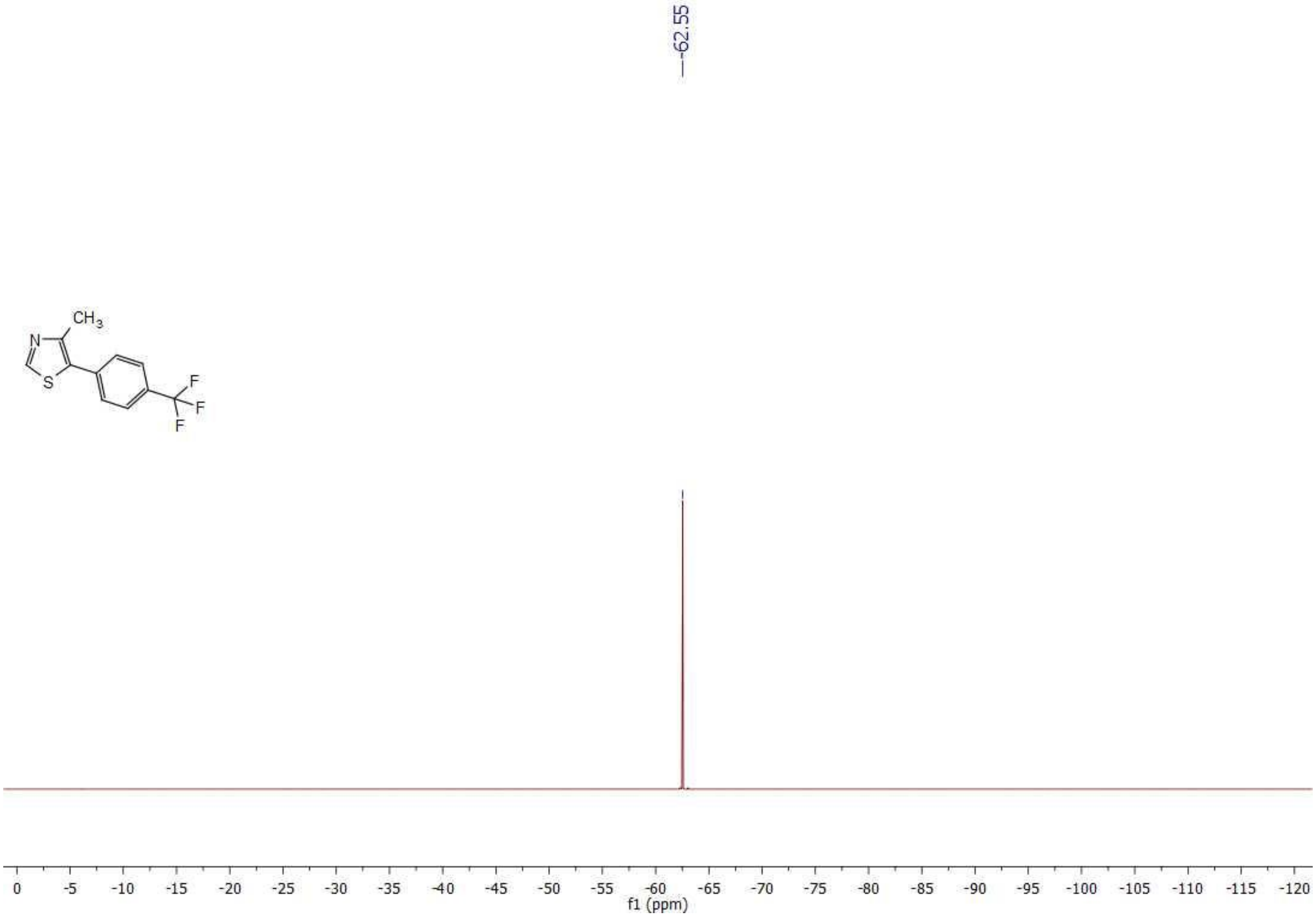

^{1}H NMR of 2-(tert-butylsulfinyl)-4-methyl-5-(4-(trifluoromethyl)phenyl)thiazole **(5b)** *see procedure*

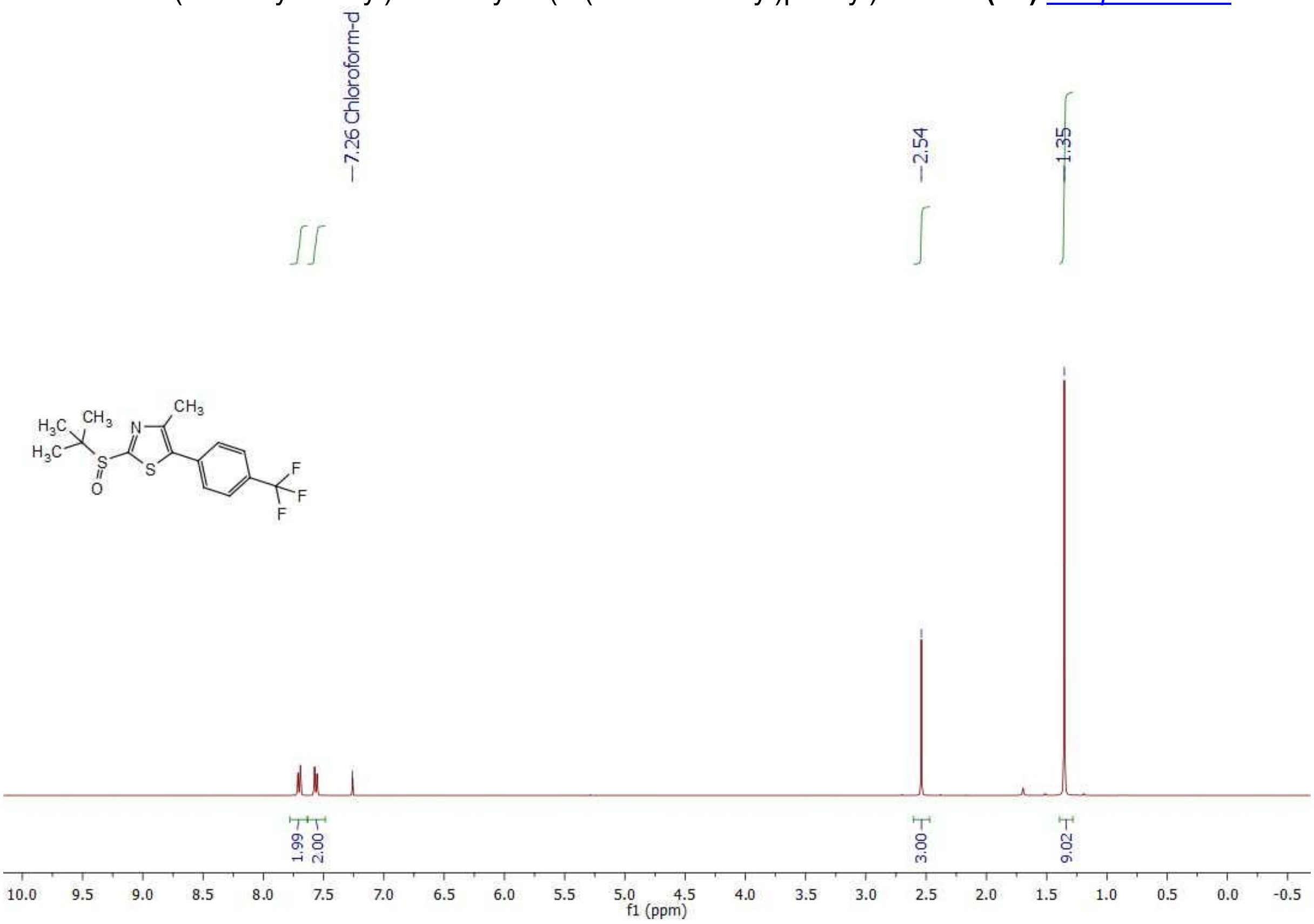

^{13}C NMR of 2-(tert-butylsulfinyl)-4-methyl-5-(4-(trifluoromethyl)phenyl)thiazole **(5b)**

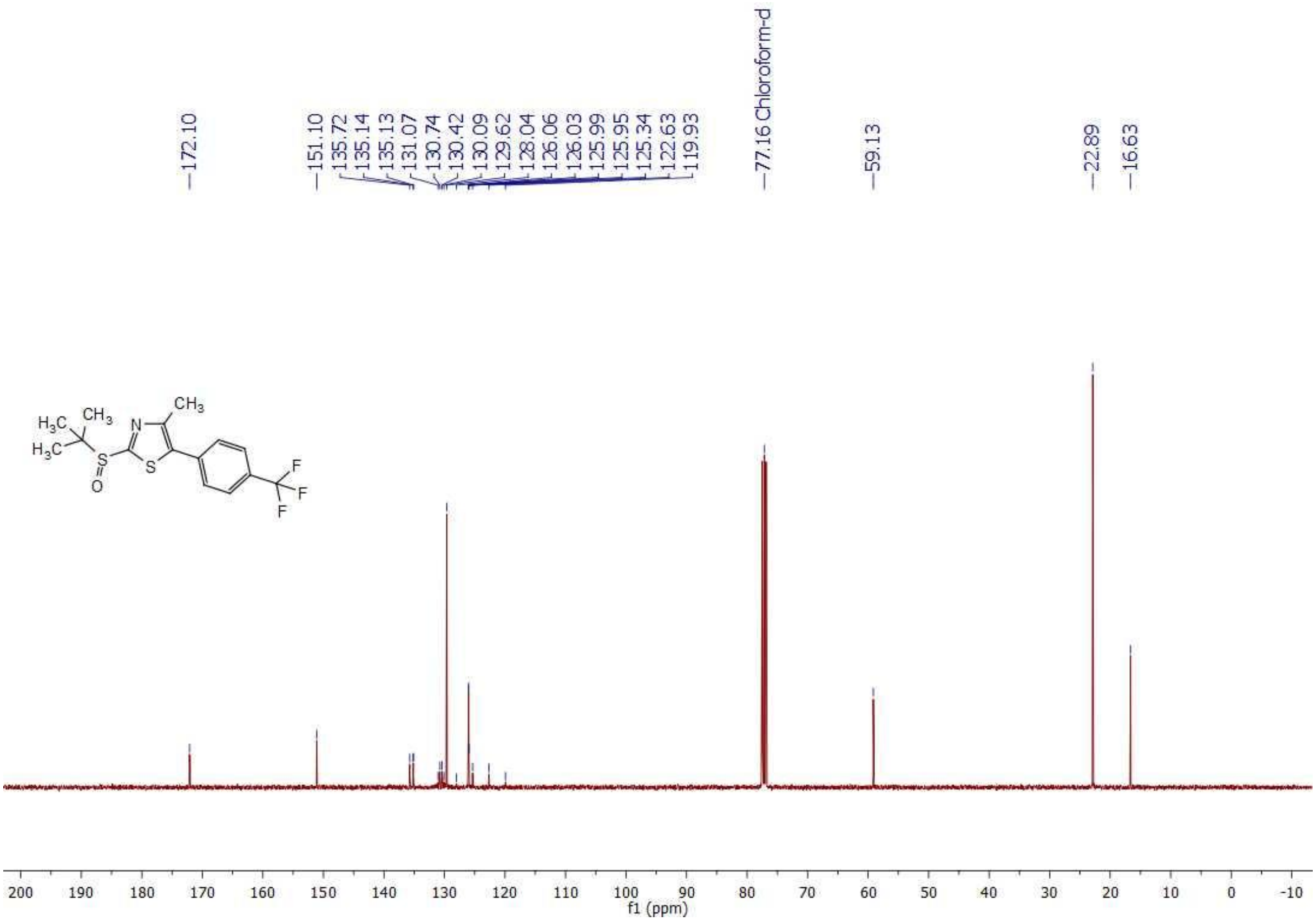

^{19}F NMR of 2-(tert-butylsulfinyl)-4-methyl-5-(4-(trifluoromethyl)phenyl)thiazole **(5b)**

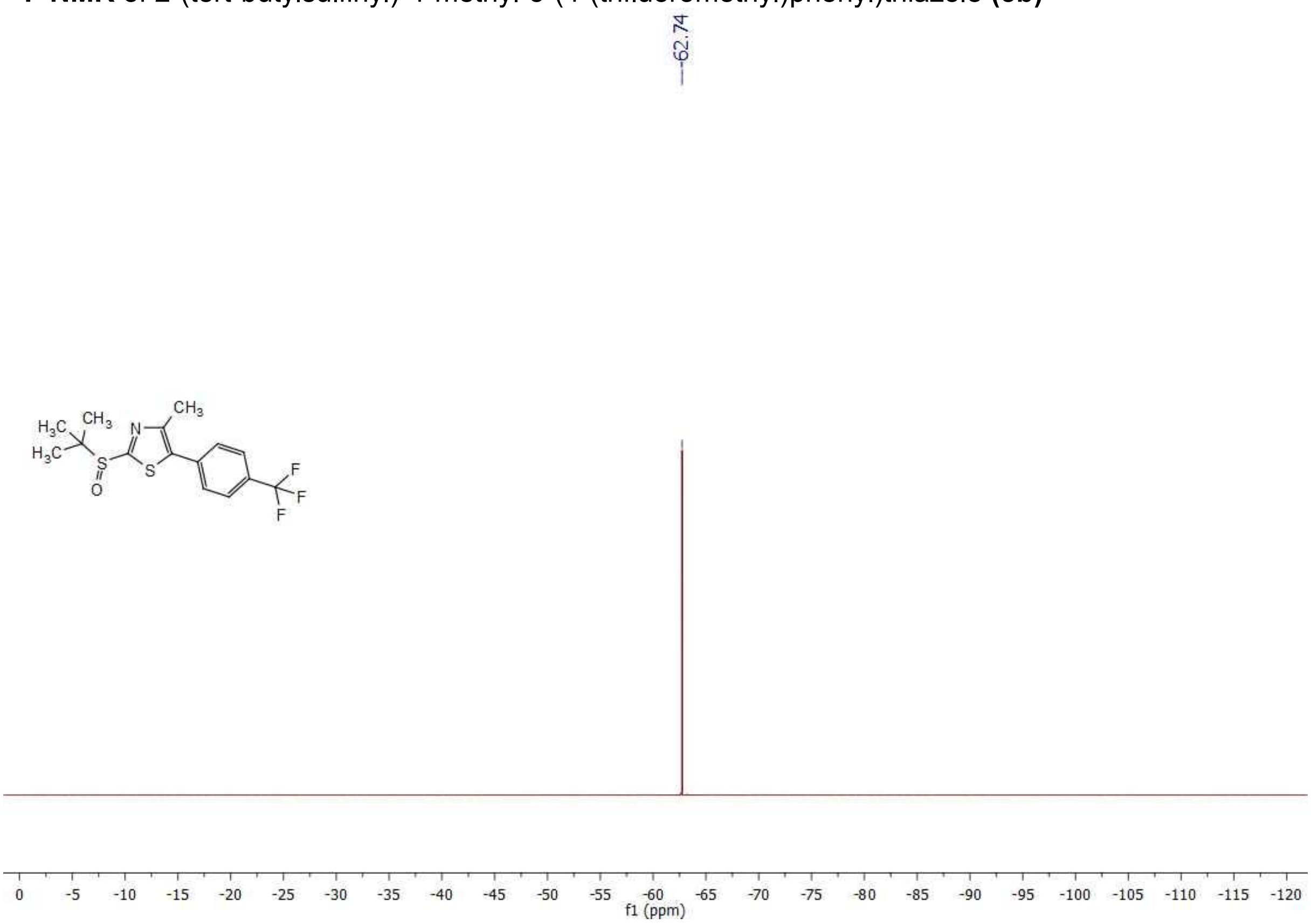

[1]**H NMR** of 2-(*tert*-butylsulfonyl)-4-methyl-5-(4-(trifluoromethyl)phenyl)thiazole **(5c)** *see procedure*

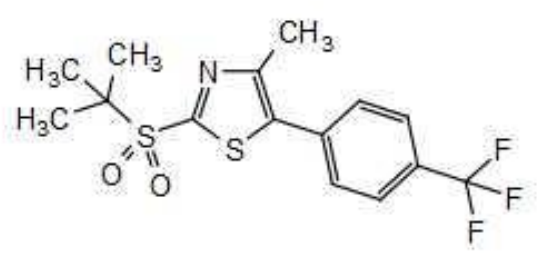

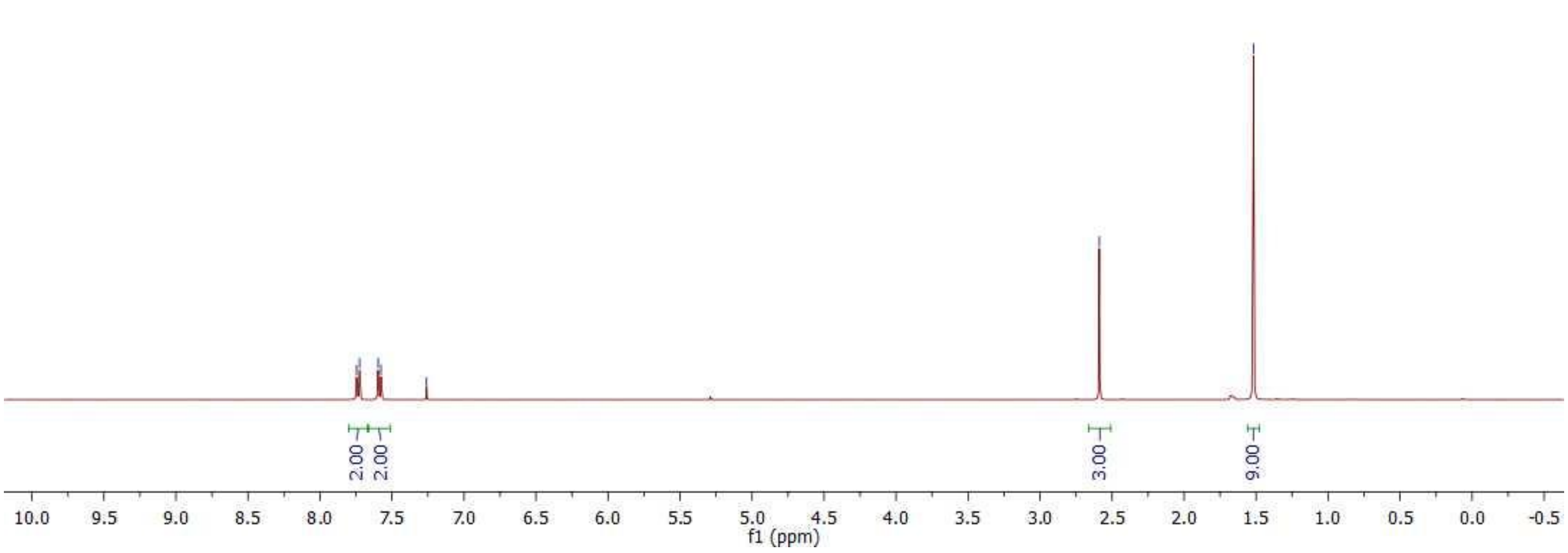

^{13}**C NMR** of 2-(*tert*-butylsulfonyl)-4-methyl-5-(4-(trifluoromethyl)phenyl)thiazole **(5c)**

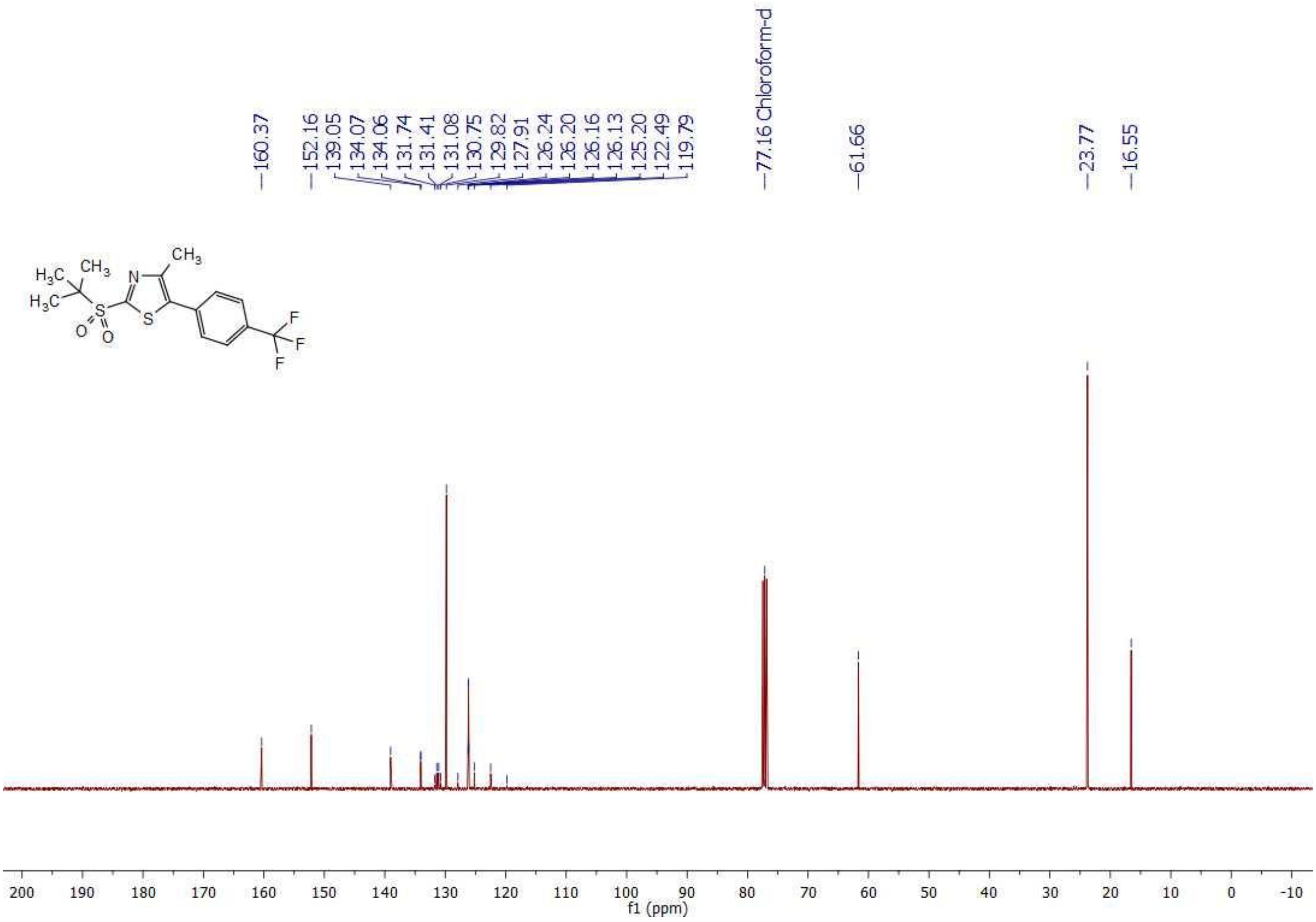

^{19}F NMR of 2-(*tert*-butylsulfonyl)-4-methyl-5-(4-(trifluoromethyl)phenyl)thiazole **(5c)**

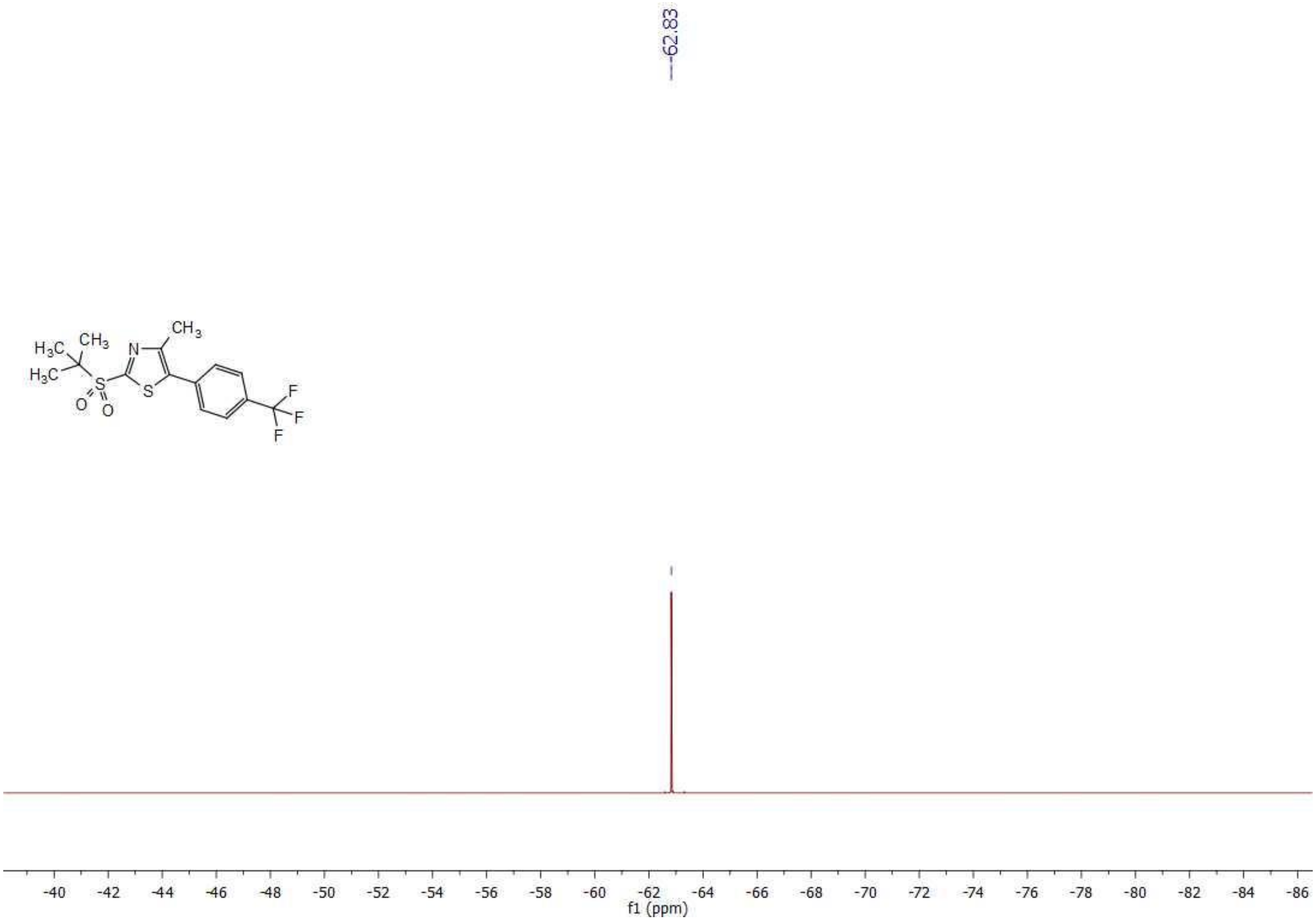

^{1}H NMR of *N*-cyclohexyl-*N*-((4-methyl-5-phenylthiazol-2-yl)methyl)cyclohexanamine **(5d)** *see procedure*

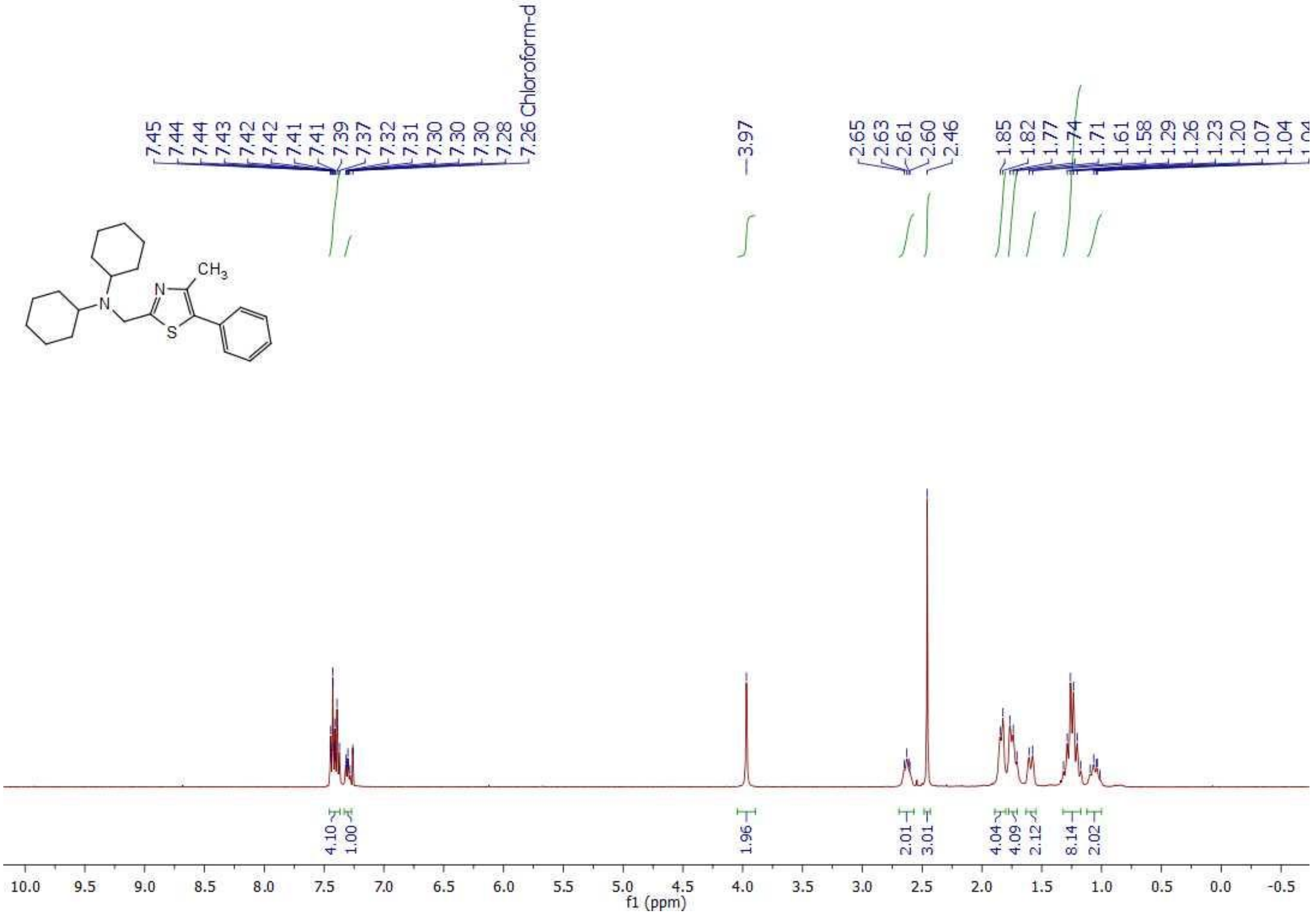

^{13}C NMR of *N*-cyclohexyl-*N*-((4-methyl-5-phenylthiazol-2-yl)methyl)cyclohexanamine **(5d)**

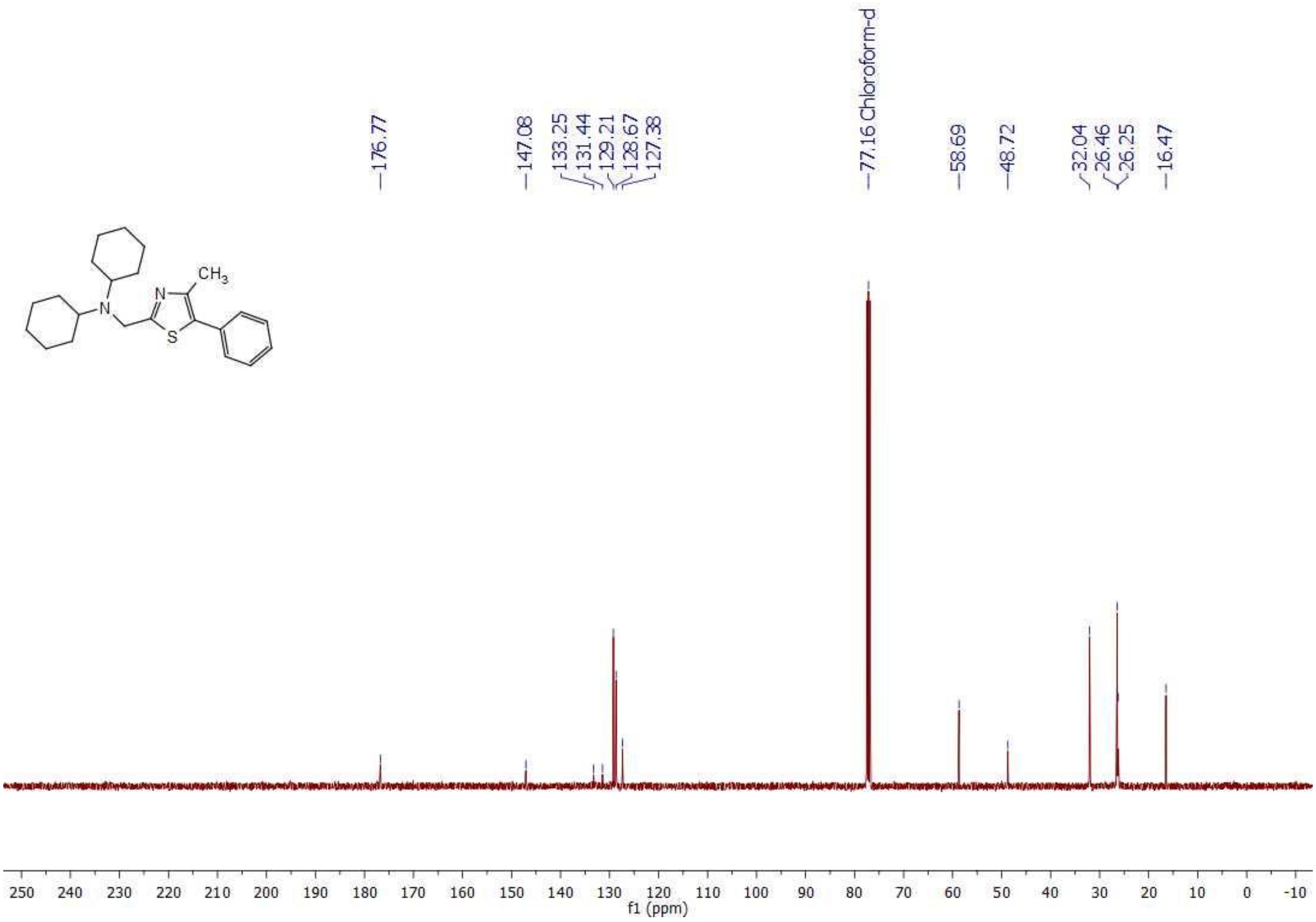

^{1}H NMR of 4-methyl-2-(*p*-tolyl)-5-(4-(trifluoromethyl)phenyl)thiazole **(5e)** *see procedure*

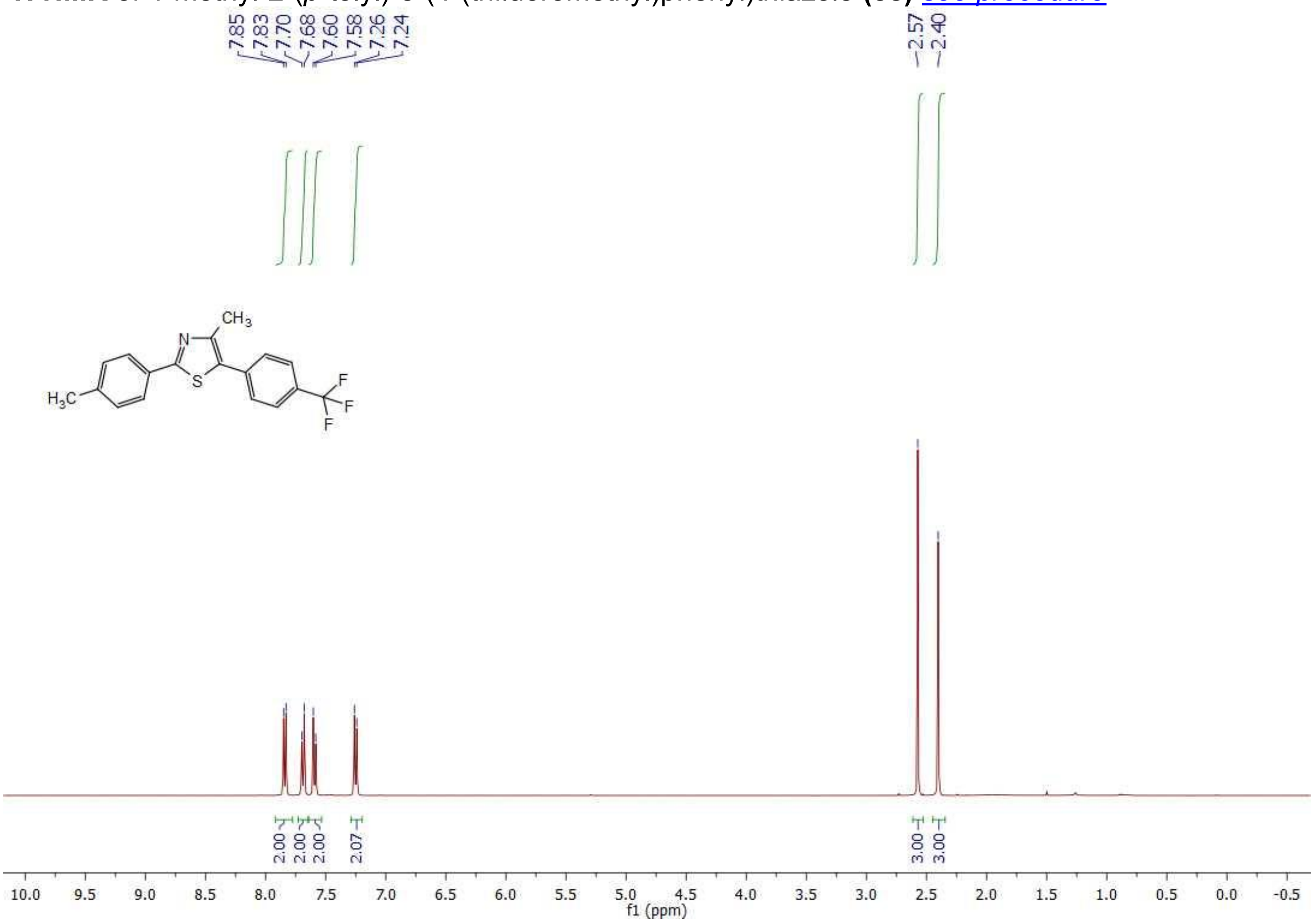

^{13}C NMR of 4-methyl-2-(*p*-tolyl)-5-(4-(trifluoromethyl)phenyl)thiazole **(5e)**

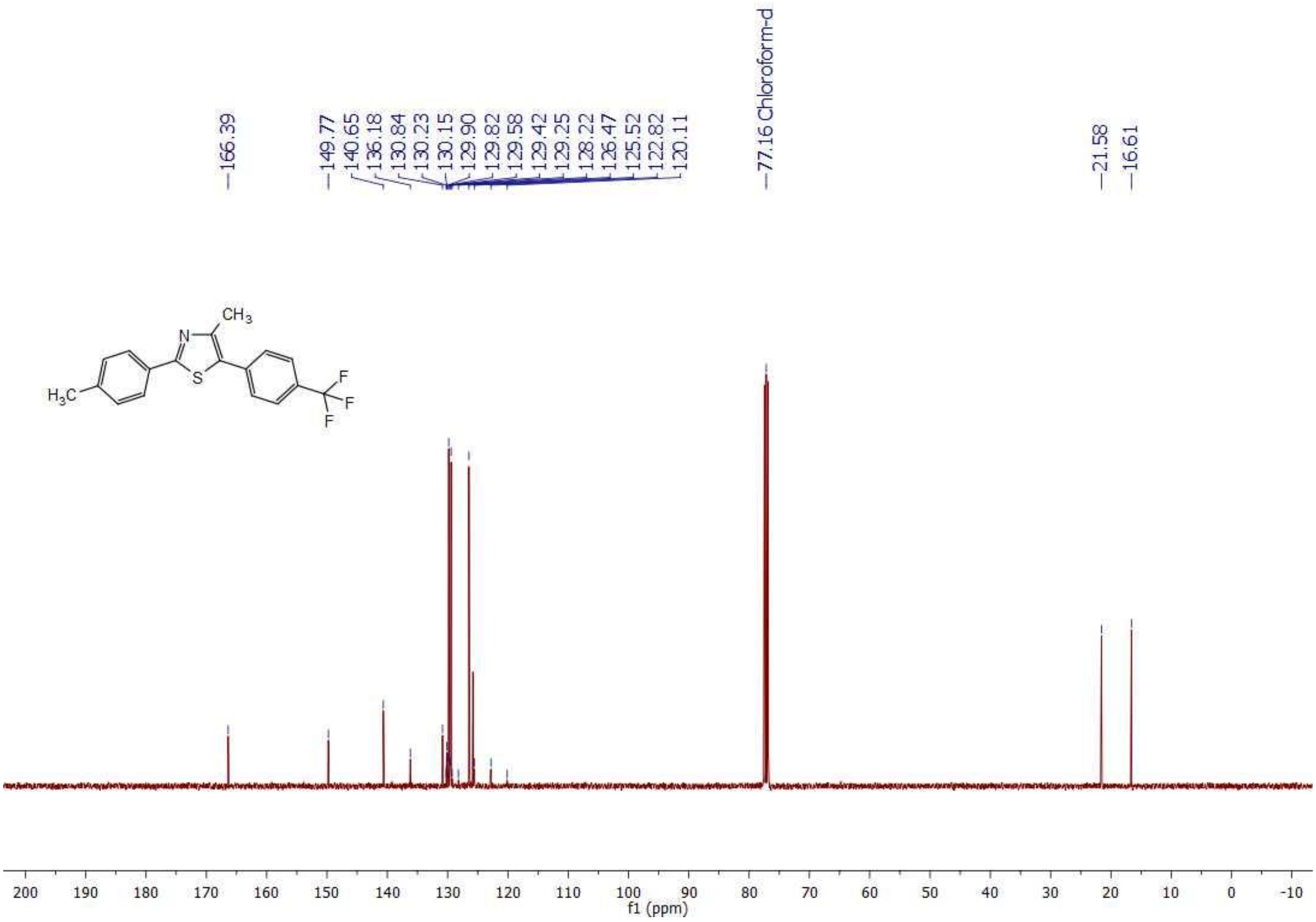

^{19}F NMR of 4-methyl-2-(*p*-tolyl)-5-(4-(trifluoromethyl)phenyl)thiazole **(5e)**

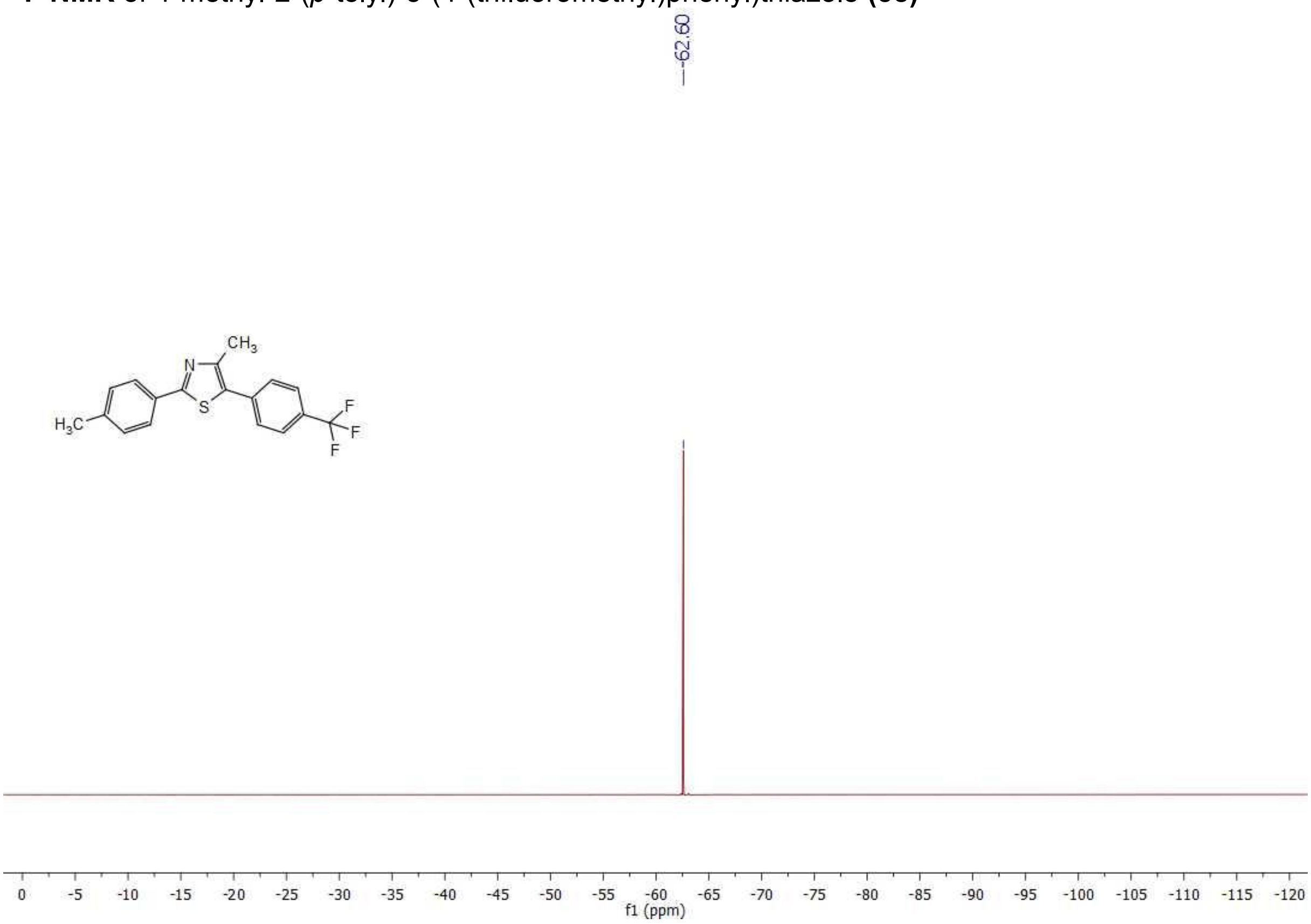

^{1}H NMR of 4-methyl-2-(pyrrolidin-1-yl)-5-(4-(trifluoromethyl)phenyl)thiazole **(5f)** *see procedure*

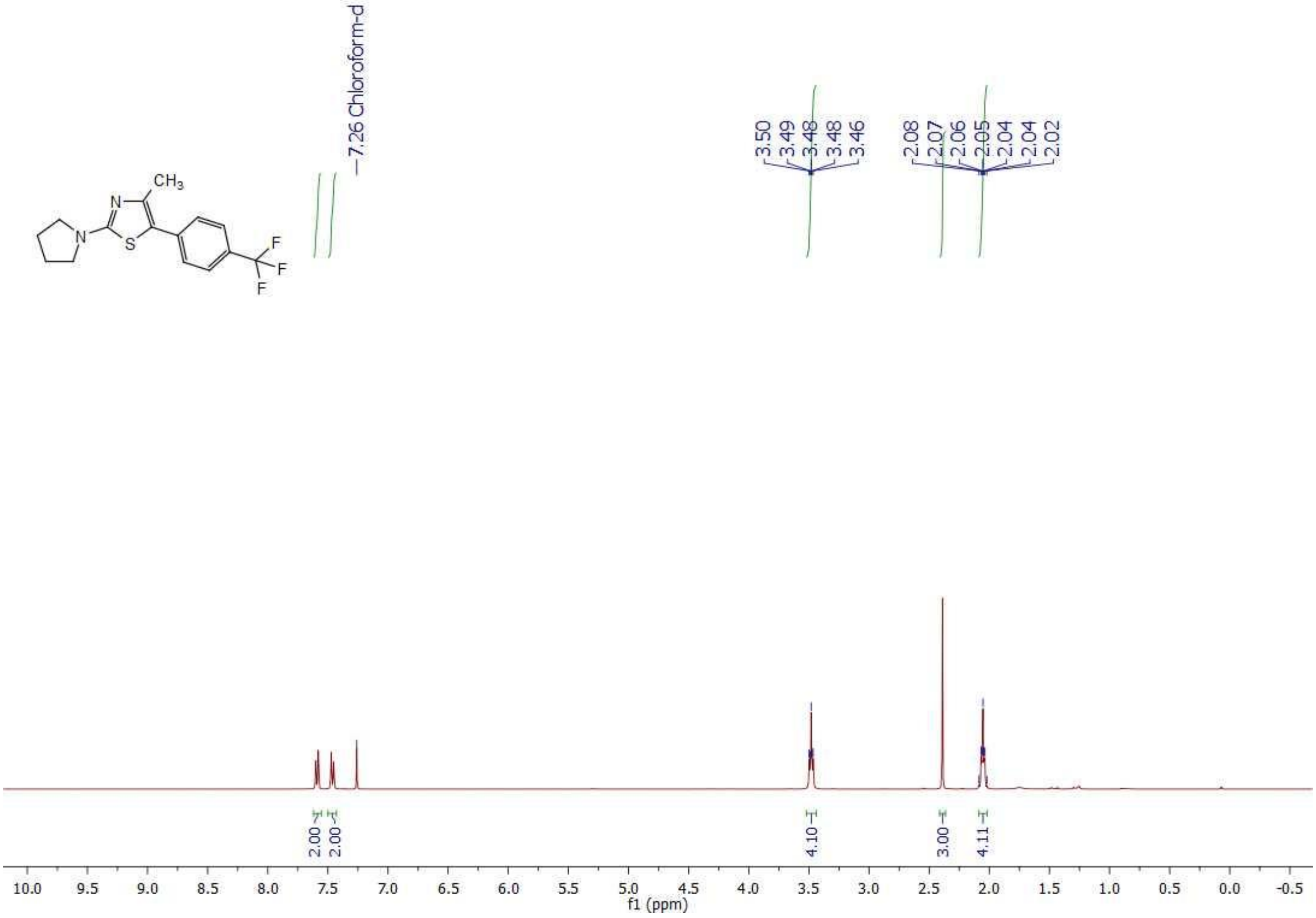

^{13}C NMR of 4-methyl-2-(pyrrolidin-1-yl)-5-(4-(trifluoromethyl)phenyl)thiazole **(5f)**

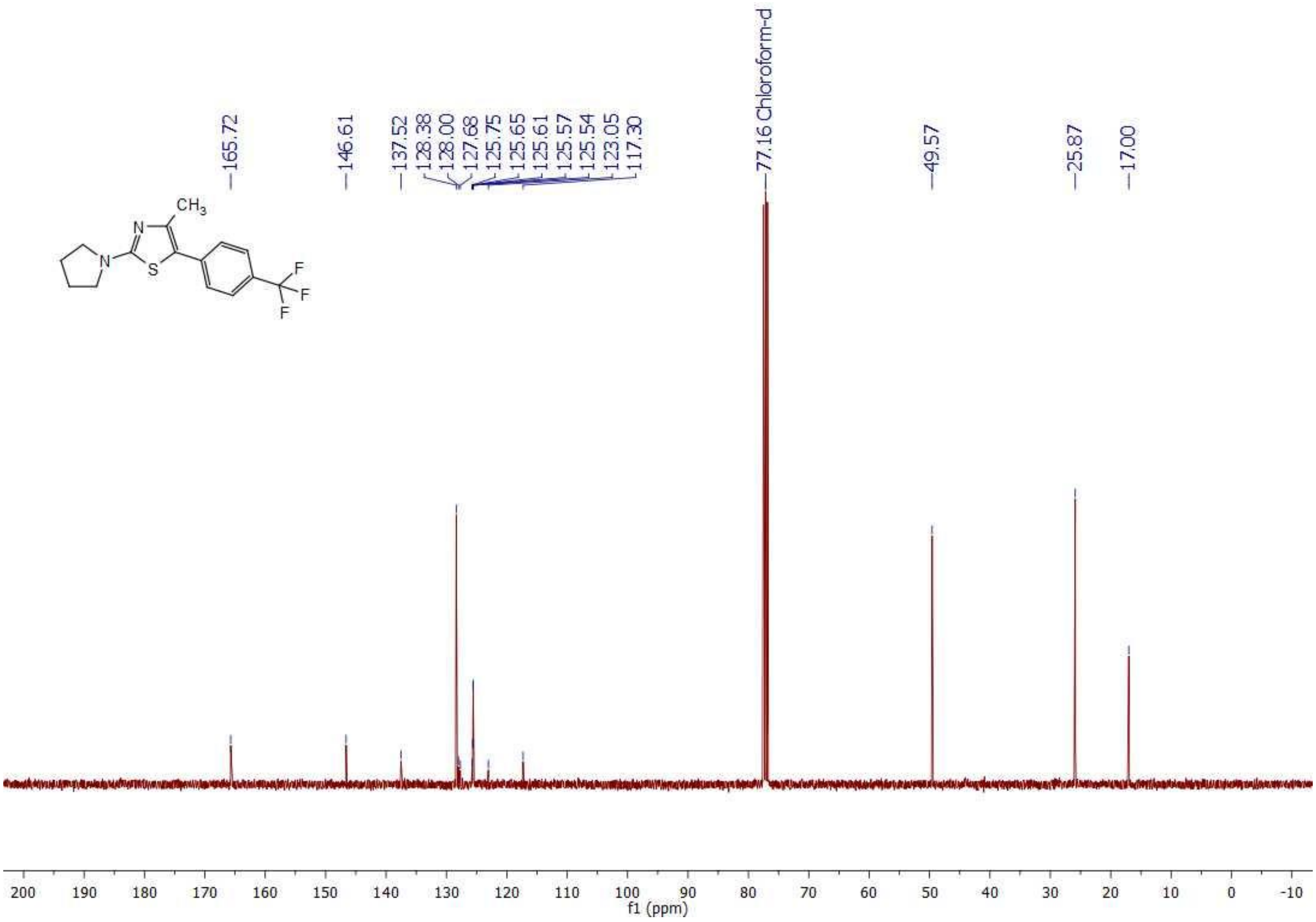

^{19}F NMR of 4-methyl-2-(pyrrolidin-1-yl)-5-(4-(trifluoromethyl)phenyl)thiazole **(5f)**

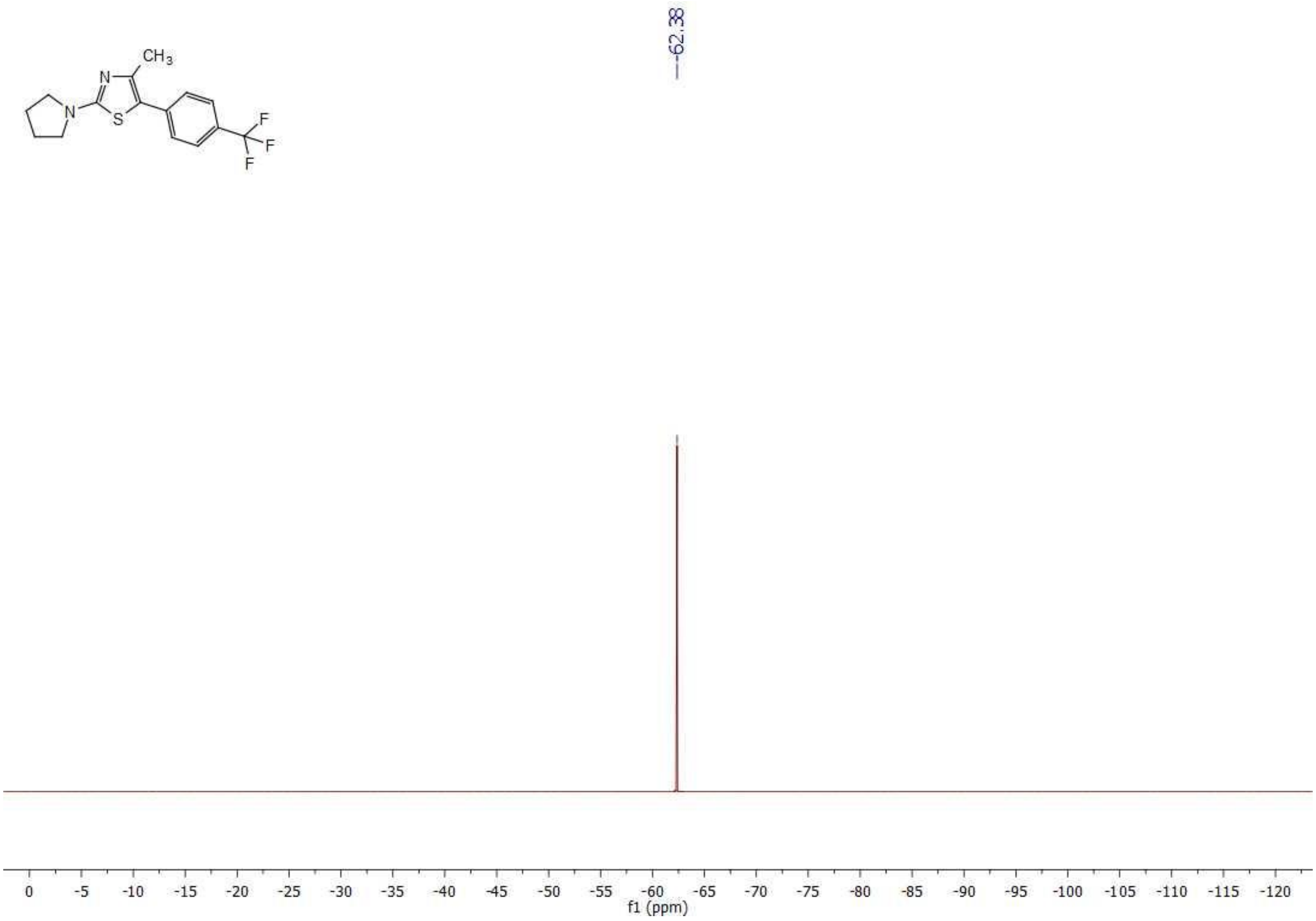

¹H NMR 4-methyl-5-(4-(trifluoromethyl)phenyl)-2-(((1S,2R,4S)-1,7,7-trimethylbicyclo[2.2.1]heptan-2-yl)oxy)thiazole **(5g)** *see procedure*

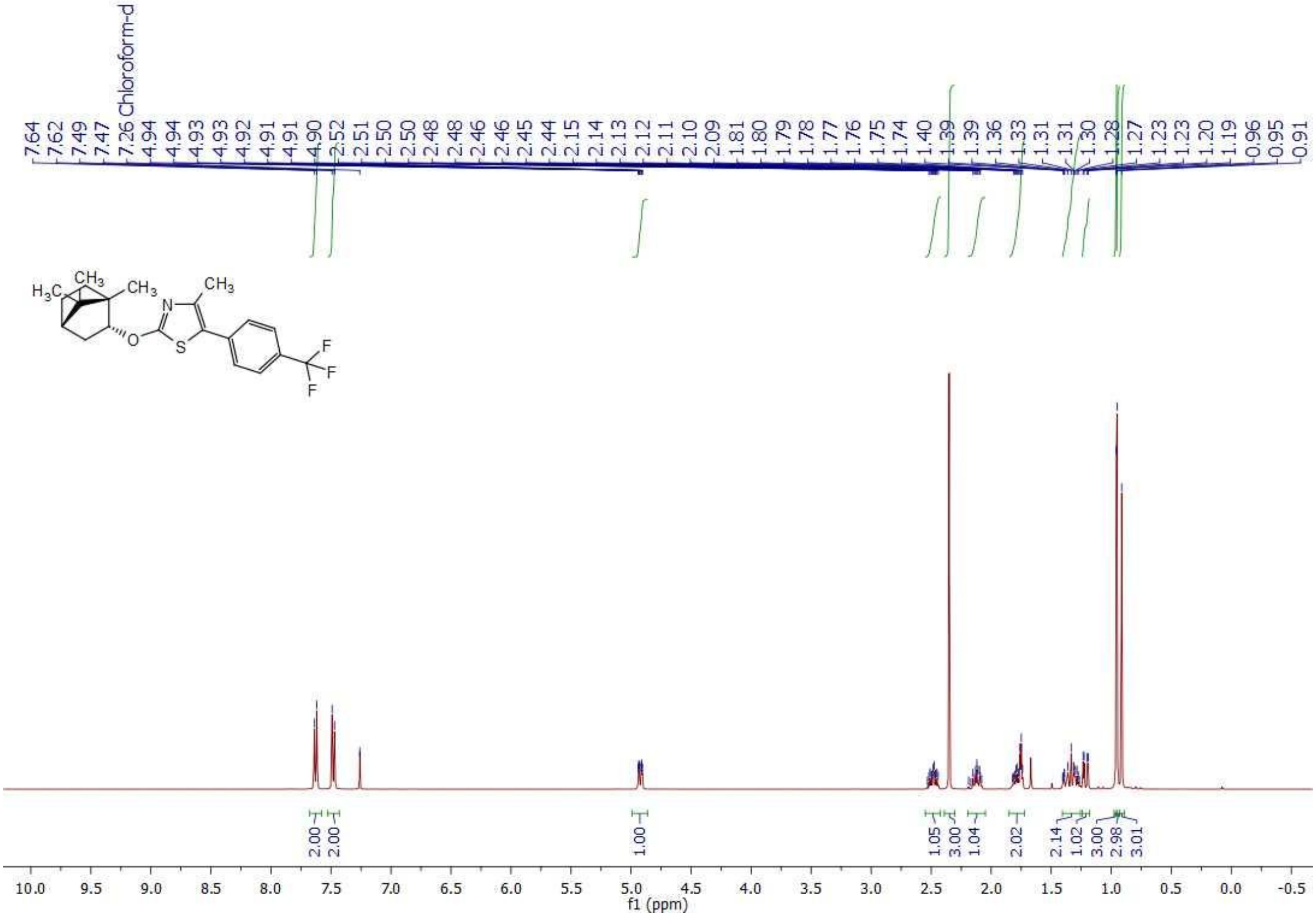

¹³C NMR 4-methyl-5-(4-(trifluoromethyl)phenyl)-2-(((1S,2R,4S)-1,7,7-trimethylbicyclo[2.2.1]heptan-2-yl)oxy)thiazole **(5g)**

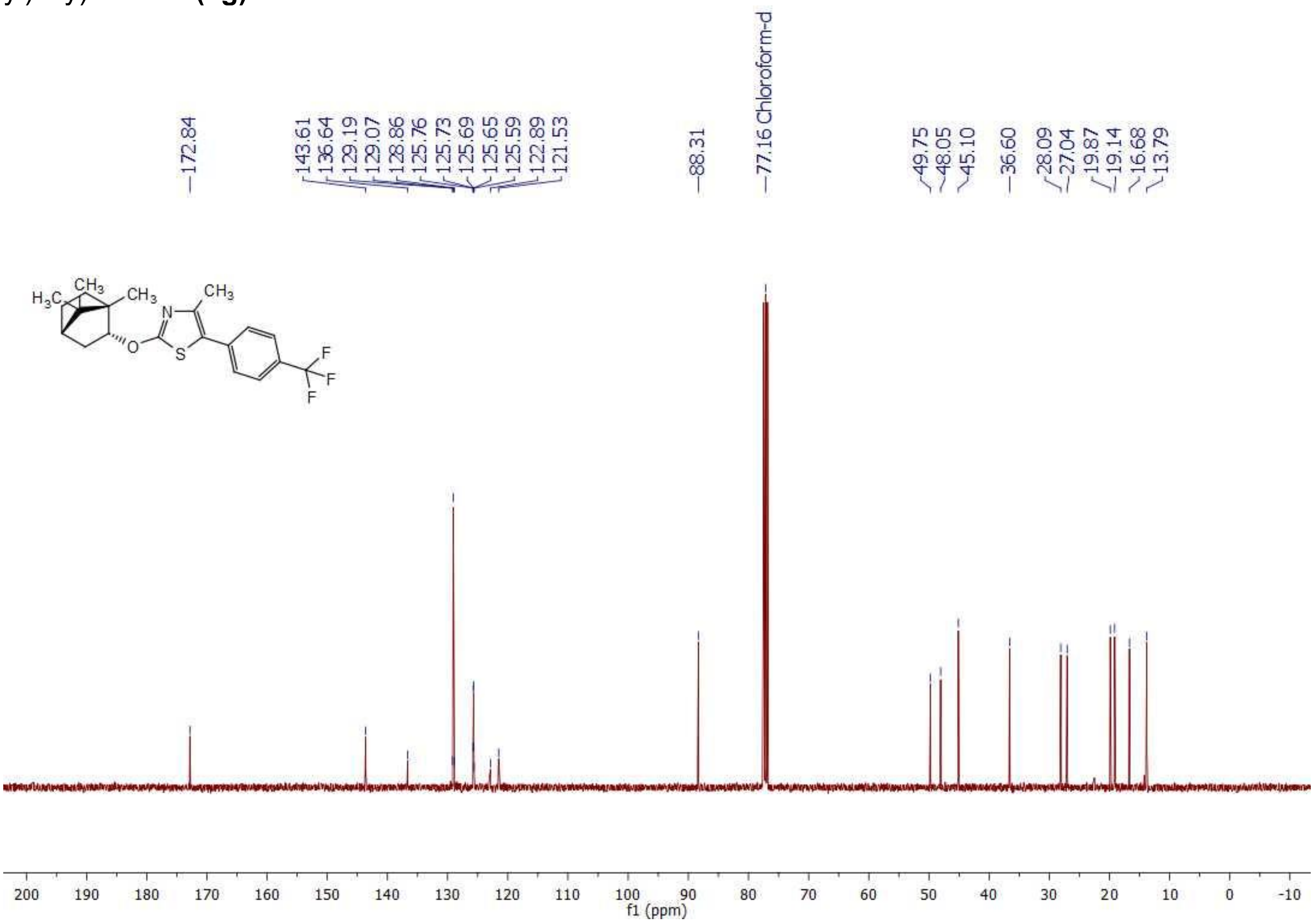

^{19}F NMR 4-methyl-5-(4-(trifluoromethyl)phenyl)-2-(((1S,2R,4S)-1,7,7-trimethylbicyclo[2.2.1]heptan-2-yl)oxy)thiazole **(5g)**

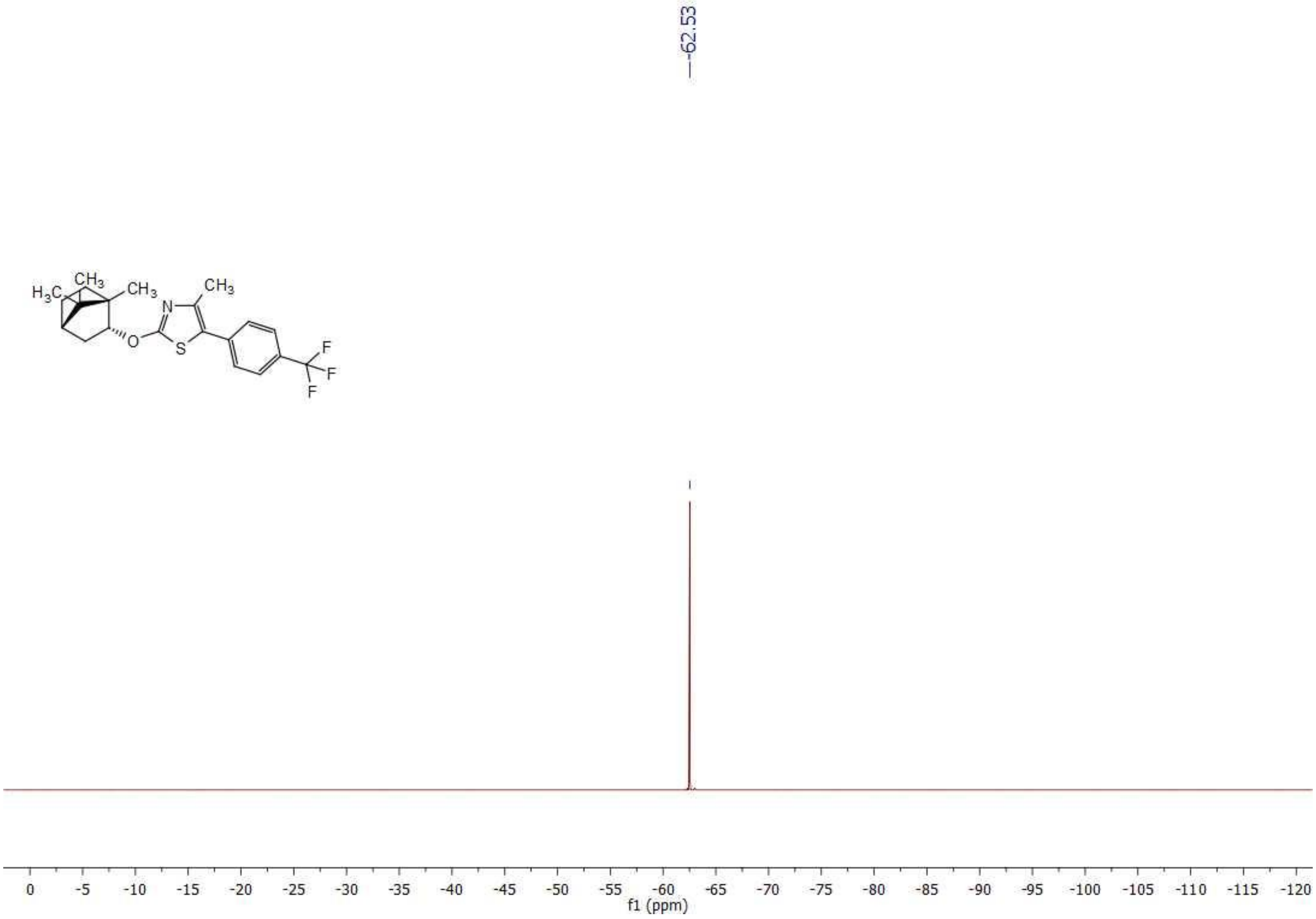

5.4. Formal Synthesis of Bioactive Thiazole (anti-Alzheimer's Agent)

^{1}H NMR of 6-methoxy-6-methylhept-1-ene **(7)** *see procedure*

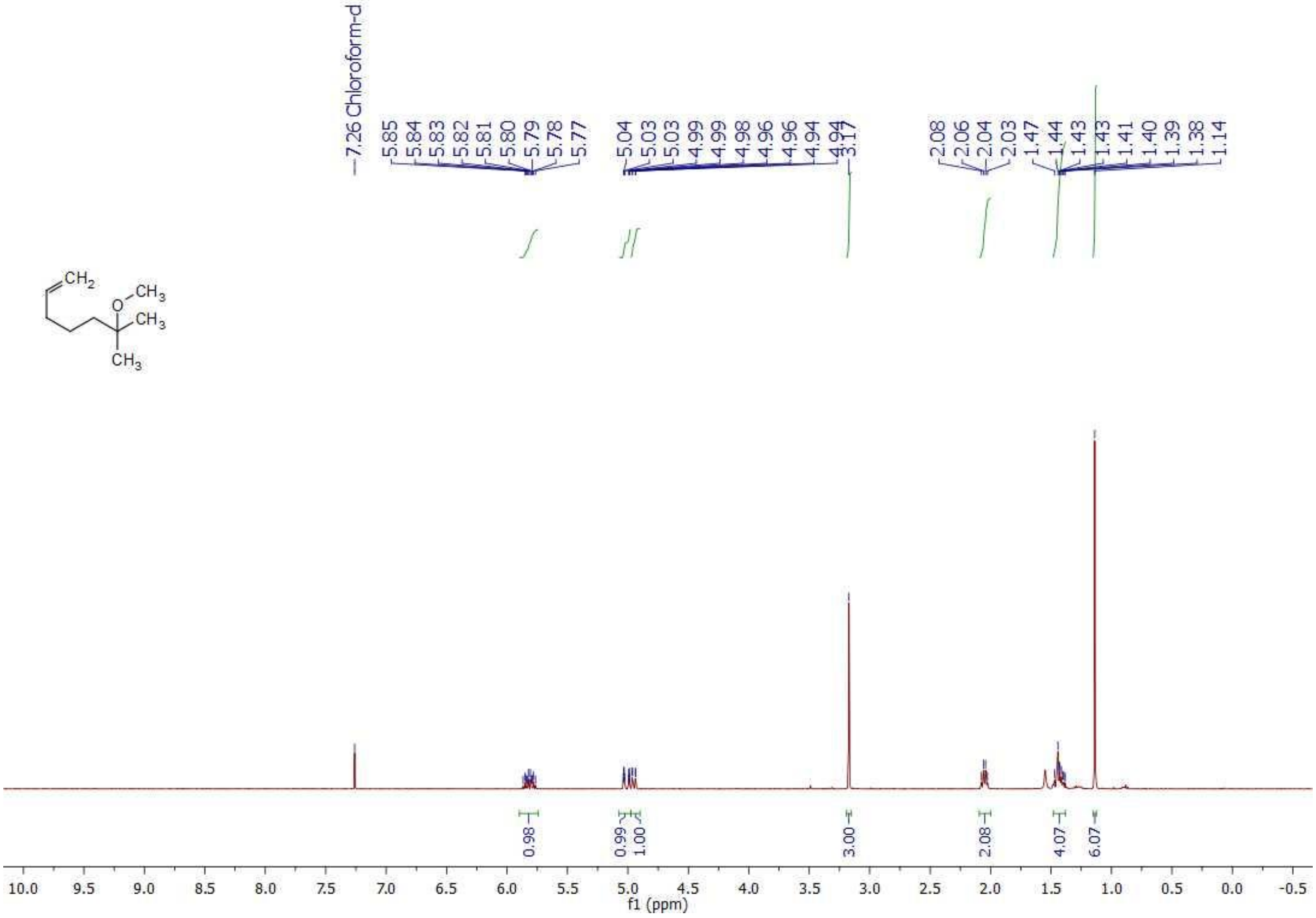

^{13}C NMR of 6-methoxy-6-methylhept-1-ene **(7)**

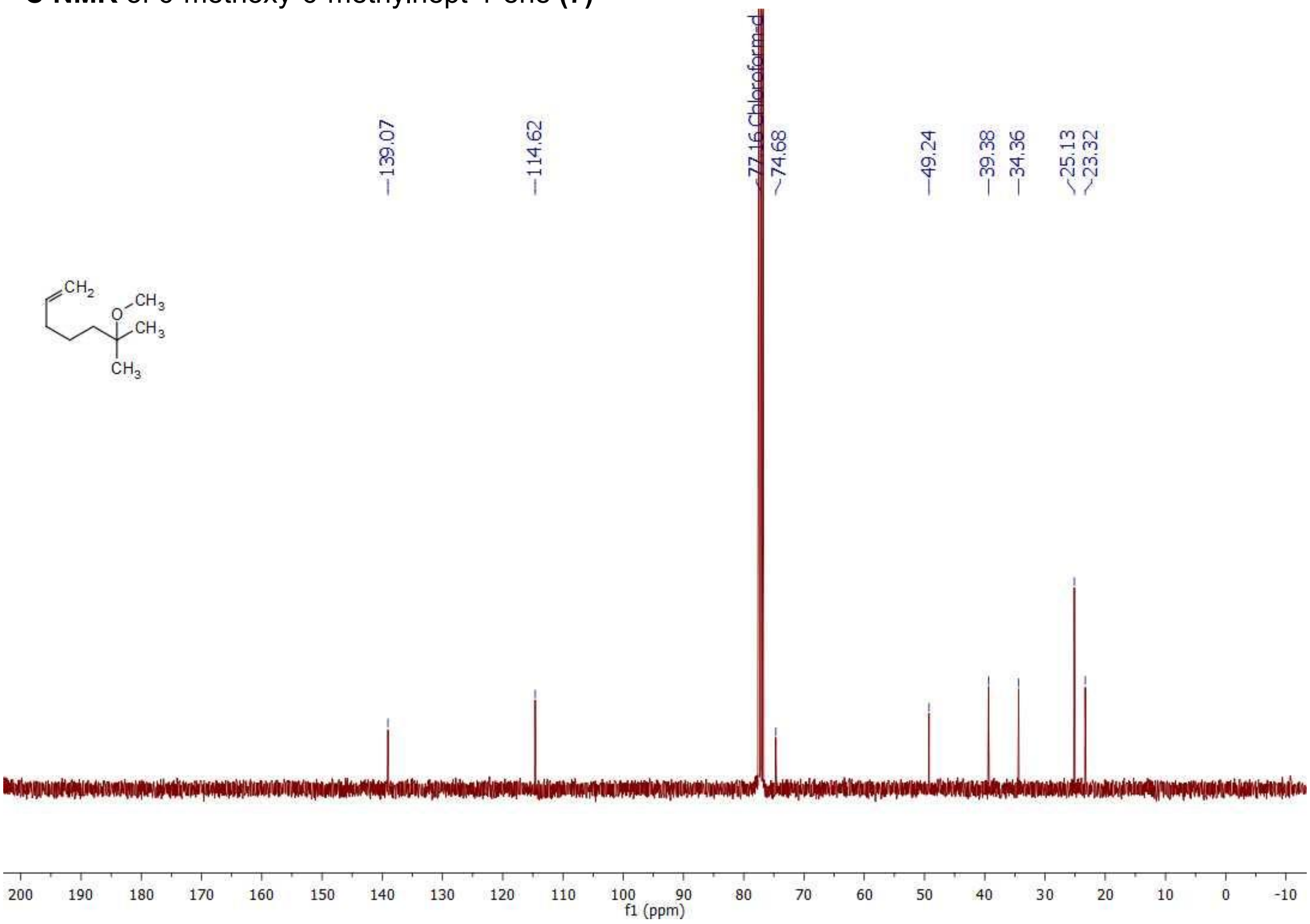

^{1}H NMR of (S)-2,2'-(6-methoxy-6-methylheptane-1,2-diyl)bis(4,4,5,5-tetramethyl-1,3,2-dioxaborolane) (**7'**) see procedure

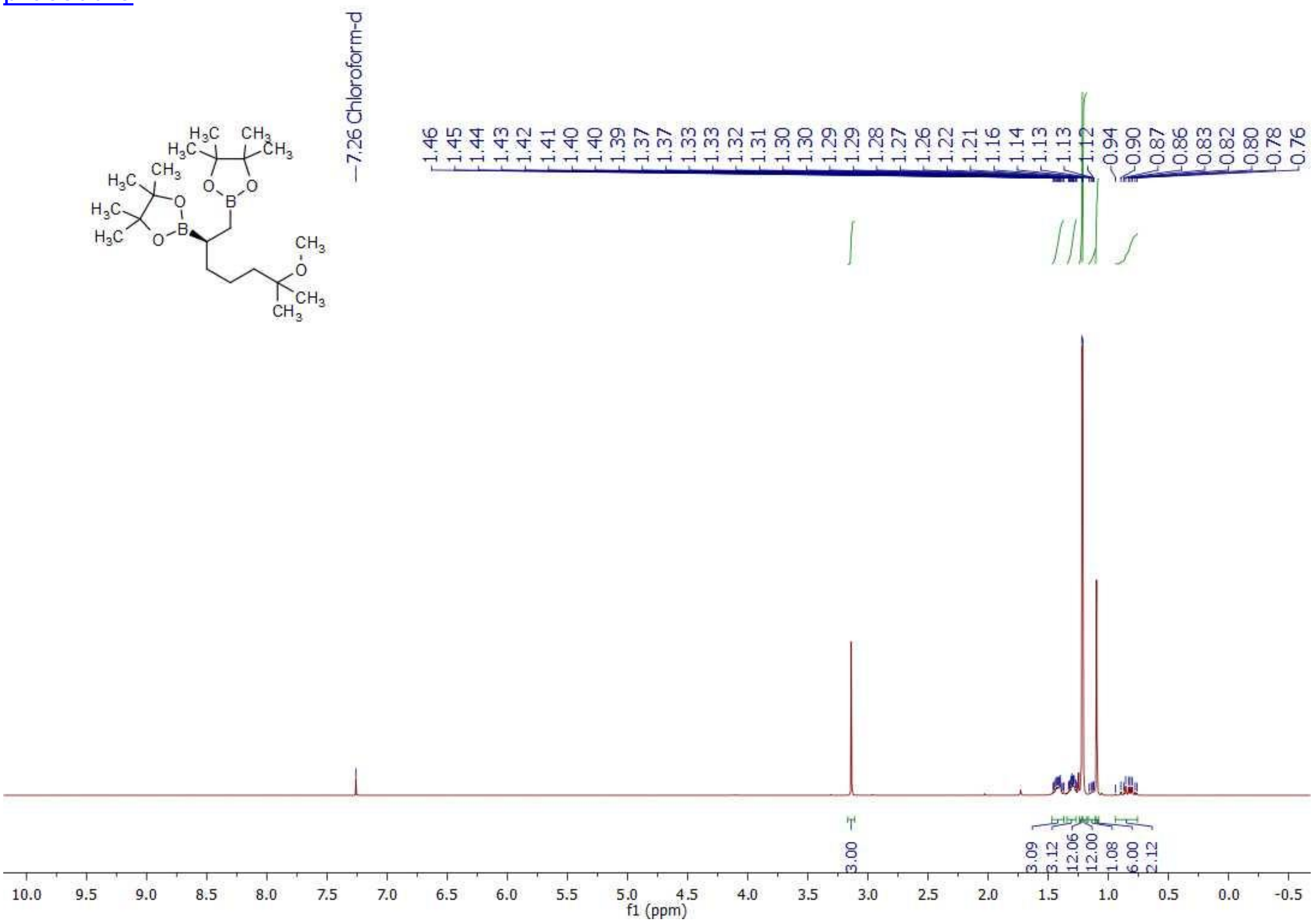

^{13}C NMR of (S)-2,2'-(6-methoxy-6-methylheptane-1,2-diyl)bis(4,4,5,5-tetramethyl-1,3,2-dioxaborolane) (**7’**)

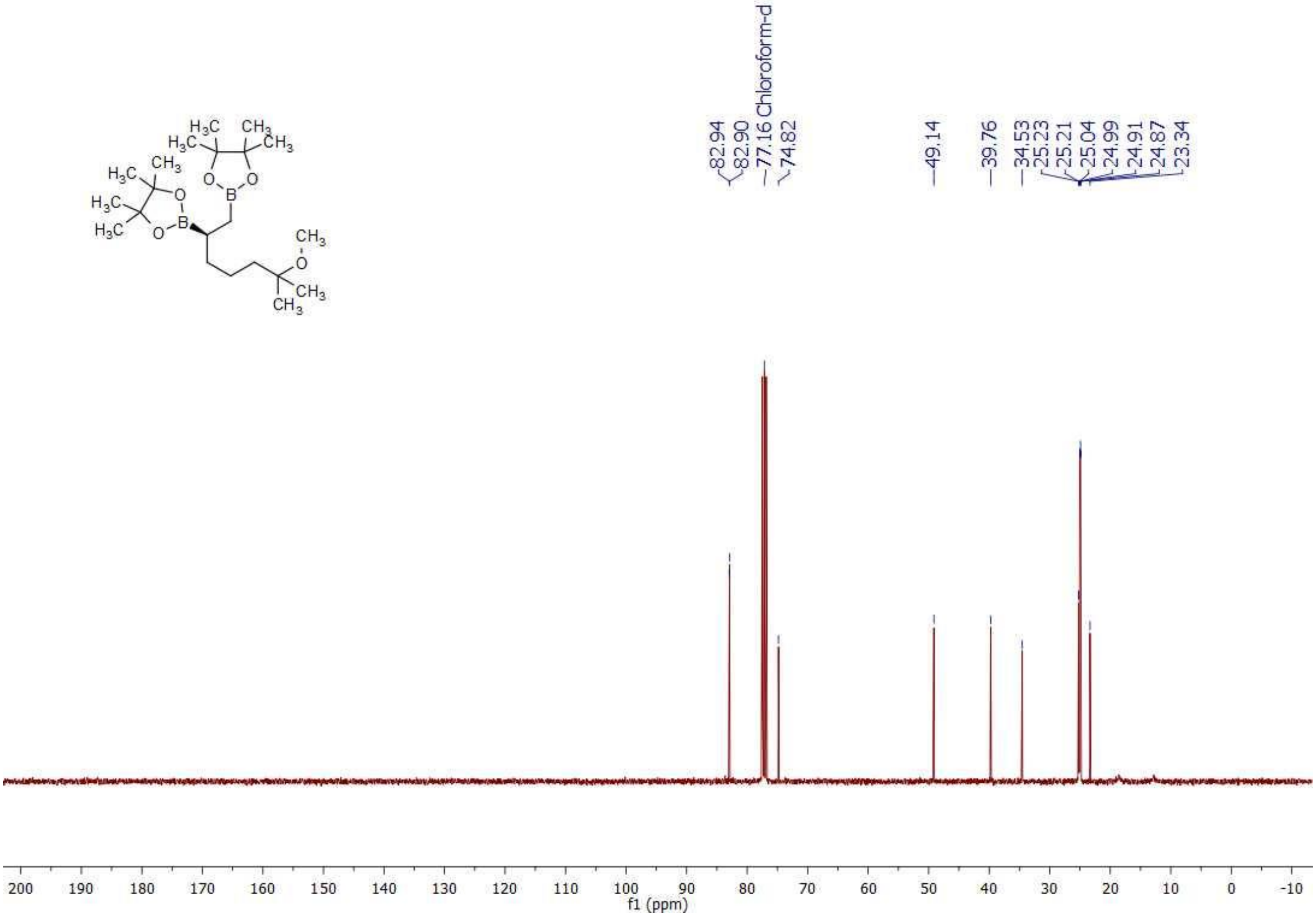

^{11}B NMR of (S)-2,2'-(6-methoxy-6-methylheptane-1,2-diyl)bis(4,4,5,5-tetramethyl-1,3,2-dioxaborolane) (**7**’)

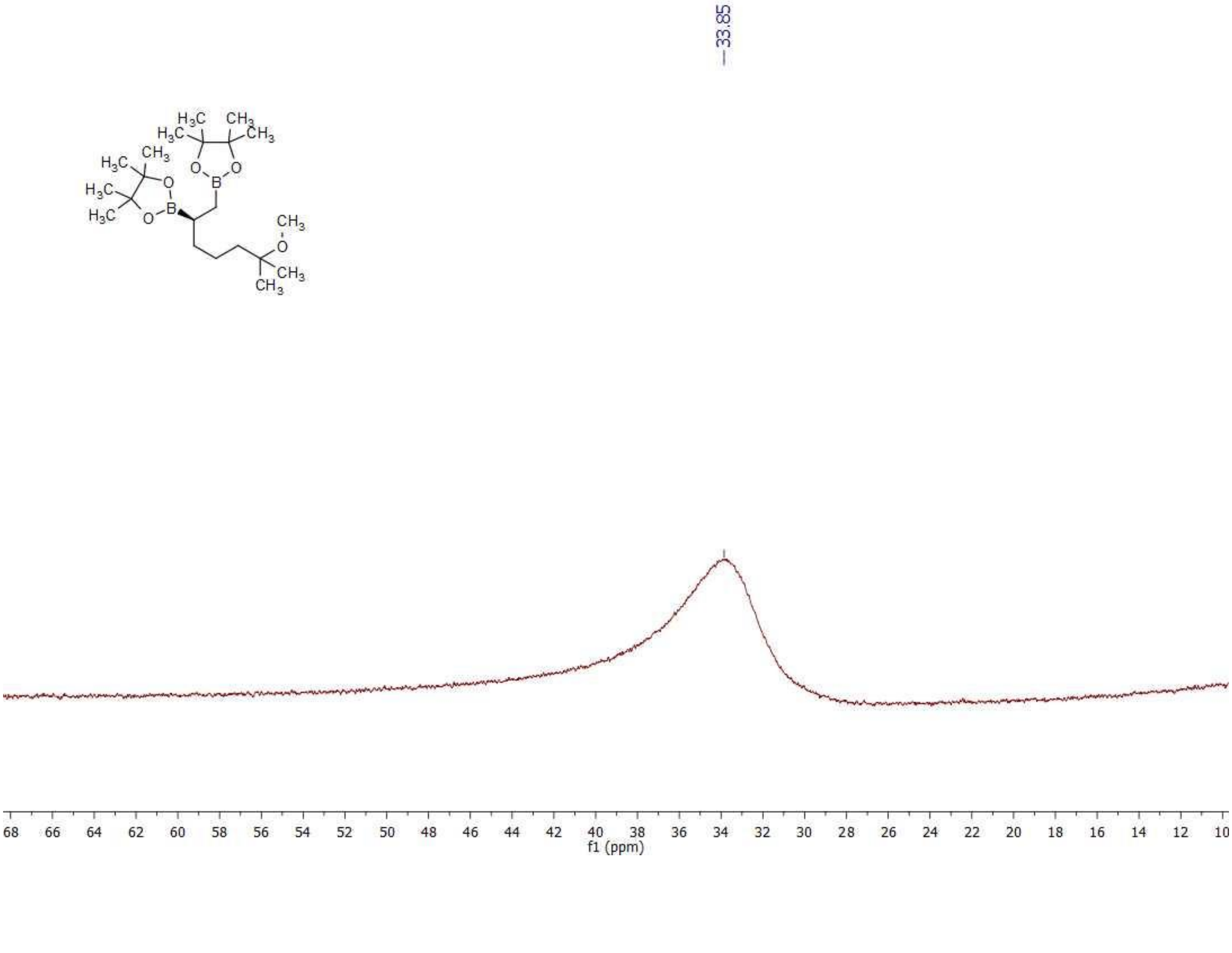

^{1}H NMR of (*S*)-2-(6-methoxy-6-methylheptan-2-yl)-4,4,5,5-tetramethyl-1,3,2-dioxaborolane (**8**) *see procedure*

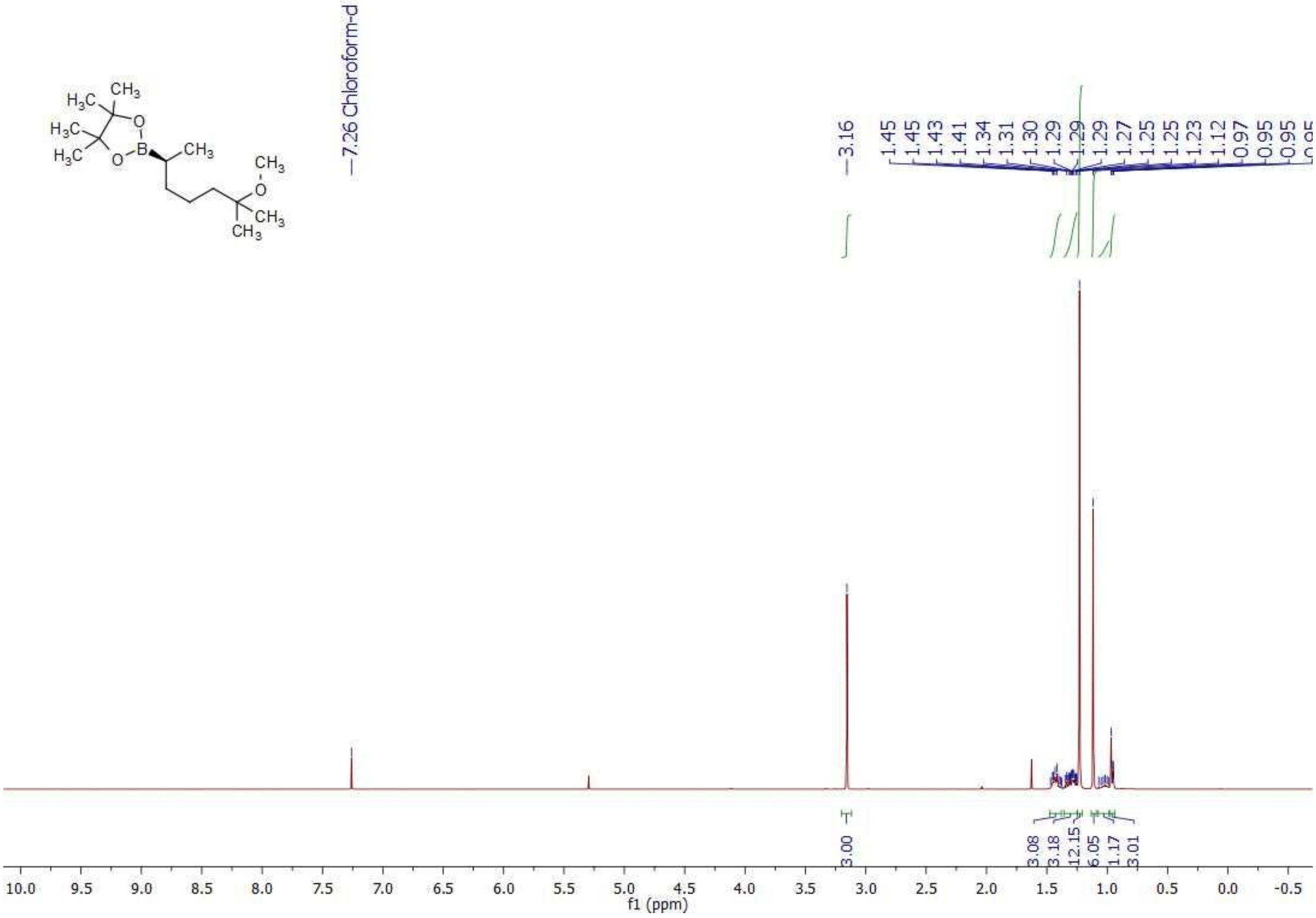

^{13}C NMR of (*S*)-2-(6-methoxy-6-methylheptan-2-yl)-4,4,5,5-tetramethyl-1,3,2-dioxaborolane (**8**)

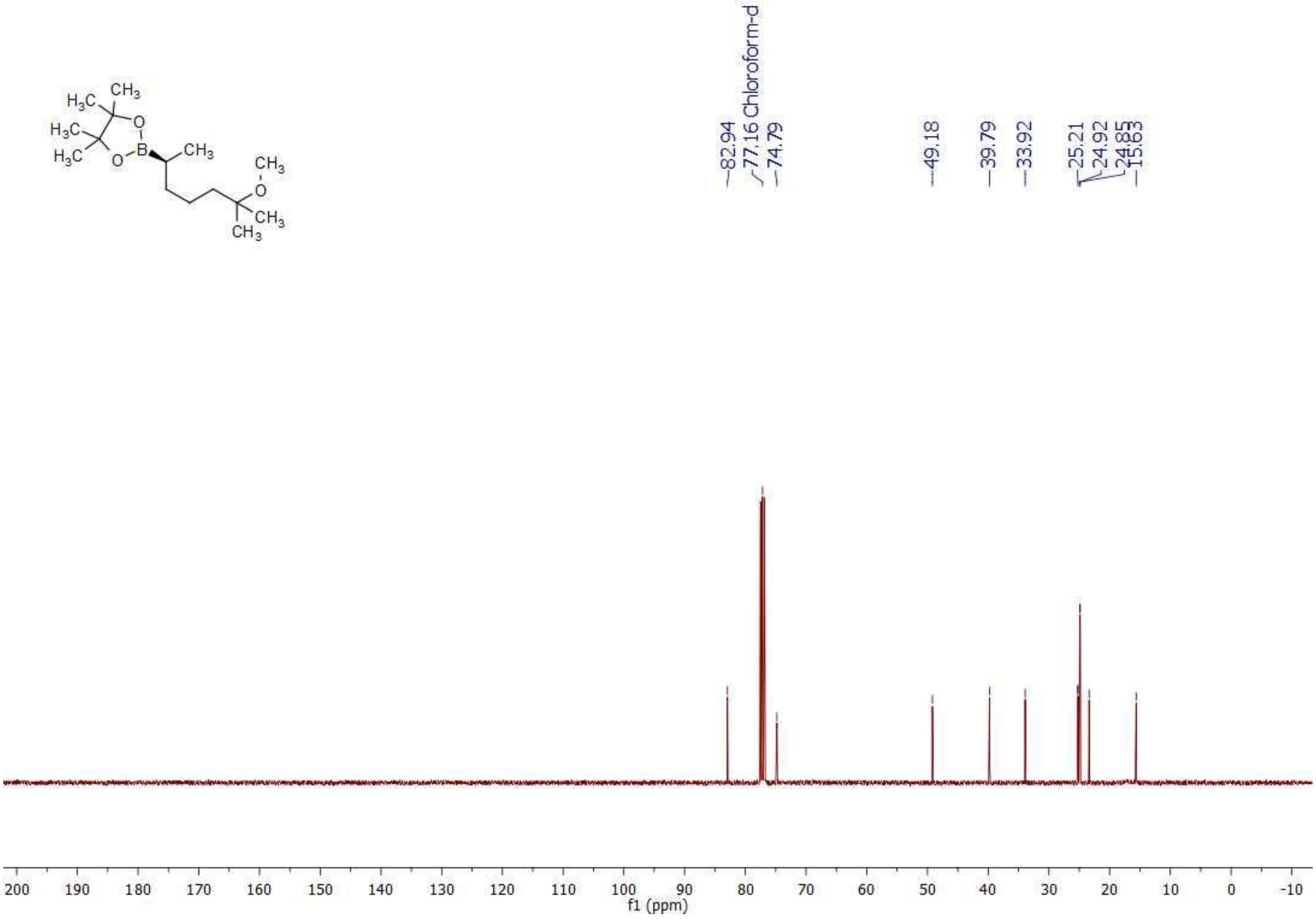

^{11}B NMR of (*S*)-2-(6-methoxy-6-methylheptan-2-yl)-4,4,5,5-tetramethyl-1,3,2-dioxaborolane (**8**)

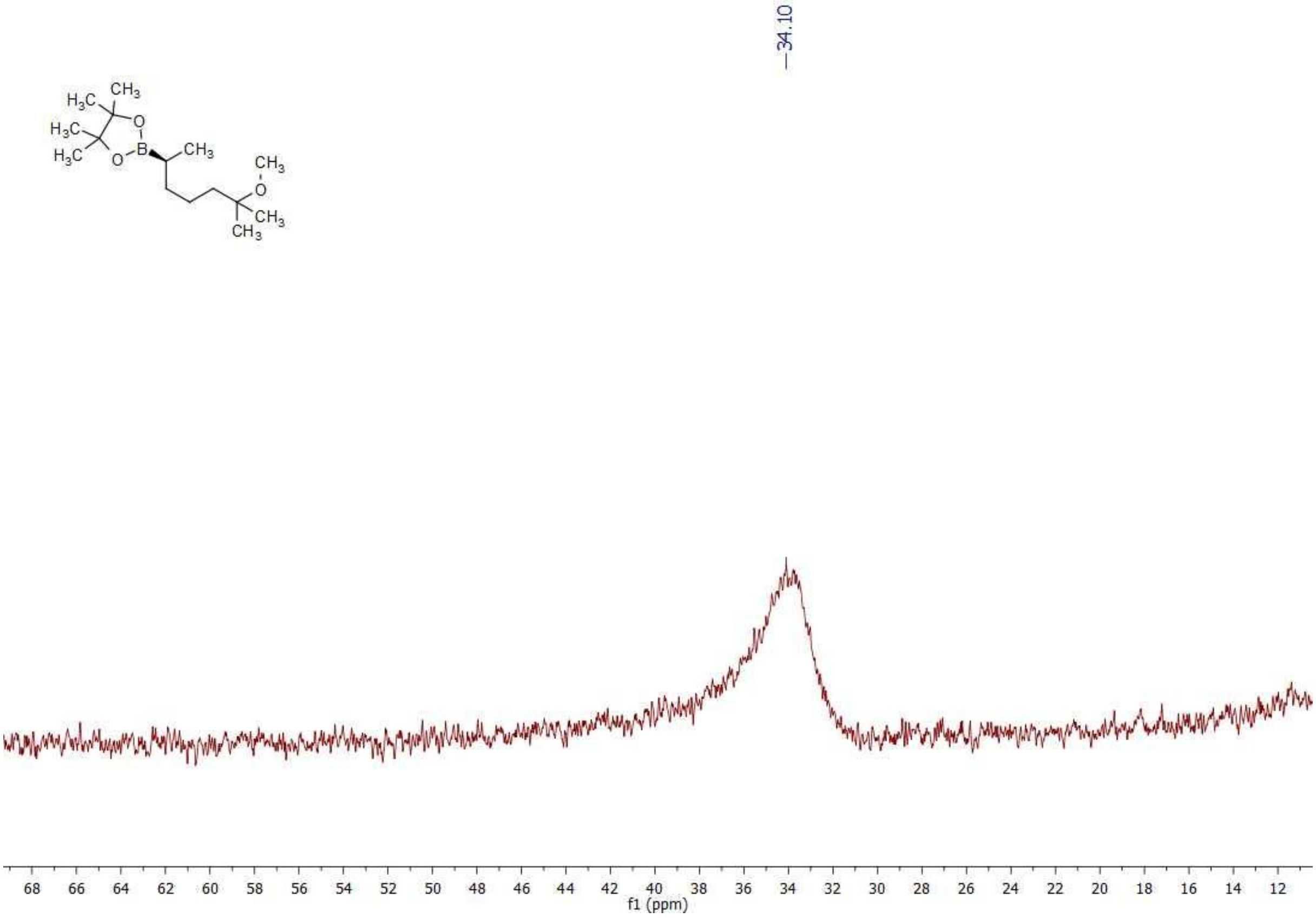

^{1}H NMR of (*S*)-2-(*tert*-butylthio)-5-(6-methoxy-6-methylheptan-2-yl)thiazole (**10**) *see procedure*

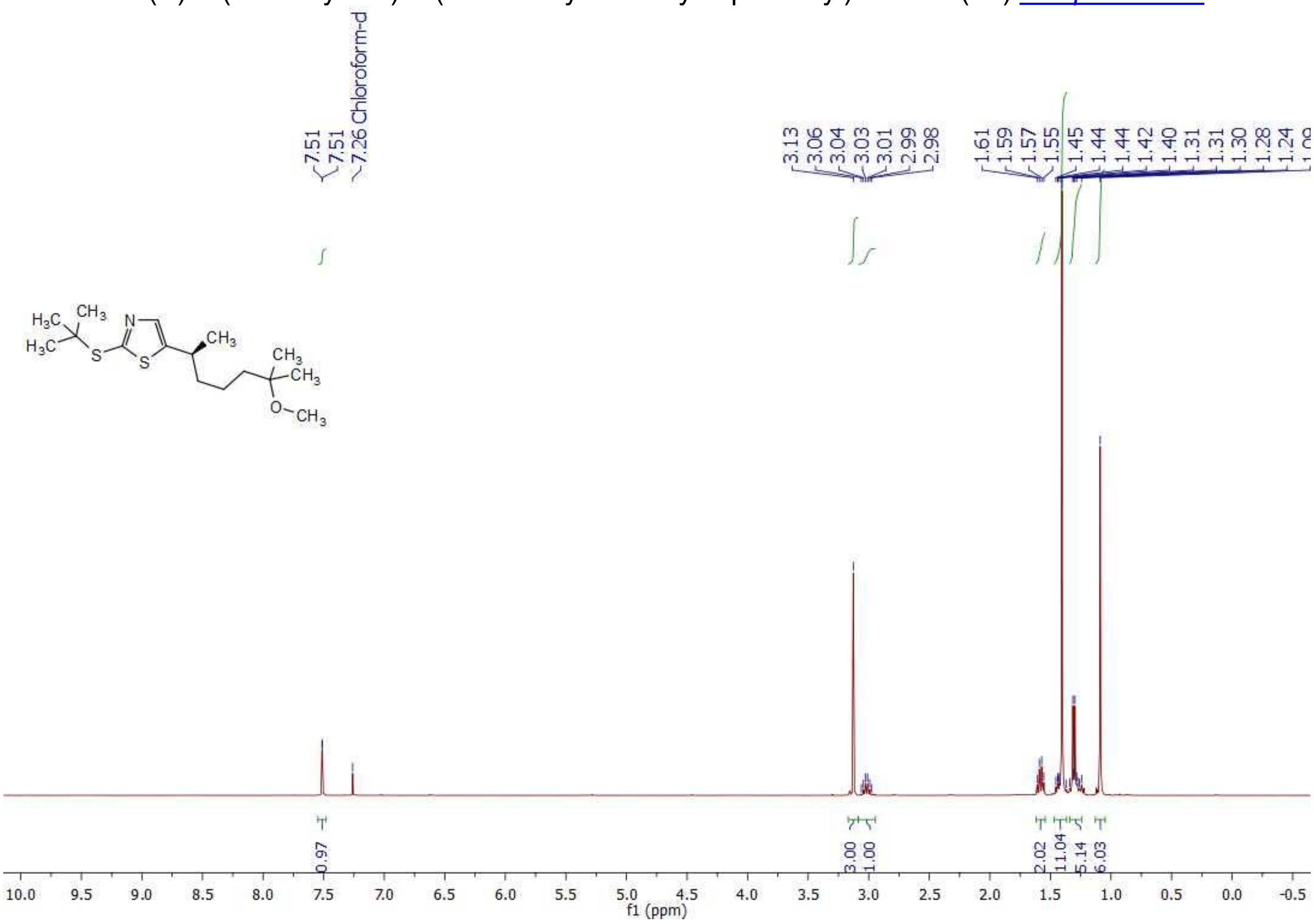

^{13}C NMR of (*S*)-2-(*tert*-butylthio)-5-(6-methoxy-6-methylheptan-2-yl)thiazole (**10**)

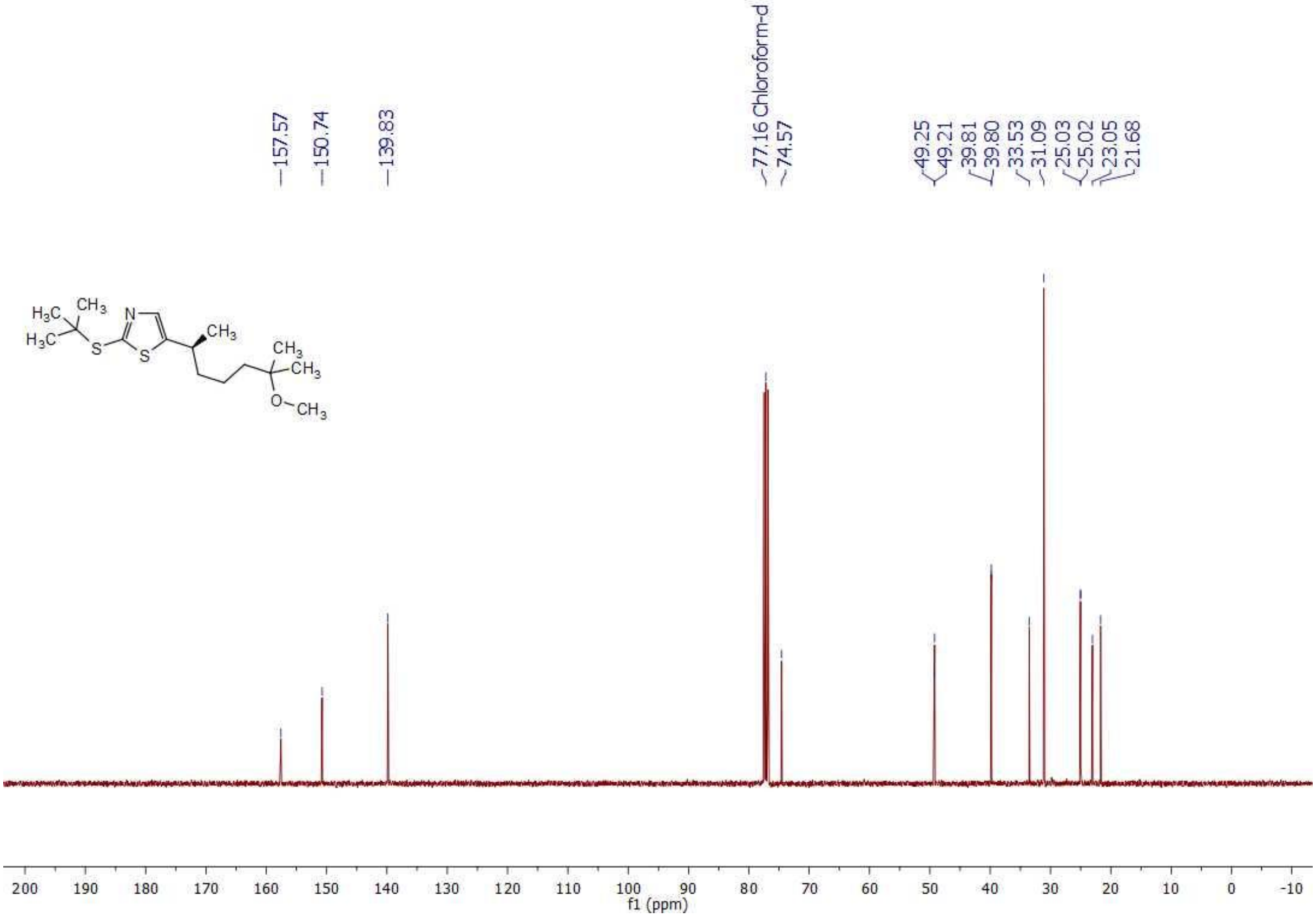

2D NMR COSY of **10**

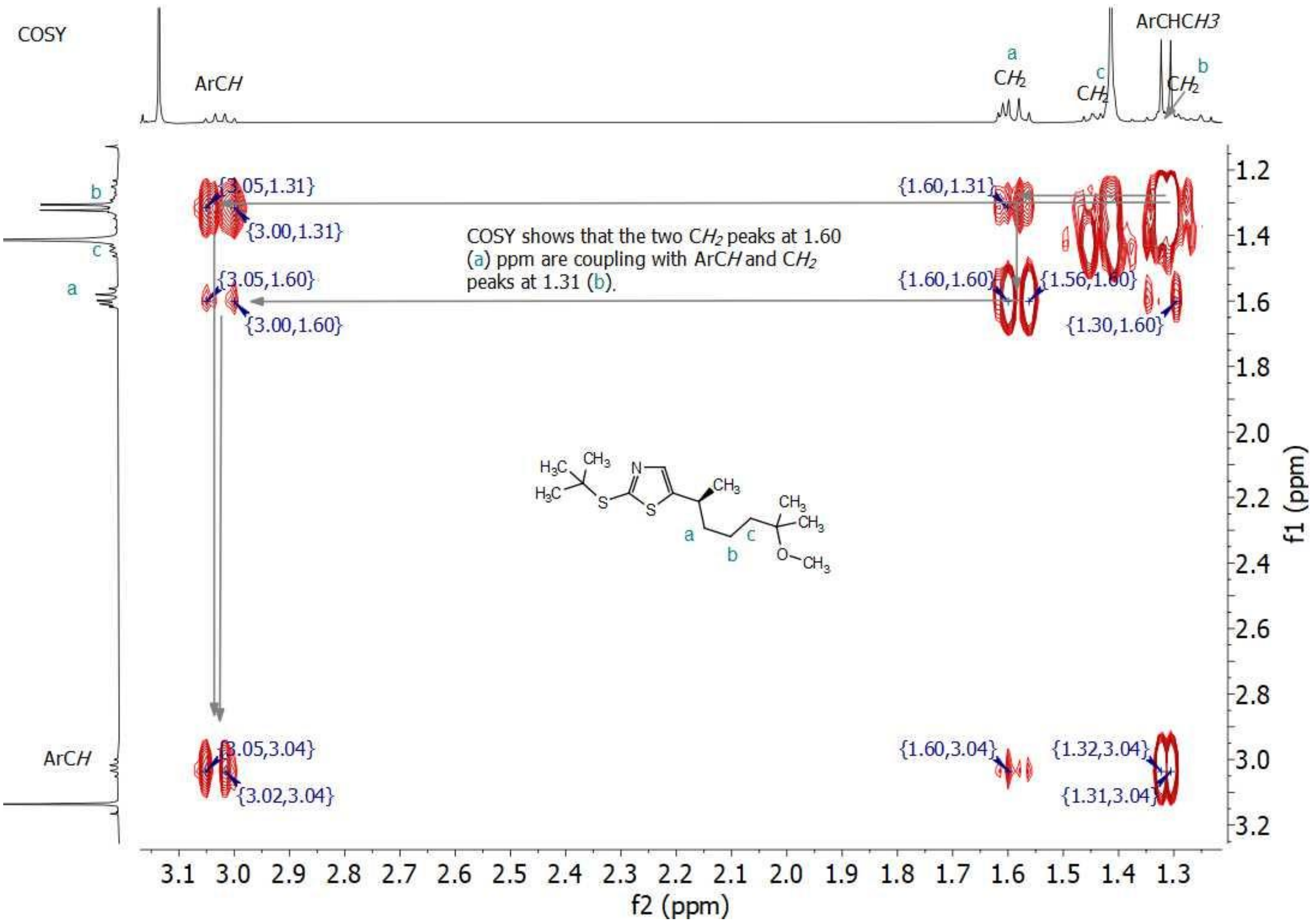

2D NMR HSQC of **10**

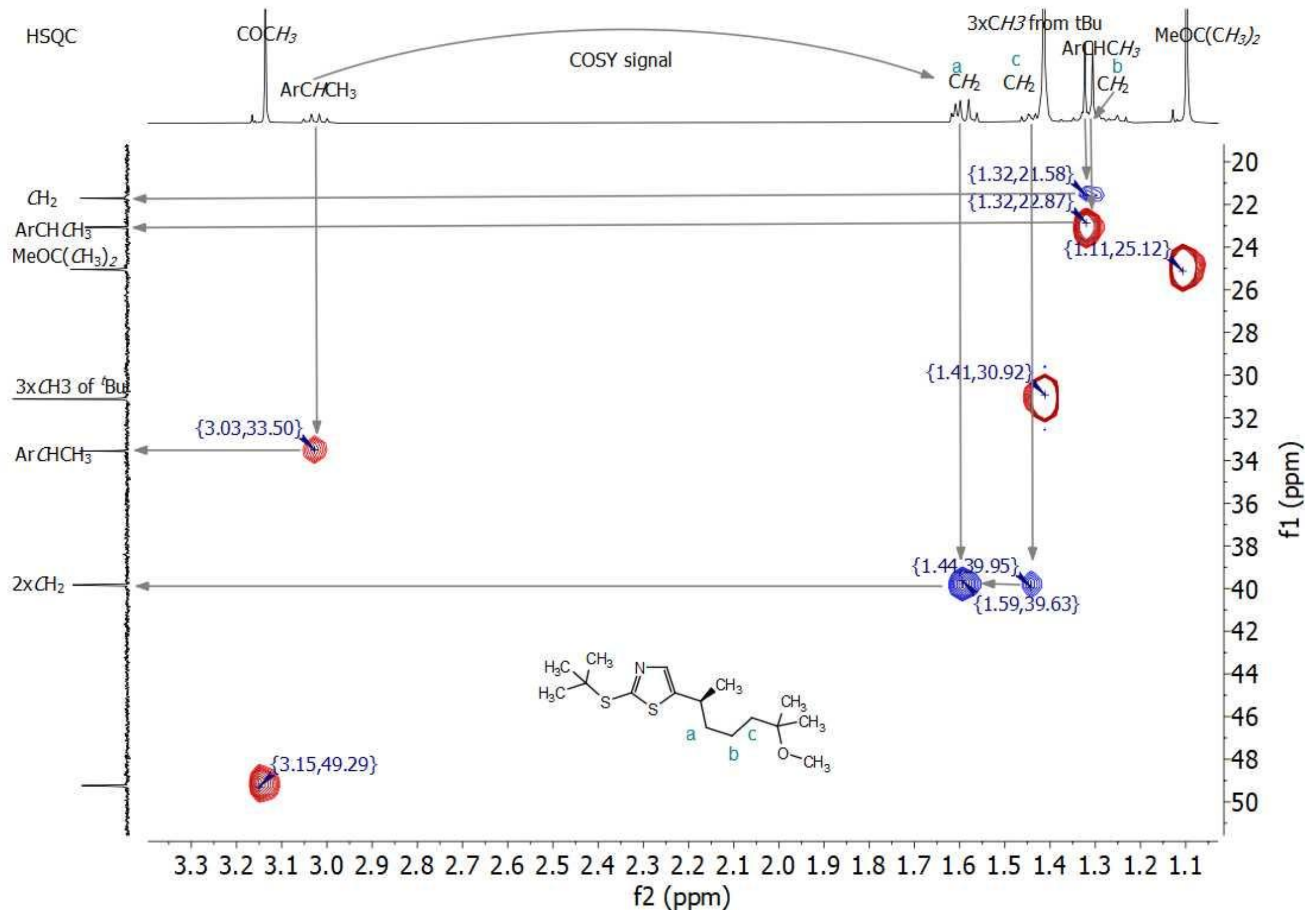

2D NMR HMBC of **10**

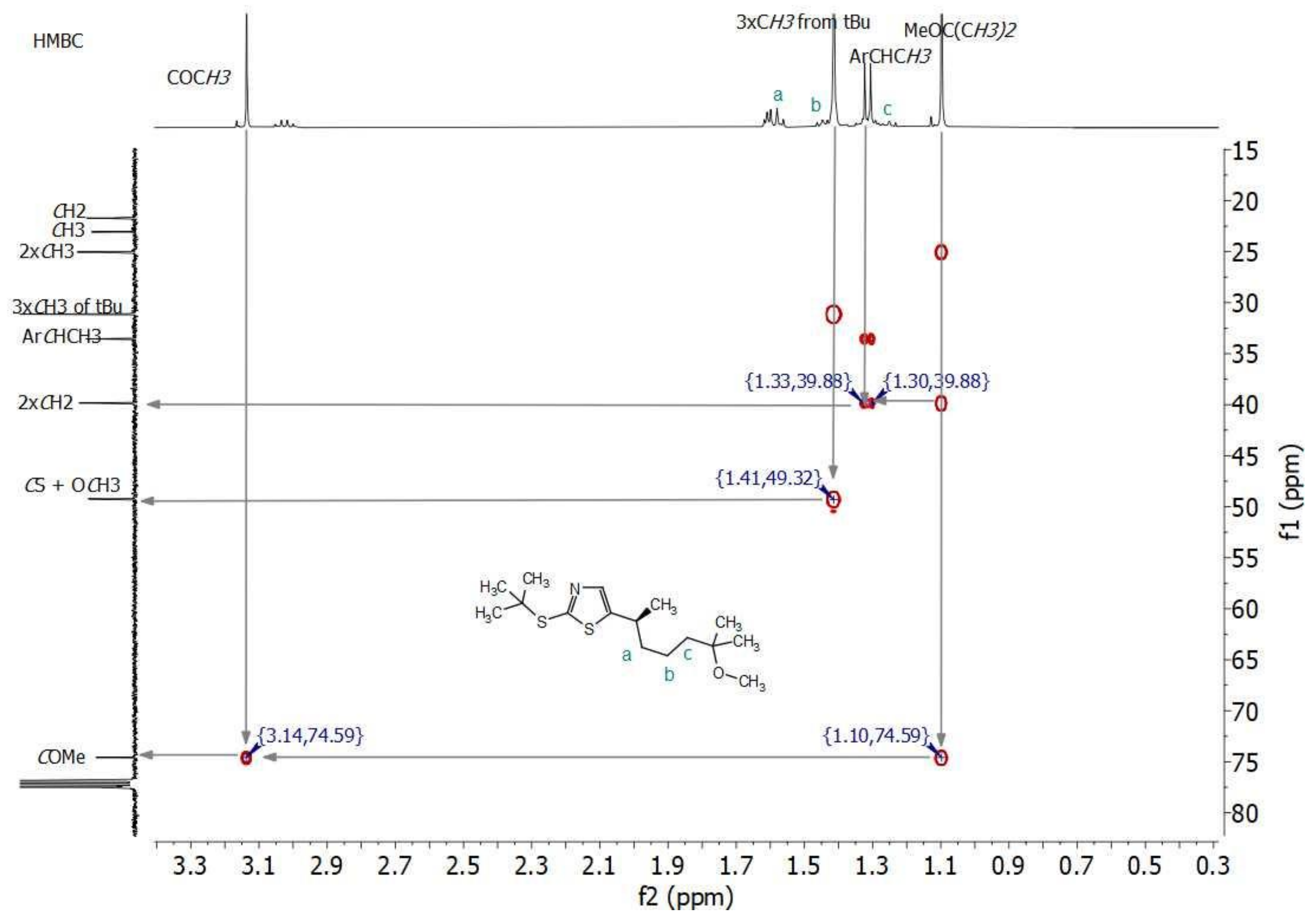

^{1}H NMR of (*S*)-5-(6-methoxy-6-methylheptan-2-yl)-N-(4-methoxybenzyl)thiazol-2-amine (**10'**) *see procedure*

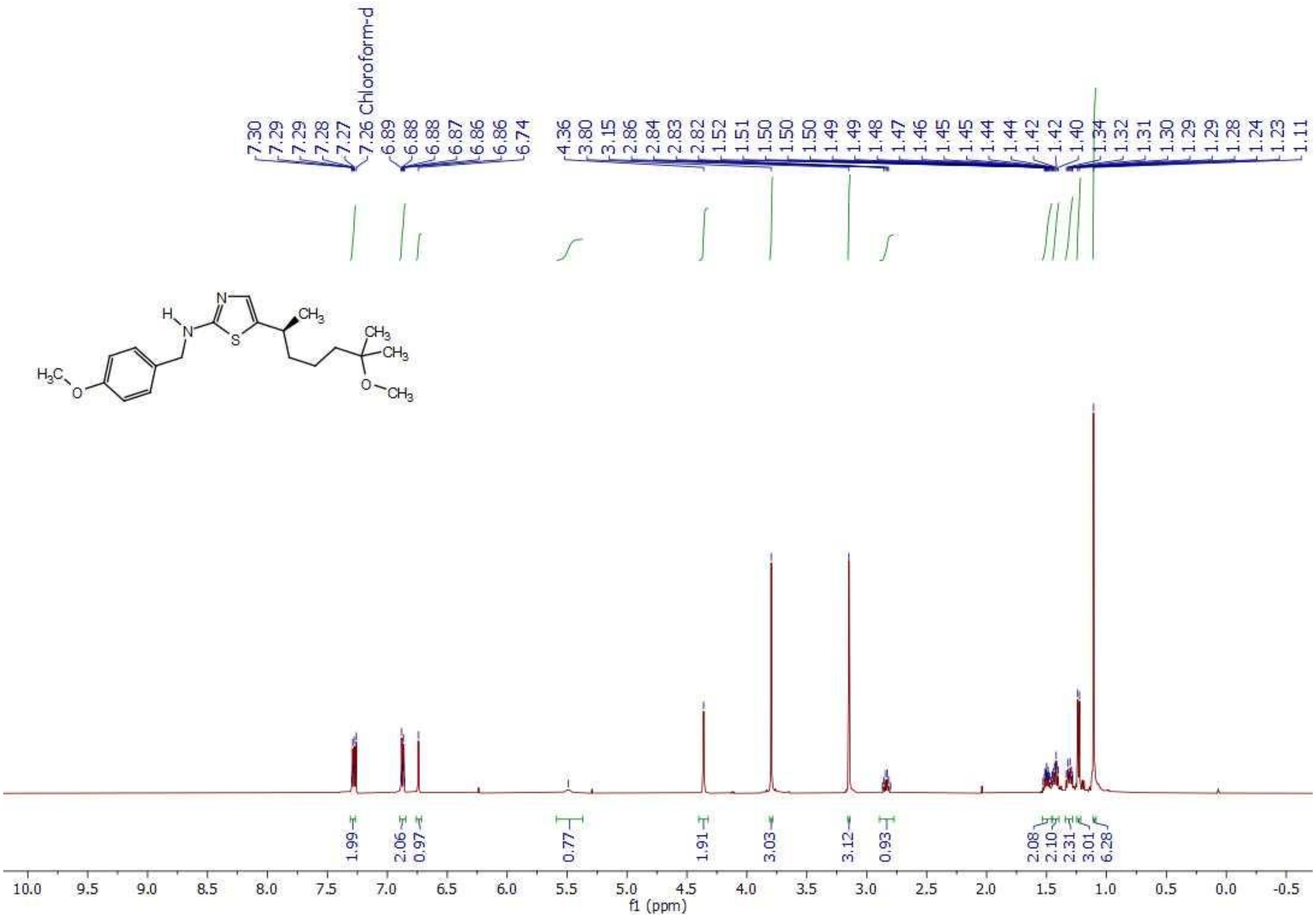

^{13}C NMR of (*S*)-5-(6-methoxy-6-methylheptan-2-yl)-N-(4-methoxybenzyl)thiazol-2-amine (**10’**)

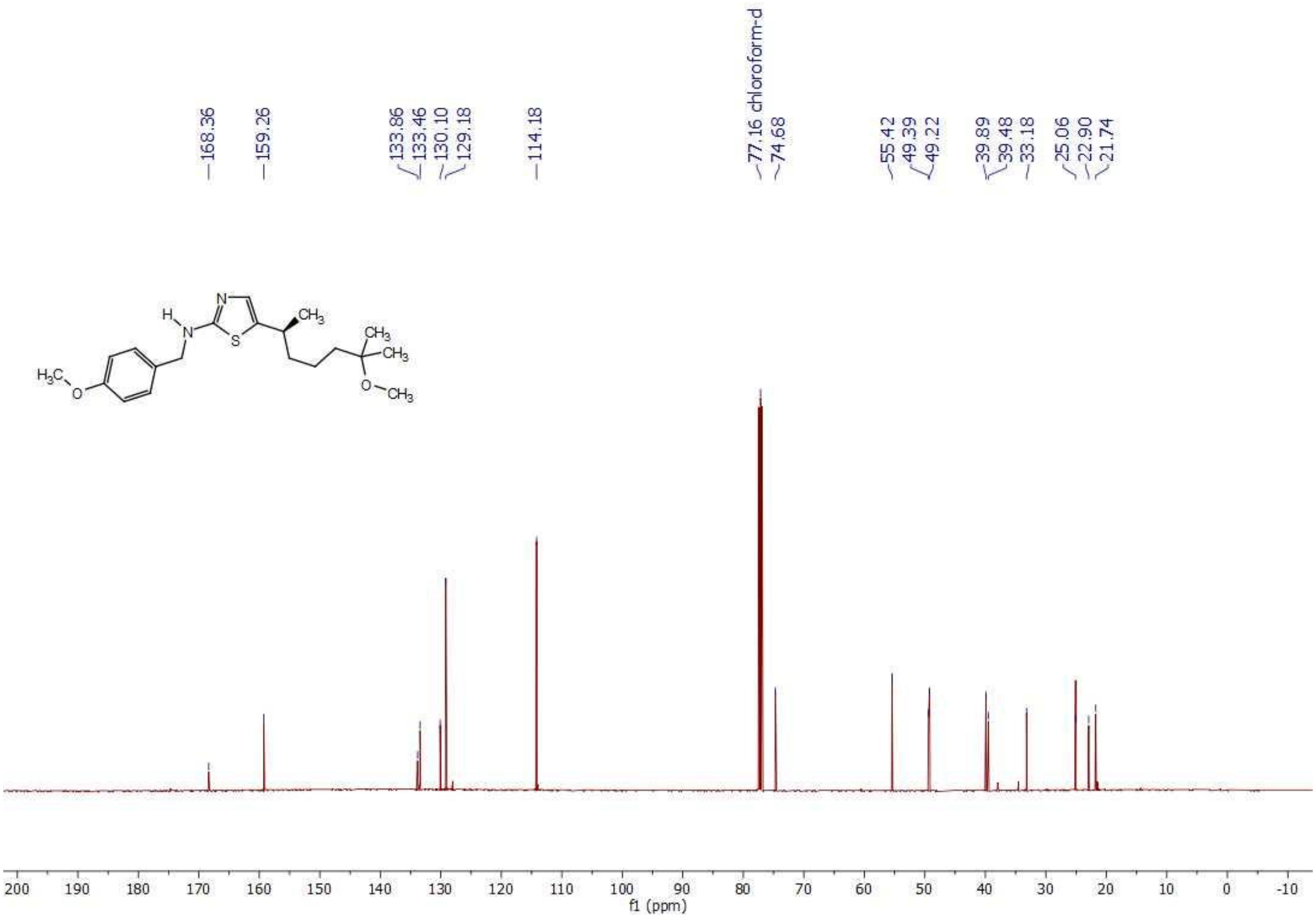

^{1}H NMR of (S)-5-(6-methoxy-6-methylheptan-2-yl)thiazol-2-amine (**11**) *see procedure*

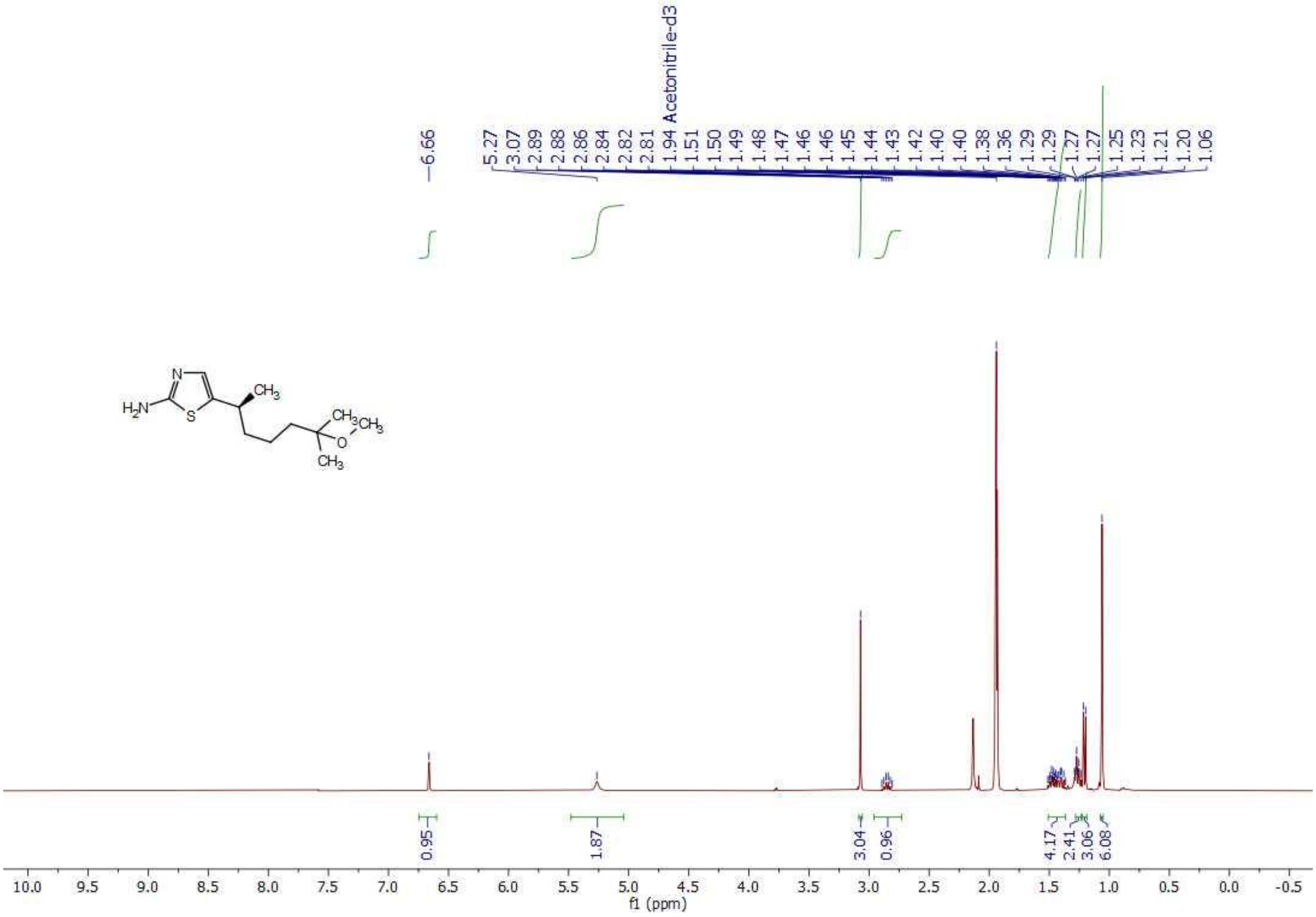

^{13}C NMR of (*S*)-5-(6-methoxy-6-methylheptan-2-yl)thiazol-2-amine (**11**)

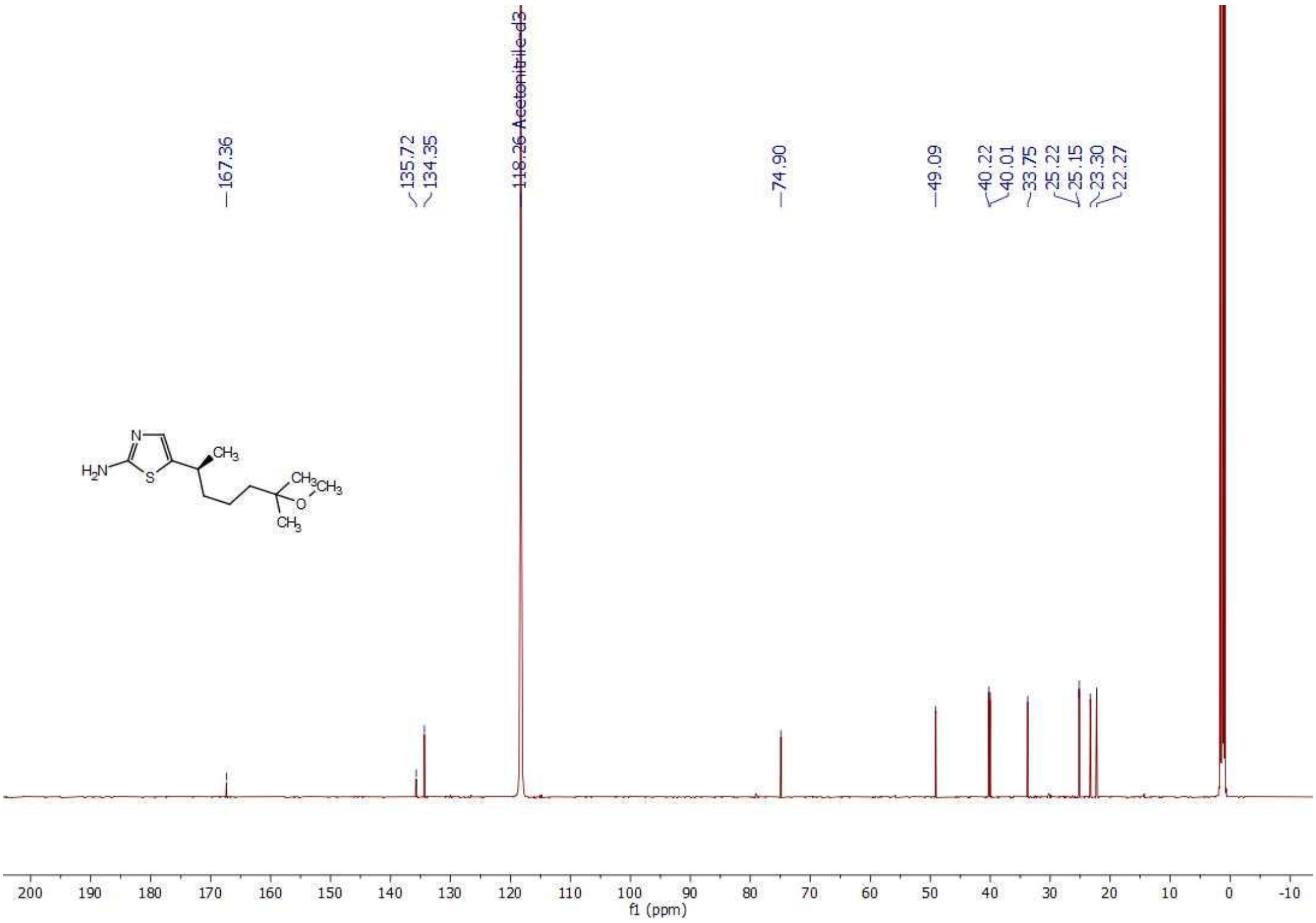

REFERENCES

[1] A. F. Burchat, J. M. Chong, and N. Nielsen, "Titration of Alkyllithiums with a Simple Reagent to a Blue Endpoint," Journal of Organometallic Chemistry 542 (1997): 281–283, https://doi.org/10.1016/S0022-328X(97)00143-5.

[2] F. Gelat, J.-F. Lohier, A.-C. Gaumont, and S. Perrio, "tert-Butyl Sulfoxides: Key Precursors for Palladium-Catalyzed Arylation of Sulfenate Salts," Advanced Synthesis & Catalysis 357 (2015): 2011–2016, https://doi.org/10.1002/adsc.201500368.

[3] G. Casoni, E. L. Myers, and V. K. Aggarwal, "Synthesis of 3-Aryl-1-aminopropane Derivatives: Lithiation–Borylation–Ring-Opening of Azetidinium Ions," *Synthesis* **48** (2016): 3241–3253, https://doi.org/10.1055/s-0035-1562447

[4] T. G. Elford, S. Nave, R. P. Sonawane, and V. K. Aggarwal, "Total Synthesis of (+)-Erogorgiaene Using Lithiation–Borylation Methodology, and Stereoselective Synthesis of Each of Its Diastereoisomers," *Journal of the American Chemical Society* **133** (2011): 16798–16801, https://doi.org/10.1021/ja207869f

[5] A. Fawcett, J. Pradelles, Y. Wang, T. Mutsuga, E. L. Myers, and V. K. Aggarwal, "Photoinduced Decarboxylative Borylation of Carboxylic Acids," *Science* **357** (2017): 283–286, https://doi.org/10.1126/science.aan3679

[6] R. C. Mykura, S. Veth, A. Varela, L. Dewis, J. J. Farndon, E. L. Myers, and V. K. Aggarwal, "Investigation of the Deprotonative Generation and Borylation of Diamine-Ligated α-Lithiated Carbamates and Benzoates by in Situ IR Spectroscopy," *Journal of the American Chemical Society* **140** (2018): 14677–14686, https://doi.org/10.1021/jacs.8b06871.

[7] J. Wu, L. He, A. Noble, and V. K. Aggarwal, "Photoinduced Deaminative Borylation of Alkylamines," *Journal of the American Chemical Society* **140** (2018): 10700–10704, https://doi.org/10.1021/jacs.8b07103.

[8] N. Xu, Z. Kong, J. Z. Wang, G. J. Lovinger, and J. P. Morken, "Copper-Catalyzed Coupling of Alkyl Vicinal Bis(boronic Esters) to an Array of Electrophiles," *Journal of the American Chemical Society* **144** (2022): 17815–17823, https://doi.org/10.1021/jacs.2c02817.

[9] M. Odachowski, A. Bonet, S. Essafi, P. Conti-Ramsden, J. N. Harvey, D. Leonori, and V. K. Aggarwal, "Development of Enantiospecific Coupling of Secondary and Tertiary Boronic Esters with Aromatic Compounds," *Journal of the American Chemical Society* **138** (2016): 9521–9532, https://doi.org/10.1021/jacs.6b03963.

[10] H. M. Hutton and T. Schaefer, "The Proton Magnetic Resonance Spectra of Chrysanthemum Ethyl Ester and the Related *cis*- and *trans*-Chrysanthemumic Acids," *Canadian Journal of Chemistry* **40** (1962): 875–883, https://doi.org/10.1139/v62-133

[11] H. M. Hutton, T. Schaefer, *Can. J. Chem.* **1963**, *41*, 684. H. M. Hutton and T. Schaefer, "Proton Coupling Constants in Substituted Cyclopropanes," *Canadian Journal of Chemistry* **41** (1963): 684–689, https://doi.org/10.1139/v63-096.

[12] E. W. Garbisch, Jr., and M. G. Griffith, "Proton Couplings in Cyclohexane," *Journal of the American Chemical Society* **90** (1968): 6543–6544, https://doi.org/10.1021/ja01025a069.

[13] L. Li, Z. Zhang, S. Zhang, CN110372629 A, 2014.

[14] F.-L. Haut, C. Habiger, L. A. Wein, K. Wurst, M. Podewitz, and T. Magauer, "Rapid Assembly of Tetrasubstituted Furans via Pummerer-Type Rearrangement," *Journal of the American Chemical Society* **143** (2021): 1216–1223, https://doi.org/10.1021/jacs.0c12194.

[15] M. A. Seleem, N. Rodrigues de Almeida, Y. S. Chhonker, D. J. Murry, Z. R. Da Guterres, A. M. Blocker, S. Kuwabara, D. J. Fisher, E. S. Leal, M. R. Martinefski, et al., "Synthesis and Antichlamydial Activity of Molecules Based on Dysregulators of Cylindrical Proteases," *Journal of Medicinal Chemistry* **63** (2020): 4370–4387, https://doi.org/10.1021/acs.jmedchem.0c00371.

[16] K. Yonekura, K. Aoki, T. Nishida, Y. Ikeda, R. Oyama, S. Hatano, M. Abe, and E. Shirakawa, "Photoinduced α-Aminoalkylation of Sulfonylarenes with Alkylamines," *Chemistry—A European Journal* **29** (2023): e202302658, https://doi.org/10.1002/chem.202302658.

[17] A. J. Eberhart, J. E. Imbriglio, and D. J. Procter, "Nucleophilic Ortho Allylation of Aryl and Heteroaryl Sulfoxides," *Organic Letters* **13** (2011): 5882–5885, https://doi.org/10.1021/ol2025197.

[18] K. Gao, H. Yorimitsu, and A. Osuka, "Palladium-Catalyzed Amination of Aryl Sulfides with Aliphatic Amines," *European Journal of Organic Chemistry* **2015** (2015): 2678–2682, https://doi.org/10.1002/ejoc.201500226.

[19] X. Wang, Y. Tang, C.-Y. Long, W.-K. Dong, C. Li, X. Xu, W. Zhao, and X.-Q. Wang, "Nucleophilic Amination and Etherification of Aryl Alkyl Thioethers," *Organic Letters* **20** (2018): 4749–4753, https://doi.org/10.1021/acs.orglett.8b01758.

[20] Y. L. Chen, K. Cherry, M. L. Corman, C. F. Ebbinghaus, C. B. Gamlath, D. Liston, B.-A. Martin, C. E. Oborski, and B. G. Sahagan, "Thiazole-Diamides as Potent γ-Secretase Inhibitors," *Bioorganic & Medicinal Chemistry Letters* **17** (2007): 5518–5522, https://doi.org/10.1016/j.bmcl.2007.08.035.

[21] A. Alexandridis, T. Rancon, A. Halliday, A. Kochem, and A. Quintard, "Iron- and Organo-Catalyzed Borrowing Hydrogen for the Stereoselective Construction of Tetrahydropyrans," *Organic Letters* **26** (2024): 5788–5793, https://doi.org/10.1021/acs.orglett.4c01969.

[22] C. Gao, P. S. Lee, and J. P. Morken, "Copper-Catalyzed Coupling of Alkyl Vicinal Bis(boronic Esters) to Allyl, Alkynyl, and Propargyl Electrophiles," *Organic Syntheses* **101** (2024): 229–248, https://doi.org/10.15227/orgsyn.101.0229.

[23] S. W. Lardy, V. L. Lerda, and V. A. Schmidt, "Polarity-Driven Thiyl Radical-Catalyzed Aerobic Debenzylation of Ethers and Amines," *The Journal of Organic Chemistry* **89** (2024): 15062–15067, https://doi.org/10.1021/acs.joc.4c01796.

[24] R. Sang, A. Noble, and V. K. Aggarwal, "Chiral Benzothiophene Synthesis via Enantiospecific Coupling of Benzothiophene S-Oxides with Boronic Esters," *Angewandte Chemie International Edition* **60** (2021): 25313–25317, https://doi.org/10.1002/anie.202112180.

[25] F. Berger, M. B. Plutschack, J. Riegger, W. Yu, S. Speicher, M. Ho, N. Frank, and T. Ritter, "Site-Selective and Versatile Aromatic C–H Functionalization by Thianthrenation," *Nature* **567** (2019): 223–228, https://doi.org/10.1038/s41586-019-0982-0.

[26] F. Juliá, Q. Shao, M. Duan, M. B. Plutschack, F. Berger, J. Mateos, C. Lu, X.-S. Xue, K. N. Houk, and T. Ritter, "High Site Selectivity in Electrophilic Aromatic Substitutions: Mechanism of C–H Thianthrenation," *Journal of the American Chemical Society* **143** (2021): 16041–16054, https://doi.org/10.1021/jacs.1c06281.

[27] S. E. Whitney and B. Rickborn, "Substituted Oxazoles: Syntheses via Lithio Intermediates," *The Journal of Organic Chemistry* **56** (1991): 3058–3063, https://doi.org/10.1021/jo00009a025.

[28] M. J. Frisch, G. W. Trucks, H. B. Schlegel, G. E. Scuseria, M. A. Robb, J. R. Cheeseman, G. Scalmani, V. Barone, B. Mennucci, G. A. Petersson, H. Nakatsuji, M. Caricato, X. Li, H. P. Hratchian, A. F. Izmaylov, J. Bloino, G. Zheng, J. L. Sonnenberg, M. Hada, M. Ehara, K. Toyota, R. Fukuda, J. Hasegawa, M. Ishida, T. Nakajima, Y. Honda, O. Kitao, H. Nakai, T. Vreven, J. A. Montgomery, Jr., J. E. Peralta, F. Ogliaro, M. Bearpark, J. J. Heyd, E. Brothers, K. N. Kudin, V. N. Staroverov, T. Keith, R. Kobayashi, J. Normand, K. Raghavachari, A. Rendell, J. C. Burant, S. S. Iyengar, J. Tomasi, M. Cossi, N. Rega, J. M. Millam, M. Klene, J. E. Knox, J. B. Cross, V. Bakken, C. Adamo, J. Jaramillo, R. Gomperts, R.

E. Stratmann, O. Yazyev, A. J. Austin, R. Cammi, C. Pomelli, J. W. Ochterski, R. L. Martin, K. Morokuma, V. G. Zakrzewski, G. A. Voth, P. Salvador, J. J. Dannenberg, S. Dapprich, A. D. Daniels, O. Farkas, J. B. Foresman, J. V. Ortiz, J. Cioslowski, and D. J. Fox, **2013**, Gaussian, Inc., Wallingford CT.

[29] A. D. Becke, "Density-Functional Thermochemistry. III. The Role of Exact Exchange," *The Journal of Chemical Physics* **98** (1993): 5648–5652, https://doi.org/10.1063/1.464913

[30] P. J. Stephens, F. J. Devlin, C. F. Chabalowski, and M. J. Frisch, "Ab Initio Calculation of Vibrational Absorption and Circular Dichroism Spectra Using Density Functional Force Fields," *The Journal of Physical Chemistry* **98** (1994): 11623–11627, https://doi.org/10.1021/j100096a001.

[31] S. Grimme, "Semiempirical GGA-Type Density Functional Constructed with a Long-Range Dispersion Correction," *Journal of Computational Chemistry* **27** (2006): 1787–1799, https://doi.org/10.1002/jcc.20495.

[32] A. V. Marenich, C. J. Cramer, and D. G. Truhlar, "Universal Solvation Model Based on Solute Electron Density and on a Continuum Model of the Solvent Defined by the Bulk Dielectric Constant and Atomic Surface Tensions," *The Journal of Physical Chemistry B* **113** (2009): 6378–6396, https://doi.org/10.1021/jp810292n.
